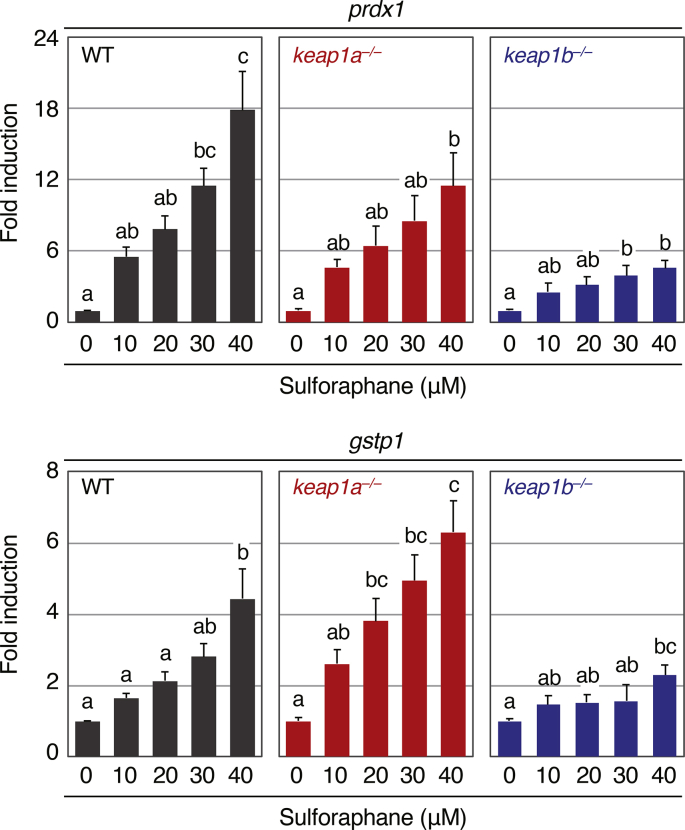# Generation and characterization of *keap1a-* and *keap1b*-knockout zebrafish

**DOI:** 10.1016/j.redox.2020.101667

**Published:** 2020-08-11

**Authors:** Vu Thanh Nguyen, Lixuan Bian, Junya Tamaoki, Shiro Otsubo, Masafumi Muratani, Atsuo Kawahara, Makoto Kobayashi

**Affiliations:** a Department of Molecular and Developmental Biology, Faculty of Medicine, University of Tsukuba, 1-1-1 Tennodai, Tsukuba, 305-8575, Japan; b Division of Aquaculture Biotechnology, Biotechnology Center of Ho Chi Minh City, Ho Chi Minh City, Viet Nam; c Department of Genome Biology, Faculty of Medicine, University of Tsukuba, 1-1-1 Tennodai, Tsukuba, 305-8575, Japan; d Laboratory for Developmental Biology, Center for Medical Education and Sciences, Graduate School of Medical Science, University of Yamanashi, Chuo, Yamanashi, 409-3898, Japan

**Keywords:** Antioxidant activity, Keap1–Nrf2 pathway, Knockout zebrafish, Oxidative stress, Sulforaphane

## Abstract

The Keap1–Nrf2 pathway is an evolutionarily conserved mechanism that protects cells from oxidative stress and electrophiles. Under homeostatic conditions, Keap1 interacts with Nrf2 and leads to its rapid proteasomal degradation, but when cells are exposed to oxidative stress/electrophiles, Keap1 senses them, resulting in an improper Keap1–Nrf2 interaction and Nrf2 stabilization. Keap1 is therefore considered both an “inhibitor” of and “stress sensor” for Nrf2 activation. Interestingly, fish and amphibians have two Keap1s (Keap1a and Keap1b), while there is only one in mammals, birds and reptiles. A phylogenetic analysis suggested that mammalian Keap1 is an ortholog of fish Keap1b, not Keap1a. In this study, we investigated the differences and similarities between Keap1a and Keap1b using zebrafish genetics. We generated zebrafish knockout lines of *keap1a* and *keap1b*. Homozygous mutants of both knockout lines were viable and fertile. In both mutant larvae, the basal expression of Nrf2 target genes and antioxidant activity were up-regulated in an Nrf2-dependent manner, suggesting that both Keap1a and Keap1b can function as Nrf2 inhibitors. We also analyzed the effects of the Nrf2 activator sulforaphane in these mutants and found that *keap1a-*, but not *keap1b-*, knockout larvae responded to sulforaphane, suggesting that the stress/chemical-sensing abilities of the two Keap1s are different.

## Highlights

• Fish and amphibians have two Keap1s: Keap1a and Keap1b.
• Mammalian Keap1 is an ortholog of fish Keap1b, not Keap1a.
• Both Keap1a and Keap1b can function as Nrf2 inhibitors.
• The sulforaphane-sensing abilities of Keap1a and Keap1b are different.

## Graphical abstract

**Figure:**
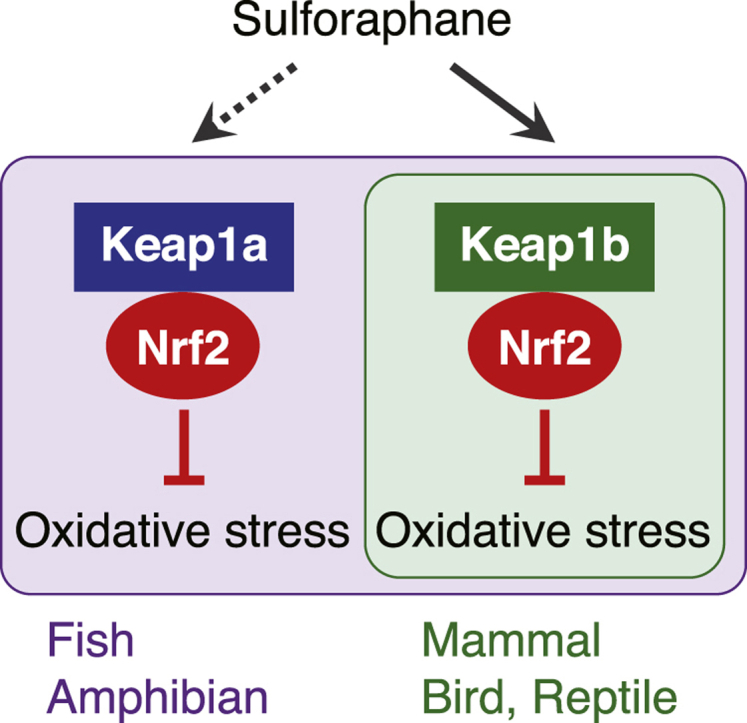

## 1 Introduction

The Kelch-like ECH-associated protein 1 (Keap1)–NF-E2 p45-related factor 2 (Nrf2) pathway is an evolutionarily conserved mechanism that protects cells against oxidative stress and electrophilic xenobiotics [[1], [2], [3], [4]]. Nrf2 is a transcription factor that transactivates a variety of cytoprotective genes in response to many types of insults/stresses. The physiological importance of the Nrf2-dependent cytoprotection has been demonstrated in Nrf2-deficient mice [5], rats [6] and zebrafish [7]. Keap1 is an Nrf2-specific adaptor protein for the Cullin 3-E3 ubiquitin ligase that binds to the Nrf2-ECH homology 2 (Neh2) domain of Nrf2 and facilitates its ubiquitination and proteasomal degradation, leading to the down-regulation of Nrf2 target genes under homeostatic conditions [[8], [9], [10], [11]]. In addition to this negative function in the Nrf2-dependent gene regulation, Keap1 also plays a role as a sensor for various Nrf2-activating electrophiles and oxidative stress using its reactive cysteine residues [[12], [13], [14], [15]].

We have been studying the Keap1–Nrf2 pathway in zebrafish and found that its physiological roles and molecular mechanisms are quite similar to those in mice, suggesting that the Keap1–Nrf2 pathway is highly conserved among vertebrates [7,[16], [17], [18]]. Keap1 is a single gene in mammals, but there are two co-orthologs (*keap1a* and *keap1b*) in zebrafish [16,19].

In the present study, we generated and characterized *keap1a*- and *keap1b-*knockout zebrafish and compared their phenotypes to elucidate the differences and similarities between Keap1a and Keap1b as “inhibitors” of and “stress sensors” for Nrf2 activation.

## 2 Materials and methods

### 2.1 Zebrafish and chemicals

AB (wild-type), *nfe2l2a*^*fh318*^ [7], *keap1a*^*it302*^ and *keap1b*^*it308*^ strains were used. For genotyping *keap1a*^*it302*^ and *keap1b*^*it308*^, polymerase chain reaction (PCR) was carried out using the following primer sets: 5′-CTGCTTGAGCTGATCAGTCAGG and 5′-CGGCTCTCTGCGTCCCAG (*keap1a*^*it302*^) and 5′-TCAGCGGCCTGCTGTACG and 5′-CGGGCCCATTGTTGCGTC (*keap1b*^*it308*^). Genotyping of *nfe2l2a*^*fh318*^ was performed as previously described [20]. Before using for experiments in this paper, we backcrossed *keap1a*^*it302*^ and *keap1b*^*it308*^ lines with wild-type AB strain more than 4 times after their generation. Both lines are available from the National BioResource Project of Japan (https://shigen.nig.ac.jp/zebra/index_en.html). Sulforaphane and hydrogen peroxide (H_2_O_2_) were purchased from LKT Laboratories (St. Paul, MN) and Wako (Osaka, Japan), respectively.

All animal experiments were performed in accordance with the animal protocol approved by the Animal Experiment Committee of the University of Tsukuba. All methods were carried out in accordance with the Regulation for Animal Experiments in our university and Fundamental Guideline for Proper Conduct of Animal Experiment and Related Activities in Academic Research Institutions under the jurisdiction of the Ministry of Education, Culture, Sports, Science and Technology of Japan.

### 2.2 Phylogenetic analyses

The phylogenetic tree was constructed using the CLUSTAL W program (http://clustalw.ddbj.nig.ac.jp) and plotted with NJplot (http://doua.prabi.fr/software/njplot).

### 2.3 Gene knockout

Knockout lines of zebrafish *keap1a* and *keap1b* were generated using the clustered regularly interspaced short palindromic repeats (CRISPR)–CRISPR-associated sequences 9 (Cas9) technology as previously described [21]. In brief, guide RNAs for *keap1a*- or *keap1b*-specific (25 pg each) and Cas9 mRNA (250 pg) were co-injected into the yolk of single-cell-stage wild-type AB embryos. Plasmids for the guide RNAs were constructed using the plasmid vector pDR274 (Addgene, Watertown, MA, USA) and oligonucleotides: 5′-taggGCGAGAGCGAGGTCTACA and 5′-aaacTGTAGACCTCGCTCTCGC (*keap1a*); 5′-taggCCTGCTGTACGCCGTGGG and 5′-aaacCCCACGGCGTACAGCAGG (*keap1b*). RNAs were transcribed using the T7 MAXIscript Kit (Ambion, Austin, TX, USA), and Cas9 mRNA was synthesized by the mMESSAGE mMACHINE SP6 kit (Ambion) using pCS2+hspCas9 (Addgene) as a template.

### 2.4 RNA sequence (RNA-seq) analyses

Total RNA was extracted from 5-days-post-fertilization (dpf) larvae of *keap1a*- and *keap1b*-knockout lines and wild-type AB treated with or without sulforaphane using ISOGEN II (Nippon Gene, Tokyo, Japan). Larvae were treated with sulforaphane from 4.5 dpf to 5 dpf (12 h). RNA-seq library was constructed with 500 ng of total RNA using NEBNext Poly(A) mRNA Magnetic Isolation Module (New England Biolabs, Beverly, MA, USA) and NEBNext Ultra Directional RNA Library Prep Kit for Illumina (New England Biolabs). Libraries were validated using Bioanalyzer (Agilent Technologies, Santa Clara, CA, USA) to determine the size distribution and concentration. Sequencing was performed by Tsukuba i-Laboratory LLP using NextSeq500 (Illumina, San Diego, CA, USA) with a paired-end 36-base read option. Sequencing reads in FASTQ format were imported to CLC Genomics Workbench (CLC-GW, ver. 10.1.1, Qiagen, Hilden, Germany) and mapped on the zebrafish reference genome assembly GRCz10. Reads were quantified for 31,701 genes in ENSEMBLE annotation provided in CLC-GW, and quantified reads per kilobase per million mapped reads (RPKM) value were obtained. RPKM values from 4 samples (untreated and sulforaphane-treated wild-type AB, untreated *keap1a*-knockout, untreated *keap1b*-knockout) were normalized using Normalization Tool in CLC-GW with mean-scaling, with median of means as reference value, 5% trimming option settings. Fold-changes (FC) of normalized RPKM values were calculated between two samples, and differentially expressed genes were identified by threshold of |FC|>1.5. A gene ontology (GO) analysis was carried out using DAVID 6.8 with GOTERM_BP_DIRECT (BP: biological processes) [22] after converting identified zebrafish genes into their human homologs using bioDBnet [23] and ZFIN (https://zfin.org/).

### 2.5 Gene expression analyses

Quantitative real-time PCR (qPCR) was carried out as previously described [17]. For qPCR analysis, larvae were treated with sulforaphane for 6 h at 5 dpf. Primer sets for *keap1a* and *keap1b* are as follows: 5′-GTGCATAAGCTGGTTCTGGC and 5′-GACTTGAGGACAAACGTCTC (*keap1a*); 5′-AGTAACGCCATCGGCATCG and 5′-TGAAGAACTCCTCCTGCGTC (*keap1b*). Primers for *prdx1* and *gstp1* were described previously [17].

### 2.6 Survival assays

Survival assays were performed as previously described [24]. At 4 dpf, larvae were exposed to 2 mM of H_2_O_2_ for 120 h. Sulforaphane was administered at 3.5 dpf, 12 h prior to H_2_O_2_ exposure. Dead larvae were collected and stored at –20 °C until genotyping together with surviving larvae.

### 2.7 Statistical analyses

The survival data were calculated using the Kaplan–Meier method and analyzed by the log-rank test. The statistical significance of gene induction was determined by two-tailed *t*-test. Comparison of gene induction levels between different genotypes was performed using a one-way analysis of variance followed by Bonferroni's multiple comparisons test. All statistical analyses were performed using EZR [25], which is a graphical user interface for R (The R Foundation for Statistical Computing, Vienna, Austria). *P* values of <0.05 were considered to be statistically significant and indicated with asterisks (*p < 0.05, **p < 0.01, ***p < 0.001).

## 3 Results

### 3.1 Keap1b is an authentic ortholog of mammalian Keap1, and Keap1a is a fish/amphibian-specific gene

We initially believed that the Keap1a and Keap1b genes are restricted to fish that had been generated by the teleost-specific whole genome duplication (WGD) [26,27](Fig. 1A, green rectangle), but our assumption was wrong. In addition to teleosts, Keap1a/Keap1b are also present in non-teleost fish, such as polypteri, sharks and coelacanths, and even in anuran and urodele amphibians (Fig. 1B). Intriguingly, phylogenetic analyses showed that mammalian Keap1 belongs to the Keap1b subfamily, and no reptiles, birds and mammals have Keap1a genes, suggesting that both Keap1a and Keap1b were conserved during the evolution process from fish to tetrapods, with Keap1a subsequently lost during further evolution to amniotes (Fig. 1A). These findings led us to hypothesize that Keap1b is an authentic ortholog of mammalian Keap1, while Keap1a is a fish/amphibian-specific gene that may have unique roles that Keap1/Keap1b do not have.

**Fig. 1:**
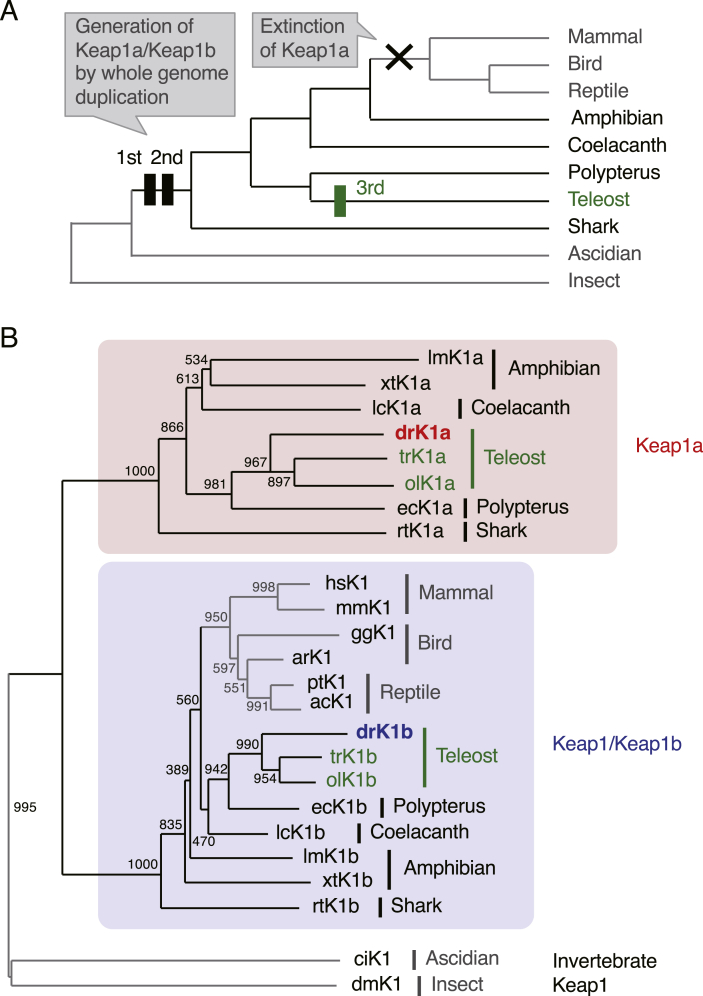
**The comparison of Keap1 proteins.** (A) Simplified phylogeny of vertebrate and invertebrate animals. Black and green rectangles represent two WGD events that occurred early in vertebrate evolution and an additional WGD in the teleost ancestor, respectively. (B) Phylogenetic tree of Keap1 family proteins. Amino acid sequences in the broad complex, tramtrack and bric-a-brac domains–intervening region (BTB–IVR) were obtained from GenBank (http://www.ncbi.nlm.nih.gov/) and NewtBase (http://newtbase.eko.uj.edu.pl/). Accession numbers: lmK1a, c152694_g1_i1 (NewtBase contig); xtK1a, XM_012953851; lcK1a, XM_014486756; drK1a, NM_182864; trK1a, XM_011618924; olK1a, XM_004082216; ecK1a, XM_028816380; rtK1a, XM_020523471; hsK1, NM_203500; mmK1, NM_016679; ggK1, KU321503; arK1, XM_026068028; ptK1, XM_026711369; acK1, XM_003216399; drK1b, NM_001113477; trK1b, XM_003972594; olK1b, XM_023955710; ecK1b, XM_028823679; lcK1b, XM_005994311; lmK1b, c118729_g1_i1 (NewtBase contig); xtK1b, NM_001008023; rtK1b, XM_020519602; ciK1, XM_002128019; dmK1, NM_142337. Abbreviations: ac, *Anolis carolinensis* (lizard); ar, *Apteryx rowi* (kiwi); ci, *Ciona intestinalis* (ascidian); dm, *Drosophila melanogaster* (fly); dr, *Danio rerio* (zebrafish); ec, *Erpetoichthys calabaricus* (snakefish); gg, *Gallus gallus* (chicken); hs, *Homo sapiens* (human); lc, *Latimeria chalumnae* (coelacanth); lm, *Lissotriton montandoni* (newt); mm, *Mus musculus* (mouse); ol, *Oryzias latipes* (medaka); pt, *Pseudonaja textilis* (snake); rt, *Rhincodon typus* (shark); tr, *Takifugu rubripes* (pufferfish); xt, *Xenopus tropicalis* (frog). Of note, all 74 teleosts in the Ensembl Genome Browser have both Keap1a and Keap1/Keap1b genes, while all 104 mammals, 13 birds and 11 reptiles have only Keap1/Keap1b. (For interpretation of the references to colour in this figure legend, the reader is referred to the Web version of this article.)

### 3.2 keap1a- and keap1b-knockout zebrafish were viable and fertile

**Fig. 2:**
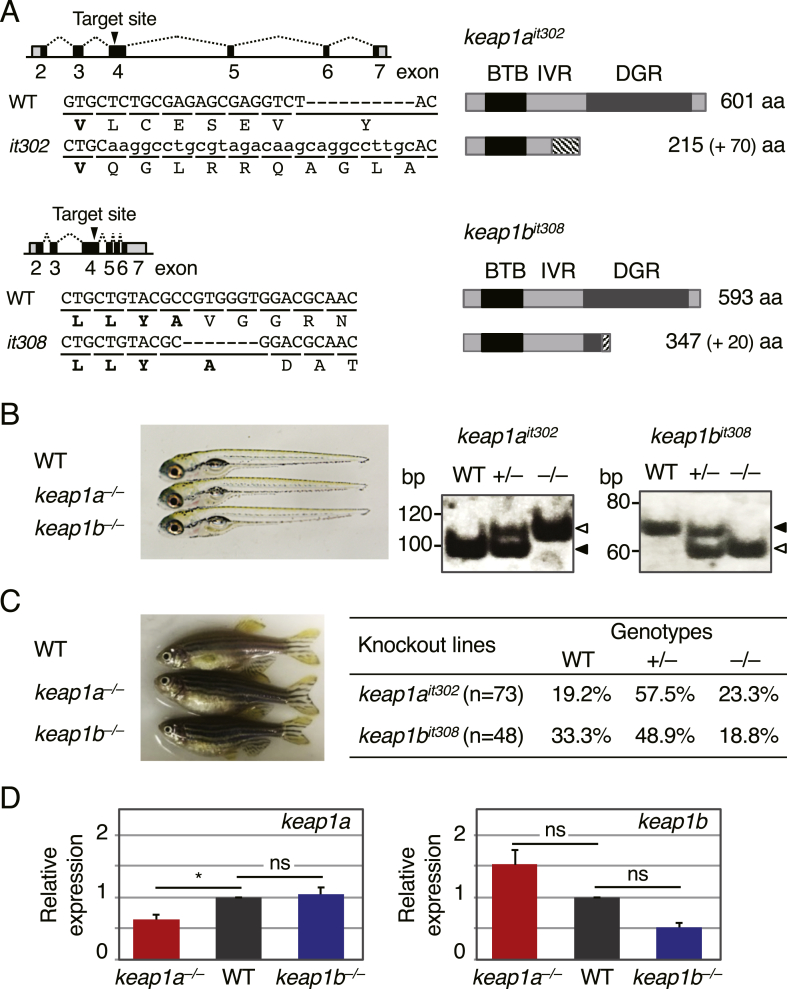
**The generation of*****keap1a-*****and*****keap1b-*****knockout zebrafish.** (A) Gene knockout of *keap1a* and *keap1b* using CRISPR–Cas9 technology. CRISPR target sites were designed in exon 4 of the *keap1a* and *keap1b* loci (black arrowheads). In *keap1a*^*it302*^ and *keap1b*^*it308*^ lines, 70- and 20-amino acids-extra peptides were added after the original Val215 in Keap1a and after the original Ala347 in Keap1b proteins, respectively (diagonal stripes). (B) *keap1a-* and *keap1b*-homogygous knockout larvae at 5 dpf. No obvious differences were observed between wild-type and knockout larvae. Right panels show PCR genotyping of *keap1a*^*it302*^-and *keap1b*^*it308*^-knockout larvae. WT, +/– and −/− indicate wild-type, heterozygous and homozygous fish, respectively. Open and closed arrowheads denote knockout and wild-type alleles, respectively. (C) *keap1a*^*it302*^-and *keap1b*^*it308*^-homozygous knockout adults at 4 mpf. No obvious differences were observed between wild-type and knockout adults (females in this picture). The genotypes of 4-mpf adults derived from heterozygous parents were roughly according to the expected Mendelian ratio. (D) The relative expression of *keap1a* and *keap1b* in wild-type, homozygous *keap1a*^*it302*^-and *keap1b*^*it308*^-knockout larvae at 5 dpf analyzed by qPCR. The expression of wild-type specimens was normalized to 1. Each experiment was conducted at least four times with duplicate samples. Asterisks indicate significant differences (*p < 0.05). ns, not significant.

To investigate the differences and similarities between Keap1a and Keap1b, we generated *keap1a-* and *keap1b-*knockout zebrafish lines using CRISPR–Cas9 technology [28,29]. Target sites for CRISPR–Cas9 were designed in exon 4, which led to the deletion of the Nrf2-interacting diglycine repeat (DGR) domains in both Keap1a and Keap1b (Fig. 2A). Two mutant lines, *keap1a*^*it302*^ and *keap1b*^*it308*^, were generated: the *keap1a*^*it302*^ line has an 18-base pair (bp) deletion and 28-bp insertion, and the *keap1b*^*it308*^ line has a 7-bp deletion in the CRISPR target sites, resulting in the C-terminal deletion of Keap1a after Val215 and that of Keap1b after Ala347 due to frameshift mutations (Fig. 2A).

As shown in Fig. 2B, no obvious abnormality was found in either the *keap1a*- or *keap1b*-homozygous knockout larvae at 5 dpf derived from heterozygous parents. For genotyping, we designed specific PCR primers to amplify 10-bp-longer and 7-bp-shorter PCR products for knockout alleles compared with the wild-type allele (Fig. 2B, right). We raised these larvae to adulthood and then genotyped them at four months-post fertilization (mpf). The results showed that the genotypes of both knockout adults were roughly according to the expected Mendelian ratio, suggesting that the homozygous knockout adults were both viable (Fig. 2C, right). No obvious difference was found between homozygous knockout mutant and wild-type in both larval (Fig. 2B, left) and adult stages (Fig. 2C, left). To eliminate the possibility of genetic compensation of deleterious gene mutations by the up-regulation of their paralog genes [30], we next examined the gene expression of *keap1a* and *keap1b* in *keap1b*- and *keap1a*-homozygous knockout larvae (Fig. 2D). Homozygous knockout larvae used in this experiment were derived from homozygous parents, suggesting that both *keap1a*- and *keap1b*-knockout lines were fertile. The expression of *keap1a* and *keap1b* in *keap1b*- and *keap1a*-knockout larvae, respectively, was not significantly different from that in wild-type animals, implying that the phenotypes (viable and fertile) of *keap1a-* and *keap1b*-knockout zebrafish were not due to genetic compensation for each other.

### 3.3 The effects of keap1a- and keap1b-disruption on gene expression profiles were similar

**Fig. 3:**
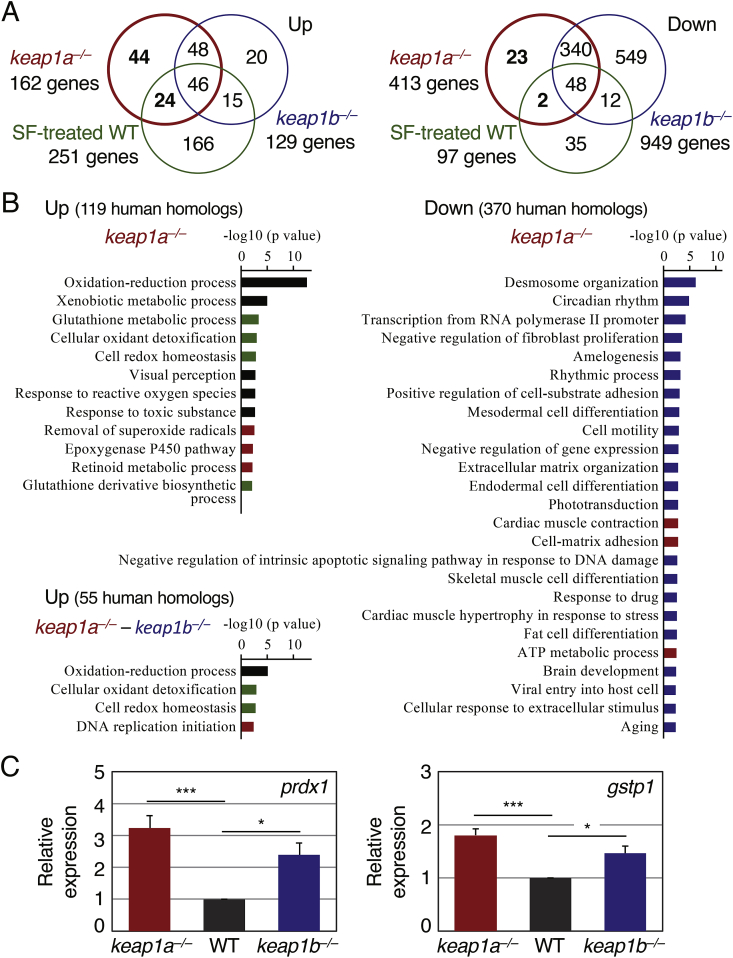
**Altered genes and pathways in *keap1a*- and *keap1b*-knockout larvae.** (A) Venn diagrams showing up- (left) and down-regulated genes (right) in 5-dpf larvae of *keap1a*^*it302*^-and *keap1b*^*it308*^-homozygous knockout larvae and of wild-type AB larvae treated with 40 μM sulforaphane identified by an RNA-seq analysis in comparison with untreated AB larvae. (B) A GO enrichment analysis of biological processes in *keap1a*- and *keap1b-*knockout larvae. Red, blue, green and black bars indicate the processes enriched in only *keap1a*-knockout larvae, both *keap1a*- and *keap1b*-knockout larvae, both *keap1a*-knockout and sulforaphane-treated AB larvae, and all three larvae, respectively. (C) The comparison of the basal expression of Nrf2 target genes between wild-type and Keap1 knockout larvae. The expression of *prdx1* and *gstp1* in wild-type AB, *keap1a*- and *keap1b*-knockout larvae at 5 dpf was analyzed by qPCR. The expression in wild-type specimens was normalized to 1. Each experiment was conducted at least four times with duplicate samples. Asterisks indicate significant differences (*p < 0.05, ***p < 0.001). (For interpretation of the references to colour in this figure legend, the reader is referred to the Web version of this article.)

To detect specific molecular alterations in *keap1a*-knockout zebrafish, we performed a whole-transcriptome analysis by RNA-seq. When 5-dpf *keap1a*- and *keap1b*-knockout larvae were compared against wild-type AB larvae in standard condition (Table S1), 162 and 129 genes were up-regulated in *keap1a*- and *keap1b*-knockout larvae, while 413 and 949 genes were down-regulated, respectively (Fig. 3A, red and blue circles, 1.5-fold). Out of 162 up- and 413 down-regulated genes in *keap1a*-knockout larvae, 94 (58.0%) and 388 (93.9%) overlapped with those in *keap1b*-knockout larvae, respectively, suggesting that most of the affected genes were common between the two types of knockout larvae, especially in the case of down-regulated genes.

To compare the effects of Keap1 disruption and Nrf2 activation, we further performed an RNA-seq analysis of wild-type AB larvae in the presence of sulforaphane, an isothiocyanate found abundantly in broccoli sprouts and known to be a potent Nrf2 activator [31]. The analysis identified 251 up- and 97 down-regulated genes (Fig. 3A, green circles, 1.5-fold) by sulforaphane-treatment. 43.2% (70 genes) and 12.1% (50 genes) of those genes were also altered by *keap1a*-knockout larvae, respectively.

To identify and compare the biological processes that were altered by *keap1a* disruption, *keap1b* disruption or sulforaphane treatment, a GO analysis was performed using DAVID bioinformatics resources [22](Tables S2–S7). Fig. 3B shows the major biological processes that were significantly enriched by *keap1a*-disruption (*p* < 0.01). Of the 162 up- and 413 down-regulated genes in *keap1a*-knockout larvae, 119 (73.4%) and 370 (89.6%) genes were mapped to human homologs, respectively, and used for the GO analysis. The analysis of the up-regulated genes demonstrated that affected biological processes were mainly downstream targets of Nrf2 [4,17,32], such as oxidation-reduction process and xenobiotic metabolic process, which were common in all *keap1a*-, *keap1b-*knockout and sulforaphane-treated larvae (black bars), and the glutathione metabolic process and cellular oxidant detoxification, which were common in *keap1a*-knockout and sulforaphane-treated larvae (green bars)(see also Tables S2–S4). The enriched biological processes in the GO analysis of the down-regulated genes were markedly similar between *keap1a*- and *keap1b-*knockout larvae (blue bars)(see also Tables S5–S7), and the processes that have been shown to be related to Nrf2, such as desmosome organization and circadian rhythm [[33], [34], [35]], were high ranked. Some biological processes were found to be specific to *keap1a-*knockout larvae (red bars). To identify the processes only related to *keap1a*, we analyzed the genes that were up-regulated in *keap1a-* but not in *keap1b-*knockout larvae (Fig. 3B, *keap1a*^*–/–*^ – *keap1b*^*–/–*^), but target processes of Nrf2 still seemed to account for the majority, except DNA replication initiation (Tables S8 and S9).

Taken together, these results suggested that a major role of *keap1a* in zebrafish larvae is to inhibit Nrf2 functions, which is quite similar to that of *keap1b*. To compare the contribution of *keap1a* and *keap1b* in suppressing Nrf2 activities, the expression of *peroxiredoxin 1* (*prdx1*) and *glutathione S-transferase P1* (*gstp1*) was examined by qPCR (Fig. 3C). These are two typical target genes of zebrafish Nrf2 that were identified by an RNA-seq analysis under all three conditions here, namely the results of RNA-seq analyses using *keap1a* knockout larvae, *keap1b*-knockout larvae, and sulforaphane-treated wild-type larvae, as well as by a microarray analysis using diethyl maleate-treated 4-dpf larvae [36] and Nrf2-overexpressing embryos at 8 h post-fertilization [17]. Compared with wild-type AB, the expression of *prdx1* and *gstp1* was significantly higher in *keap1a*- (*prdx1*: 3.2-fold; *gstp1*: 1.8-fold) and *keap1b*- (*prdx1*: 2.4-fold; *gstp1*: 1.5-fold) knockout larvae. The results indicated that both zebrafish Keap1a and Keap1b contribute to Nrf2 inhibition in a similar manner at the larval stage.

### 3.4 The antioxidant activity was up-regulated in both keap1a- and keap1b-knockout larvae in an Nrf2-dependent manner

**Fig. 4:**
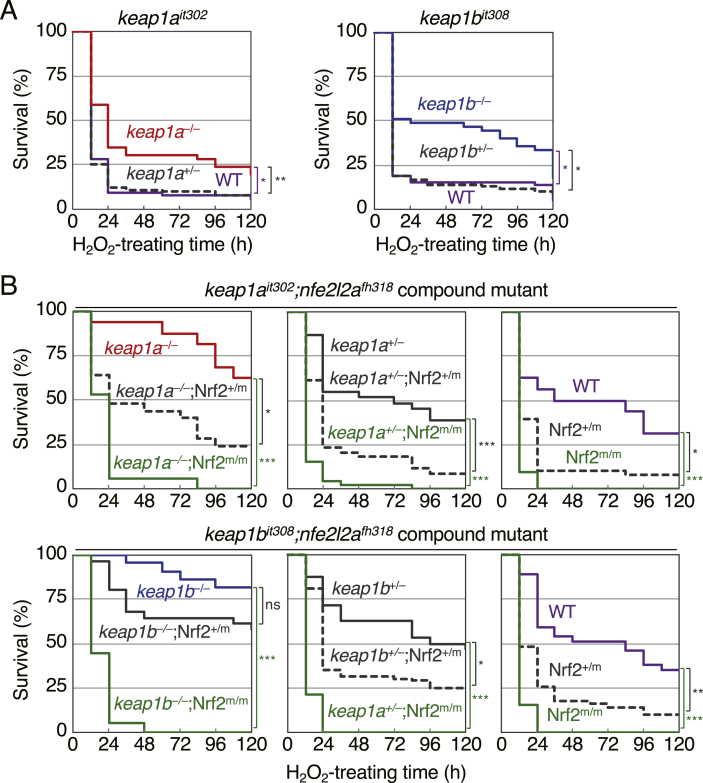
**Survival assays of*****keap1a-*****and*****keap1b-*****knockout larvae against H**_**2**_**O**_**2**_**.** (A) The survival rate of *keap1a*^*it302*^ and *keap1b*^*it308*^ larvae after exposure to H_2_O_2_. At 4 dpf, larvae were exposed to 2 mM of H_2_O_2_, and the survival was observed every 12 h until 9 dpf. Tested numbers were as follows: *keap1a*^*it302*^ (WT n = 53, +/– n = 99, −/− n = 46), *keap1b*^*it308*^ (WT n = 59, +/– n = 107, −/− n = 45). Data from four independent experiments were combined. (B) The survival rate of *keap1a*^*it302*^;*nfe2l2a*^*fh318*^ and *keap1b*^*it308*^;*nfe2l2a*^*fh318*^ compound mutant larvae. Tested numbers were follows: *keap1a*^*it302*^;*nfe2l2a*^*fh318*^ (−/−;WT n = 16, −/−; +/m n = 25, −/−;m/m n = 17, –/+; WT n = 31, –/+; m/+ n = 94, –/+; m/m n = 45, WT; WT n = 16, WT; +/m n = 38, WT; m/m n = 21), *keap1b*^*it308*^;*nfe2l2a*^*fh318*^ (−/−;WT n = 22, −/−; +/m n = 31, −/−;m/m n = 18, –/+; WT n = 32, –/+; m/+ n = 79, –/+; m/m n = 37, WT; WT n = 37, WT; +/m n = 50, WT; m/m n = 26). Data from four independent experiments were combined. Asterisks indicate significant differences (*p < 0.05, **p < 0.01, ***p < 0.001).

The up-regulation of Nrf2 target gene expression under basal conditions suggests that the antioxidant activity may also be up-regulated in *keap1a*- and *keap1b*-knockout larvae. To test this possibility, the effects of *keap1a*- and *keap1b*-knockout on sensitivities against oxidative stress was analyzed by a survival assay. We previously showed that the activation of Nrf2 up-regulated the survival rates of zebrafish larvae against H_2_O_2_ [7]. Larvae at 4 dpf were treated with 2 mM H_2_O_2_, and their survival was analyzed using the Kaplan-Meier method (Fig. 4A). For *keap1a*-knockout mutants, 90% of heterozygous mutants and wild-type larvae died within 24 h after H_2_O_2_ treatment, while 25%–30% of homozygous mutants were survived. Similarly, more than 80% of heterozygous mutants and wild-type larvae died among *keap1b-*knockout mutants, while 30%–50% of homozygous mutants survived. These results suggest that the antioxidant activity is up-regulated in *keap1a*- and *keap1b*-knockout larvae.

We assumed that the up-regulated antioxidant activities in *keap1a*- and *keap1b*-knockout larvae were due to the up-regulation of the Nrf2 activity. To confirm this assumption, we examined the effects of Nrf2 mutation on antioxidant activities in *keap1a*- and *keap1b*-knockout larvae by generating and using compound mutant lines with *nfe2l2a*^*fh318*^ (*nfe2l2a*: zebrafish ortholog of mammalian Nrf2 gene) [7]. As shown in Fig. 4B, the survival rates against H_2_O_2_ toxicity of both *keap1a*- and *keap1b*-knockout larvae were markedly reduced by introducing an Nrf2 mutation, indicating that the up-regulated antioxidant activities in these knockout mutants were Nrf2-dependent. All of these results suggest that Keap1a and Keap1b have similar Nrf2-inhibitory activities *in vivo.*

### 3.5 Keap1b showed significantly higher sensing ability to sulforaphane than Keap1a

**Fig. 5:**
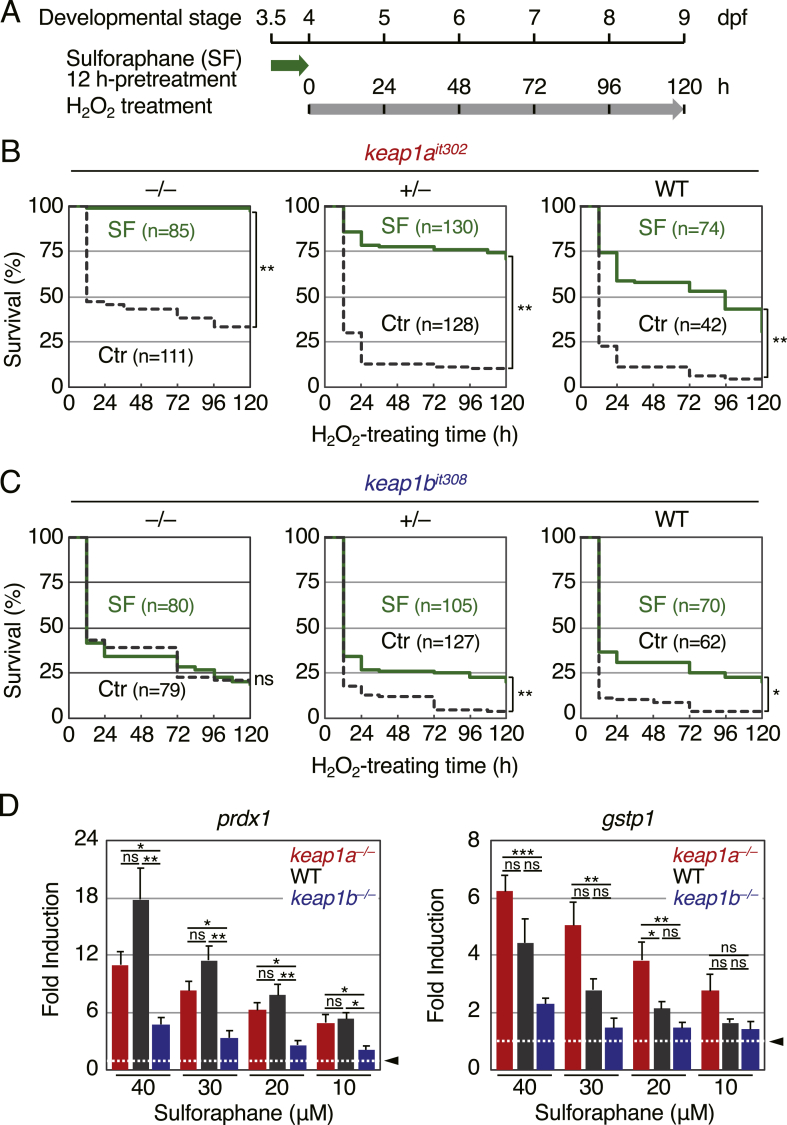
**The effect of sulforaphane pretreatment on the survival of zebrafish larvae after exposure to H**_**2**_**O**_**2**_**.** (A) A schematic illustration of the experiment. Larvae were treated with 2 mM of H_2_O_2_ at 4 dpf after 12-h pretreatment with 40 μM sulforaphane (SF). (B, C) The survival rate of *keap1a*^*it302*^ and *keap1b*^*it308*^ larvae. (D) The fold increase in the expression of Nrf2 target genes in *keap1a*- and *keap1b*-homozygous knockout and wild-type larvae with sulforaphane treatment. Larvae at 5 dpf were treated with or without sulforaphane at the indicated concentration for 6 h, and the expression of *prdx1* and *gstp1* was analyzed by qPCR. The expression of untreated larvae was normalized to 1 (arrowheads, white dotted lines). Each experiment was conducted at least three times with duplicate samples. Asterisks indicate significant differences (*p < 0.05, **p < 0.01, ***p < 0.001). ns, not significant.

In addition to Nrf2-inhibitory activity, Keap1 also plays a role as a sensor for a variety of Nrf2 activators. We previously showed that the pretreatment of sulforaphane 12 h prior to H_2_O_2_ treatment enhanced the survival rates of H_2_O_2_-treated zebrafish larvae in an Nrf2-dependent manner [7,37]. To clarify whether Keap1a and/or Keap1b are required for the antioxidant activity of sulforaphane, we performed a survival analysis using *keap1a*- and *keap1b*-knockout larvae generated from heterozygous matings (Fig. 5A). Pretreatment of sulforaphane increased the survival against H_2_O_2_ toxicity in *keap1a*-homozygous knockout larvae as well as wild-type larvae, but no such effects were noted in *keap1b*-homozygous knockout larvae (Fig. 5B and C). We previously showed that only Keap1b, not Keap1a, responded to sulforaphane in Keap1–Nrf2 co-overexpressed 8-h-post-fertilization embryos [13]. Our current and previous results suggested that Keap1b but not Keap1a is sensitive to sulforaphane.

We also analyzed the sulforaphane-induced expression of the Nrf2 target genes *prdx1* and *gstp1* by qPCR in *keap1a*- and *keap1b*-knockout larvae (Fig. 5D, Tables S11 and 12). In wild-type larvae, a 17.8- and 4.4-fold increase in *prdx1* and *gstp1* mRNA, respectively, was observed 6 h after treatment with 40 μM sulforaphane. This sulforaphane-induced expression of both genes was significantly reduced in *keap1b*-knockout larvae (*prdx1*, 4.8-fold; *gstp1,* 2.3-fold) but not in *keap1a*-knockout larvae (*prdx1*, 11.0-fold; *gstp1,* 6.2-fold), suggesting that both Keap1a and Keap1b proteins have the ability to sense sulforaphane, although the ability of Keap1a is weaker than that of Keap1b. To confirm this possibility, the dose-dependent effects of sulforaphane (10–40 μM) on the induction of these genes was analyzed. As shown in Fig. 5D (and also Fig. S2, Tables S11 and 12), 10–30 μM sulforaphane showed almost no effects on the up-regulation of *gstp1* in *keap1b*-knockout larvae (10 μM, 1.4-fold [*p* = 0.18]; 20 μM, 1.5-fold [*p* = 0.063]; 30 μM, 1.5-fold [*p* = 0.23]) but had significant gene-inducing activities in *keap1a*-knockout larvae (10 μM, 2.7-fold [*p* = 0.025]; 20 μM, 3.8-fold [*p* = 0.0041]; 30 μM, 5.0-fold [*p* = 0.0024]). Taken together, these results indicated that Keap1b is better at sensing sulforaphane than Keap1a.

## 4 Discussion

In this paper, we showed that neither *keap1a* nor *keap1b* are essential for zebrafish growth and fertility using knockout lines of these genes. With knockout of either gene in larvae, Nrf2 was activated, and the antioxidant activity was enhanced. Furthermore, we found that the antioxidant activity of sulforaphane was significantly lower in *keap1b-*knockout larvae than in wild-type larvae and *keap1a*-knockout larvae, demonstrating that Keap1b, but not Keap1a, is a physiological sensor protein for the group of electrophiles including sulforaphane.

Since all 77 fish and amphibians in the Ensembl Genome Browser (http://www.ensembl.org) have both Keap1a and Keap1b, we assumed that Keap1a must have an important role during developmental period, especially at the larval stage, but the *keap1a*-homozygous knockout larvae showed no obvious phenotypes and became normal adults in a Mendelian ratio. We therefore next examined the effects of *keap1a-*disruption on the larval gene expression by an RNA-seq analysis and detected 162 up- and 413-down regulated genes. However, the gene lineups in *keap1a*-knockout larvae were similar to those in *keap1b*-knockout larvae, and many of them, especially up-regulated genes, are included in the gene lineups in sulforaphane-treated wild-type larvae, which we consider to be Nrf2 target genes. The only category found as *keap1a*-specific was DNA replication initiation-related genes, such as minichromosome maintenance complex component 2 (*mcm2*), *mcm4* and *mcm5*. This is an interesting finding, since Keap1 was shown to interact with MCM3 protein, which is a subunit of the hexametric MCM2-7 complex required for the initiation and elongation of DNA replication in eukaryotes [38,39]. It is possible that differences in the MCM3-binding affinities between Keap1a and Keap1b may affect the gene expression of MCM complex subunits. In this sense, interaction with other Keap1-binding proteins, such as Sqstm1/p62, are also interesting. The amino acid residue corresponding to Ser602 in mouse Keap1, which seems to be important for the Keap1–Sqstm1/p62 interaction [40], was not conserved in Keap1a [2]. The binding specificity to Sqstm1/p62 protein may differ between Keap1a and Keap1b, which may lead to different responses to lysosomal stress. It will be interesting to examine the differences between *keap1a*- and *keap1b*-knockout zebrafish in the phenotypic response to a variety of environment stresses in the future. For down-regulated genes in *keap1a*-knockout larvae, 94% were overlapped with those in *keap1b*-knockout larvae but only 12% with those in sulforaphane-treated wild-type larvae. It may be due to secondary effects of the persistent up-regulation of Nrf2 in both *keap1a*- and *keap1b*-knockout larvae. To confirm this possibility, gene expression analyses of *keap1a;keap1b*-compound knockout larvae should be performed in the future.

Regarding the Nrf2-inhibitory activity, we expected to find no significant difference between Keap1a and Keap1b, since similar degradation activity of Nrf2 was previously shown in Keap1a- and Keap1b-overexpressing embryos [19]. The current findings support this notion, since the up-regulation of the Nrf2 target genes and antioxidant activities was observed in both *keap1a*- and *keap1b*-knockout larvae. In addition, we were interested to find that Nrf2 was inhibited to some extent in *keap1a*- and *keap1b*-knockout larvae under uninduced conditions and was strongly activated after sulforaphane treatment, suggesting that both Keap1a and Keap1b were able to inhibit Nrf2 without the help of their counterpart. Since fish Keap1a and Keap1b do not have cysteine residues corresponding to mouse Cys273 and Cys288, respectively [2], which are thought to be involved in Nrf2 inhibition [41], we hypothesized that Keap1a–Keap1b heterodimers formed between overexpressed and endogenous proteins, which were able to inhibit the Nrf2 activity in contrast to their unfunctional homodimers in Keap1a- and Keap1b-overexpressing embryos [19]. Indeed, we showed that Keap1a and Keap1b form heterodimers *in vitro* [19], and Wakabayashi et al. demonstrated that mouse C273A and C288A mutant Keap1s, which do not function alone, inhibited the Nrf2 activity when co-expressed in cultured cells [41]. However, our hypothesis turned out to be not correct. The results here clearly demonstrated that both Keap1a- and Keap1b-homodimers were able to inhibit the Nrf2 activity.

Cys151 has been identified as a specific sensor for sulforaphane in mammalian Keap1 [13,42,43]. Although both zebrafish Keap1a and Keap1b have cysteine residues that correspond to mammalian Cys151, the residue corresponding to Lys150 that may enhance the reactivity of Cys151 is threonine in Keap1a [13]. Indeed, we previous showed using Keap1-overexpressing embryos that zebrafish Keap1a has a lower affinity for Cys151-targeting type electrophiles, such as sulforaphane, than Keap1b [13]. The results here not only support our notion that zebrafish mainly use Keap1b to sense sulforaphane-type antioxidants but also prompts our hypothesis that the expression ratio between Keap1a and Keap1b affects the animal's reactivity to such molecules. Water environments vary in their oxygen concentration, vegetation, flow, salt concentration, water pressure, temperature and other aspects. It may be possible that animals living in water, such as fish and amphibians, have advantage over amniotes in that they can more easily adapt to changes in their environment. The identification of Nrf2 activators mediated by Keap1a that may be specific to water environments will help clarify this possibility in the future.

## Authors contributions

V.T.N., L.B. and S.O. performed the experiments; S.O. and A.K designed constructs for genome editing; J.T. and M.M. conducted data analysis; V.T.N. and M.K. designed the study and wrote the paper.

## Declaration of competing interest

The authors have no conflict of interests to declare.

## Figures and Tables

**FigS1:**
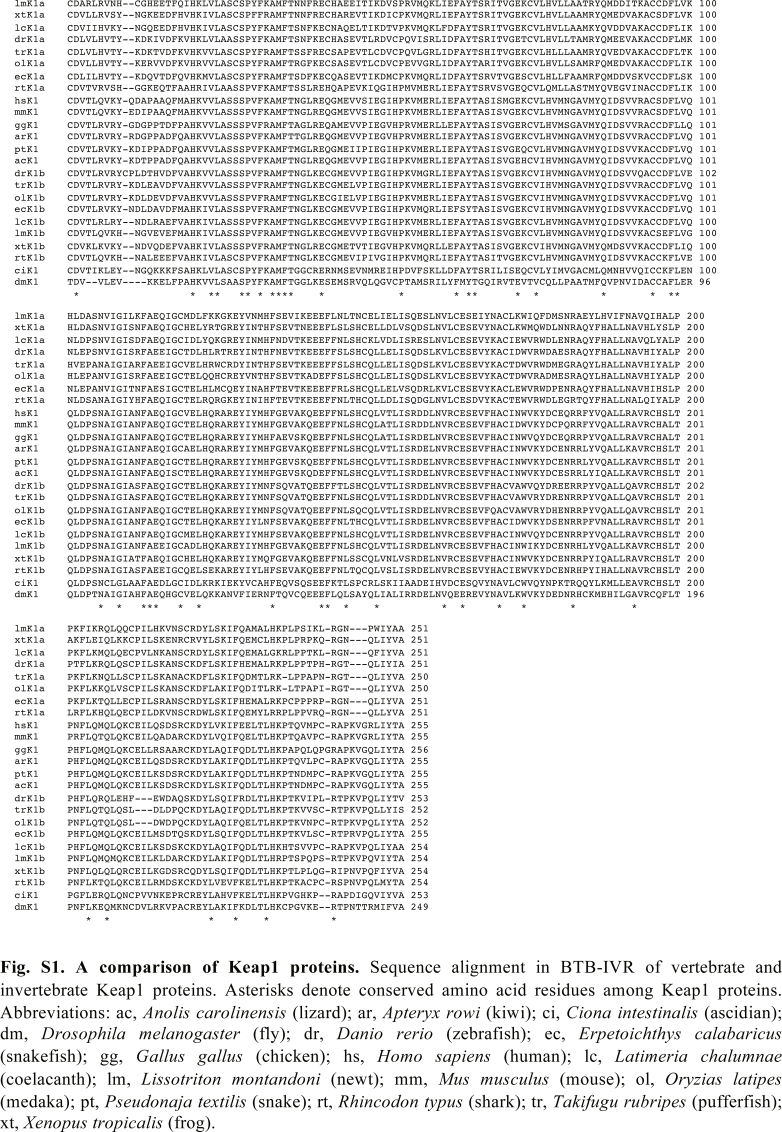


**Figs2:**