# Supplementary material for: Generation and characterization of keap1a- and keap1b-knockout zebrafish
Source: Redox Biol. 2020 Aug 11;36:101667. doi: 10.1016/j.redox.2020.101667 (PMC7452054; doi:10.1016/j.redox.2020.101667)
Supplement: Multimedia component 1 [file mmc1.docx]

Table S1. The genes up-regulated by *keap1a* disruption, *keap1b* disruption or sulforaphane treatment.

| Genes | Gene name in ZFIN | Human homologs | *keap1a* -knockout larvae vs AB larvae | *keap1b* -knockout larvae vs AB larvae | sulforaphane- treated AB larvae vs untreated AB larvae |
| --- | --- | --- | --- | --- | --- |
| BX901974.1 | – |  | 47 | 1 | 4 |
| ccbl2_2 | - | KYAT3 | 43 | 49 | 0 |
| si:ch73-329n5.6 | si:ch73-329n5.6 |  | 41.33333333 | 19 | 0.666666667 |
| si:dkeyp-73d8.6 | si:dkeyp-73d8.6 |  | 17.25 | 13 | 0.75 |
| si:dkeyp-73d8.9 | si:dkeyp-73d8.9 |  | 9.470588235 | 5.176470588 | 0.588235294 |
| zgc:194930 | zgc:194930 |  | 8.571428571 | 5.285714286 | 1.142857143 |
| saa | saa | SAA1 | 8 | 1.5 | 4 |
| zgc:163083 | zgc:163083 | DHRS2 | 7.19047619 | 3.047619048 | 9.523809524 |
| si:dkey-11k2.7 | si:dkey-11k2.7 | PTGR1 | 7.154545455 | 2.936363636 | 16.06363636 |
| scpp9 | scpp9 |  | 6.75 | 11 | 1.75 |
| si:ch1073-412h12.3 | si:ch1073-412h12.3 |  | 6.428571429 | 7.285714286 | 7.571428571 |
| SYCE3 | – | SYCE3 | 6.375 | 4.875 | 2.625 |
| BX511021.2 | – |  | 4.833333333 | 1.933333333 | 30.8 |
| per2 | per2 | PER2 | 4.638888889 | 6.75 | 0.472222222 |
| si:dkey-203a12.3 | spink2.3 | SPINK4 | 4.5 | 11 | 3 |
| mal2 | mal2 | MAL2 | 4.285714286 | 3.142857143 | 2 |
| si:ch1073-425j19.2 | si:ch1073-425j19.2 |  | 4.258064516 | 3.483870968 | 1.709677419 |
| mycbp | mycbp | MYCBP | 4.15 | 3.95 | 1.9 |
| srsf10b | srsf10b | SRSF10 | 3.933333333 | 3.6 | 1.433333333 |
| zgc:153317 | zgc:153317 | C15orf48 | 3.5 | 5.5 | 13 |
| si:dkeyp-75b4.10 | si:dkeyp-75b4.10 | ZC3H12A | 3.333333333 | 4 | 0.666666667 |
| CABZ01075125.2 | - |  | 3.25 | 0.75 | 1 |
| mfap4 | mfap4 | MFAP4 | 3.235294118 | 2 | 1.647058824 |
| gip | gip | GIP | 3.222222222 | 3.277777778 | 1.166666667 |
| zgc:174259 | zgc:174259 | SERPINA9 | 3.096153846 | 2.942307692 | 0.865384615 |
| si:dkey-14d8.19 | si:dkey-14d8.19 |  | 3 | 4 | 1 |
| ugt5a2 | ugt5a2 | UGT1A5 | 3 | 2.7 | 2.8 |
| si:ch211-84k18.3p | si:ch211-84k18.3p |  | 3 | 2.333333333 | 0.777777778 |
| si:ch211-142d6.2 | si:ch211-142d6.2 | CBLN3 | 2.964285714 | 2.857142857 | 0.892857143 |
| alas1 | alas1 | ALAS1 | 2.96039604 | 2.21980198 | 1.510891089 |
| BX255883.1 | - |  | 2.941176471 | 1.411764706 | 0 |
| SNORD99 | - | SNORD99 | 2.928571429 | 2.452380952 | 1.428571429 |
| BX908782.2 | - |  | 2.882352941 | 1.705882353 | 1.764705882 |
| cp | cp | CP | 2.847133758 | 2.101910828 | 1.203821656 |
| cyp2aa8 | cyp2aa8 | CYP2C8 | 2.803030303 | 2.96969697 | 1.060606061 |
| CCDC134 (1 of  many)ccdc3a | - | CCDC3 | 2.777777778 | 1.222222222 | 1.166666667 |
| selj | selenoj | SELENOF | 2.768292683 | 2.768292683 | 1.414634146 |
| zgc:109934 | fthl31 | FTMT | 2.648 | 2.048 | 16.032 |
| zgc:158432 | zgc:158432 |  | 2.631578947 | 1.473684211 | 1 |
| cry5 | cry5 | CRY2 | 2.553191489 | 3.744680851 | 0.765957447 |
| crp3 | crp3 | CRP | 2.539130435 | 1.808695652 | 1.339130435 |
| wdr76 | wdr76 | WDR76 | 2.529411765 | 3.352941176 | 0.588235294 |
| myoz3a | myoz3a | MYOZ3 | 2.506024096 | 0.602409639 | 0.975903614 |
| zgc:194659 | zgc:194659 |  | 2.5 | 1.7 | 2 |
| CRISP3 | – | CRISPLD2 | 2.461538462 | 1.730769231 | 3.134615385 |
| golt1ba | golt1ba | GOLT1B | 2.454545455 | 1.5 | 1.681818182 |
| serpinh1b | serpinh1b | SERPINH1 | 2.438596491 | 2.01754386 | 2.67251462 |
| 5S_rRNA_664 | - |  | 2.4 | 0.7 | 3.5 |
| si:cabz01076231.1 | si:cabz01076231.1 | CABP4 | 2.391304348 | 2.52173913 | 3.260869565 |
| si:dkey-48g21.7 | si:dkey-48g21.7 |  | 2.384615385 | 2.615384615 | 1.384615385 |
| MEP1B (1 of many) | - | MEP1B | 2.359375 | 1.84375 | 1.53125 |

| si:ch211-106h4.4 | si:ch211-106h4.4 |  | 2.352941176 | 1.117647059 | 2.588235294 |
| --- | --- | --- | --- | --- | --- |
| zgc:172053 | zgc:172053 | REG1A | 2.307692308 | 1.153846154 | 0.538461538 |
| krt93 | krt93 | KRT15 | 2.234042553 | 1.957446809 | 1.382978723 |
| c3a.2 | c3a.2 | C3 | 2.215686275 | 1.176470588 | 1.117647059 |
| si:zfos-411a11.2 | si:zfos-411a11.2 | CYP2C9 | 2.208333333 | 1.479166667 | 1.083333333 |
| TGM1 (1 of many)_2 | - | TGM2 | 2.195121951 | 1.43902439 | 1 |
| xpc | xpc | XPC | 2.157894737 | 3.192982456 | 0.736842105 |
| si:dkey-30c15.2 | si:dkey-30c15.2 |  | 2.133333333 | 2.266666667 | 2.8 |
| tg | tg | TG | 2.111111111 | 2.444444444 | 2.111111111 |
| cuzd1.1 | cuzd1.1 | CUZD1 | 2.1 | 2.6 | 1.7 |
| crygm2e | crygm2e | CRYGD | 2.083333333 | 3.083333333 | 1.75 |
| zgc:194626 | leap2 | LEAP2 | 2.04 | 1.2 | 0.92 |
| gstp1 | gstp1 | GSTP1 | 2.038800705 | 1.734567901 | 7.238977072 |
| lrit1b | lrit1b | LRIT1 | 2.035971223 | 2 | 1.374100719 |
| lonrf1l | lonrf1l | LONRF1 | 2.03030303 | 2.323232323 | 0.676767677 |
| si:ch73-23l24.1 | si:ch73-23l24.1 | OTOS | 2.021505376 | 2.462365591 | 1.010752688 |
| htatip2 | htatip2 | HTATIP2 | 2.020408163 | 1.489795918 | 2.816326531 |
| si:dkey-57c15.11 | si:dkey-57c15.11 |  | 2.005263158 | 2.378947368 | 0 |
| cyb561a3b | cyb561a3b | CYB561A3 | 2 | 2 | 26 |
| CABZ01079764.1 | - |  | 1.949152542 | 1.559322034 | 0.93220339 |
| lygl1 | lygl1 | LYG2 | 1.902777778 | 1.277777778 | 1.277777778 |
| CR735126.1 | - |  | 1.888888889 | 1.888888889 | 1.388888889 |
| cmbl | cmbl | CMBL | 1.880952381 | 1.773809524 | 6.107142857 |
| prss60.2 | prss60.2 | PRSS48 | 1.880952381 | 1.380952381 | 1.738095238 |
| taldo1 | taldo1 | TALDO1 | 1.876056338 | 1.6 | 2.036619718 |
| ccdc125 | ccdc125 | CCDC125 | 1.875 | 2.375 | 1.333333333 |
| cpox | cpox | CPOX | 1.872340426 | 1.319148936 | 1.170212766 |
| selenbp1 | selenbp1 | SELENBP1 | 1.87012987 | 1.61038961 | 1.733766234 |
| ces3 | ces3 | CES1 | 1.868217054 | 2.290697674 | 1.166666667 |
| gatm | gatm | GATM | 1.856780735 | 1.651457541 | 0.93113646 |
| si:dkey-22i16.7 | si:dkey-22i16.7 |  | 1.837837838 | 1.810810811 | 1.310810811 |
| prdx1 | prdx1 | PRDX1 | 1.832402235 | 1.988826816 | 8.782122905 |
| aifm4 | aifm4 | AIFM1 | 1.830188679 | 1.226415094 | 7.566037736 |
| si:ch211-161h7.5 | si:ch211-161h7.5 |  | 1.830065359 | 3.137254902 | 0.594771242 |
| mcm2 | mcm2 | MCM2 | 1.815384615 | 1.396153846 | 0.696153846 |
| TGM1 (1 of many)_1 | - | TGM2 | 1.808510638 | 1.340425532 | 1.14893617 |
| si:ch211-158m24.12 | si:ch211-158m24.12 |  | 1.804761905 | 2.104761905 | 0.457142857 |
| TSTD1 | – | TSTA3 | 1.803571429 | 1.767857143 | 6.071428571 |
| gstp2 | gstp2 | GSTP1 | 1.793220339 | 1.772881356 | 5.183050847 |
| ugdh | ugdh | UGDH | 1.792307692 | 1.038461538 | 1.730769231 |
| si:dkey-7c18.24 | si:dkey-7c18.24 |  | 1.787162162 | 1.057432432 | 1.881756757 |
| pcyt1bb | pcyt1bb | PCYT1B | 1.786324786 | 1.905982906 | 1.299145299 |
| si:dkey-4e7.3 | si:dkey-4e7.3 |  | 1.784810127 | 1.037974684 | 1.025316456 |
| abhd4 | abhd4 | ABHD4 | 1.784090909 | 2.386363636 | 2.772727273 |
| proca | proca | PROC | 1.783783784 | 1.108108108 | 1.432432432 |
| si:dkey-96g2.1 | si:dkey-96g2.1 |  | 1.777777778 | 1 | 0.805555556 |
| si:ch211-252f13.6 | si:ch211-252f13.6 |  | 1.774193548 | 1.161290323 | 1.709677419 |
| mpped2a | mpped2a | MPPED2 | 1.767857143 | 1.089285714 | 1.517857143 |
| ethe1 | ethe1 | ETHE1 | 1.759856631 | 1.519713262 | 2.569892473 |
| slc22a6l | slc22a6l | SLC22A8 | 1.75 | 1.208333333 | 2.083333333 |
| DNM2 (1 of many) | - | DNM2 | 1.744680851 | 1.446808511 | 1.319148936 |
| LRAT | - | LRAT | 1.725490196 | 2.274509804 | 1.37254902 |
| cyp2k18 | cyp2k18 | CYP2C18 | 1.724137931 | 1.379310345 | 0.862068966 |
| ppp1r27b | ppp1r27b | PPP1R27 | 1.714285714 | 2 | 1.285714286 |
| trdn | trdn | TRDN | 1.701492537 | 1.910447761 | 1.243781095 |
| gsto2 | gsto2 | GSTO1 | 1.671559633 | 1.469724771 | 4.181651376 |
| chaf1b | chaf1b | CHAF1B | 1.666666667 | 0.909090909 | 0.787878788 |

| slc13a1 | slc13a1 | SLC13A1 | 1.666666667 | 1.333333333 | 0.619047619 |
| --- | --- | --- | --- | --- | --- |
| tmprss13a | tmprss13a | TMPRSS13 | 1.659090909 | 1.204545455 | 1.909090909 |
| keap1b | keap1b | KEAP1 | 1.650793651 | 0.507936508 | 4.19047619 |
| mcm5 | mcm5 | MCM5 | 1.647342995 | 1.280193237 | 0.628019324 |
| grk7a | grk7a | GRK7 | 1.646766169 | 1.664179104 | 1.154228856 |
| pan3 | pan3 | PAN3 | 1.636363636 | 0.939393939 | 1.454545455 |
| eevs | eevs |  | 1.629482072 | 2.605577689 | 0.888446215 |
| si:cabz01117780.1 | si:cabz01117780.1 |  | 1.62745098 | 1.529411765 | 1.960784314 |
| mcm4 | mcm4 | MCM4 | 1.62254902 | 1.210784314 | 0.715686275 |
| whsc1 | whsc1 | NSD2 | 1.621621622 | 1 | 0.864864865 |
| apoa4a | apoa4a | APOA4 | 1.606666667 | 1.186666667 | 0.593333333 |
| tgm2a | tgm2a | TGM2 | 1.605769231 | 1.692307692 | 1.192307692 |
| SI | - | SI | 1.60472973 | 0.824324324 | 1.091216216 |
| si:rp71-1c10.11 | si:rp71-1c10.11 |  | 1.598958333 | 2.317708333 | 1.677083333 |
| cbr1l | cbr1l | CBR3 | 1.594458438 | 1.576826196 | 3.20906801 |
| pmelb | pmelb | PMEL | 1.586206897 | 1.137931034 | 2.068965517 |
| abl1 | abl1 | ABL1 | 1.586206897 | 0.655172414 | 0.793103448 |
| fam162a | fam162a | FAM162A | 1.585774059 | 1.435146444 | 1.59832636 |
| slc26a3.2 | slc26a3.2 | SLC26A3 | 1.585365854 | 1.089430894 | 1.788617886 |
| si:ch211-224m9.4 | si:ch211-224m9.4 |  | 1.578947368 | 1.052631579 | 1.157894737 |
| BX322612.1 | - |  | 1.571428571 | 1.523809524 | 0.952380952 |
| gsr | gsr | GSR | 1.567164179 | 1.313432836 | 6.194029851 |
| cbln8 | cbln8 | CBLN3 | 1.566666667 | 5.033333333 | 1.633333333 |
| rpe65a | rpe65a | RPE65 | 1.564444444 | 1.647407407 | 1.259259259 |
| dhdhl | dhdhl | DHDH | 1.559808612 | 1.64354067 | 1.523923445 |
| si:dkey-238c7.12 | si:dkey-238c7.12 |  | 1.557760814 | 1.697201018 | 1.486005089 |
| cart3 | cart3 | CARTPT | 1.55 | 1.4 | 1 |
| khsrp | khsrp | KHSRP | 1.549222798 | 0.994818653 | 1.14507772 |
| OLFM4 (1 of many)_1 | - | OLFM3 | 1.548387097 | 1.016129032 | 0.85483871 |
| sod3a | sod3a | SOD3 | 1.545454545 | 1.5 | 2.227272727 |
| CABZ01069194.1 | - |  | 1.541666667 | 2 | 0.958333333 |
| mansc1 | mansc1 | MANSC4 | 1.540540541 | 0.983783784 | 1.124324324 |
| gclc | gclc | GCLC | 1.536082474 | 1.216494845 | 2.577319588 |
| mgst1.2 | mgst1.2 | MGST1 | 1.532846715 | 1.496350365 | 2.729927007 |
| zgc:172051 | zgc:172051 |  | 1.53164557 | 0.924050633 | 1.037974684 |
| rerg | rerg | RERG | 1.53125 | 1.1875 | 1.03125 |
| txnrd1 | txnrd1 | TXNRD3 | 1.526162791 | 0.944767442 | 1.819767442 |
| pgd | pgd | PGD | 1.524916944 | 1.401993355 | 3.548172757 |
| dnmt1 | dnmt1 | DNMT1 | 1.523809524 | 1.19047619 | 0.833333333 |
| fth1a | fth1a | FTH1 | 1.522402597 | 1.733928571 | 1.455681818 |
| si:ch211-237l4.6 | si:ch211-237l4.6 |  | 1.52173913 | 1.5 | 1.304347826 |
| si:ch211-284e13.6 | si:ch211-284e13.6 |  | 1.52173913 | 1.347826087 | 1.47826087 |
| zanl | zanl | ZAN | 1.519083969 | 0.916030534 | 1.083969466 |
| ssbp3b | ssbp3b | SSBP3 | 1.504347826 | 0.991304348 | 1.452173913 |
| irbp | rbp3 | RBP3 | 1.501216545 | 1.309002433 | 1.080291971 |
| si:ch211-203h15.7 | si:ch211-203h15.7 |  | 1.5 | 3.166666667 | 3.333333333 |
| plekhf1 | plekhf1 | PLEKHF1 | 1.5 | 1.246153846 | 1.415384615 |
| lamb2 | lamb2 | LAMB2 | 1.5 | 1.093023256 | 1.058139535 |
| foxd2 | foxd2 | FOXD1 | 1.5 | 0.975 | 1.825 |
| zswim8 | zswim8 | ZSWIM8 | 1.5 | 0.9375 | 1.645833333 |
| aqp11 | aqp11 | AQP11 | 1.5 | 0.620689655 | 0.810344828 |
| NPC2 (1 of many) | - | NPC2 | 1.5 | 0.611111111 | 0.555555556 |
| si:ch211-14a17.10 | si:ch211-14a17.10 |  | 1.5 | 3.25 | 5.75 |
| BX323596.2 | - |  | 1.5 | 1.5 | 1.5 |
| CU633479.2 | - |  | 1.489583333 | 1.697916667 | 1.208333333 |
| proser1 |  |  | 1.486486486 | 0.837837838 | 1.27027027 |
| si:ch211-246m6.4 | si:ch211-246m6.4 | SCX | 1.484848485 | 1.424242424 | 1.545454545 |

| si:ch211-103f14.3 | si:ch211-103f14.3 |  | 1.484848485 | 1.151515152 | 2.121212121 |
| --- | --- | --- | --- | --- | --- |
| acox1 |  |  | 1.483333333 | 0.983333333 | 1.116666667 |
| fxr2 |  |  | 1.481481481 | 1.234567901 | 1.012345679 |
| slc7a9 | slc7a9 | SLC7A9 | 1.481481481 | 1.037037037 | 1.925925926 |
| zgc:165423 |  |  | 1.478589421 | 1.204030227 | 0.949622166 |
| usp19 |  |  | 1.47826087 | 0.869565217 | 1.391304348 |
| si:dkey-251i10.2 | si:dkey-251i10.2 |  | 1.473988439 | 1.49132948 | 3.728323699 |
| PPM1K (1 of many) | - | PPM1J | 1.472727273 | 1.636363636 | 0.709090909 |
| atp1a2a |  |  | 1.469879518 | 1.096385542 | 1.162650602 |
| serpinb1l4 | serpinb1l4 | SERPINB6 | 1.468085106 | 1.234042553 | 1.595744681 |
| mkrn2_1 |  |  | 1.467741935 | 1.209677419 | 1.225806452 |
| cry4 |  |  | 1.466666667 | 1.466666667 | 1.3 |
| AIFM2 | aifm2 | AIFM2 | 1.464968153 | 1.038216561 | 3.121019108 |
| smg1 |  |  | 1.463414634 | 0.902439024 | 1.390243902 |
| cyp2aa1 | cyp2aa1 | CYP2C8 | 1.46 | 1.06 | 1.62 |
| mep1a.2 |  |  | 1.457627119 | 1.016949153 | 1.084745763 |
| zgc:198419 | fthl28 | FTMT | 1.457142857 | 1.523809524 | 14.17142857 |
| acsf2 |  |  | 1.456 | 0.968 | 0.88 |
| si:ch211-278f21.5 |  |  | 1.454545455 | 0.696969697 | 1.181818182 |
| mcm3 |  | MCM3 | 1.453333333 | 1.08 | 0.66 |
| prps1a |  |  | 1.451923077 | 1.067307692 | 1.158653846 |
| capn3b |  |  | 1.450980392 | 1.392156863 | 0.882352941 |
| hells |  | HELLS | 1.447761194 | 1.268656716 | 0.582089552 |
| mat2aa |  |  | 1.44691358 | 1.333333333 | 1.091358025 |
| pglyrp6 | pglyrp6 | PGLYRP2 | 1.444444444 | 3 | 2.333333333 |
| CABZ01078858.1 |  |  | 1.443298969 | 0.81443299 | 1.195876289 |
| cyp2p6 | cyp2p6 | CYP2J2 | 1.442307692 | 0.634615385 | 0.846153846 |
| ace |  |  | 1.439189189 | 1.084459459 | 1.472972973 |
| abcc2 | abcc2 | ABCC2 | 1.4375 | 1.0625 | 2.9025 |
| zgc:173593 | fthl30 | FTMT | 1.434782609 | 1.043478261 | 3.652173913 |
| zgc:101553 | acot19 | ACOT7 | 1.428571429 | 1.642857143 | 0.642857143 |
| si:ch73-31d8.2 | si:ch73-31d8.2 |  | 1.428571429 | 1.571428571 | 2.071428571 |
| acp5b | acp5b | ACP5 | 1.421052632 | 1.789473684 | 2.421052632 |
| si:dkey-95j14.1 |  |  | 1.414634146 | 0.926829268 | 1.341463415 |
| hoxb13a |  |  | 1.411764706 | 0.970588235 | 0.970588235 |
| zgc:153115 | zgc:153115 | KLF16 | 1.411764706 | 0.794117647 | 1.588235294 |
| egfl6_1 |  |  | 1.410658307 | 1.269592476 | 1.094043887 |
| rbm12 |  |  | 1.41025641 | 0.948717949 | 0.820512821 |
| si:ch211-114n24.7 |  |  | 1.409090909 | 1.022727273 | 1.113636364 |
| mn1a |  |  | 1.408163265 | 1.040816327 | 1.346938776 |
| ankrd46a |  | ANKRD46 | 1.404761905 | 0.642857143 | 1.214285714 |
| si:ch73-236j9.2 | si:ch73-236j9.2 | SLC35E2B | 1.4 | 0.7 | 1.5 |
| si:ch211-244c8.4 |  |  | 1.397058824 | 0.926470588 | 1.294117647 |
| dhrs13l1 | dhrs13l1 | DHRS13 | 1.39673913 | 1.342391304 | 2.358695652 |
| dlgap4a |  |  | 1.396551724 | 0.913793103 | 1.051724138 |
| si:ch1073-174d20.2 |  |  | 1.395348837 | 1.162790698 | 1.348837209 |
| spsb4a |  |  | 1.395348837 | 1.069767442 | 1.418604651 |
| si:ch211-194m7.3 |  |  | 1.392857143 | 0.892857143 | 0.928571429 |
| ivns1abpa |  |  | 1.391791045 | 1.052238806 | 1.205223881 |
| si:ch211-253h3.1 | abr | ABR | 1.388888889 | 0.888888889 | 1.5 |
| rb1 |  |  | 1.388888889 | 0.861111111 | 0.833333333 |
| tbc1d12a |  |  | 1.387755102 | 0.897959184 | 1.428571429 |
| napgb | napgb | NAPG | 1.385620915 | 1.287581699 | 1.54248366 |
| ubr5 |  |  | 1.385542169 | 0.939759036 | 1.240963855 |
| myh9b |  |  | 1.385026738 | 0.903743316 | 0.957219251 |
| dclk1a |  |  | 1.384615385 | 0.897435897 | 0.948717949 |
| cyyr1 |  |  | 1.38 | 0.76 | 1.12 |

| txnbb |  | TNXB | 1.378378378 | 1.378378378 | 0.567567568 |
| --- | --- | --- | --- | --- | --- |
| iqgap1 |  |  | 1.377358491 | 0.849056604 | 0.924528302 |
| slc5a1 |  |  | 1.376470588 | 0.976470588 | 1.058823529 |
| sik2b |  |  | 1.375 | 1.178571429 | 1.125 |
| CELA1 (1 of many)_6 | - | CELA1 | 1.375 | 2.28125 | 1.322916667 |
| mt2 | mt2 | MT2A | 1.373563218 | 1.377011494 | 4.997701149 |
| gsto1 | gsto1 | GSTO1 | 1.373134328 | 1.28358209 | 2.164179104 |
| sf1 |  |  | 1.373076923 | 0.865384615 | 1.346153846 |
| TNC (1 of many) |  |  | 1.371428571 | 0.942857143 | 0.942857143 |
| hnrnpaba |  | HNRNPAB | 1.371349096 | 0.948539638 | 1.136300417 |
| b4galt1 | b4galt1 | B4GALT1 | 1.37037037 | 0.851851852 | 1.851851852 |
| pbx4 |  |  | 1.36746988 | 0.843373494 | 1.265060241 |
| mcm6 |  | MCM6 | 1.367346939 | 0.850340136 | 0.632653061 |
| mta2 |  |  | 1.366666667 | 0.9 | 1.216666667 |
| nid2a |  |  | 1.366197183 | 0.929577465 | 1 |
| fbxw7 |  |  | 1.365384615 | 0.846153846 | 1.269230769 |
| pygo2 |  |  | 1.365079365 | 0.936507937 | 1.285714286 |
| nptx1l |  |  | 1.363636364 | 0.927272727 | 1.290909091 |
| si:ch211-153b23.5 | si:ch211-153b23.5 |  | 1.362903226 | 0.887096774 | 2.717741935 |
| rras |  |  | 1.362068966 | 0.74137931 | 1.448275862 |
| glg1a |  |  | 1.361904762 | 0.838095238 | 1.20952381 |
| slc35b4 | slc35b4 | SLC35B4 | 1.358974359 | 0.602564103 | 1.769230769 |
| tspo | tspo | TSPO | 1.355555556 | 2.115555556 | 1.32 |
| hpgd |  |  | 1.355555556 | 1.2 | 0.755555556 |
| chd8 |  |  | 1.354166667 | 0.854166667 | 1.375 |
| gba2 |  |  | 1.353846154 | 0.892307692 | 1.492307692 |
| ubtd1a |  |  | 1.352941176 | 1.117647059 | 1.367647059 |
| abcb4 |  | ABCB4 | 1.351020408 | 0.951020408 | 0.955102041 |
| sardh |  |  | 1.35 | 1.083333333 | 1.066666667 |
| cyp2ad6 | cyp2ad6 | CYP2J2 | 1.348314607 | 0.674157303 | 0.640449438 |
| col2a1b |  |  | 1.344594595 | 1.048648649 | 0.924324324 |
| hk2 |  |  | 1.342857143 | 0.942857143 | 0.971428571 |
| impg1a |  |  | 1.341463415 | 0.756097561 | 1.268292683 |
| zgc:77058 |  |  | 1.34 | 0.86 | 1.06 |
| hspa4a | hspa4a | HSPA4 | 1.339534884 | 0.865116279 | 1.865116279 |
| aplp2 |  |  | 1.338461538 | 0.835897436 | 1.471794872 |
| col14a1b | col14a1b | COL14A1 | 1.336538462 | 1.086538462 | 1.134615385 |
| fbxl14b |  |  | 1.333333333 | 1.075757576 | 1.333333333 |
| btbd6a |  |  | 1.333333333 | 0.974358974 | 1.320512821 |
| hkdc1 |  |  | 1.333333333 | 0.717948718 | 1.102564103 |
| pon1 | pon1 | PON2 | 1.333333333 | 1.666666667 | 3.555555556 |
| slc37a4b_2 |  |  | 1.333333333 | 0.955555556 | 1.133333333 |
| slc6a19a.2 | slc6a19a.2 | SLC6A19 | 1.331189711 | 0.829581994 | 1.742765273 |
| histh1l |  |  | 1.330252101 | 0.883193277 | 1.159663866 |
| txn | txn | TXN | 1.329341317 | 1.281437126 | 7.479041916 |
| col9a3 |  |  | 1.328947368 | 1.019138756 | 1.017942584 |
| gpx1a | gpx1a | GPX1 | 1.327777778 | 1.294444444 | 2.15 |
| epha4a |  |  | 1.327272727 | 0.927272727 | 1.2 |
| dlat |  |  | 1.326446281 | 1.024793388 | 1.214876033 |
| mgst3b | mgst3b | MGST3 | 1.325404377 | 0.988582303 | 2.877259753 |
| col2a1a |  |  | 1.323492723 | 0.943035343 | 1.036590437 |
| ptgdsb.1 | ptgdsb.1 | PTGDS | 1.320077973 | 2.249902534 | 0.885769981 |
| arhgef9b |  |  | 1.318181818 | 0.886363636 | 1.272727273 |
| apoba |  | APOB | 1.311688312 | 0.948051948 | 0.597402597 |
| bco1l | bco1l | BCO1 | 1.307692308 | 1.461538462 | 1.641025641 |
| CHCHD5 |  |  | 1.307692308 | 1.41025641 | 1.051282051 |
| mdh1ab | mdh1ab | MDH1 | 1.305555556 | 3.472222222 | 1.388888889 |

| gabrr1 |  |  | 1.304347826 | 1.413043478 | 1.304347826 |
| --- | --- | --- | --- | --- | --- |
| crkl |  |  | 1.302752294 | 0.981651376 | 1.394495413 |
| CABZ01041812.1 | – |  | 1.302325581 | 0.930232558 | 6.162790698 |
| unc45b |  |  | 1.301369863 | 1.02739726 | 1.219178082 |
| sema5a |  |  | 1.3 | 0.775 | 1.3 |
| rbm4.3 |  |  | 1.29969419 | 0.819571865 | 1.183486239 |
| gsta.2 | gsta.2 | GSTA | 1.299595142 | 1.109311741 | 3.740890688 |
| rbm4.2 |  |  | 1.299065421 | 0.803738318 | 1.14953271 |
| ctnnb2 |  |  | 1.298969072 | 0.945017182 | 1.182130584 |
| gpx4a | gpx4a | GPX5 | 1.297450425 | 1.780453258 | 1.144475921 |
| gata2a |  |  | 1.295454545 | 1.022727273 | 1.181818182 |
| fam208ab |  |  | 1.295454545 | 0.840909091 | 1.272727273 |
| crygm2b | crygm2b | CRYGB | 1.294117647 | 1.941176471 | 1.235294118 |
| zgc:92066 | fthl27 | FTMT | 1.294117647 | 1.483065954 | 4.130124777 |
| canx |  |  | 1.29216152 | 0.7695962 | 1.190023753 |
| CABZ01077402.1 |  |  | 1.288888889 | 0.888888889 | 1.177777778 |
| znf106a |  |  | 1.2875 | 0.775 | 1.3125 |
| magi1b |  |  | 1.2875 | 0.775 | 1.2 |
| tefa |  |  | 1.286833856 | 1.4169279 | 0.993730408 |
| tmem51a | tmem51a | TEM51 | 1.285714286 | 0.761904762 | 1.952380952 |
| zgc:56585 |  |  | 1.285714286 | 0.738095238 | 1.452380952 |
| dyrk1b |  |  | 1.284810127 | 0.860759494 | 1.253164557 |
| smyd2b |  |  | 1.284313725 | 1.058823529 | 1.107843137 |
| nup153 |  | NUP153 | 1.283018868 | 0.660377358 | 1.113207547 |
| ilf3b |  |  | 1.282894737 | 0.861842105 | 1.148026316 |
| nr2e3 | nr2e3 | NR2E3 | 1.282051282 | 0.846153846 | 1.820512821 |
| col9a2 |  |  | 1.281907433 | 1.035063114 | 1.053295933 |
| hnrnpa0b |  | HNRNPA3 | 1.281860465 | 0.861395349 | 1.044651163 |
| dtnba |  |  | 1.28125 | 1.10625 | 1.09375 |
| soul5 | soul5 | HEBP1 | 1.279389313 | 2.546564885 | 0.650381679 |
| dio1 |  |  | 1.278481013 | 1.215189873 | 0.860759494 |
| ctdspl2a |  |  | 1.278481013 | 0.873417722 | 1.139240506 |
| MFAP4 (1 of many)_9 | - | MFAP5 | 1.277777778 | 2.388888889 | 1.555555556 |
| pdzd3b |  |  | 1.277777778 | 0.855555556 | 0.933333333 |
| aco2 |  |  | 1.276264591 | 0.837872892 | 1.167315175 |
| hoxd9a | hoxd9a | HOXD9 | 1.275 | 0.8 | 1.625 |
| ipo4 |  |  | 1.274509804 | 0.68627451 | 1.019607843 |
| lrrc58a | lrrc58a | LRRC58 | 1.272727273 | 1.045454545 | 1.590909091 |
| morc2 |  |  | 1.27 | 0.82 | 1.06 |
| atp1a3b |  |  | 1.269662921 | 0.820224719 | 1.161048689 |
| scarb1 | scarb1 | SCARB1 | 1.268292683 | 1.634146341 | 4.536585366 |
| shc1 |  |  | 1.267857143 | 0.696428571 | 1.160714286 |
| sgca |  |  | 1.26744186 | 1.023255814 | 1.151162791 |
| ppm1bb |  | PPM1B | 1.266666667 | 0.881481481 | 1.496296296 |
| stt3b |  |  | 1.264516129 | 0.741935484 | 1.219354839 |
| parp1 |  |  | 1.264 | 0.984 | 0.76 |
| gpr39 |  |  | 1.262975779 | 0.885813149 | 1.415224913 |
| slc1a4 | slc1a4 | SLC1A4 | 1.260869565 | 0.695652174 | 1.52173913 |
| ifi30 |  |  | 1.260416667 | 1.182291667 | 1.338541667 |
| atp2a2b |  | ATP2A2 | 1.259259259 | 0.580246914 | 1.358024691 |
| nfe2l1b | nfe2l1b | NFE2L1 | 1.259259259 | 1.037037037 | 2.481481481 |
| RBP1 |  |  | 1.259259259 | 1 | 0.703703704 |
| HNRNPUL1 (1 of many) | hnrnpua | HNRNPU | 1.255813953 | 0.813953488 | 1.063122924 |
| abca1b |  | ABCA1 | 1.255102041 | 0.795918367 | 0.87755102 |
| selo | selenoo1 | SELENOO | 1.254901961 | 0.960784314 | 1.647058824 |
| CABZ01092282.1 |  |  | 1.254098361 | 0.663934426 | 1.106557377 |
| ftr83 | ftr83 | RFPL3 | 1.253521127 | 0.605633803 | 1.070422535 |

| tnc |  |  | 1.251612903 | 0.929032258 | 1.006451613 |
| --- | --- | --- | --- | --- | --- |
| si:dkey-266f7.9 | si:dkey-266f7.9 |  | 1.25 | 1.770833333 | 1.666666667 |
| prpf4bb |  |  | 1.25 | 0.985294118 | 1.161764706 |
| pdzk1 |  |  | 1.25 | 0.952513966 | 0.997206704 |
| zgc:123284 | zgc:123284 | DHRS4 | 1.25 | 0.95 | 9.55 |
| serpinh1a |  |  | 1.25 | 0.75 | 1.25 |
| sbf1 |  |  | 1.25 | 0.708333333 | 1.270833333 |
| cltca |  |  | 1.246753247 | 0.727272727 | 1.12987013 |
| MINOS1-NBL1 |  |  | 1.246153846 | 0.815384615 | 1.076923077 |
| hsp90aa1.1 | hsp90aa1.1 | HSP90AA1 | 1.245934959 | 1.201219512 | 1.607723577 |
| insra |  |  | 1.245901639 | 0.68852459 | 1.262295082 |
| lmod3 |  |  | 1.245614035 | 0.98245614 | 1.210526316 |
| zbtb14 |  |  | 1.245283019 | 0.924528302 | 1.301886792 |
| fam110b |  |  | 1.244897959 | 0.816326531 | 1.040816327 |
| med14 |  |  | 1.243243243 | 0.810810811 | 1.324324324 |
| flnbl | flnbl | FLNC | 1.241758242 | 0.604395604 | 1.065934066 |
| igfn1.1 |  |  | 1.24137931 | 0.965517241 | 1.181034483 |
| dyrk1aa |  |  | 1.24137931 | 0.931034483 | 1.362068966 |
| rsrp1 | rsrp1 | RSRP1 | 1.240837696 | 1 | 2.863874346 |
| ankfy1 |  |  | 1.24 | 0.88 | 1.1 |
| dennd4b |  |  | 1.238095238 | 0.880952381 | 1.285714286 |
| syn2b |  |  | 1.236842105 | 0.710526316 | 0.881578947 |
| loxl2b |  |  | 1.23655914 | 1.010752688 | 0.903225806 |
| slc38a2 | slc38a2 | SLC38A2 | 1.236111111 | 0.777777778 | 1.680555556 |
| arhgap12b |  |  | 1.235294118 | 0.882352941 | 0.980392157 |
| ssb |  |  | 1.235294118 | 0.911764706 | 1.137254902 |
| zgc:92630 | zgc:92630 | DHRS11 | 1.233766234 | 1.136363636 | 3.545454545 |
| srpr |  |  | 1.231884058 | 0.833333333 | 1.050724638 |
| tktb | tktb | TKTL2 | 1.231775701 | 1.042990654 | 1.805607477 |
| atp1b2b |  |  | 1.230769231 | 0.945054945 | 1.494505495 |
| myh10 |  |  | 1.22962963 | 0.874074074 | 1.088888889 |
| si:ch211-266o15.1 |  |  | 1.229166667 | 0.958333333 | 1.270833333 |
| hagh | hagh | HAGH | 1.228855721 | 0.771144279 | 1.870646766 |
| adob_2 | adob | ADO | 1.228571429 | 0.771428571 | 2.371428571 |
| abcc12 | abcc12 | ABCC12 | 1.227272727 | 0.977272727 | 2.5 |
| samd11 |  |  | 1.227272727 | 0.840909091 | 1.136363636 |
| inaa |  |  | 1.225641026 | 1.082051282 | 1.153846154 |
| si:ch1073-464p5.5 | si:ch1073-464p5.5 |  | 1.223300971 | 0.359223301 | 1.077669903 |
| ylpm1 |  |  | 1.222222222 | 0.711111111 | 0.955555556 |
| zgc:158296 |  |  | 1.222222222 | 1.203703704 | 1.037037037 |
| llgl2 |  |  | 1.222222222 | 0.740740741 | 1.296296296 |
| vil1 |  |  | 1.221954162 | 0.66827503 | 1.41013269 |
| btbd10b |  |  | 1.220930233 | 0.738372093 | 1.412790698 |
| tes |  |  | 1.218181818 | 0.909090909 | 1.054545455 |
| mprip |  |  | 1.216981132 | 0.971698113 | 1.254716981 |
| trib2 | trib2 | TRIB2 | 1.216666667 | 0.9 | 1.533333333 |
| si:ch211-255f14.2 | si:ch211-255f14.2 |  | 1.216216216 | 0.648648649 | 1.405405405 |
| si:dkey-100n23.4 | si:dkey-100n23.4 |  | 1.215053763 | 1.848267622 | 1.620071685 |
| CABZ01020840.1 | – |  | 1.214285714 | 0.714285714 | 2.285714286 |
| dnajb1b | dnajb1b |  | 1.214285714 | 0.857142857 | 2.657142857 |
| slc15a1a |  |  | 1.21299639 | 1.003610108 | 1.21299639 |
| hgd | hgd | HGD | 1.212938005 | 1.711590296 | 1.889487871 |
| myhz1.3 | myhz1.3 | MYH4 | 1.212885154 | 1.572362278 | 1.203548086 |
| astn1 |  |  | 1.212765957 | 0.680851064 | 1.29787234 |
| sqrdl | sqor | SQOR | 1.212121212 | 2.131313131 | 4.909090909 |
| slc16a3 | slc16a3 | SLC16A3 | 1.211538462 | 0.788461538 | 1.711538462 |
| si:ch211-240l19.8 | si:ch211-240l19.8 |  | 1.210526316 | 1.473684211 | 2.421052632 |

| ciapin1 | ciapin1 | CIAPIN1 | 1.209302326 | 1.15503876 | 2.666666667 |
| --- | --- | --- | --- | --- | --- |
| kdm5ba |  |  | 1.209090909 | 0.890909091 | 1.327272727 |
| zgc:65895 |  |  | 1.208791209 | 0.934065934 | 1.10989011 |
| fam184b |  |  | 1.208695652 | 1.347826087 | 1.373913043 |
| si:dkey-119m7.4 | si:dkey-119m7.4 | SLC22A14 | 1.208333333 | 1.416666667 | 1.666666667 |
| hsd17b12a |  |  | 1.207692308 | 0.938461538 | 1.453846154 |
| ncoa6 |  |  | 1.20754717 | 0.698113208 | 1.226415094 |
| lama5 |  |  | 1.203703704 | 0.796296296 | 1.055555556 |
| me1 | me1 | ME1 | 1.203125 | 1.109375 | 1.90625 |
| myom2b |  |  | 1.202830189 | 0.995283019 | 1.16509434 |
| dusp7 |  |  | 1.202380952 | 0.821428571 | 1.285714286 |
| zgc:154093 |  |  | 1.2 | 1.044444444 | 1.066666667 |
| jam2b |  |  | 1.2 | 0.777777778 | 1.066666667 |
| akap7 |  |  | 1.2 | 1.25 | 1.2 |
| xpo1a | xpo1a | XPO1 | 1.2 | 0.893333333 | 1.933333333 |
| ezra |  |  | 1.198019802 | 0.702970297 | 0.712871287 |
| scaf1 |  |  | 1.197802198 | 0.879120879 | 1.153846154 |
| zgc:153896 |  |  | 1.197368421 | 0.894736842 | 1.276315789 |
| CABZ01059415.2 | - |  | 1.196969697 | 1.818181818 | 0.924242424 |
| sec24d |  |  | 1.196721311 | 0.803278689 | 1.229508197 |
| sin3aa |  |  | 1.196428571 | 0.714285714 | 1.357142857 |
| sgpl1 |  |  | 1.196319018 | 0.791411043 | 0.950920245 |
| ppip5k1b |  | PPIP5K1 | 1.196078431 | 0.666666667 | 0.921568627 |
| zbtb8b |  |  | 1.195121951 | 1.12195122 | 1.463414634 |
| agrn |  |  | 1.194029851 | 0.746268657 | 1.223880597 |
| arf2a |  |  | 1.193925234 | 0.778037383 | 1.310747664 |
| cdkn1ca |  |  | 1.191780822 | 0.863013699 | 1.150684932 |
| mfn1b_2 |  |  | 1.191780822 | 0.808219178 | 1.205479452 |
| si:ch73-46j18.5 |  |  | 1.191616766 | 0.784431138 | 1.251497006 |
| phox2bb |  |  | 1.191489362 | 0.70212766 | 0.914893617 |
| cap2 |  |  | 1.189473684 | 1 | 1.031578947 |
| pbxip1a |  |  | 1.189189189 | 0.882882883 | 1.288288288 |
| tardbp |  |  | 1.189102564 | 0.807692308 | 1.041666667 |
| myhz2 |  |  | 1.188419405 | 1.342514345 | 1.140636411 |
| hpdb | hpdb | HPD | 1.1872 | 1.8496 | 2.5712 |
| rcc2 |  |  | 1.186813187 | 0.758241758 | 1.115384615 |
| si:ch211-117m20.5 | tcnbb | TCN1 | 1.186295503 | 0.916488223 | 5.154175589 |
| pex13 |  |  | 1.186046512 | 0.790697674 | 1.279069767 |
| trpm4a |  | TRPM4 | 1.186046512 | 0.651162791 | 1.162790698 |
| cdc42bpb |  |  | 1.185714286 | 0.728571429 | 1.3 |
| ucp2 | ucp2 | UCP2 | 1.185663925 | 1.124559342 | 1.678025852 |
| pgp | pgp | PGP | 1.185185185 | 0.759259259 | 1.555555556 |
| si:dkeyp-116a7.2 | si:dkeyp-116a7.2 |  | 1.184931507 | 1.116438356 | 1.780821918 |
| CABZ01072043.1 |  |  | 1.184466019 | 0.699029126 | 1.402912621 |
| cyp2x7 | cyp2x7 | CYP2A7 | 1.183333333 | 1.45 | 0.3 |
| abcg2a | abcg2a | ABCG2 | 1.182481752 | 1.496350365 | 1.240875912 |
| si:cabz01083442.1 | si:cabz01083442.1 | ABCA5 | 1.181818182 | 0.696969697 | 1.575757576 |
| auts2a |  |  | 1.181818182 | 0.75 | 1.204545455 |
| CRELD1 |  | CRELD1 | 1.181818182 | 0.568181818 | 1.363636364 |
| smad1 |  |  | 1.180722892 | 0.891566265 | 1.445783133 |
| slc45a4 |  |  | 1.18 | 0.7 | 1.34 |
| hcfc1b |  | HCFC1 | 1.178571429 | 0.642857143 | 1.214285714 |
| col1a1a | col1a1a | COL1A1 | 1.178419936 | 1.002651113 | 1.046129374 |
| tcf20 |  |  | 1.177777778 | 0.977777778 | 1.377777778 |
| slc30a10 | slc30a10 | SLC30A10 | 1.177419355 | 1.048387097 | 1.5 |
| usp10 |  |  | 1.177419355 | 0.790322581 | 1.14516129 |
| yme1l1a |  |  | 1.176470588 | 0.764705882 | 1.382352941 |

| acad11 |  |  | 1.175438596 | 0.912280702 | 1.105263158 |
| --- | --- | --- | --- | --- | --- |
| cfhl2 |  |  | 1.173913043 | 0.913043478 | 0.956521739 |
| fam213b |  |  | 1.173076923 | 1.134615385 | 0.903846154 |
| cgnl1 |  |  | 1.171428571 | 0.957142857 | 1.114285714 |
| si:dkey-28b4.8 | si:dkey-28b4.8 | ATP13A5 | 1.169811321 | 0.660377358 | 1.301886792 |
| hk1 |  |  | 1.169590643 | 0.953216374 | 1.356725146 |
| bbox1 |  | BBOX1 | 1.169014085 | 0.633802817 | 1.098591549 |
| ctnnb1 |  |  | 1.16772554 | 0.758576874 | 1.18678526 |
| atxn2 |  |  | 1.166666667 | 0.777777778 | 1.388888889 |
| dyrk1ab |  | DYRK1A | 1.166666667 | 0.638888889 | 1.444444444 |
| akt2 |  | AKT2 | 1.165413534 | 0.578947368 | 1.473684211 |
| kpna4 |  |  | 1.164179104 | 0.825870647 | 1.23880597 |
| zgc:162331 | zgc:162331 | SUSD3 | 1.163934426 | 0.967213115 | 1.606557377 |
| eya4 |  |  | 1.162162162 | 1.202702703 | 1.175675676 |
| asah2 |  |  | 1.162079511 | 0.935779817 | 1.400611621 |
| tnrc6b |  |  | 1.16 | 0.78 | 1.18 |
| pi4kb |  | PI4KB | 1.159090909 | 0.636363636 | 1.295454545 |
| mark2b |  |  | 1.158730159 | 0.825396825 | 1.365079365 |
| fgfrl1a |  |  | 1.158536585 | 0.829268293 | 1.073170732 |
| lbr |  | LBR | 1.158536585 | 0.62195122 | 0.841463415 |
| gltpa |  | GLTP | 1.157894737 | 0.631578947 | 1.394736842 |
| gnal2 |  |  | 1.157142857 | 0.957142857 | 1.171428571 |
| mafba |  |  | 1.15625 | 0.671875 | 1.375 |
| pbld2 |  |  | 1.155555556 | 1.066666667 | 1.177777778 |
| si:dkey-201i2.1 | si:dkey-201i2.1 |  | 1.155555556 | 0.444444444 | 0.8 |
| rbfox2 |  |  | 1.155172414 | 0.724137931 | 1.362068966 |
| slc15a1b | slc15a1b | SLC15A1 | 1.154929577 | 0.657276995 | 1.657276995 |
| fa2h |  | FA2H | 1.154929577 | 0.647887324 | 1.38028169 |
| osgn1 |  |  | 1.154761905 | 1 | 1.154761905 |
| rdh10a | rdh10a | RDH10 | 1.153846154 | 0.769230769 | 1.746153846 |
| GTPBP2 (1 of many) |  |  | 1.152542373 | 0.86440678 | 1.101694915 |
| map2 |  |  | 1.150943396 | 0.679245283 | 1.132075472 |
| suco |  |  | 1.150684932 | 0.739726027 | 1.342465753 |
| fap |  |  | 1.15 | 0.883333333 | 0.916666667 |
| mov10b.1 |  |  | 1.15 | 0.95 | 1.4 |
| epb41l5 |  |  | 1.15 | 0.725 | 1.35 |
| hiat1b |  |  | 1.14893617 | 0.744680851 | 1.361702128 |
| ggt1b | ggt1b | GGT1 | 1.148648649 | 0.824324324 | 1.756756757 |
| fuca2 | fuca2 | FUCA2 | 1.148514851 | 0.742574257 | 1.569306931 |
| tp53bp2b |  |  | 1.145833333 | 0.729166667 | 1.229166667 |
| plpp3 |  |  | 1.144508671 | 0.884393064 | 1.144508671 |
| ugt5d1 | ugt5d1 | UGT1A10 | 1.142857143 | 0.696428571 | 0.892857143 |
| tmem41ab | tmem41ab | TMEM41A | 1.142857143 | 0.857142857 | 0.959183673 |
| nomo |  |  | 1.142857143 | 0.704081633 | 1.020408163 |
| polr2a |  | POLR2A | 1.141509434 | 0.641509434 | 1.245283019 |
| pitrm1 |  |  | 1.137931034 | 0.827586207 | 1.24137931 |
| rdh5 | rdh | RDH5 | 1.136363636 | 1.015151515 | 0.96969697 |
| si:dkey-14d8.23_1 | - |  | 1.136363636 | 1.045454545 | 2 |
| f9b |  |  | 1.136363636 | 0.727272727 | 0.75 |
| rbfox1l |  |  | 1.135 | 0.865 | 1 |
| pfkmb |  |  | 1.134615385 | 1.076923077 | 1.096153846 |
| rbm14a |  | RBM14 | 1.133333333 | 0.666666667 | 1.288888889 |
| gsk3b | gsk3ba | GSK3B | 1.1328125 | 0.6640625 | 1.046875 |
| tmx2b |  | TMX2 | 1.132352941 | 0.602941176 | 1.411764706 |
| fam168b |  |  | 1.131386861 | 0.795620438 | 1.262773723 |
| stip1 | stip1 | STIP1 | 1.130434783 | 0.789297659 | 1.505016722 |
| emc1 |  | EMC1 | 1.12962963 | 0.574074074 | 1.203703704 |

| bhlha9 | bhlha9 | BHLHA9 | 1.129032258 | 0.870967742 | 1.548387097 |
| --- | --- | --- | --- | --- | --- |
| lamc3 |  |  | 1.128571429 | 0.871428571 | 0.957142857 |
| tgfbi |  |  | 1.128458498 | 0.924901186 | 1.130434783 |
| nolc1 |  |  | 1.128342246 | 0.754010695 | 1.037433155 |
| zgc:66156 |  |  | 1.127272727 | 0.945454545 | 0.909090909 |
| smyd1b |  |  | 1.126829268 | 1.029268293 | 1.336585366 |
| dennd5b |  |  | 1.126760563 | 0.690140845 | 1.098591549 |
| tln1 |  | TLN1 | 1.126582278 | 0.658227848 | 1.088607595 |
| si:ch211-246e12.3 | si:ch211-246e12.3 |  | 1.126582278 | 0.632911392 | 1.075949367 |
| irf2bp1 |  |  | 1.126436782 | 0.793103448 | 1.068965517 |
| si:ch211-266g18.10 |  |  | 1.12565445 | 1.497382199 | 1.12565445 |
| vgll2b |  |  | 1.125 | 0.75 | 1.069444444 |
| ptprk |  |  | 1.125 | 0.71875 | 1.203125 |
| col6a3_1 |  |  | 1.122807018 | 0.789473684 | 1.01754386 |
| csnk1da |  |  | 1.120943953 | 0.887905605 | 1.126843658 |
| gmps |  | GMPS | 1.120879121 | 0.626373626 | 1.065934066 |
| stra6 |  |  | 1.12037037 | 1.074074074 | 0.962962963 |
| NAALADL1 | si:ch211-214j8.1 | NAALAD2 | 1.119047619 | 0.619047619 | 1.571428571 |
| DNAJA4 | – | DNAJA4 | 1.117647059 | 1.132352941 | 2.882352941 |
| nog2 |  |  | 1.117647059 | 0.725490196 | 1.235294118 |
| PTGES3L | si:ch211-131k2.3 | PTGES3L | 1.117021277 | 1.085106383 | 1.617021277 |
| sulf1 |  |  | 1.11627907 | 0.790697674 | 1.139534884 |
| lmnb1 |  | LMNB1 | 1.115942029 | 0.666666667 | 0.971014493 |
| porb |  |  | 1.115492958 | 0.825352113 | 0.830985915 |
| dpys |  |  | 1.114583333 | 0.791666667 | 0.760416667 |
| si:ch211-188p14.4 |  |  | 1.114285714 | 1.085714286 | 1.371428571 |
| synpo2la |  |  | 1.114285714 | 0.714285714 | 1.028571429 |
| dock9a |  | DOCK9 | 1.114285714 | 0.6 | 1.171428571 |
| si:ch211-127i16.2 |  | MAOB; IL4I1 | 1.113207547 | 0.603773585 | 1.094339623 |
| apex1 |  |  | 1.113095238 | 0.845238095 | 0.791666667 |
| zgc:92137 | zgc:92137 |  | 1.111285266 | 2.379310345 | 1.136363636 |
| zgc:195245 |  |  | 1.111111111 | 1.166666667 | 1.444444444 |
| lonp1 | lonp1 | LONP1 | 1.111111111 | 0.873015873 | 1.888888889 |
| arid1aa |  |  | 1.111111111 | 0.73015873 | 1.158730159 |
| ndrg1b | ndrg1b | NDRG1 | 1.111111111 | 0.981481481 | 2.166666667 |
| ZNF609 (1 of many) |  |  | 1.111111111 | 0.722222222 | 1.111111111 |
| cnn3b |  | CNN3 | 1.111111111 | 0.555555556 | 1.055555556 |
| coq10b |  |  | 1.109289617 | 1.06010929 | 1.142076503 |
| zc3h4 |  |  | 1.109090909 | 0.672727273 | 1.072727273 |
| pcsk5b |  |  | 1.108695652 | 0.847826087 | 0.956521739 |
| mia3 |  |  | 1.108695652 | 0.695652174 | 1.260869565 |
| tjp1b |  |  | 1.108695652 | 0.695652174 | 1.260869565 |
| prkra |  |  | 1.108695652 | 0.673913043 | 1.108695652 |
| hsp90aa1.2 | hsp90aa1.2 | HSP90AA1 | 1.106666667 | 1.05 | 3.496666667 |
| CABZ01088484.1 |  |  | 1.105633803 | 0.507042254 | 1 |
| hmgcl |  |  | 1.105263158 | 0.828947368 | 1.434210526 |
| kdm6a |  |  | 1.105263158 | 0.684210526 | 1.368421053 |
| dhrs13a.1 | dhrs13a.1 | DHRS13 | 1.104651163 | 1.337209302 | 1.244186047 |
| crx_1 |  |  | 1.103896104 | 0.675324675 | 1.214285714 |
| alpi.2 | alpi.2 | ALPI | 1.103286385 | 1.023474178 | 2.441314554 |
| dct |  |  | 1.103092784 | 0.891752577 | 1.164948454 |
| cttnbp2 |  |  | 1.101694915 | 0.711864407 | 1.288135593 |
| eif4g1a |  |  | 1.101382488 | 0.67281106 | 1.064516129 |
| ccdc90b |  |  | 1.1 | 1.133333333 | 1 |
| flii |  |  | 1.1 | 0.7 | 1.133333333 |
| CU639469.1 |  |  | 1.097560976 | 0.715447154 | 1.18699187 |
| saga |  |  | 1.096774194 | 0.916129032 | 1.187096774 |

| arhgap5 |  | ARHGAP5 | 1.096774194 | 0.64516129 | 1.241935484 |
| --- | --- | --- | --- | --- | --- |
| pck1 |  |  | 1.096288867 | 0.673019057 | 1.106318957 |
| spon1b |  |  | 1.096 | 0.768 | 1.136 |
| spred1 |  |  | 1.095652174 | 0.773913043 | 1.217391304 |
| tox |  |  | 1.095541401 | 0.668789809 | 1.063694268 |
| ehd1a |  |  | 1.094339623 | 0.726415094 | 1.283018868 |
| gphnb |  |  | 1.09375 | 0.71875 | 1.1875 |
| rdh8b |  | RDH12 | 1.093023256 | 0.790697674 | 1.395348837 |
| zgc:66479 |  |  | 1.092150171 | 0.887372014 | 1.027303754 |
| ubap2l |  |  | 1.091603053 | 0.679389313 | 1.099236641 |
| cltcb |  |  | 1.091503268 | 0.738562092 | 1.14379085 |
| rxraa |  |  | 1.090909091 | 0.672727273 | 1.018181818 |
| nr1d4a | nr1d4a | NR1D1 | 1.090909091 | 0.636363636 | 2.272727273 |
| si:ch211-274j7.3 | si:ch211-274j7.3 |  | 1.090909091 | 0.409090909 | 0.795454545 |
| arf4b | arf4b | ARF5 | 1.090909091 | 0.363636364 | 0.363636364 |
| apba2b |  |  | 1.09009009 | 0.693693694 | 1.171171171 |
| pck2 |  |  | 1.089622642 | 0.693396226 | 0.775943396 |
| fam57ba |  |  | 1.088888889 | 0.822222222 | 1.355555556 |
| ifrd2 |  |  | 1.088709677 | 0.927419355 | 1.201612903 |
| tnpo1 |  | TNPO1 | 1.088235294 | 0.617647059 | 1.411764706 |
| gclm | gclm | GCLM | 1.086956522 | 0.5 | 5 |
| cx43.4 |  |  | 1.086956522 | 0.730434783 | 1.095652174 |
| cdk5r2b |  |  | 1.086956522 | 0.710144928 | 1.115942029 |
| eng2b |  |  | 1.086206897 | 0.931034483 | 1.034482759 |
| tmem135 | tmem | TMTM135 | 1.085714286 | 0.8 | 1.057142857 |
| asxl1 |  | ASXL1 | 1.085714286 | 0.657142857 | 1.157142857 |
| si:ch73-43g23.1 |  |  | 1.085106383 | 0.744680851 | 1.042553191 |
| vps37b | vps37ba | VPS37B | 1.085106383 | 0.468085106 | 1.021276596 |
| slc10a2 |  |  | 1.083333333 | 0.958333333 | 1.4375 |
| si:dkeyp-79f4.1 | si:dkeyp-79f4.1 |  | 1.083333333 | 0.583333333 | 2.458333333 |
| cbsa |  | CBS; CBSL | 1.081967213 | 0.557377049 | 0.983606557 |
| hoxd10a |  |  | 1.081632653 | 0.857142857 | 1.020408163 |
| prr12a |  |  | 1.081081081 | 0.689189189 | 1.121621622 |
| guca1c |  |  | 1.080924855 | 1.300578035 | 1.10982659 |
| PLIN3 |  |  | 1.080808081 | 0.737373737 | 1.494949495 |
| zfr |  |  | 1.080808081 | 0.671717172 | 0.929292929 |
| larp4ab |  | LARP4 | 1.080645161 | 0.661290323 | 1.35483871 |
| tbc1d5 | tbc1d5 | TBC1D5 | 1.078125 | 0.75 | 1.546875 |
| sec14l7 |  | SEC14L4 | 1.076923077 | 0.630769231 | 0.738461538 |
| npepps |  |  | 1.076470588 | 0.723529412 | 1.241176471 |
| scrib |  | SCRIB | 1.075471698 | 0.58490566 | 1.150943396 |
| rgs9a |  |  | 1.075268817 | 0.795698925 | 1.35483871 |
| ubl3a |  |  | 1.074561404 | 0.75877193 | 1.311403509 |
| ubtf |  |  | 1.074534161 | 0.763975155 | 1.149068323 |
| slc20a1b |  | SLC20A1 | 1.074324324 | 0.736486486 | 1.114864865 |
| amer2 |  |  | 1.073529412 | 0.838235294 | 1.058823529 |
| gc3 |  |  | 1.073529412 | 0.735294118 | 1.117647059 |
| mylipb |  |  | 1.073033708 | 1.06741573 | 1.056179775 |
| sox11b |  |  | 1.072874494 | 0.850202429 | 1.089068826 |
| atp2b3a | atp2b3a | ATP2B3 | 1.072072072 | 0.666666667 | 1.063063063 |
| clstn1 |  |  | 1.07183908 | 0.712643678 | 1.212643678 |
| si:dkey-100j22.4 |  |  | 1.071794872 | 1.102564103 | 1.087179487 |
| sult1st5 | sult1st5 | SULT1C4 | 1.071428571 | 1.357142857 | 5.571428571 |
| smg5 |  |  | 1.071428571 | 0.9 | 1.114285714 |
| kdm3b |  |  | 1.071428571 | 0.757142857 | 1.171428571 |
| srpk1b |  | SRPK1 | 1.070422535 | 0.563380282 | 0.929577465 |
| col11a2 | col11a2 | COL11A2 | 1.07037037 | 0.681481481 | 0.959259259 |

| smu1b |  |  | 1.068965517 | 0.827586207 | 1.327586207 |
| --- | --- | --- | --- | --- | --- |
| kif5aa |  |  | 1.068825911 | 0.688259109 | 1.044534413 |
| si:dkey-89b17.4 |  |  | 1.068493151 | 0.767123288 | 1.205479452 |
| phactr4b |  |  | 1.068181818 | 0.704545455 | 1.113636364 |
| mpeg1.2 | mpeg1.2 | MPEG1 | 1.068181818 | 0.568181818 | 1.772727273 |
| mthfd2 |  |  | 1.067567568 | 0.716216216 | 1.22972973 |
| chchd3b |  |  | 1.066666667 | 0.933333333 | 1.1 |
| adgrb3 |  |  | 1.066666667 | 0.766666667 | 1.133333333 |
| mtss1la |  |  | 1.066666667 | 0.766666667 | 1.083333333 |
| irf2bpl |  |  | 1.066666667 | 0.723076923 | 1.102564103 |
| stm | stm |  | 1.066176471 | 0.987132353 | 1.854779412 |
| pex5 |  | PEX5 | 1.06557377 | 0.639344262 | 1.098360656 |
| plk3 |  |  | 1.064935065 | 0.844155844 | 1.194805195 |
| myclb |  |  | 1.064516129 | 0.924731183 | 1.23655914 |
| pvalb8 | pvalb8 | OCM | 1.064285714 | 1.483333333 | 0.370634921 |
| mt-atp8 |  |  | 1.064164649 | 0.782082324 | 1.141848265 |
| PARG |  |  | 1.063829787 | 0.680851064 | 1.127659574 |
| zfand2a | zfand2a | ZFAND2B | 1.063636364 | 1.072727273 | 2.463636364 |
| grp |  | GRP | 1.0625 | 1.21875 | 0.53125 |
| cad |  |  | 1.0625 | 0.675 | 0.8875 |
| camk2b1 |  | CAMK2B | 1.061643836 | 0.630136986 | 1.308219178 |
| zic1 |  |  | 1.061538462 | 0.753846154 | 1.21025641 |
| pygb |  |  | 1.061538462 | 0.746153846 | 0.876923077 |
| rad54l2 |  |  | 1.060606061 | 0.787878788 | 1.060606061 |
| mef2ca |  |  | 1.060240964 | 0.879518072 | 1.21686747 |
| zgc:158263 | zgc:158263 | CERK | 1.06 | 1.16 | 1.64 |
| nras |  | NRAS | 1.06 | 0.64 | 1.32 |
| vcanb |  | VCAN | 1.059210526 | 0.638157895 | 0.743421053 |
| map1b |  |  | 1.058823529 | 1.088235294 | 1.176470588 |
| murcb |  |  | 1.058823529 | 0.764705882 | 0.917647059 |
| dag1 |  |  | 1.058252427 | 0.718446602 | 1.009708738 |
| vat1 |  | VAT1 | 1.057803468 | 0.630057803 | 0.849710983 |
| hif1ab |  | HIF1A | 1.056451613 | 0.653225806 | 1.310483871 |
| col6a2 |  | COL6A2 | 1.055944056 | 0.615384615 | 0.986013986 |
| znf687b |  | ZNF687 | 1.055555556 | 0.638888889 | 1.305555556 |
| nlk1 |  | NLK | 1.055555556 | 0.62962963 | 1.37037037 |
| nrxn2b |  | NRXN2 | 1.054545455 | 0.618181818 | 1.054545455 |
| ccdc43 |  | CCDC43 | 1.054054054 | 0.594594595 | 1.27027027 |
| sh3bp5b |  | SH3BP5 | 1.052631579 | 0.631578947 | 0.877192982 |
| ctnnd1 |  | CTNND1 | 1.051546392 | 0.577319588 | 1.154639175 |
| smarcd1 |  |  | 1.051282051 | 0.794871795 | 1.185897436 |
| dgkaa |  |  | 1.050847458 | 0.762711864 | 0.949152542 |
| rnf111 |  | RNF111 | 1.05 | 0.6 | 1.233333333 |
| si:ch1073-13h15.3 | si:ch1073-13h15.3 |  | 1.049180328 | 0.704918033 | 2.704918033 |
| hnrnpa0a_1 |  | HNRNPA3 | 1.048991354 | 0.567723343 | 0.959654179 |
| insm1b |  | INSM1 | 1.048780488 | 0.658536585 | 1.182926829 |
| krt1-c5 |  | KRT17 | 1.047619048 | 1.047619048 | 0.857142857 |
| casq1a |  |  | 1.046966732 | 1.070450098 | 1.02739726 |
| arrdc1a |  |  | 1.046511628 | 0.76744186 | 1.325581395 |
| tcf7l2 |  | TCF7L2 | 1.045454545 | 0.666666667 | 1.121212121 |
| magi1a | magi1a | MAGI1 | 1.045454545 | 0.5 | 1.090909091 |
| slc1a2b |  | SLC1A2 | 1.044585987 | 0.585987261 | 1.135881104 |
| smpdl3b |  |  | 1.044444444 | 0.8 | 1.051851852 |
| fpgs |  |  | 1.044117647 | 0.75 | 1.279411765 |
| fam120a |  |  | 1.043478261 | 0.68115942 | 1.15942029 |
| emid1 |  |  | 1.043478261 | 0.673913043 | 0.945652174 |
| pcxb | pcxb | PC | 1.043478261 | 0.282608696 | 0.847826087 |

| c10h21orf59 |  |  | 1.042857143 | 0.914285714 | 1.171428571 |
| --- | --- | --- | --- | --- | --- |
| B4GALNT2 (1 of many) |  |  | 1.042553191 | 1 | 1.489361702 |
| arid2 |  | ARID2 | 1.041666667 | 0.666666667 | 1.333333333 |
| si:dkey-283b1.6 | si:dkey-283b1.6 |  | 1.041666667 | 0.625 | 2 |
| arr3b |  |  | 1.040847201 | 1.148260212 | 1.075642965 |
| igf2bp3 |  |  | 1.040609137 | 0.705583756 | 0.969543147 |
| ppp1r10 | ppp1r10 | PPP1R10 | 1.04 | 0.79 | 1.53 |
| sde2 |  |  | 1.04 | 0.733333333 | 1.133333333 |
| npc1 |  | NPC1 | 1.038961039 | 0.636363636 | 1.207792208 |
| mtdha |  |  | 1.037037037 | 0.7 | 1.222222222 |
| fbln2 |  |  | 1.036363636 | 0.8 | 0.872727273 |
| bhlhe40 |  |  | 1.036241611 | 0.920805369 | 1.06442953 |
| col16a1 | col16a1 | COL15A1 | 1.035714286 | 0.678571429 | 1.130952381 |
| mapk6 | mapk6 | MAPK6 | 1.033783784 | 0.459459459 | 1.114864865 |
| hapln1a |  |  | 1.033333333 | 0.75 | 0.925 |
| si:dkey-28e7.3 | si:dkey-28e7.3 | HAP1 | 1.033333333 | 0.616666667 | 1.583333333 |
| pparg | pparg | PPARG | 1.032258065 | 0.483870968 | 1.403225806 |
| insm1a |  |  | 1.030927835 | 0.75257732 | 1.391752577 |
| ppp2r2d |  | PPP2R2D | 1.03030303 | 0.560606061 | 0.863636364 |
| CABZ01088933.1 |  |  | 1.029850746 | 0.880597015 | 1.388059701 |
| si:ch211-207l14.1 |  |  | 1.02970297 | 0.920792079 | 1.059405941 |
| daam1b |  | DAAM1 | 1.028985507 | 0.637681159 | 0.971014493 |
| GOLPH3 (1 of many) |  |  | 1.028735632 | 0.718390805 | 0.988505747 |
| eif4a1a |  |  | 1.028277635 | 0.686375321 | 1.100257069 |
| cant1b | cant1b | CANT1 | 1.027522936 | 0.582568807 | 1.619266055 |
| comta |  |  | 1.02739726 | 0.671232877 | 1.054794521 |
| npm1b |  |  | 1.025 | 0.675 | 1.375 |
| sec61a1l |  |  | 1.024390244 | 0.707317073 | 1.292682927 |
| hnrnpub |  | HNRNPU | 1.024390244 | 0.589430894 | 1.130081301 |
| gdap2 | gdap2 | GDAP2 | 1.023809524 | 0.80952381 | 1.523809524 |
| ptpn13 |  | PTPN13 | 1.023255814 | 0.651162791 | 1.093023256 |
| ptk7a | ptk7a | PTK7 | 1.023255814 | 0.465116279 | 1.046511628 |
| col18a1 | col18a1a | COL18A1 | 1.02293578 | 0.623853211 | 1.004587156 |
| iqsec1b |  | IQSEC1 | 1.022727273 | 0.590909091 | 1.25 |
| papd5 |  |  | 1.02247191 | 0.764044944 | 1.303370787 |
| dpysl3 |  | DPYSL3 | 1.020833333 | 0.638888889 | 1.034722222 |
| nmrk2 |  | NMRK1 | 1.020661157 | 0.842975207 | 0.652892562 |
| homezb |  |  | 1.020408163 | 0.693877551 | 1.163265306 |
| rab1aa |  | RAB1A | 1.02020202 | 0.555555556 | 0.909090909 |
| brsk2b |  |  | 1.02 | 0.86 | 1.26 |
| rapgef2 |  | RAPGEF2 | 1.02 | 0.62 | 1.14 |
| fut8a |  |  | 1.019607843 | 0.764705882 | 1.245098039 |
| sema3fa |  | SEMA3F | 1.019607843 | 0.666666667 | 1.039215686 |
| epn2 |  | EPN2 | 1.019230769 | 0.653846154 | 1.192307692 |
| clcn3 | clcn3 | CLCN3 | 1.019230769 | 0.5 | 1.25 |
| mat1a |  | MAT1A | 1.019193858 | 0.575815739 | 0.77159309 |
| chd9 |  | CHD9 | 1.018867925 | 0.58490566 | 1.226415094 |
| vaspb |  |  | 1.018604651 | 0.734883721 | 1.297674419 |
| hebp2 | hebp2 | HEBP2 | 1.018518519 | 1.824074074 | 0.805555556 |
| camk2g1 |  | CAMK2G | 1.018181818 | 0.654545455 | 1.375757576 |
| pfklb |  |  | 1.017857143 | 0.696428571 | 1.017857143 |
| sppl3 |  | SPPL3 | 1.017857143 | 0.571428571 | 1.267857143 |
| dcaf12 |  |  | 1.016666667 | 0.866666667 | 1.033333333 |
| nptnb |  |  | 1.016304348 | 0.744565217 | 1.184782609 |
| SLC41A3 |  |  | 1.016042781 | 0.828877005 | 1.144385027 |
| cfh |  |  | 1.015957447 | 0.686170213 | 0.962765957 |
| xpo1b | xpo1b | XPO1 | 1.015873016 | 0.777777778 | 2.063492063 |

| mob1bb |  |  | 1.014705882 | 0.808823529 | 1.264705882 |
| --- | --- | --- | --- | --- | --- |
| tjp1a |  | TJP1 | 1.014492754 | 0.550724638 | 1.043478261 |
| cluha |  |  | 1.01369863 | 0.698630137 | 0.808219178 |
| xpot |  |  | 1.013513514 | 0.756756757 | 1.243243243 |
| rhoub |  |  | 1.012987013 | 0.74025974 | 1.246753247 |
| chd4b |  |  | 1.011764706 | 0.694117647 | 0.958823529 |
| shmt2 |  | SHMT2 | 1.009433962 | 0.58490566 | 1.20754717 |
| spns1 |  | SPNS1 | 1.009345794 | 0.654205607 | 1.23364486 |
| aktip | aktip | AKTIP | 1.007518797 | 0.669172932 | 1.593984962 |
| itm2cb |  |  | 1.006134969 | 0.877300613 | 1.214723926 |
| si:ch211-214p16.2 | si:ch211-214p16.2 |  | 1 | 1.739726027 | 0.917808219 |
| si:ch211-11c3.4 | si:ch211-11c3.4 |  | 1 | 1.666666667 | 2.083333333 |
| LCT (1 of many) | - | LCTL | 1 | 1.571428571 | 0.428571429 |
| fgl1 |  |  | 1 | 1.394736842 | 1.131578947 |
| si:ch211-103n10.5 |  |  | 1 | 1.266666667 | 1.25 |
| emilin2b |  |  | 1 | 1.025641026 | 1.051282051 |
| cyp1a |  |  | 1 | 0.9 | 0.755555556 |
| si:ch211-225b11.1 | si:ch211-225b11.1 | SLC6A16 | 1 | 0.882352941 | 4.352941176 |
| ip6k1 |  |  | 1 | 0.846153846 | 1.153846154 |
| slc25a48 |  | SLC25A48 | 1 | 0.828571429 | 1.285714286 |
| fam135a |  |  | 1 | 0.795454545 | 1.363636364 |
| si:dkey-219e21.2 |  |  | 1 | 0.793103448 | 1.137931034 |
| zdhhc9 |  |  | 1 | 0.76 | 1.2 |
| atn1 |  |  | 1 | 0.755102041 | 1.12244898 |
| uacab |  |  | 1 | 0.746666667 | 1.16 |
| si:ch211-147k10.6 |  |  | 1 | 0.742857143 | 1.057142857 |
| scg2a |  |  | 1 | 0.72972973 | 1.310810811 |
| pcdh18b |  |  | 1 | 0.708860759 | 1.215189873 |
| tarbp2 |  |  | 1 | 0.704225352 | 1.338028169 |
| ppp2r5b |  |  | 1 | 0.686567164 | 0.985074627 |
| fli1a |  |  | 1 | 0.678571429 | 1 |
| lef1 |  |  | 1 | 0.670731707 | 1.195121951 |
| sema3aa |  |  | 1 | 0.669811321 | 1.113207547 |
| ank2b |  | ANK2 | 1 | 0.653333333 | 1.16 |
| atf7b |  | ATF7; NPFF | 1 | 0.646153846 | 1.338461538 |
| nrxn1a |  | NRXN1 | 1 | 0.645833333 | 1.135416667 |
| ugt5c3 | ugt5c3 | UGT3A1 | 1 | 0.64 | 1.84 |
| kdelr2a |  | KDELR2 | 1 | 0.613559322 | 1.155932203 |
| scp2b |  | SCP2 | 1 | 0.603960396 | 0.811881188 |
| gnb1a |  | GNB1 | 1 | 0.601694915 | 1.028248588 |
| pum1 |  | PUM1 | 1 | 0.595890411 | 1.116438356 |
| dpysl2b |  | DPYSL2 | 1 | 0.541284404 | 0.853211009 |
| fbxo11a_1 | - | FBXO11 | 1 | 0.491803279 | 1.344262295 |
| sap130a | sap130a | SAP130 | 1 | 0.488888889 | 1.111111111 |
| slc39a4 | slc39a4 | SLC39A4 | 1 | 0.4 | 0.9 |
| igfn1.3 |  |  | 0.997844828 | 0.995689655 | 1.073275862 |
| col5a2a |  |  | 0.993318486 | 0.71714922 | 1.062360802 |
| si:dkey-14d8.7 | si:dkey-14d8.7 |  | 0.99188641 | 2.462474645 | 0.997971602 |
| flot2a |  | FLOT2 | 0.991825613 | 0.566757493 | 1.057220708 |
| tmem176l.2 | tmem176l.2 | MS4A3 | 0.991525424 | 0.652542373 | 1.330508475 |
| epn1 |  | EPN1 | 0.990825688 | 0.614678899 | 1.137614679 |
| cyp3c4 |  | CYP3A4 | 0.989795918 | 0.642857143 | 1.030612245 |
| pora |  |  | 0.989583333 | 0.71875 | 0.947916667 |
| si:ch1073-190k2.1 | si:ch1073-190k2.1 |  | 0.988023952 | 0.365269461 | 1.682634731 |
| trim13 |  |  | 0.987654321 | 0.691358025 | 1.234567901 |
| baz2ba |  | BAZ2B | 0.987341772 | 0.544303797 | 1.17721519 |
| rcn3 |  | RCN3 | 0.986666667 | 0.826666667 | 0.56 |

| cry1ab | cry1b | CRY1 | 0.985915493 | 0.873239437 | 0.605633803 |
| --- | --- | --- | --- | --- | --- |
| TMEM120A (1 of many) | - | TMEM120B | 0.985915493 | 0.492957746 | 0.676056338 |
| gmds |  |  | 0.984848485 | 0.727272727 | 1.212121212 |
| mark3b |  | MARK3 | 0.984848485 | 0.621212121 | 1.333333333 |
| im:7150988 |  |  | 0.984716157 | 0.80349345 | 0.884279476 |
| and1 |  |  | 0.98469657 | 0.910817942 | 1.008970976 |
| smarcc2 |  |  | 0.984375 | 0.703125 | 1.171875 |
| ca4b |  |  | 0.983870968 | 0.85483871 | 1.322580645 |
| RB1CC1 |  |  | 0.983870968 | 0.741935484 | 1.209677419 |
| slc25a33 |  | SLC25A33 | 0.983388704 | 0.890365449 | 1.199335548 |
| zgc:101577 | ilrun | ILRUN | 0.983333333 | 0.65 | 1 |
| per3 |  | PER3 | 0.983333333 | 0.65 | 0.75 |
| fam120c |  | FAM120C | 0.983050847 | 0.644067797 | 1.169491525 |
| col4a1 |  | COL4A1 | 0.982758621 | 0.655172414 | 1.086206897 |
| phf20b |  | PHF20 | 0.982758621 | 0.568965517 | 1.465517241 |
| col4a5 |  | COL4A4 | 0.982404692 | 0.607038123 | 1.08797654 |
| kank1a |  | KANK1 | 0.982142857 | 0.642857143 | 1.089285714 |
| sp3b |  | SP3 | 0.981818182 | 0.563636364 | 1.163636364 |
| tnw_1 |  |  | 0.981632653 | 0.726530612 | 0.955102041 |
| scamp4 |  | SCAMP4 | 0.980392157 | 0.62745098 | 1.176470588 |
| itgbl1 |  |  | 0.97979798 | 0.818181818 | 1.070707071 |
| pls3 |  | PLS3 | 0.979591837 | 0.586734694 | 1.193877551 |
| rock2b_2 |  |  | 0.97826087 | 0.673913043 | 1.152173913 |
| slain2 |  | SLAIN2 | 0.97826087 | 0.565217391 | 1.217391304 |
| prrc2c |  | PRRC2C | 0.97826087 | 0.543478261 | 1 |
| smad4a |  |  | 0.977272727 | 0.795454545 | 1.204545455 |
| cicb |  | CIC | 0.977272727 | 0.636363636 | 1.159090909 |
| ncoa2 |  | NCOA2 | 0.976744186 | 0.627906977 | 1.255813953 |
| zswim5 |  | ZSWIM5 | 0.976744186 | 0.61627907 | 1.046511628 |
| rnf145b |  | RNF145 | 0.976190476 | 0.642857143 | 1.380952381 |
| sin3ab |  | SIN3A | 0.975609756 | 0.585365854 | 1.341463415 |
| entpd6 |  |  | 0.975308642 | 0.790123457 | 1.037037037 |
| ext2 |  |  | 0.974683544 | 0.683544304 | 1.392405063 |
| axin2 |  |  | 0.974358974 | 0.679487179 | 1.08974359 |
| ptprz1b |  |  | 0.974025974 | 0.714285714 | 1.142857143 |
| slc35e3 | slc35e3 | SLC35E3 | 0.973684211 | 0.368421053 | 1.684210526 |
| ctcf |  | CTCF | 0.972826087 | 0.64673913 | 0.923913043 |
| sgk1 |  |  | 0.972222222 | 0.772222222 | 1.077777778 |
| cbsb |  |  | 0.972222222 | 0.756944444 | 1.354166667 |
| slc43a1b |  |  | 0.971428571 | 0.757142857 | 1.128571429 |
| neurod4 |  |  | 0.971428571 | 0.674285714 | 1.274285714 |
| si:ch211-209p16.4 |  |  | 0.970588235 | 0.931372549 | 1.068627451 |
| arpp21 |  | ARPP19 | 0.970588235 | 0.588235294 | 0.985294118 |
| xirp2a |  |  | 0.97 | 0.72 | 1.03 |
| lrrn1 |  | LRRN1 | 0.97 | 0.65 | 1.15 |
| sec14l8 |  | SEC14L6 | 0.96969697 | 0.545454545 | 0.651515152 |
| sepw2b | selenow2b | MIEN1 | 0.969465649 | 0.885496183 | 2.5 |
| sult1st6 |  | SULT1C2 | 0.969387755 | 0.693877551 | 0.647959184 |
| ankrd9 |  |  | 0.969273743 | 1.237430168 | 0.980446927 |
| adka |  | ADK | 0.969230769 | 0.630769231 | 0.888461538 |
| clstn3 |  | CLSTN3 | 0.969072165 | 0.618556701 | 1.175257732 |
| kpna5 |  |  | 0.967741935 | 0.688172043 | 1.322580645 |
| serbp1b |  | SERBP1 | 0.966755319 | 0.662234043 | 1.026595745 |
| alkbh5 |  |  | 0.966101695 | 0.745762712 | 1.237288136 |
| si:dkey-177p2.6 |  |  | 0.965517241 | 0.908045977 | 1.413793103 |
| im:6907928 |  |  | 0.965517241 | 0.74137931 | 1.25862069 |
| si:ch211-270n8.1 |  |  | 0.963768116 | 1.039855072 | 0.916666667 |

| fam83hb |  |  | 0.963636364 | 0.8 | 0.818181818 |
| --- | --- | --- | --- | --- | --- |
| serpinb14 |  |  | 0.962962963 | 0.703703704 | 1.333333333 |
| CABZ01114898.1 | - |  | 0.962962963 | 0.425925926 | 0.87037037 |
| MAL (1 of many)_2 |  |  | 0.962264151 | 0.867924528 | 0.962264151 |
| sept15 |  |  | 0.961904762 | 0.698412698 | 1.111111111 |
| zgc:153846 | zgc:153846 | CRYGC | 0.96 | 2.52 | 0.76 |
| si:ch73-106l15.4 | si:ch73-106l15.4 |  | 0.96 | 1.5 | 1.02 |
| vps72 |  |  | 0.959183673 | 0.693877551 | 1.346938776 |
| siah2l |  |  | 0.957446809 | 0.765957447 | 1.14893617 |
| zgc:194392 |  | C1orf115 | 0.956989247 | 0.666666667 | 0.698924731 |
| tnnt2d |  |  | 0.956960323 | 1.061197041 | 1.165433759 |
| actn3b |  | ACTN3 | 0.956736712 | 0.665018541 | 0.992583436 |
| csnk1db |  |  | 0.956521739 | 0.70923913 | 1.184782609 |
| si:dkey-65b13.13 |  |  | 0.955882353 | 0.955882353 | 1.279411765 |
| g6pd | g6pd | G6PD | 0.955882353 | 0.617647059 | 2.705882353 |
| ZNF423 |  | ZNF423 | 0.953846154 | 0.615384615 | 1.076923077 |
| si:ch73-52f24.4 |  |  | 0.952380952 | 0.962962963 | 1.074074074 |
| mthfr |  |  | 0.952380952 | 0.746031746 | 0.793650794 |
| esrrga |  | ESRRG | 0.952380952 | 0.603174603 | 1.333333333 |
| lrrc58b | lrrc58b | LRRC58 | 0.952380952 | 0.412698413 | 1.222222222 |
| hadhaa |  |  | 0.952380952 | 0.79047619 | 1.076190476 |
| meis2a |  | MEIS2 | 0.95221843 | 0.631399317 | 1.139931741 |
| eif4g2a |  | EIF4G2 | 0.952054795 | 0.616438356 | 1.321917808 |
| pdcb |  |  | 0.951612903 | 0.929032258 | 1.25483871 |
| msrb1b |  |  | 0.951456311 | 1.038834951 | 1.252427184 |
| slc6a1b |  | SLC6A1 | 0.950292398 | 0.576023392 | 1.052631579 |
| adkb |  |  | 0.950177936 | 0.722419929 | 0.782918149 |
| sspn |  |  | 0.95 | 0.7 | 0.916666667 |
| galca |  | GALC | 0.95 | 0.575 | 1.15 |
| top2b |  |  | 0.949152542 | 0.734463277 | 0.988700565 |
| kiaa1549la |  | KIAA1549L | 0.949152542 | 0.610169492 | 1.06779661 |
| srp68 |  | SRP68 | 0.948453608 | 0.597938144 | 1.010309278 |
| aoc2 | aoc2 | AOC2 | 0.947945205 | 0.830136986 | 2.24109589 |
| rusc1 |  | RUSC1 | 0.947368421 | 0.526315789 | 1.210526316 |
| casc3 | casc3 | CASC3 | 0.947368421 | 0.49122807 | 1.210526316 |
| myoc |  |  | 0.946564885 | 0.938931298 | 1.183206107 |
| ch1073-291c23.1 | vit | VIT | 0.945578231 | 0.659863946 | 0.863945578 |
| nr1d2b |  | NR1D2 | 0.944827586 | 0.727586207 | 1.027586207 |
| dhcr7 |  |  | 0.944444444 | 0.733333333 | 0.766666667 |
| plekha6 |  | PLEKHA6 | 0.944444444 | 0.655555556 | 1.188888889 |
| arhgef9a |  | ARHGEF9 | 0.944444444 | 0.592592593 | 1.185185185 |
| pde6c | pde6c | PDE6C | 0.944360902 | 0.784962406 | 1.043609023 |
| cx30.3 |  |  | 0.943661972 | 0.774647887 | 1.295774648 |
| arl3l1 |  |  | 0.942307692 | 0.730769231 | 1.115384615 |
| asap1b |  | ASAP1 | 0.942307692 | 0.653846154 | 1.153846154 |
| cpo |  |  | 0.942196532 | 0.682080925 | 1.306358382 |
| fgfr2 |  | FGFR2 | 0.94214876 | 0.595041322 | 1 |
| ace2 |  |  | 0.942093541 | 0.712694878 | 1.405345212 |
| AC024175.22 |  |  | 0.941176471 | 0.941176471 | 1.294117647 |
| entpd8 |  |  | 0.941176471 | 0.794117647 | 0.794117647 |
| dnaja1 | dnaja1 | DNAJA1 | 0.941176471 | 0.588235294 | 1.784313725 |
| srm | srm | SRM | 0.941176471 | 0.450980392 | 0.960784314 |
| stk35l |  |  | 0.940594059 | 0.861386139 | 1.198019802 |
| sgcd |  |  | 0.94 | 0.84 | 1.12 |
| cdc42l |  | CDC42 | 0.94 | 0.553333333 | 1.18 |
| prrc2a |  | PRRC2A | 0.939655172 | 0.568965517 | 1.112068966 |
| tsc22d2 |  | TSC22D3 | 0.938461538 | 0.615384615 | 1.323076923 |

| rcor1 |  | RCOR1 | 0.938461538 | 0.6 | 1.169230769 |
| --- | --- | --- | --- | --- | --- |
| col17a1a | col17a1a | COL17A1 | 0.938297872 | 0.695744681 | 0.929787234 |
| si:dkey-97i18.5 | si:dkey-97i18.5 |  | 0.938271605 | 0.345679012 | 0.987654321 |
| asrgl1 |  |  | 0.9375 | 0.7 | 0.8625 |
| papss2a | papss2a | PAPSS2 | 0.936936937 | 0.45045045 | 1.400900901 |
| rabgap1l |  |  | 0.936842105 | 0.684210526 | 1.252631579 |
| hao2 |  |  | 0.936507937 | 0.746031746 | 1 |
| gnat2 |  |  | 0.936259771 | 0.885147324 | 1.144317498 |
| pou3f3b |  | POU5F2 | 0.935483871 | 0.661290323 | 1.266129032 |
| slc1a3b |  | SLC1A3 | 0.935064935 | 0.545454545 | 1.116883117 |
| pcxa |  | PC | 0.935064935 | 0.545454545 | 0.974025974 |
| snx10a | snx10a | SNX10 | 0.933333333 | 1.933333333 | 1.6 |
| si:dkeyp-69b9.3 |  |  | 0.933333333 | 0.833333333 | 1.1 |
| cdh17 |  | CDH17 | 0.933085502 | 0.600371747 | 0.933085502 |
| serinc2 |  |  | 0.932885906 | 0.771812081 | 1.228187919 |
| trip12 |  | TRIP12 | 0.932432432 | 0.594594595 | 1.243243243 |
| golim4a |  | GOLIM4 | 0.93220339 | 0.548022599 | 0.875706215 |
| si:ch1073-303d10.1 | si:ch1073-303d10.1 |  | 0.931818182 | 1 | 2 |
| enah |  |  | 0.930555556 | 0.694444444 | 1.083333333 |
| col5a1 |  | COL5A1 | 0.93015873 | 0.612698413 | 1.012698413 |
| acaca |  | ACACA | 0.926829268 | 0.634146341 | 1.146341463 |
| tpmt.1 | tpmt.1 | TPMT | 0.926829268 | 1.487804878 | 5.235772358 |
| c3b.1 |  |  | 0.926470588 | 0.75 | 1.088235294 |
| bhlhe41 |  |  | 0.925619835 | 0.752066116 | 1.347107438 |
| atp6v0b |  | ATP6V0B | 0.924855491 | 0.595375723 | 1.121387283 |
| slco5a2 | slco5a1b | SLCO4A1 | 0.924242424 | 0.515151515 | 1.151515152 |
| nup98 |  | NUP98 | 0.923076923 | 0.634615385 | 1.134615385 |
| nt5c2a |  | NT5C1A | 0.921875 | 0.65625 | 1.265625 |
| si:dkey-33c14.3 |  |  | 0.921854305 | 0.680463576 | 1.029801325 |
| abi3bpb |  |  | 0.921686747 | 0.771084337 | 0.86746988 |
| col17a1b | col17a1b | COL17A1 | 0.921568627 | 0.862745098 | 1.058823529 |
| capn2a |  | CAPN2 | 0.920634921 | 0.507936508 | 1.158730159 |
| akap1b |  |  | 0.920454545 | 0.681818182 | 1.090909091 |
| snx18a |  | SNX18 | 0.92 | 0.666666667 | 0.96 |
| ncoa3 |  | NCOA3 | 0.92 | 0.52 | 1.06 |
| aqp8a.2 |  | AQP6 | 0.919087137 | 0.597510373 | 0.89626556 |
| appbp2 |  | APPBP2 | 0.918032787 | 0.639344262 | 1.213114754 |
| sept6 |  | SEPTIN6 | 0.917582418 | 0.620879121 | 1.153846154 |
| kirrel3l |  | KIRREL2 | 0.916666667 | 0.604166667 | 1.166666667 |
| sptlc1 |  |  | 0.916666667 | 0.777777778 | 1.319444444 |
| nfil3-5 | nfil3-5 | NFIL3 | 0.916666667 | 0.611111111 | 1.083333333 |
| slc26a5 | slc26a5 | SLC26A5 | 0.916666667 | 0.583333333 | 1.638888889 |
| ube2h | ube2h | UBE2H | 0.915730337 | 0.5 | 1.314606742 |
| hspa9 |  |  | 0.915632754 | 0.694789082 | 1.248138958 |
| dacha |  | DACH2 | 0.914893617 | 0.553191489 | 1.14893617 |
| ank3b |  |  | 0.914285714 | 0.714285714 | 1.028571429 |
| mafaa | mafaa | MAFA | 0.913043478 | 0.5 | 1.195652174 |
| mex3b | mex3b | MEX3B | 0.913043478 | 0.47826087 | 1.152173913 |
| si:ch211-217g15.3 |  |  | 0.913043478 | 0.695652174 | 1.322981366 |
| ptprfb |  | PTPRF | 0.912621359 | 0.514563107 | 1.087378641 |
| gnb5a |  | GNB5 | 0.912280702 | 0.596491228 | 1.052631579 |
| RDH13 (1 of many)_1 |  | RDH13 | 0.910769231 | 0.729230769 | 1.027692308 |
| myh9a |  | MYH9 | 0.910447761 | 0.630597015 | 0.97761194 |
| si:ch211-173n18.3 |  |  | 0.909090909 | 0.803030303 | 0.924242424 |
| ctsc |  | CTSC | 0.909090909 | 0.590909091 | 1.393939394 |
| fgfrl1b |  |  | 0.909090909 | 0.681818182 | 1.204545455 |
| CR855996.2 | - |  | 0.909090909 | 0.272727273 | 1.045454545 |

| tax1bp1b |  |  | 0.908847185 | 0.715817694 | 1.302949062 |
| --- | --- | --- | --- | --- | --- |
| zfr2 |  | ZFR2 | 0.907894737 | 0.539473684 | 1.184210526 |
| pik3r1 |  | PIK3R1 | 0.90776699 | 0.611650485 | 1.126213592 |
| sv2a |  | SV2A | 0.907563025 | 0.596638655 | 1.117647059 |
| mfn2 |  | MFN2 | 0.907407407 | 0.611111111 | 1.074074074 |
| nell2b |  | NELL2 | 0.907142857 | 0.585714286 | 0.95 |
| STXBP6 |  |  | 0.90625 | 0.671875 | 0.8125 |
| hnrnpua |  | HNRNPU | 0.905660377 | 0.58490566 | 0.981132075 |
| slc6a19a.1 | slc6a19a.1 | SLC6A19 | 0.905660377 | 0.358490566 | 0.943396226 |
| per1b |  | PER1 | 0.905325444 | 0.50887574 | 0.810650888 |
| gcc1 |  |  | 0.903846154 | 0.75 | 1.153846154 |
| cacng2a |  | CACNG2 | 0.903846154 | 0.538461538 | 0.980769231 |
| si:ch211-81a5.8 | si:ch211-81a5.8 |  | 0.903448276 | 0.779310345 | 2.020689655 |
| si:dkey-97a13.12 |  |  | 0.903225806 | 1.403225806 | 0.919354839 |
| si:dkey-19a16.7 | si:dkey-19a16.7 |  | 0.903225806 | 0.677419355 | 1.548387097 |
| hoxc8a |  | HOXC8 | 0.903225806 | 0.661290323 | 0.887096774 |
| higd1a | higd1a | HIGD1A | 0.902985075 | 0.820895522 | 1.962686567 |
| fam214a | fam214a | FAM214A | 0.902777778 | 0.652777778 | 1.5 |
| pir | pir | PIR | 0.902439024 | 1.097560976 | 6.585365854 |
| amph |  | AMPH | 0.901515152 | 0.666666667 | 1.212121212 |
| ankrd33aa | ankrd33aa | ANKRD33 | 0.901408451 | 0.915492958 | 1.507042254 |
| nucks1a |  |  | 0.900647948 | 0.742980562 | 1.058315335 |
| rdh12l | rdh12l | RDH12 | 0.9 | 0.8 | 1.666666667 |
| grn2 | grn2 | GRN | 0.9 | 0.666666667 | 0.4 |
| tmem251 | tmem251 | TMEM251 | 0.9 | 0.625 | 1.575 |
| ahcyl2 | ahcyl2b | AHCYL2 | 0.9 | 0.605263158 | 0.994736842 |
| hnrnph3 | hnrnph3 | HNRNPH3 | 0.9 | 0.45 | 1.2 |
| mmp14a |  | MMP14 | 0.899159664 | 0.647058824 | 1.142857143 |
| scamp5b |  |  | 0.898550725 | 0.753623188 | 1.289855072 |
| wipi2 |  | WIPI2 | 0.8984375 | 0.6484375 | 1.359375 |
| snx1a |  | SNX1 | 0.898305085 | 0.610169492 | 1.261016949 |
| st6galnac |  |  | 0.897959184 | 0.836734694 | 1.244897959 |
| cnga3a |  |  | 0.896551724 | 1.293103448 | 1.310344828 |
| si:dkeyp-94b4.1 | svild | SVIL | 0.896551724 | 0.655172414 | 0.913793103 |
| prr33 |  |  | 0.896039604 | 0.757425743 | 0.992574257 |
| qdpra | qdpra | QDPR | 0.894736842 | 0.852631579 | 1.978947368 |
| zgc:66427 | zgc:66427 | ZNRF1 | 0.894736842 | 0.526315789 | 1.122807018 |
| cntfr |  | CNTFR | 0.893617021 | 0.617021277 | 1.290780142 |
| mtss1 |  | MTSS1 | 0.892857143 | 0.625 | 1.035714286 |
| rgma |  | RGMA | 0.892857143 | 0.583333333 | 0.988095238 |
| si:dkeyp-87e3.1 | si:dkeyp-87e3.1 |  | 0.89258312 | 0.411764706 | 1.391304348 |
| fitm2 | fitm2 | FITM2 | 0.891891892 | 0.486486486 | 1.378378378 |
| zfand5a | zfand5a | ZFAND5 | 0.891156463 | 0.724489796 | 1.676870748 |
| got1 |  | GOT1 | 0.890946502 | 0.534979424 | 1.288065844 |
| nsun2 |  | NSUN2 | 0.890410959 | 0.630136986 | 1.123287671 |
| prss35 |  | PRSS35 | 0.888888889 | 0.632478632 | 1.05982906 |
| COLQ (1 of many) |  | COLQ | 0.888888889 | 0.611111111 | 1.111111111 |
| yap1 |  | YAP1 | 0.888888889 | 0.548611111 | 1.020833333 |
| krt222 |  | KRT222 | 0.888888889 | 0.611111111 | 1.111111111 |
| si:ch211-127l15.5 | tm9sf5 | TM9SF4 | 0.888888889 | 0.577777778 | 1.155555556 |
| adipor1a |  | ADIPOR1 | 0.8875 | 0.525 | 1.35 |
| dhtkd1 |  | DHTKD1 | 0.887323944 | 0.502347418 | 0.920187793 |
| rorab |  |  | 0.886956522 | 0.686956522 | 0.826086957 |
| tns2b |  |  | 0.886792453 | 0.698113208 | 1.075471698 |
| pak2a |  | PAK2 | 0.886363636 | 0.636363636 | 1.113636364 |
| slc20a1a |  | SLC20A1 | 0.884615385 | 0.717948718 | 1.102564103 |
| gmpr2 |  | GMPR2 | 0.884615385 | 0.512820513 | 0.807692308 |

| si:dkey-56m19.6 |  |  | 0.884297521 | 0.611570248 | 1.20661157 |
| --- | --- | --- | --- | --- | --- |
| si:ch211-214j24.9 | snap91b | SNAP91 | 0.883333333 | 0.516666667 | 1.094444444 |
| cpne3 |  |  | 0.882978723 | 0.680851064 | 1.095744681 |
| ppfia3 |  | PPFIA3 | 0.882352941 | 0.588235294 | 1.058823529 |
| mcamb |  | MCAM | 0.882352941 | 0.541176471 | 0.905882353 |
| cyr61 |  |  | 0.880733945 | 0.697247706 | 0.770642202 |
| abca12 |  | ABCA12 | 0.880597015 | 0.597014925 | 1.119402985 |
| si:ch211-132b12.7 |  |  | 0.879746835 | 0.965189873 | 1.164556962 |
| phkb |  |  | 0.878787879 | 0.737373737 | 1.262626263 |
| epdl1 | epdl1 | EPDR1 | 0.878172589 | 0.720812183 | 1.644670051 |
| tesk2 |  |  | 0.87804878 | 0.682926829 | 1.195121951 |
| zpr1 |  |  | 0.87755102 | 0.775510204 | 1.408163265 |
| ensaa | ensaa | ENSA | 0.876923077 | 0.415384615 | 1.230769231 |
| acsl4a |  | ACSL4 | 0.876404494 | 0.539325843 | 1.112359551 |
| dpep1 |  | DPEP1 | 0.876404494 | 0.516853933 | 1.426966292 |
| myod1 |  |  | 0.875968992 | 0.720930233 | 0.759689922 |
| tmco1 |  | TMCO1 | 0.875 | 0.64375 | 1.1625 |
| cobll1b | cobll1b | COBLL1 | 0.875 | 0.597222222 | 1.166666667 |
| muc13b |  | MUC5B | 0.873134328 | 0.597014925 | 1.358208955 |
| ogdhb | ogdhb | OGDH | 0.873015873 | 0.396825397 | 1.015873016 |
| CREB3L3 (1 of many) |  | CREB3L3 | 0.872340426 | 0.574468085 | 0.765957447 |
| slc13a2 |  |  | 0.872131148 | 0.862295082 | 1.036065574 |
| myh11a | myh11a | MYH11 | 0.871559633 | 0.47706422 | 0.706422018 |
| mark3a |  |  | 0.871428571 | 0.685714286 | 1.2 |
| bves |  |  | 0.87037037 | 0.888888889 | 1.203703704 |
| si:ch1073-392o20.2 |  | TGOLN2 | 0.87037037 | 0.574074074 | 1.37037037 |
| tmem229b | tmem229b | TMEM229B | 0.87037037 | 0.537037037 | 1.148148148 |
| myo1cb | myo1cb | MYO1C | 0.87037037 | 0.462962963 | 0.740740741 |
| si:dkey-183j2.10 |  |  | 0.87 | 0.7 | 1.24 |
| zgc:154142 | zgc:154142 |  | 0.869158879 | 0.495327103 | 0.887850467 |
| si:ch211-214p16.1 | si:ch211-214p16.1 |  | 0.868421053 | 1.328947368 | 1.723684211 |
| hspbp1 | hspbp1 | HSPBP1 | 0.868421053 | 0.473684211 | 1.763157895 |
| itpk1a |  | ITPK1 | 0.868131868 | 0.598901099 | 0.741758242 |
| slc39a10 |  | SLC39A10 | 0.867924528 | 0.547169811 | 1.094339623 |
| kat6a |  | KAT6A | 0.867924528 | 0.509433962 | 1.037735849 |
| zgc:153240 |  |  | 0.867647059 | 0.705882353 | 1.205882353 |
| cilp |  |  | 0.866666667 | 0.685714286 | 1.00952381 |
| mettl9 |  | METTL9 | 0.865384615 | 0.557692308 | 0.884615385 |
| hnrnpul1 |  | HNRNPUL1 | 0.864864865 | 0.671171171 | 0.837837838 |
| cybrd1 | cybrd1 | CYBRD1 | 0.863636364 | 1.590909091 | 1.136363636 |
| cpe |  | CPE | 0.863157895 | 0.552631579 | 1.242105263 |
| acvr1ba |  | ACVR1B | 0.863013699 | 0.575342466 | 0.98630137 |
| npdc1a |  | NPDC1 | 0.862745098 | 0.617647059 | 1.039215686 |
| baz2a |  | BAZ2A | 0.862745098 | 0.509803922 | 1 |
| tcf12 |  | TCF12 | 0.8625 | 0.575 | 1.1125 |
| tgm1l1 |  | TGM1 | 0.8625 | 0.525 | 1.0125 |
| arf1 |  | ARF1 | 0.862385321 | 0.541284404 | 1.146788991 |
| rerea | rerea | RERE | 0.860759494 | 0.493670886 | 1.025316456 |
| plcd1a |  | PLCD1 | 0.860465116 | 0.558139535 | 1.255813953 |
| entpd1 |  |  | 0.859504132 | 0.760330579 | 0.884297521 |
| and2 |  |  | 0.859429367 | 0.783576896 | 1.148921364 |
| hspb6 |  |  | 0.85786802 | 0.766497462 | 1.228426396 |
| myom1a |  | MYOM1 | 0.857541899 | 0.61452514 | 1.069832402 |
| pou3f3a |  | POU3F3 | 0.857142857 | 0.614285714 | 1.042857143 |
| brd4 | brd4 | BRD7 | 0.857142857 | 0.428571429 | 1.166666667 |
| si:cabz01007794.1 |  |  | 0.856589147 | 0.771317829 | 1.054263566 |
| aplp1 |  | APLP1 | 0.855172414 | 0.637931034 | 1.110344828 |

| si:ch211-105c13.3 |  |  | 0.85483871 | 0.85125448 | 1.155913978 |
| --- | --- | --- | --- | --- | --- |
| HACD1 |  |  | 0.85483871 | 0.698924731 | 1.23655914 |
| gabrb2 |  | GABRB2 | 0.85483871 | 0.580645161 | 1.209677419 |
| si:ch1073-83n3.2 | si:ch1073-83n3.2 |  | 0.854166667 | 0.520833333 | 1.166666667 |
| col6a1 | col6a1 | COL6A1 | 0.854166667 | 0.475 | 0.7625 |
| slc32a1 |  | SLC32A1 | 0.852941176 | 0.617647059 | 1.021008403 |
| slc16a9b | slc16a9b | SLC16A9 | 0.852941176 | 0.470588235 | 2.676470588 |
| bcl2l1 | bcl2l1 | BCL2L1 | 0.852631579 | 0.4 | 1.063157895 |
| arid1ab |  | ARID1A | 0.851851852 | 0.537037037 | 1.018518519 |
| si:ch211-188p14.5 |  |  | 0.851351351 | 0.837837838 | 0.918918919 |
| ciartb |  | CIART | 0.851351351 | 0.635135135 | 1.121621622 |
| sema3d |  |  | 0.85106383 | 0.755319149 | 0.808510638 |
| si:dkey-195m11.8 |  |  | 0.85 | 0.725 | 1.25 |
| rlbp1b |  |  | 0.846153846 | 0.929487179 | 1.121794872 |
| cd151 |  | CD151 | 0.846153846 | 0.564102564 | 1.333333333 |
| kctd17 |  | KCTD17; DCDC1 | 0.846153846 | 0.564102564 | 1.307692308 |
| gss | gss | GSS | 0.846153846 | 0.538461538 | 1.846153846 |
| ago4 | ago4 | AGO4 | 0.845238095 | 0.369047619 | 1.119047619 |
| NPNT (1 of many) |  |  | 0.844444444 | 0.8 | 1.311111111 |
| tmem182a |  | TMEM182 | 0.844036697 | 0.52293578 | 1.183486239 |
| anos1a |  | ANOS1 | 0.843283582 | 0.634328358 | 1.194029851 |
| col12a1b | col12a1b | COL12A1 | 0.843137255 | 0.470588235 | 0.823529412 |
| pwp2h |  | PWP2 | 0.842857143 | 0.557142857 | 0.685714286 |
| atg9a |  |  | 0.842105263 | 0.684210526 | 1.473684211 |
| map3k5 | map3k5 | MAP3K5 | 0.842105263 | 0.5 | 1.342105263 |
| rarga |  | RARB | 0.841269841 | 0.650793651 | 0.920634921 |
| susd6 |  | SUSD6 | 0.84057971 | 0.594202899 | 1.391304348 |
| coch |  |  | 0.84 | 0.78 | 0.82 |
| si:ch211-261n11.5 |  |  | 0.84 | 0.74 | 1 |
| adam10a |  | ADAM10 | 0.84 | 0.588571429 | 1.308571429 |
| mafa | mafa | MAF | 0.839285714 | 0.339285714 | 1.160714286 |
| acat2 |  |  | 0.838235294 | 0.867647059 | 1.338235294 |
| hoxa10b |  |  | 0.837209302 | 0.674418605 | 1.139534884 |
| ncam1b |  | NCAM1 | 0.836734694 | 0.591836735 | 1.06122449 |
| inppl1a | inppl1a | INPPL1 | 0.836734694 | 0.489795918 | 1.204081633 |
| bcl7bb |  |  | 0.836363636 | 0.727272727 | 0.963636364 |
| mpp6b |  | MPP6 | 0.836363636 | 0.581818182 | 1.018181818 |
| fah | fah | FAH | 0.83625731 | 0.994152047 | 2.228070175 |
| PDE4DIP |  | PDE4D | 0.836065574 | 0.540983607 | 1.31147541 |
| fam13a |  |  | 0.833333333 | 0.677777778 | 1.277777778 |
| pmpca | pmpca | PMPCA | 0.833333333 | 0.489583333 | 0.958333333 |
| sdccag3 |  |  | 0.833333333 | 0.857142857 | 1.19047619 |
| crabp2a |  |  | 0.833333333 | 0.680555556 | 0.805555556 |
| si:dkey-183c16.7 | acot18 | ACOT8 | 0.833333333 | 0.5 | 1.238095238 |
| cdc42ep4b |  | CDC42EP4 | 0.830769231 | 0.630769231 | 0.923076923 |
| strn3 |  | STRN3 | 0.830188679 | 0.622641509 | 1.047169811 |
| si:dkeyp-57d7.4 | si:dkeyp-57d7.4 |  | 0.829787234 | 0.531914894 | 1.372340426 |
| bcor | bcor | BCOR | 0.829787234 | 0.468085106 | 1.042553191 |
| mfi2 | meltf | MELTF | 0.829787234 | 0.361702128 | 0.446808511 |
| cyp7a1 |  | CYP7A1 | 0.829268293 | 0.653658537 | 0.824390244 |
| slc23a1 |  | SLC23A1 | 0.828571429 | 0.628571429 | 1.371428571 |
| tob1a |  | TOB1 | 0.827731092 | 0.658263305 | 1.113445378 |
| abca1a | abca1a | ABCA1 | 0.827586207 | 0.448275862 | 1.25862069 |
| trim3b |  | TRIM3 | 0.827160494 | 0.580246914 | 1.061728395 |
| sp4 |  | SP4 | 0.826923077 | 0.596153846 | 1.019230769 |
| cipcb |  | CIPC | 0.826086957 | 0.652173913 | 1.108695652 |
| pnp4a |  | PNP | 0.824427481 | 0.511450382 | 0.885496183 |

| agxt2 |  | AGXT2 | 0.824175824 | 0.725274725 | 0.659340659 |
| --- | --- | --- | --- | --- | --- |
| msx3 |  | MSX2 | 0.823529412 | 0.529411765 | 1.215686275 |
| si:ch211-161h7.8 | si:ch211-161h7.8 |  | 0.823529412 | 0.647058824 | 1.470588235 |
| stk10 |  | STK10 | 0.823529412 | 0.514705882 | 1.132352941 |
| zgc:136870 | zgc:136870 | GIMAP4 | 0.823529412 | 0.5 | 1.617647059 |
| TST |  |  | 0.822580645 | 0.741935484 | 1.161290323 |
| ppp3cb | ppp3cb | PPP3CA | 0.821917808 | 0.48630137 | 1.171232877 |
| zgc:194887 | zgc:194887 |  | 0.821917808 | 0.356164384 | 1.068493151 |
| foxd3 |  | FOXD3 | 0.821052632 | 0.515789474 | 1.105263158 |
| aqp3a |  | AQP3 | 0.820592824 | 0.5975039 | 0.848673947 |
| camk1gb |  | CAMK1G | 0.820512821 | 0.666666667 | 1.333333333 |
| spryd7b | spryd7b | SPRYD7 | 0.820512821 | 0.58974359 | 1.512820513 |
| apc |  | APC | 0.819047619 | 0.504761905 | 1.019047619 |
| gnb5b |  |  | 0.81865285 | 0.787564767 | 1.119170984 |
| cry1bb | cry3b | CRY2 | 0.818181818 | 1.090909091 | 1.090909091 |
| cldn15lb |  | CLDN15 | 0.818181818 | 0.590909091 | 1.136363636 |
| stard7 |  | STARD7 | 0.818181818 | 0.545454545 | 1.056818182 |
| cx28.9 | cx28.9 | GJB7 | 0.817567568 | 0.513513514 | 1.385135135 |
| dusp4 |  | DUSP4 | 0.816666667 | 0.55 | 1.083333333 |
| impdh1a |  |  | 0.816326531 | 0.693877551 | 1.163265306 |
| thbs1b |  | THBS1 | 0.816 | 0.568 | 0.876 |
| dspa |  | DSP | 0.816 | 0.504 | 0.904 |
| egr1 |  |  | 0.815436242 | 0.677852349 | 1.104026846 |
| rtn2b | rtn2b | RTN2 | 0.81372549 | 0.431372549 | 0.960784314 |
| parp3 |  | PARP3 | 0.813559322 | 0.542372881 | 0.966101695 |
| maff | maff | MAFF | 0.812865497 | 0.631578947 | 2.192982456 |
| cx32.3 |  |  | 0.81264637 | 0.695550351 | 1.117096019 |
| creb3l2 |  | CREB3L2 | 0.8125 | 0.583333333 | 1.208333333 |
| rrm2_1 |  |  | 0.811594203 | 1.101449275 | 0.884057971 |
| rhcgl1 |  | RHCG | 0.811447811 | 0.646464646 | 0.828282828 |
| kcnip3a |  |  | 0.810526316 | 0.673684211 | 0.894736842 |
| klf8 |  | KLF8 | 0.80952381 | 0.547619048 | 1.023809524 |
| tcp11l2 |  | TCP11L2 | 0.809322034 | 0.605932203 | 1.36440678 |
| kif1b | kif1b | KIF1B | 0.808823529 | 0.426470588 | 1.073529412 |
| shisa7b | shisa7b | SHISA7 | 0.808510638 | 0.404255319 | 1.276595745 |
| tfap4 | tfap4 | TFAP4 | 0.808510638 | 0.404255319 | 0.893617021 |
| klhl43 | klhl43 | KLHL42 | 0.808290155 | 0.497409326 | 0.792746114 |
| suclg2 |  | SUCLG2 | 0.808270677 | 0.518796992 | 1.09962406 |
| elavl3 |  | ELAVL3 | 0.807829181 | 0.505338078 | 1.09252669 |
| mtmr7b |  |  | 0.807692308 | 0.730769231 | 1.096153846 |
| fabp11b | fabp4b | FABP5 | 0.807692308 | 0.384615385 | 0.961538462 |
| chl1b |  | CHL1 | 0.807017544 | 0.561403509 | 1.070175439 |
| eomesa |  |  | 0.806451613 | 0.870967742 | 0.951612903 |
| mzt2b |  | MZT2B | 0.806451613 | 0.580645161 | 1.35483871 |
| dpysl5b |  | DPYSL5 | 0.805970149 | 0.574626866 | 1 |
| pcdh17 |  | PCDH17 | 0.805825243 | 0.601941748 | 1.009708738 |
| bcl7ba |  |  | 0.805555556 | 0.944444444 | 1.055555556 |
| si:dkey-111e8.1 |  | RNF214 | 0.804347826 | 0.52173913 | 1.282608696 |
| alg3 | alg3 | ALG3 | 0.804347826 | 0.434782609 | 1.108695652 |
| tnfrsf1a | tnfrsf1a | TNFRSF1A | 0.804347826 | 0.391304348 | 1.413043478 |
| CABZ01009157.1 | - |  | 0.803921569 | 0.490196078 | 1.019607843 |
| lamp2 |  | LAMP2 | 0.803680982 | 0.607361963 | 1.349693252 |
| xiap |  | XIAP | 0.803278689 | 0.519125683 | 1.284153005 |
| calua | calua | CALU | 0.801136364 | 0.477272727 | 0.965909091 |
| clpxb |  |  | 0.8 | 0.775 | 0.975 |
| cx35.4 |  |  | 0.8 | 0.68 | 0.8 |
| ulk2 |  | ULK2 | 0.8 | 0.651612903 | 1.341935484 |

| ef1 | elf1 | ELF1 | 0.8 | 0.618181818 | 1.090909091 |
| --- | --- | --- | --- | --- | --- |
| cyfip2 |  | CYFIP2 | 0.8 | 0.523809524 | 1.114285714 |
| atf5b |  | ATF5 | 0.8 | 0.522222222 | 1.477777778 |
| cnot6a | cnot6a | CNOT6 | 0.8 | 0.4 | 0.9125 |
| slc7a3a | slc7a3a | SLC7A3 | 0.8 | 0.381818182 | 1.218181818 |
| tango2 |  | TANGO2 | 0.8 | 0.583333333 | 1.1 |
| atp1b4 |  | ATP1B4 | 0.8 | 0.53968254 | 1.136507937 |
| cnn2 |  | CNN2 | 0.797794118 | 0.610294118 | 1.040441176 |
| ak3 | ak3 | AK3 | 0.797297297 | 0.432432432 | 0.959459459 |
| emd | emd | EMD | 0.795918367 | 0.448979592 | 1.12244898 |
| stk40 | stk40 | STK40 | 0.795454545 | 0.454545455 | 0.954545455 |
| prr15la |  | PRR15L | 0.794871795 | 0.512820513 | 1.384615385 |
| gpa33 | gpa33b | GPA33 | 0.794871795 | 0.153846154 | 1.102564103 |
| camkk1b |  |  | 0.794520548 | 0.780821918 | 1.465753425 |
| bscl2 | bscl2 | BSCL2 | 0.794117647 | 0.352941176 | 1.411764706 |
| stk38l |  |  | 0.793103448 | 0.706896552 | 1.034482759 |
| mtr |  | MTR | 0.79245283 | 0.528301887 | 0.830188679 |
| si:ch73-185c24.2 | si:ch73-185c24.2 | LRRC18 | 0.79245283 | 0.471698113 | 0.830188679 |
| slc4a10a | slc4a10a | SLC4A10 | 0.79245283 | 0.462264151 | 1.188679245 |
| CABZ01067153.1 | - |  | 0.791666667 | 0.333333333 | 1.020833333 |
| crfb6 | ifngr2 | IFNGR1 | 0.791044776 | 0.567164179 | 1.253731343 |
| zgc:162144 |  |  | 0.790697674 | 1.093023256 | 1.162790698 |
| fscn1b |  | FSCN1 | 0.790697674 | 0.581395349 | 1.209302326 |
| txnipb |  | TXNIP | 0.79047619 | 0.861904762 | 0.657142857 |
| zgc:92161 |  |  | 0.790322581 | 0.774193548 | 1.096774194 |
| tal2 |  | TAL2 | 0.789473684 | 0.614035088 | 1.192982456 |
| aldh4a1 | aldh4a1 | ALDH4A1 | 0.789473684 | 0.49122807 | 0.815789474 |
| si:ch211-214p16.3 |  |  | 0.7890625 | 0.890625 | 0.8359375 |
| ptenb | ptenb | PTEN | 0.788888889 | 0.477777778 | 0.988888889 |
| ache |  | ACHE | 0.788732394 | 0.549295775 | 1.117370892 |
| ccl34b.1 |  |  | 0.788461538 | 0.865384615 | 1.076923077 |
| fbn2b_1 | - | FBN3 | 0.788461538 | 0.384615385 | 0.807692308 |
| pprc1 |  | PPRC1 | 0.788235294 | 0.611764706 | 1.023529412 |
| march7_1 |  | MARCH7 | 0.788235294 | 0.576470588 | 0.858823529 |
| sec23b |  | SEC23B | 0.787709497 | 0.592178771 | 0.865921788 |
| midn |  | MIDN | 0.787096774 | 0.503225806 | 1.077419355 |
| zgc:112320 | pde6ga | PDE6G | 0.786666667 | 0.626666667 | 2.84 |
| opn9 | opn9 | OPN5 | 0.785714286 | 0.5 | 1.285714286 |
| rad21b | rad21b | RAD21 | 0.785714286 | 0.428571429 | 1.125 |
| si:ch211-198a12.6 | si:ch211-198a12.6 | ZNF526 | 0.785714286 | 0.552380952 | 1.061904762 |
| c3b.2 |  | C3 | 0.784313725 | 0.62745098 | 0.960784314 |
| slc7a2 |  | SLC7A2 | 0.784313725 | 0.509803922 | 1.019607843 |
| sulf2b |  | SULF2 | 0.784313725 | 0.568627451 | 1.058823529 |
| b4galt1l |  | B4GALT1 | 0.783783784 | 0.513513514 | 1.337837838 |
| aftpha |  | AFTPH | 0.783333333 | 0.55 | 1.05 |
| gna13b |  | GNA13 | 0.782608696 | 0.586956522 | 1.282608696 |
| pld1a |  | PLD1 | 0.782608696 | 0.543478261 | 1.260869565 |
| arhgef1b |  | ARHGEF1 | 0.782051282 | 0.564102564 | 1.217948718 |
| si:ch73-269m14.2 | si:ch73-269m14.2 | C5orf15 | 0.782051282 | 0.448717949 | 0.91025641 |
| enpp7.1 |  | ENPP7 | 0.781637717 | 0.915632754 | 0.535980149 |
| sh2b1 |  | SH2B1 | 0.780821918 | 0.534246575 | 1.054794521 |
| si:dkey-91i10.3 | si:dkey-91i10.3 | CYP27A1 | 0.78 | 0.56 | 0.52 |
| itpk1b | itpk1b | ITPK1 | 0.78 | 0.48 | 1.08 |
| nrap |  | NRAP | 0.777777778 | 0.604938272 | 1.172839506 |
| me2 |  | ME2 | 0.777777778 | 0.521367521 | 0.948717949 |
| dyrk2 | dyrk2 | DYRK2 | 0.777777778 | 0.5 | 0.981481481 |
| dapk3 | dapk3 | DAPK3 | 0.777777778 | 0.444444444 | 0.98989899 |

| atp2b2 | atp2b2 | ATP2B2 | 0.777777778 | 0.407407407 | 1.024691358 |
| --- | --- | --- | --- | --- | --- |
| spry4 |  | SPRY4 | 0.777777778 | 0.611111111 | 1 |
| zgc:158291 |  | POU5F1B | 0.776859504 | 0.58677686 | 1.099173554 |
| itpkcb | itpkcb | ITPKC | 0.775862069 | 0.482758621 | 1.189655172 |
| abcc4 | abcc4 | ABCC4 | 0.775862069 | 0.465517241 | 1.517241379 |
| amotl2a |  | AMOTL2 | 0.775510204 | 0.56462585 | 0.965986395 |
| rgra |  |  | 0.775280899 | 0.831460674 | 0.775280899 |
| si:ch73-44m9.5 | si:ch73-44m9.5 |  | 0.775147929 | 0.597633136 | 0.934911243 |
| smpd1 |  |  | 0.771428571 | 0.771428571 | 0.971428571 |
| s100z | s100z | S100Z | 0.770833333 | 1.25 | 2.1875 |
| myo1b | myo1b | MYO1B | 0.77 | 0.42 | 0.83 |
| prkab1a | prkab1a | PRKAB1 | 0.769230769 | 0.461538462 | 1.051282051 |
| kdm2ba | kdm2ba | KDM2B | 0.769230769 | 0.423076923 | 0.961538462 |
| cpt1ab |  | CPT1A | 0.768656716 | 0.544776119 | 1.208955224 |
| dbpb | dbpb | DBP | 0.768421053 | 0.526315789 | 1.726315789 |
| gabrb3 |  | GABRB3 | 0.767123288 | 0.506849315 | 0.97260274 |
| tspan7b |  | TSPAN7 | 0.767068273 | 0.526104418 | 1.265060241 |
| DNAJB5 (1 of many) |  |  | 0.766666667 | 0.813333333 | 1.166666667 |
| chrne |  | CHRNE | 0.766666667 | 0.555555556 | 1.033333333 |
| snx5 |  | SNX5 | 0.764044944 | 0.595505618 | 1.033707865 |
| tspan33a | tspan33a | TSPAN33 | 0.763157895 | 0.657894737 | 1.5 |
| st6gal2a |  | ST6GAL2 | 0.762711864 | 0.508474576 | 1.101694915 |
| tyr |  | TYR | 0.762711864 | 0.508474576 | 0.86440678 |
| nr1d1 |  | NR1D1 | 0.762035763 | 0.775790922 | 1.398899587 |
| crtac1a |  |  | 0.761904762 | 1.244444444 | 1.279365079 |
| AC024175.14 | mt-tm | MT-TM | 0.761904762 | 0.494047619 | 1.107142857 |
| si:ch73-335l21.1 |  |  | 0.76 | 0.84 | 1.1 |
| tmem183a | tmem183a | TMEM183A | 0.76 | 0.6 | 1.16 |
| kdm6ba | kdm6ba | KDM6B | 0.759259259 | 0.462962963 | 1.092592593 |
| rcvrn3 |  | RCVRN | 0.759067358 | 0.549222798 | 0.955958549 |
| desi1a | desi1a | DESI1 | 0.75862069 | 0.405172414 | 1.086206897 |
| hsp70.1 | hsp70.1 | HSPA1L | 0.757575758 | 0.681818182 | 1.893939394 |
| myoz2b |  | MYOZ2 | 0.757281553 | 0.54368932 | 0.941747573 |
| zgc:175280 | zgc:175280 | SLC7A13 | 0.756944444 | 0.472222222 | 1.006944444 |
| lmbrd1 |  | LMBRD1 | 0.756756757 | 0.662162162 | 1.243243243 |
| si:ch211-214p13.3 |  |  | 0.756097561 | 0.853658537 | 1.170731707 |
| pspc1 |  | PSPC1 | 0.755555556 | 0.566666667 | 0.955555556 |
| pkp3a |  | PKP3 | 0.755434783 | 0.565217391 | 0.826086957 |
| tcf3b | tcf3b | TCF3 | 0.754901961 | 0.441176471 | 1 |
| p4ha2 | p4ha2 | P4HA2 | 0.754385965 | 0.719298246 | 1.543859649 |
| synpo2b |  | SYNPO2 | 0.754385965 | 0.50877193 | 0.859649123 |
| syn1 | syn1 | SYN1 | 0.753846154 | 0.292307692 | 0.907692308 |
| gda |  | GDA | 0.751677852 | 0.610738255 | 0.644295302 |
| bms1 | bms1 | BMS1 | 0.751445087 | 0.445086705 | 0.843930636 |
| pnpla6 | pnpla6 | PNPLA6 | 0.75 | 0.482142857 | 0.910714286 |
| pou4f2 | pou4f2 | POU4F2 | 0.75 | 0.402173913 | 1.108695652 |
| larsa_1 | lars1a | LARS1 | 0.75 | 0.625 | 0.854166667 |
| abcb8 |  | ABCB8 | 0.75 | 0.566666667 | 0.916666667 |
| nr2f2 | nr2f2 | NR2F2 | 0.75 | 0.366666667 | 0.9 |
| MFAP4 (1 of  many)_12mfap4 | - | MFAP2 | 0.75 | 1.25 | 2.25 |
| anxa11b | anxa11b | ANXA11 | 0.748538012 | 0.444444444 | 1.040935673 |
| rh50 | rh50 |  | 0.746031746 | 0.714285714 | 0.301587302 |
| pik3r3a | pik3r3a | PIK3R3 | 0.746031746 | 0.46031746 | 1.603174603 |
| myt1a | myt1a | MYT1 | 0.744897959 | 0.336734694 | 1.102040816 |
| ido1 |  | IDO2 | 0.742857143 | 0.580952381 | 0.628571429 |
| ctsf |  | CTSF | 0.741721854 | 0.593818985 | 1.25607064 |

| sgcb | sgcb | SGCB | 0.741176471 | 0.294117647 | 0.870588235 |
| --- | --- | --- | --- | --- | --- |
| irg1l | irg1l | ACOD1 | 0.740740741 | 0.481481481 | 1.740740741 |
| dpysl4 | dpysl4 | DPYSL4 | 0.740740741 | 0.481481481 | 0.851851852 |
| si:dkeyp-69b9.6 |  | ZNF865 | 0.739130435 | 0.52173913 | 1.217391304 |
| sdf4 |  | SDF4 | 0.738562092 | 0.60130719 | 1.130718954 |
| mipep | mipep | MIPEP | 0.738095238 | 0.404761905 | 1.071428571 |
| SRL (1 of many) | - | SRL | 0.737373737 | 0.353535354 | 0.888888889 |
| slc1a2a |  |  | 0.736842105 | 0.701754386 | 1.438596491 |
| ppm1nb | ppm1nb | PPM1N | 0.736263736 | 0.43956044 | 1.076923077 |
| lipg |  | LIPG | 0.735849057 | 0.566037736 | 1.075471698 |
| prkaa1 | prkaa1 | PRKAA1 | 0.735849057 | 0.471698113 | 1.150943396 |
| sqstm1 | sqstm1 | SQSTM1 | 0.735714286 | 0.546428571 | 2.267857143 |
| lct | lct | LCT | 0.734693878 | 0.387755102 | 1.040816327 |
| si:dkey-38p12.3 | si:dkey-38p12.3 | ARHGEF15 | 0.734375 | 0.5 | 0.96875 |
| osbpl2b | osbpl2b | OSBPL2 | 0.734375 | 0.453125 | 0.828125 |
| prph2a |  | PRPH2 | 0.734042553 | 0.521276596 | 1.14893617 |
| kmo |  | KMO | 0.733333333 | 0.65 | 1.15 |
| sez6l2 | sez6l2 | SEZ6L2 | 0.733333333 | 0.433333333 | 1.016666667 |
| scn1bb |  | SCN1B | 0.731958763 | 0.649484536 | 0.93814433 |
| angptl3 |  | ANGPTL3 | 0.731707317 | 0.707317073 | 0.658536585 |
| gpat3 | gpat3 | GPAT4 | 0.731707317 | 0.487804878 | 1.109756098 |
| CPLX1 (1 of many) |  | CPLX1 | 0.731563422 | 0.536873156 | 0.935103245 |
| arl3l2_1 |  |  | 0.731481481 | 0.851851852 | 1.425925926 |
| taf6_2 |  | TAF6L | 0.731343284 | 0.552238806 | 0.955223881 |
| ptrfb | cavin1b | CAVIN1 | 0.730769231 | 0.41025641 | 0.653846154 |
| hcls1 |  | HCLS1 | 0.730337079 | 0.573033708 | 1.011235955 |
| camk2d1 | camk2d1 | CAMK2D | 0.729166667 | 0.333333333 | 0.875 |
| ptprn2 |  | PTPRN2 | 0.72815534 | 0.621359223 | 1.029126214 |
| sst6 | sst6 | SST | 0.727272727 | 1.681818182 | 2.136363636 |
| praf2 |  | PRAF2 | 0.727272727 | 0.654545455 | 1.109090909 |
| gbgt1l4 |  | GBGT1 | 0.727272727 | 0.6 | 1.054545455 |
| ppp3ca |  | PPP3CA | 0.726495726 | 0.512820513 | 1.256410256 |
| sox2 | sox2 | SOX2 | 0.725888325 | 0.456852792 | 1.223350254 |
| celf3a | celf3a | CELF3 | 0.724832215 | 0.355704698 | 1.093959732 |
| prox1a | prox1a | PROX1 | 0.724637681 | 0.449275362 | 0.724637681 |
| larp1b | larp1b | LARP1B | 0.722972973 | 0.378378378 | 1.128378378 |
| ifitm1 |  |  | 0.722222222 | 0.798611111 | 0.888888889 |
| slc29a1a | slc29a1a | SLC29A1 | 0.721518987 | 0.481012658 | 1.050632911 |
| syncripl | syncripl | SYNCRIP | 0.721311475 | 0.43442623 | 1 |
| grin1a | grin1a | GRIN1 | 0.720779221 | 0.474025974 | 0.980519481 |
| errfi1 | errfi1a | ERRFI1 | 0.71875 | 0.4375 | 1.296875 |
| cbx8b |  | CBX8 | 0.716981132 | 0.641509434 | 1.226415094 |
| tmprss4a | tmprss4a | TMPRSS4 | 0.714285714 | 0.714285714 | 1.642857143 |
| gadd45aa | gadd45aa | GADD45A | 0.714285714 | 0.607142857 | 2.303571429 |
| hhip |  | HHIP | 0.714285714 | 0.542857143 | 0.771428571 |
| atxn7l3 |  | ATXN7L3 | 0.714285714 | 0.510204082 | 0.93877551 |
| phf2 | phf2 | PHF2 | 0.714285714 | 0.387755102 | 1.285714286 |
| apoa4b.3 | apoa4b.3 | APOA4 | 0.714046823 | 0.446488294 | 0.384615385 |
| mapk3 | mapk3 | MAPK3 | 0.712707182 | 0.392265193 | 1.033149171 |
| mt-nd4l |  | ND4 | 0.710073069 | 0.508872651 | 0.956419624 |
| kdm2ab |  | KDM2A | 0.708333333 | 0.520833333 | 1.208333333 |
| zyx | zyx | ZYX | 0.708333333 | 0.458333333 | 1.229166667 |
| lamc1 | lamc1 | LAMC1 | 0.708333333 | 0.380208333 | 0.942708333 |
| ciarta |  | CIART | 0.70783848 | 0.570071259 | 1.220902613 |
| pla2g6 | pla2g6 | PLA2G6 | 0.707317073 | 0.43902439 | 1.219512195 |
| si:ch211-133j6.3 | krt18a.2 | KRT18 | 0.706896552 | 0.620689655 | 0.672413793 |
| trpv6 |  |  | 0.705882353 | 0.941176471 | 1.333333333 |

| si:ch211-125o16.4 | si:ch211-125o16.4 | AHNAK2 | 0.704918033 | 0.614754098 | 1.032786885 |
| --- | --- | --- | --- | --- | --- |
| adrbk2 | grk3 | GRK3 | 0.704545455 | 0.363636364 | 0.954545455 |
| idh3g |  | IDH3G | 0.703703704 | 0.524691358 | 0.969135802 |
| desi1b | desi1b | DESI1 | 0.703703704 | 0.481481481 | 2.259259259 |
| glulb |  | GLUL | 0.703367876 | 0.637305699 | 1.161917098 |
| rab14l |  | RAB14 | 0.703296703 | 0.593406593 | 0.978021978 |
| sh3gl1b |  | SH3GL1 | 0.70212766 | 0.595744681 | 0.765957447 |
| si:ch211-195b13.1 | si:ch211-195b13.1 |  | 0.702020202 | 0.646464646 | 0.878787879 |
| nck1a |  | NCK1 | 0.701298701 | 0.532467532 | 0.961038961 |
| fam210ab | fam210ab | FAM210A | 0.701298701 | 0.38961039 | 1.012987013 |
| zgc:123068 |  |  | 0.7 | 1.05 | 1.05 |
| cyp27a7 |  | CYP27A1 | 0.698795181 | 0.662650602 | 0.903614458 |
| tmem263 | tmem263 | TMEM263 | 0.698630137 | 0.465753425 | 0.945205479 |
| gna13a |  | GNA13 | 0.698113208 | 0.528301887 | 1.169811321 |
| si:ch211-214j8.15 |  |  | 0.695652174 | 0.543478261 | 1 |
| add2 |  | ADD2 | 0.695652174 | 0.52173913 | 1.065217391 |
| ppp6r2a | ppp6r2a | PPP6R2 | 0.695652174 | 0.391304348 | 1.043478261 |
| ankrd1b |  | ANKRD1 | 0.695571956 | 0.540590406 | 0.998154982 |
| abcb5 | abcb5 | ABCB6 | 0.695473251 | 0.502057613 | 0.633744856 |
| si:ch211-198m17.1 | si:ch211-198m17.1 |  | 0.694444444 | 0.347222222 | 0.958333333 |
| si:dkey-95h12.1 | si:dkey-95h12.1 |  | 0.693548387 | 0.435483871 | 0.919354839 |
| jph1b | jph1b | JPH1 | 0.692622951 | 0.5 | 0.93852459 |
| cyp3c3 | cyp3c3 | CYP3A43 | 0.691542289 | 0.492537313 | 1.074626866 |
| si:ch1073-398f15.1 |  |  | 0.690909091 | 0.727272727 | 0.890909091 |
| pcf11 |  | PCF11 | 0.690721649 | 0.505154639 | 1.072164948 |
| ppm1k | ppm1k | PPM1K | 0.689655172 | 0.431034483 | 0.965517241 |
| sox10 |  | SOX10 | 0.688888889 | 0.511111111 | 1.133333333 |
| lrp12 | lrp12 | LRP12 | 0.68852459 | 0.442622951 | 1.147540984 |
| fam46ba | tent5ba | TENT5B | 0.688172043 | 0.569892473 | 0.989247312 |
| cry2 |  | CRY2 | 0.688118812 | 0.52970297 | 0.935643564 |
| oxr1a |  | OXR1 | 0.686046512 | 0.558139535 | 1.034883721 |
| reck | reck | RECK | 0.685714286 | 0.447619048 | 0.952380952 |
| myzap |  | MYZAP | 0.685185185 | 0.574074074 | 1.074074074 |
| atp2b3b | atp2b3b | ATP13A4 | 0.684210526 | 0.438596491 | 1.052631579 |
| pnp6 |  | PNP | 0.683544304 | 0.582278481 | 1.050632911 |
| zgc:91909_2 | - | RAB7A | 0.683333333 | 0.466666667 | 1.416666667 |
| ahcyl1 | ahcyl1 | AHCYL1 | 0.682926829 | 0.487804878 | 0.983739837 |
| ube2c_2 | - | UBE2C | 0.682352941 | 0.411764706 | 0.752941176 |
| CASKIN1 (1 of many) | - | CASKIN1 | 0.681818182 | 0.424242424 | 0.954545455 |
| gmpr | gmpr | GMPR | 0.681818182 | 0.481818182 | 0.927272727 |
| col11a1b | col11a1b | COL11A1 | 0.68115942 | 0.362318841 | 0.884057971 |
| si:ch1073-406l10.2 | si:ch1073-406l10.2 |  | 0.681034483 | 0.551724138 | 1.025862069 |
| nccrp1 |  |  | 0.680851064 | 0.70212766 | 1.276595745 |
| dimt1l |  | DIMT1 | 0.680851064 | 0.510638298 | 0.893617021 |
| mbnl2 | mbnl2 | MBNL2 | 0.68 | 0.48 | 1.226666667 |
| GK3P | - | GK3P | 0.68 | 0.36 | 0.8 |
| phrf1 |  | PHRF1 | 0.679245283 | 0.509433962 | 1.018867925 |
| sept3 | sept3 | SEPTIN3 | 0.679104478 | 0.492537313 | 1.052238806 |
| pgm2 | pgm2 | PGM2 | 0.677966102 | 0.406779661 | 0.796610169 |
| cobl | cobl | COBLL1 | 0.677419355 | 0.451612903 | 1.112903226 |
| tnpo2 | tnpo2 | TNPO2 | 0.677419355 | 0.35483871 | 1.032258065 |
| stc1l |  |  | 0.675324675 | 0.675324675 | 1.116883117 |
| rgmb | rgmb | RGMB | 0.675 | 0.45 | 1.2 |
| pof1b |  |  | 0.674698795 | 0.78313253 | 0.927710843 |
| cthrc1b | cthrc1b | CTHRC1 | 0.673469388 | 0.367346939 | 0.693877551 |
| si:dkey-261h17.1 | si:dkey-261h17.1 | CD34 | 0.673076923 | 0.371794872 | 0.717948718 |
| sfrp1a | sfrp1a | SFRP1 | 0.671641791 | 0.343283582 | 0.582089552 |

| ezrb | ezrb | EZR | 0.669683258 | 0.411764706 | 0.832579186 |
| --- | --- | --- | --- | --- | --- |
| akap12b | akap12b | AKAP12 | 0.666666667 | 0.41954023 | 1 |
| si:dkey-52d15.1p | si:dkey-52d15.1p |  | 0.666666667 | 0.379310345 | 0.724137931 |
| prkcdb | prkcdb | PRKCD | 0.666666667 | 0.375 | 1.083333333 |
| fam169ab | fam169ab | FAM169A | 0.666666667 | 0.354166667 | 0.916666667 |
| GPT (1 of many) |  | GPT2 | 0.666666667 | 0.833333333 | 1.333333333 |
| tnnt2c | tnnt2c | TNNT2 | 0.666666667 | 0.430555556 | 1.041666667 |
| smurf2 | smurf2 | SMURF2 | 0.666666667 | 0.428571429 | 1.095238095 |
| slc39a13 | slc39a13 | SLC39A13 | 0.666666667 | 0.404761905 | 1.095238095 |
| kcnj11 | kcnj11 | KCNJ11 | 0.666666667 | 0.288888889 | 0.955555556 |
| chka | chka | CHKA | 0.662650602 | 0.469879518 | 1.120481928 |
| srsf10a | srsf10a | SRSF1 | 0.662650602 | 0.409638554 | 0.951807229 |
| sphk2 | sphk2 | SPHK2 | 0.660714286 | 0.339285714 | 0.821428571 |
| znf1035 | znf1035 | ZNF668 | 0.660377358 | 0.433962264 | 0.905660377 |
| cds2 | cds2 | CDS2 | 0.660377358 | 0.424528302 | 0.905660377 |
| mep1b |  | MEP1B | 0.659090909 | 1.25 | 0.954545455 |
| cabp5a |  | CABP5 | 0.659090909 | 0.636363636 | 1.272727273 |
| dsg2.1 | dsg2.1 | DSG2 | 0.658227848 | 0.46835443 | 1.050632911 |
| slc2a2 | slc2a2 | SLC2A2 | 0.657142857 | 0.457142857 | 1.071428571 |
| slc38a5a | slc38a5a | SLC38A5 | 0.657142857 | 0.428571429 | 1.542857143 |
| tp53inp1 | tp53inp1 | TP53INP1 | 0.656862745 | 0.470588235 | 1.029411765 |
| add3a | add3a | ADD3 | 0.654761905 | 0.357142857 | 0.880952381 |
| skib | skib | SKI | 0.654545455 | 0.418181818 | 0.963636364 |
| dachd | dachd | DACH1 | 0.654320988 | 0.444444444 | 0.901234568 |
| tsg101a | tsg101a | TSG101 | 0.651785714 | 0.366071429 | 1.053571429 |
| pou3f1 | pou3f1 | POU3F1 | 0.65 | 0.457142857 | 0.985714286 |
| fubp3 | fubp3 | FUBP3 | 0.649006623 | 0.284768212 | 0.874172185 |
| npnt | npnta | NPNT | 0.647058824 | 0.431372549 | 0.901960784 |
| sv2bb | sv2bb | SV2B | 0.646464646 | 0.353535354 | 1.04040404 |
| ptn | ptn | PTN | 0.646258503 | 0.442176871 | 1.142857143 |
| atp1b1b | atp1b1b | ATP1B1 | 0.646017699 | 0.466814159 | 0.911504425 |
| usp21 |  | USP21 | 0.645833333 | 0.583333333 | 1.104166667 |
| taok3b | taok3b | TAOK3 | 0.645833333 | 0.5 | 0.833333333 |
| ptbp1b | ptbp1b | PTBP1 | 0.643835616 | 0.479452055 | 0.794520548 |
| spock2 | spock2 | SPOCK2 | 0.643835616 | 0.424657534 | 0.780821918 |
| tacc1 | tacc1 | TACC1 | 0.642857143 | 0.4 | 0.971428571 |
| mn1b | mn1b | MN1 | 0.642857143 | 0.333333333 | 0.642857143 |
| si:ch211-196f2.3 | si:ch211-196f2.3 | MUC5B | 0.641791045 | 0.47761194 | 1.208955224 |
| dsc2l | dsc2l | DSC | 0.641791045 | 0.358208955 | 1.194029851 |
| SSBP4 (1 of many) | - | SSBP4 | 0.641791045 | 0.343283582 | 1 |
| perp | perp | PERP | 0.640378549 | 0.416403785 | 1.113564669 |
| zgc:195001 | zgc:195001 | CD86 | 0.639037433 | 0.417112299 | 0.890374332 |
| nat15 |  | NAA60 | 0.638297872 | 0.553191489 | 1.340425532 |
| slc28a1 | slc28a1 | SLC28A1 | 0.6375 | 0.2625 | 1.3875 |
| zgc:174917 | zgc:174917 |  | 0.636363636 | 0.181818182 | 3 |
| CU929052.1 | - |  | 0.635294118 | 0.364705882 | 0.670588235 |
| zgc:100868 | zgc:100868 | PRSS33 | 0.635036496 | 0.270072993 | 2.240875912 |
| slc25a10 | slc25a10 | SLC25A10 | 0.634920635 | 0.444444444 | 0.785714286 |
| itgb4 | itgb4 | ITGB4 | 0.634920635 | 0.412698413 | 0.952380952 |
| cd99 |  | CD99 | 0.634408602 | 0.537634409 | 1.053763441 |
| bmp3 | bmp3 | BMP3 | 0.631578947 | 0.368421053 | 0.754385965 |
| sv2ba | sv2ba | SV2B | 0.631578947 | 0.355263158 | 1.078947368 |
| bet1 | bet1 | BET1 | 0.631578947 | 0.302631579 | 0.894736842 |
| znf185 | znf185 | ZNF185 | 0.630136986 | 0.520547945 | 0.867579909 |
| si:ch1073-44g3.1 | si:ch1073-44g3.1 | C5orf24 | 0.630136986 | 0.397260274 | 1.178082192 |
| AC024175.21 |  |  | 0.62962963 | 0.925925926 | 0.592592593 |
| arih1 | arih1 | ARIH1 | 0.62962963 | 0.444444444 | 1.111111111 |

| cdca4 | cdca4 | CDCA4 | 0.628865979 | 0.402061856 | 1.257731959 |
| --- | --- | --- | --- | --- | --- |
| nanos1 | nanos1 | NANOS1 | 0.62633452 | 0.444839858 | 0.857651246 |
| matn3b |  | MATN3 | 0.625 | 0.678571429 | 0.803571429 |
| cxcl19 | cxcl19 | CXCL9 | 0.625 | 0.625 | 2.25 |
| ctss2.1 | ctss2.1 | CTSS | 0.625 | 0.59375 | 2.09375 |
| efna1a | efna1a | EFNA1 | 0.625 | 0.453125 | 0.78125 |
| hprt1 | hprt1 | HPRT1 | 0.623655914 | 0.462365591 | 1.268817204 |
| dagla | dagla | DAGLA | 0.622641509 | 0.396226415 | 1.264150943 |
| micu3b | micu3b | MICU3 | 0.622222222 | 0.422222222 | 1.2 |
| rbp7a |  | RBP5 | 0.621621622 | 0.810810811 | 0.810810811 |
| fstb | fstb | FST | 0.620689655 | 0.482758621 | 1.017241379 |
| ncaldb | ncaldb | NCALD | 0.620689655 | 0.405172414 | 1.551724138 |
| CABZ01067232.1 | - |  | 0.620689655 | 0.318965517 | 0.75862069 |
| baiap2a |  | BAIAP2 | 0.62 | 0.52 | 0.86 |
| zgc:165653 | zgc:165653 | ACTN2 | 0.62 | 0.28 | 1.08 |
| lrtm2a | lrtm2a | LRTM2 | 0.62 | 0.28 | 0.84 |
| dnajc5aa |  | DNAJC5 | 0.619047619 | 0.847619048 | 0.980952381 |
| col4a6 | col4a6 | COL4A4 | 0.619047619 | 0.342857143 | 0.8 |
| si:dkey-228b2.5 | myorg | MYORG | 0.617021277 | 0.276595745 | 0.829787234 |
| enc3 | enc3 | ENC1 | 0.616666667 | 0.533333333 | 0.95 |
| msmo1 | msmo1 | MSMO1 | 0.616161616 | 0.606060606 | 0.656565657 |
| rpz | rpz |  | 0.614583333 | 0.239583333 | 1 |
| pdca | pdca | PDC | 0.613636364 | 0.568181818 | 1.090909091 |
| egr4 | egr4 | EGR1 | 0.612903226 | 0.784946237 | 1.032258065 |
| SAMD8 | - | SAMD4A | 0.612244898 | 0.448979592 | 0.918367347 |
| gprc5ba | gprc5ba | GPRC5B | 0.608695652 | 0.217391304 | 0.934782609 |
| gna11a | gna11a | GNAL | 0.608695652 | 0.195652174 | 0.97826087 |
| ythdc1 | ythdc1 | YTHDC1 | 0.606382979 | 0.436170213 | 1.010638298 |
| palm1b | palm1b | PALM | 0.605150215 | 0.450643777 | 1.051502146 |
| dnm1b | dnm1b | DNM1 | 0.604651163 | 0.302325581 | 0.790697674 |
| gas1a | gas1a | GAS1 | 0.604477612 | 0.26119403 | 0.776119403 |
| TMEM27 |  | TMEM33 | 0.603960396 | 0.673267327 | 0.772277228 |
| hgs | hgs | HGS | 0.603174603 | 0.333333333 | 1.031746032 |
| per1a | per1a | PER1 | 0.6 | 0.388888889 | 1.766666667 |
| fndc7b | fndc7b | FNDC1 | 0.6 | 0.8 | 1.275 |
| mhc1zca | mhc1zca | HLA-A | 0.6 | 0.6 | 1.55 |
| clpxa_1 | - | CLPX | 0.6 | 0.464285714 | 0.921428571 |
| foxo3b | foxo3b | FOXO3 | 0.6 | 0.330434783 | 0.773913043 |
| aqp9b | aqp9b | AQP9 | 0.6 | 0.295652174 | 1 |
| si:ch211-217k17.9 | si:ch211-217k17.9 |  | 0.598290598 | 0.632478632 | 1.085470085 |
| rbpms2a | rbpms2a | RBPMS2 | 0.597402597 | 0.357142857 | 1.181818182 |
| si:ch211-286o17.1 | si:ch211-286o17.1 | CD34 | 0.597222222 | 0.486111111 | 1.027777778 |
| vti1a | vti1a | VTI1A | 0.595238095 | 0.285714286 | 1.142857143 |
| rps8b | rps8b | RPS8 | 0.594594595 | 0.297297297 | 1.378378378 |
| rab9a | rab9a | RAB9A | 0.59375 | 0.5 | 2.1875 |
| loxl2a | loxl2a | LOXL2 | 0.592592593 | 0.543209877 | 1.283950617 |
| soul4 | soul4 | HEBP2 | 0.592592593 | 0.277777778 | 1.277777778 |
| sh3bp5la | sh3bp5la | SH3BP5L | 0.591836735 | 0.489795918 | 0.93877551 |
| prpf4ba | prpf4ba | PRPF4B | 0.590909091 | 0.363636364 | 0.886363636 |
| cyp2p9 | cyp2p9 | CYP2J2 | 0.58974359 | 0.641025641 | 1.153846154 |
| rbm15 | rbm15 | RBM15 | 0.589285714 | 0.428571429 | 0.875 |
| mych | mych | MYC | 0.587737844 | 0.484143763 | 1.059196617 |
| stxbp1b | stxbp1b | STXBP1 | 0.584269663 | 0.382022472 | 0.97752809 |
| snx8b |  | SNX8 | 0.583333333 | 0.5625 | 0.958333333 |
| si:zfos-1837d3.1 | si:zfos-1837d3.1 |  | 0.583333333 | 0.5 | 0.791666667 |
| si:ch211-134a4.2 | ly6m3 | CD180 | 0.583333333 | 0.375 | 1.5 |
| ggh | ggh | GGH | 0.583333333 | 0.361111111 | 1.361111111 |

| hbs1l | hbs1l | HBS1L | 0.582417582 | 0.483516484 | 1.087912088 |
| --- | --- | --- | --- | --- | --- |
| fam219ab | fam219ab | FAM219A | 0.581081081 | 0.391891892 | 0.702702703 |
| clip3 | clip3 | CLIP3 | 0.581081081 | 0.351351351 | 1.202702703 |
| cdkn1a | cdkn1a | CDKN1A | 0.579399142 | 0.313304721 | 1.635193133 |
| pou3f2b | pou3f2b | POU3F2 | 0.578947368 | 0.263157895 | 1.039473684 |
| bag3 | bag3 | BAG3 | 0.578125 | 0.40625 | 1 |
| adnp2b | adnp2b | ADNP2 | 0.577464789 | 0.323943662 | 0.985915493 |
| si:dkey-1f1.3_1 |  |  | 0.576923077 | 1.153846154 | 0.730769231 |
| fosab_1 |  | FOS | 0.575 | 0.6 | 0.675 |
| dhrs3a | dhrs3a | DHRS3 | 0.574468085 | 0.340425532 | 0.212765957 |
| rcvrna |  | RCVRN | 0.572864322 | 0.512562814 | 0.924623116 |
| mylk4a | mylk4a | MYLK4 | 0.571428571 | 0.39047619 | 0.942857143 |
| mmp14b | mmp14b | MMP14 | 0.571428571 | 0.385714286 | 1.128571429 |
| stat3 | stat3 | STAT3 | 0.571428571 | 0.19047619 | 0.880952381 |
| cplx3b | cplx3b | CPLX3 | 0.569767442 | 0.325581395 | 0.709302326 |
| rell1 |  | RELL1 | 0.568627451 | 0.529411765 | 1.411764706 |
| nrbp2a | nrbp2a | NRBP2 | 0.568181818 | 0.386363636 | 1.25 |
| si:dkeyp-72g9.4 | si:dkeyp-72g9.4 | DEPP1 | 0.56779661 | 0.43220339 | 1.194915254 |
| syt11b | syt11b | SYT11 | 0.566666667 | 0.4 | 0.75 |
| cacng1a | cacng1a | CACNG1 | 0.56626506 | 0.421686747 | 0.891566265 |
| keap1a | keap1a | KEAP1 | 0.564516129 | 1.209677419 | 2.306451613 |
| kdm6bb | kdm6bb | KDM6B | 0.564516129 | 0.419354839 | 0.822580645 |
| uhrf1 | uhrf1 | UHRF1 | 0.564516129 | 0.274193548 | 0.516129032 |
| timp2b | timp2b | TIMP2 | 0.5625 | 0.270833333 | 1.645833333 |
| sh3gl2 | sh3gl2a | SH3GL2 | 0.561128527 | 0.360501567 | 0.987460815 |
| guca1g | guca1g | GUCA1C | 0.560606061 | 0.363636364 | 0.636363636 |
| itga6b | itga6b | ITGA6 | 0.558139535 | 0.488372093 | 0.736434109 |
| si:dkey-18a10.3 | si:dkey-18a10.3 | HSF2 | 0.555555556 | 0.222222222 | 0.444444444 |
| si:dkey-239h2.3 | si:dkey-239h2.3 |  | 0.554054054 | 0.695945946 | 1.209459459 |
| snrkb | snrkb | SNRK | 0.551020408 | 0.408163265 | 0.836734694 |
| nid1b | nid1b | NID1 | 0.551020408 | 0.285714286 | 1.020408163 |
| rxrba | rxrba | RXRB | 0.551020408 | 0.285714286 | 0.836734694 |
| ranbp10 | ranbp10 | RANBP10 | 0.551020408 | 0.244897959 | 1.102040816 |
| ppp3cca | ppp3cca | PPP3CC | 0.549019608 | 0.31372549 | 1.098039216 |
| sec31a | sec31a | SEC31A | 0.549019608 | 0.31372549 | 0.764705882 |
| kera | kera | KERA | 0.548387097 | 0.451612903 | 1.129032258 |
| opn1mw2 | opn1mw2 | OPN1MW2 | 0.547619048 | 0.952380952 | 1.30952381 |
| DSP (1 of many) | - | DSP | 0.547169811 | 0.471698113 | 0.849056604 |
| scn1ba | scn1ba | SCN1B | 0.545454545 | 0.309090909 | 1.054545455 |
| rnf141 | rnf141 | RNF141 | 0.545454545 | 0.295454545 | 1.068181818 |
| slc4a4a | slc4a4a | SLC4A4 | 0.544117647 | 0.25 | 1 |
| cplx4a | cplx4a | CPLX4 | 0.540983607 | 0.295081967 | 1.008196721 |
| sik1 | sik1 | SIK1 | 0.540229885 | 0.494252874 | 0.850574713 |
| fgfbp1b_1 | - | FGFBP1 | 0.535714286 | 0.25 | 1.035714286 |
| plecb | plecb | PLEC | 0.534482759 | 0.379310345 | 0.844827586 |
| gch2 | gch2 | GCH1 | 0.534161491 | 0.341614907 | 0.527950311 |
| plp2 | plp2 | PLP2 | 0.532374101 | 0.309352518 | 0.776978417 |
| cadm1a | cadm1a | CADM1 | 0.529411765 | 0.254901961 | 0.921568627 |
| nt5c2b | nt5c2b | NT5C2 | 0.528301887 | 0.169811321 | 0.773584906 |
| frmd8 | frmd8 | FRMD8 | 0.527272727 | 0.327272727 | 0.927272727 |
| hsp70.2 | hsp70.2 | HSPA2 | 0.526315789 | 0.280701754 | 1.052631579 |
| slc34a2a | slc34a2a | SLC34A2 | 0.525714286 | 0.342857143 | 1.062857143 |
| gpcpd1 | gpcpd1 | GPCPD1 | 0.524390244 | 0.317073171 | 1.097560976 |
| pfkfb4b | pfkfb4b | PFKFB4 | 0.522988506 | 0.379310345 | 1.17816092 |
| add1 | add1 | ADD1 | 0.520833333 | 0.229166667 | 0.770833333 |
| ptgs2a |  | PTGS2 | 0.520547945 | 0.547945205 | 1.068493151 |
| ccdc85b | ccdc85b | CCDC85B | 0.518987342 | 0.417721519 | 0.886075949 |

| foxg1b | foxg1b | FOXG1 | 0.518796992 | 0.503759398 | 1.511278195 |
| --- | --- | --- | --- | --- | --- |
| gpd1l | gpd1l | GPD1L | 0.518518519 | 0.407407407 | 0.777777778 |
| syngr3a | syngr3a | SYNGR3 | 0.51754386 | 0.298245614 | 0.912280702 |
| rhcga | rhcga | RHCG | 0.517295597 | 0.36163522 | 0.995283019 |
| gdf10a | gdf10a | GDF10 | 0.516129032 | 0.322580645 | 0.725806452 |
| gpm6ba | gpm6ba | GPM6A | 0.516129032 | 0.080645161 | 0.870967742 |
| bag2 | bag2 | BAG2 | 0.515789474 | 0.252631579 | 0.842105263 |
| CABZ01066926.1 | - |  | 0.515625 | 0.34375 | 1.421875 |
| evpla | evpla | EVPL | 0.515625 | 0.34375 | 0.890625 |
| zgc:171679 | zgc:171679 | RFPL4B | 0.514705882 | 0.632352941 | 1.294117647 |
| isg15 | isg15 | ISG15 | 0.514285714 | 0.571428571 | 1.685714286 |
| cdk5r1b | cdk5r1b | CDK5R1 | 0.513761468 | 0.220183486 | 0.788990826 |
| CFP (1 of many) |  | CFP | 0.512820513 | 0.512820513 | 1.205128205 |
| si:ch211-134a4.1 | ly6m2 | LYPD8 | 0.510204082 | 0.489795918 | 0.755102041 |
| trim35-28 | trim35-28 | TRIM32 | 0.509433962 | 0.339622642 | 0.962264151 |
| scdb | scdb | SCD | 0.509433962 | 0.301886792 | 0.905660377 |
| C7 (1 of many) | - | C7 | 0.5 | 0.666666667 | 0.75 |
| zgc:91909_1 | rab7b | RAB8B | 0.5 | 0.5 | 1.25 |
| ahnak | ahnak | AHNAK | 0.5 | 0.362068966 | 0.827586207 |
| rapsn | rapsn | RAPSN | 0.5 | 0.35 | 0.933333333 |
| shox | shox | SHOX | 0.5 | 0.333333333 | 0.864583333 |
| acacb | acacb | ACACB | 0.5 | 0.285714286 | 0.875 |
| clockb | clockb | CLOCK | 0.5 | 0.229166667 | 0.9375 |
| dbpa | dbpa | DBP | 0.496732026 | 0.261437908 | 1.08496732 |
| atf5a | atf5a | ATF5 | 0.494949495 | 0.313131313 | 1.202020202 |
| unc119a | unc119a | UNC119 | 0.490196078 | 0.31372549 | 0.745098039 |
| CABZ01083501.2 | - |  | 0.489795918 | 0.489795918 | 0.93877551 |
| ZNF319 (1 of many) | - | ZNF76 | 0.489361702 | 0.255319149 | 0.787234043 |
| si:ch211-170d8.5 | asgrl1 | ADGR2 | 0.488372093 | 0.488372093 | 1.093023256 |
| pttg1ipa | pttg1ipa | PTTG1 | 0.487179487 | 0.333333333 | 1.141025641 |
| zfp36l1a | zfp36l1a | ZFP36L1 | 0.486666667 | 0.193333333 | 0.966666667 |
| nr4a1 | nr4a1 | NR4A1 | 0.483870968 | 0.580645161 | 0.795698925 |
| snoU2_19_1 | - |  | 0.483050847 | 0.703389831 | 1.025423729 |
| tomm70a | tomm70a | TOMM70 | 0.482758621 | 0.293103448 | 0.804597701 |
| gria2a | gria2a | GRIA2 | 0.482758621 | 0.310344828 | 0.948275862 |
| itgb1a | itgb1a | ITGB1 | 0.480519481 | 0.207792208 | 0.87012987 |
| atf3 | atf3 | ATF3 | 0.48 | 0.32 | 1.12 |
| hsp70l | hsp70l | HSPA1L | 0.479289941 | 0.639053254 | 1.313609467 |
| nova1 | nova1 | NOVA1 | 0.477272727 | 0.204545455 | 0.727272727 |
| AC024175.7 | - |  | 0.470588235 | 0.470588235 | 0 |
| caly | caly | CALY | 0.470588235 | 0.235294118 | 0.921568627 |
| pdk2b | pdk2b | PDK2 | 0.468384075 | 0.466042155 | 1.156908665 |
| ppp2ca | ppp2caa | PPP2CB | 0.468085106 | 0.29787234 | 0.978723404 |
| col4a2 | col4a2 | COL4A2 | 0.467741935 | 0.209677419 | 0.887096774 |
| klf15 | klf15 | KLF15 | 0.465909091 | 0.227272727 | 1.102272727 |
| tspan14 | tspan14 | TSPAN14 | 0.465517241 | 0.206896552 | 0.896551724 |
| ppp1r27a | ppp1r27a | PPP1R27 | 0.465517241 | 0.189655172 | 0.620689655 |
| nagk | nagk | NAGK | 0.465116279 | 0.465116279 | 1.162790698 |
| upp2 | upp2 | UPP2 | 0.464285714 | 0.464285714 | 0.821428571 |
| nocta | nocta | NOCT | 0.463768116 | 0.434782609 | 0.797101449 |
| CABZ01044277.1 | - |  | 0.462962963 | 0.388888889 | 0.277777778 |
| klc1a | klc1a | KLC1 | 0.462686567 | 0.194029851 | 0.955223881 |
| si:ch1073-440b2.1 | si:ch1073-440b2.1 | LONRF2 | 0.461538462 | 0.230769231 | 0.807692308 |
| barhl1b | barhl1b | BARHL1 | 0.461538462 | 0.192307692 | 0.807692308 |
| cyp24a1 | cyp24a1 | CYP24A1 | 0.458333333 | 0.819444444 | 0.777777778 |
| a1cf | a1cf | A1CF | 0.456140351 | 0.157894737 | 1.105263158 |
| wu:fi18e02 | scarb2c | SCARB2 | 0.453781513 | 0.411764706 | 1.067226891 |

| igfbp1a | igfbp1a | IGFBP1 | 0.453038674 | 0.552486188 | 1.071823204 |
| --- | --- | --- | --- | --- | --- |
| xpr1a | xpr1a | XPR1 | 0.452830189 | 0.094339623 | 0.735849057 |
| HPX (1 of many) | - | MMP20 | 0.452380952 | 0.547619048 | 1.261904762 |
| sypa | sypa | SYP | 0.45026178 | 0.232984293 | 0.910994764 |
| tmem30aa | tmem30aa | TMEM30A | 0.45 | 0.2375 | 0.9875 |
| zgc:113531 | zgc:113531 | CHRD | 0.446153846 | 0.261538462 | 0.753846154 |
| hlfa | hlfa | HLF | 0.44198895 | 0.154696133 | 0.806629834 |
| sox9a | sox9a | SOX9 | 0.441558442 | 0.142857143 | 0.987012987 |
| nudt4a | nudt4a | NUDT4 | 0.43877551 | 0.295918367 | 0.683673469 |
| pink1 | pink1 | PINK1 | 0.434782609 | 0.260869565 | 0.97826087 |
| ssx2ipb | ssx2ipb | SSX2IP | 0.432432432 | 0.297297297 | 1.189189189 |
| pnp5a | pnp5a | PNP | 0.429032258 | 0.34516129 | 0.751612903 |
| si:ch211-133l5.5 | si:ch211-133l5.5 |  | 0.428571429 | 0.464285714 | 1.428571429 |
| itgb1b.1 | itgb1b.1 | ITGB1 | 0.426829268 | 0.219512195 | 0.780487805 |
| nr1d2a | nr1d2a | NR1D2 | 0.425925926 | 0.305555556 | 1.393518519 |
| myha | myha | MYH6 | 0.423076923 | 1.019230769 | 1.346153846 |
| socs1a | socs1a | SOCS1 | 0.423076923 | 0.653846154 | 1 |
| pcdh1a4 | pcdh1a4 | PCDHA8 | 0.423076923 | 0.461538462 | 0.634615385 |
| si:ch211-194c3.5 | si:ch211-194c3.5 | C20orf194 | 0.42 | 0.2 | 0.76 |
| itgav | itgav | ITGAV | 0.418803419 | 0.222222222 | 0.914529915 |
| armc1 | armc1 | ARMC1 | 0.418604651 | 0.255813953 | 1.069767442 |
| ldlra | ldlra | LDLR | 0.418181818 | 0.236363636 | 1.072727273 |
| gpm6bb | gpm6bb | GPM6B | 0.416666667 | 0.208333333 | 0.833333333 |
| lpin1 | lpin1 | LPIN1 | 0.415300546 | 0.240437158 | 1.06557377 |
| epgn | epgn | EPGN | 0.41509434 | 0.622641509 | 1.150943396 |
| phf8 | phf8 | PHF8 | 0.414634146 | 0.146341463 | 0.780487805 |
| slc25a25a | slc25a25a | SLC25A25 | 0.413793103 | 0.551724138 | 0.931034483 |
| synm | synm | SYNM | 0.413043478 | 0.282608696 | 0.717391304 |
| col4a3bpb | cert1b | CERT1 | 0.413043478 | 0.22826087 | 1.065217391 |
| tet3 | tet3 | TET3 | 0.412698413 | 0.174603175 | 0.873015873 |
| ppp3ccb | ppp3ccb | PPP3CC | 0.412280702 | 0.201754386 | 0.868421053 |
| si:dkey-31i7.1 | si:dkey-31i7.1 |  | 0.411764706 | 0.147058824 | 0.852941176 |
| tpd52 | tpd52 | TPD52 | 0.410714286 | 0.125 | 0.642857143 |
| guk1a | guk1a | GUK1 | 0.409638554 | 0.21686747 | 1 |
| glra3 | glra3 | GLRA3 | 0.409090909 | 0.295454545 | 0.727272727 |
| tmem161a | tmem161a | TMEM161A | 0.409090909 | 0.295454545 | 0.704545455 |
| pvrl1b | nectin1b | NECTIN1 | 0.408163265 | 0.204081633 | 0.918367347 |
| cxcl18b | cxcl18b | PPBP | 0.407407407 | 0.481481481 | 1 |
| marcksa | marcksa | MARCKS | 0.406976744 | 0.162790698 | 0.790697674 |
| zgc:162255 | zgc:162255 | C6orf89 | 0.403846154 | 0.269230769 | 1.076923077 |
| atp6v0a1b | atp6v0a1b | ATP6V0A1 | 0.403225806 | 0.241935484 | 0.758064516 |
| sptbn2 | sptbn2 | SPTBN2 | 0.403225806 | 0.177419355 | 0.758064516 |
| isca1 | isca1 | ISCA1 | 0.402985075 | 0.263681592 | 1.109452736 |
| calr3b | calr3b | CALB2 | 0.401913876 | 0.124401914 | 0.688995215 |
| ube2ql1 | ube2ql1 | UBE2QL1 | 0.397058824 | 0.205882353 | 0.75 |
| nppa | nppa | NPPA | 0.396226415 | 0.339622642 | 0.79245283 |
| prkcba | prkcba | PRKCB | 0.395348837 | 0.139534884 | 0.511627907 |
| sfrp2 | sfrp2 | SFRP2 | 0.395061728 | 0.308641975 | 0.679012346 |
| sept8b | sept8b | SEPTIN11 | 0.388059701 | 0.432835821 | 0.940298507 |
| znf648 | znf648 | ZNF648 | 0.387096774 | 0.430107527 | 0.52688172 |
| ddit4 | ddit4 | DDIT4 | 0.386904762 | 0.571428571 | 1.642857143 |
| mpzl1l | mpzl1l | MPZL1 | 0.386363636 | 0.272727273 | 1.159090909 |
| myom1b | myom1b | MYOM1 | 0.385321101 | 0.174311927 | 0.733944954 |
| pfkfb3 | pfkfb3 | PFKFB3 | 0.383561644 | 0.232876712 | 0.767123288 |
| si:ch211-66o10.6 | si:ch211-66o10.6 |  | 0.381443299 | 0.329896907 | 0.855670103 |
| sp8b | sp8b | SP8 | 0.380952381 | 0.261904762 | 0.738095238 |
| eral1 | eral1 | ERAL1 | 0.38 | 0.3 | 0.86 |

| rps6kb1a | rps6kb1a | RPS6KA1 | 0.377777778 | 0.288888889 | 0.844444444 |
| --- | --- | --- | --- | --- | --- |
| serpine1 | serpine1 | SERPINE1 | 0.375 | 0.517857143 | 1.553571429 |
| tpmt.2 | tpmt.2 | TPMT | 0.375 | 1.05 | 7.25 |
| syngr1a | syngr1a | SYNGR1 | 0.373831776 | 0.168224299 | 0.841121495 |
| nampta | nampta | NAMPT | 0.373134328 | 0.179104478 | 1.134328358 |
| arl4ca | arl4ca | ARL4C | 0.37037037 | 0.222222222 | 0.740740741 |
| pacsin1a | pacsin1a | PACSIN1 | 0.367088608 | 0.202531646 | 0.632911392 |
| pank1b | pank1b | PANK1 | 0.365384615 | 0.115384615 | 0.75 |
| ing5a | ing5a | ING5 | 0.362068966 | 0.224137931 | 1.017241379 |
| grinaa | grinaa | GRINA | 0.361702128 | 0.191489362 | 1.234042553 |
| ptx3a | ptx3a | PTX3 | 0.36 | 0.36 | 1.08 |
| pnp5b | pnp5b | PNP | 0.36 | 0.36 | 1.88 |
| scarb2 | scarb2 | SCARB2 | 0.359375 | 0.234375 | 0.671875 |
| pitpnab | pitpnab | PITPNA | 0.358333333 | 0.208333333 | 0.85 |
| zc3h7b | zc3h7ba | ZC3H7B | 0.357142857 | 0.196428571 | 0.767857143 |
| prnpa | prnpa | PRNP | 0.355932203 | 0.288135593 | 1.677966102 |
| igfbp1b | igfbp1b | IGFBP1 | 0.347826087 | 0.326086957 | 0.652173913 |
| pcsk2 | pcsk2 | PCSK2 | 0.346153846 | 0.076923077 | 0.711538462 |
| cipca | cipca | CIPC | 0.340909091 | 0.272727273 | 1.272727273 |
| dera | dera | DERA | 0.340425532 | 0.127659574 | 0.85106383 |
| vwa11 | vwa11 | VMA1 | 0.340206186 | 0.082474227 | 0.762886598 |
| ugp2a | ugp2a | UGP2 | 0.337016575 | 0.209944751 | 0.939226519 |
| fkbp5 | fkbp5 | FKBP5 | 0.334285714 | 0.257142857 | 1.248571429 |
| fitm1 | fitm1 | FITM1 | 0.333333333 | 0.736842105 | 0.964912281 |
| nr1d4b | nr1d4b | NR1D1 | 0.333333333 | 0.266666667 | 4.6 |
| tmem176l.3a | tmem176l.3a | MS4A6A | 0.333333333 | 0.19047619 | 1.166666667 |
| chp1 | chp1 | CHP1 | 0.333333333 | 0.111111111 | 0.733333333 |
| tcap | tcap | TCAP | 0.328358209 | 0.371002132 | 0.735607676 |
| hsp70.3 | hsp70.3 | HSPA1L | 0.328125 | 0.421875 | 1.53125 |
| fbxo32 | fbxo32 | FBXO32 | 0.326923077 | 0.365384615 | 1.365384615 |
| rtn2a | rtn2a | RTN1 | 0.326923077 | 0.096153846 | 0.730769231 |
| nfil3-6 | nfil3-6 | NFIL3 | 0.326086957 | 0.434782609 | 0.652173913 |
| crema | crema | CREM | 0.325581395 | 0.209302326 | 0.511627907 |
| slc2a1b | slc2a1b | SLC2A1 | 0.325581395 | 0.069767442 | 0.651162791 |
| sun1 | sun1 | SUN1 | 0.323529412 | 0.220588235 | 0.941176471 |
| rac1b | rac1b | RAC1 | 0.323076923 | 0.2 | 0.676923077 |
| tmub2 | tmub2 | TMUB2 | 0.322580645 | 0.177419355 | 1.080645161 |
| CU856539.1 | - |  | 0.321428571 | 0.142857143 | 0.75 |
| znf1179 | znf1179 | ZNF729 | 0.319148936 | 0.460992908 | 1.312056738 |
| grhl1 | grhl1 | GRHL1 | 0.319148936 | 0.170212766 | 0.829787234 |
| ppp2r5d | ppp2r5d | PPP2R5D | 0.317647059 | 0.211764706 | 0.752941176 |
| dgat2 | dgat2 | DGAT2 | 0.316666667 | 0.216666667 | 0.566666667 |
| stk35 | stk35 | STK35 | 0.315789474 | 0.333333333 | 0.894736842 |
| mmp9 | mmp9 | MMP9 | 0.315789474 | 0.263157895 | 1.526315789 |
| vsnl1b | vsnl1b | VSNL1 | 0.315555556 | 0.164444444 | 0.804444444 |
| paqr6 | paqr6 | PAQR6 | 0.315068493 | 0.205479452 | 0.98630137 |
| si:dkey-103g5.3 | si:dkey-103g5.3 |  | 0.307692308 | 0.230769231 | 0.730769231 |
| sptbn1 | sptbn1 | SPTBN1 | 0.30625 | 0.1625 | 0.8 |
| si:dkey-202l22.6 | si:dkey-202l22.6 | URGCP-MRPS24 | 0.304347826 | 0.31884058 | 0.695652174 |
| timp4a | timp4.3 | TIMP2 | 0.3 | 0.3 | 0.775 |
| si:ch211-197k17.3 | si:ch211-197k17.3 | AVPI1 | 0.3 | 0.2375 | 0.8625 |
| oat | oat | OAT | 0.298245614 | 0.070175439 | 0.719298246 |
| gls2a | gls2a | GLS2 | 0.296296296 | 0.092592593 | 0.518518519 |
| LEPROT | leprot | LEPROT | 0.291666667 | 1.125 | 0.958333333 |
| adss | adss2 | ADSS2 | 0.288461538 | 0.134615385 | 0.711538462 |
| zgc:162999 | zgc:162999 | PCBP3 | 0.283783784 | 0.175675676 | 0.405405405 |
| si:ch211-264f5.6 | si:ch211-264f5.6 | CEACAM20 | 0.282608696 | 0.065217391 | 0.913043478 |

| limd2 | limd2 | LIMD2 | 0.28125 | 0.078125 | 0.546875 |
| --- | --- | --- | --- | --- | --- |
| brcc3 | brcc3 | BRCC3 | 0.277777778 | 0.111111111 | 1.25 |
| napba | napba | NAPB | 0.274509804 | 0.176470588 | 0.745098039 |
| daglb | daglb | DAGLB | 0.274193548 | 0.096774194 | 1.016129032 |
| socs3a_1 | - | SOCS3 | 0.272727273 | 0.772727273 | 0 |
| mgst2 | mgst2 | MGST2 | 0.272727273 | 0.418181818 | 1.945454545 |
| rab11fip4a | rab11fip4a | RAB11FIP4 | 0.271186441 | 0.084745763 | 0.830508475 |
| ap1m1 | ap1m1 | AP1M1 | 0.269230769 | 0.076923077 | 0.769230769 |
| cds1 | cds1 | CDS1 | 0.266666667 | 0.066666667 | 1.466666667 |
| fam117ba | fam117ba | FAM13A | 0.264150943 | 0.20754717 | 0.773584906 |
| CABZ01075204.1 | - |  | 0.260869565 | 0.239130435 | 0.5 |
| slc25a1b | slc25a1b | SLC25A1 | 0.25974026 | 0.168831169 | 0.532467532 |
| cmc4 | cmc4 | CMC1 | 0.259259259 | 0.555555556 | 0.851851852 |
| si:dkey-85k7.7 | si:dkey-85k7.7 |  | 0.258064516 | 0.215053763 | 0.978494624 |
| nptxra | nptxra | NPTXR | 0.254901961 | 0.098039216 | 0.62745098 |
| pmaip1 | pmaip1 | PMAIP1 | 0.25 | 0.6875 | 1.3125 |
| acox3 | acox3 | ACOX3 | 0.245283019 | 0.226415094 | 0.849056604 |
| slc2a1a | slc2a1a | SLC2A1 | 0.244444444 | 0.133333333 | 1.044444444 |
| ppp3r1a | ppp3r1a | PPP3R1 | 0.243478261 | 0.113043478 | 0.582608696 |
| zgc:193593_1 | – |  | 0.242857143 | 0.442857143 | 2.742857143 |
| hmga2 | hmga2 | HMGA2 | 0.241935484 | 0.14516129 | 0.564516129 |
| RGS9BP | rgs9bp | RGS3 | 0.24137931 | 0.098522167 | 0.763546798 |
| csrnp1b | csrnp1b | CSRNP1 | 0.240740741 | 0.138888889 | 0.842592593 |
| prodha | prodha | PRODH | 0.233870968 | 0.129032258 | 0.766129032 |
| slc6a11b | slc6a11b | SLC6A11 | 0.233333333 | 0.083333333 | 0.933333333 |
| irs2a | irs2a | IRS2 | 0.230769231 | 0.128205128 | 1.076923077 |
| aldh3a2b | aldh3a2b | ALDH3A1 | 0.226415094 | 0.056603774 | 0.830188679 |
| mat2ab | mat2ab | MAT2A | 0.226315789 | 0.073684211 | 0.689473684 |
| klhl40a | klhl40a | KLHL40 | 0.222222222 | 0.055555556 | 1.194444444 |
| socs3a_2 | - | SOCS3 | 0.222222222 | 0 | 1.333333333 |
| cyp2p8 | cyp2p8 | CYP2J2 | 0.220779221 | 0.5 | 1.025974026 |
| fbxo28 | fbxo28 | FBXO28 | 0.218181818 | 0.090909091 | 0.654545455 |
| cyp2p7 | cyp2p7 | CYP2J2 | 0.206521739 | 0.880434783 | 0.717391304 |
| tefb | tefb | TEF | 0.203947368 | 0.052631579 | 0.776315789 |
| tusc5a | trarg1a | TRARG1 | 0.196078431 | 0.117647059 | 0.862745098 |
| ca2 | ca2 | CA2 | 0.187878788 | 0.012121212 | 0.272727273 |
| zgc:136410 | zgc:136410 |  | 0.173076923 | 0.057692308 | 0.384615385 |
| ca5a | ca5a | CA5B | 0.166666667 | 0.055555556 | 0.703703704 |
| myf6 | myf6 | MYF6 | 0.162790698 | 0.209302326 | 0.720930233 |
| si:dkey-184p18.2 | si:dkey-184p18.2 | C18orf21 | 0.159090909 | 0.159090909 | 1.136363636 |
| cxcl8b.1 | cxcl8b.1 | CXCL12 | 0.157894737 | 0.157894737 | 1.105263158 |
| ndfip1l | ndfip1l | NDFIP1 | 0.157894737 | 0.140350877 | 0.771929825 |
| mapta | mapta | MAPT | 0.155555556 | 0.033333333 | 0.733333333 |
| klf9 | klf9 | KLF9 | 0.153061224 | 0.066326531 | 0.857142857 |
| thy1 | thy1 | THY1 | 0.152777778 | 0.666666667 | 1.333333333 |
| si:dkey-19d21.2 | si:dkey-19d21.2 |  | 0.151515152 | 0.242424242 | 1.575757576 |
| cyp2aa7 | cyp2aa7 | CYP2C8 | 0.15 | 0.15 | 1.6 |
| si:ch73-14h1.2 | si:ch73-14h1.2 |  | 0.136363636 | 0.295454545 | 1.181818182 |
| itln3 | itln3 | ITLN1 | 0.129032258 | 0.935483871 | 0.967741935 |
| AC024175.17 | - |  | 0.121801181 | 0.158956693 | 0.175688976 |
| si:ch211-66k16.27 | si:ch211-66k16.27 |  | 0.111111111 | 0.166666667 | 0.537037037 |
| si:ch211-186e20.6p | si:ch211-186e20.6p |  | 0.064516129 | 0.483870968 | 0.903225806 |
| si:ch211-114l13.11 | si:ch211-114l13.11 |  | 0.050505051 | 0.222222222 | 2.202020202 |
| myhc4 | myhc4 | MYH4 | 0.047904192 | 0.810379242 | 1.592814371 |
| RNaseP_nuc_1 | – | RPP40 | 0.035460993 | 0.014184397 | 0.007092199 |
| si:dkeyp-3b12.8 | si:dkeyp-3b12.8 |  | 0.02739726 | 0.030821918 | 0.006849315 |
| 5_8S_rRNA_8 | – |  | 0.024383164 | 0.019448476 | 0.025253991 |

| rn7sk | rn7sk | RN7SK | 0.018549747 | 0.04272063 | 0.012928612 |
| --- | --- | --- | --- | --- | --- |
| serpina7 | serpina7 | SERPINA9 | 0.016949153 | 0.694915254 | 1.13559322 |
| U4_1 | – |  | 0.015936255 | 0.015936255 | 0 |
| BX537263.2 | – |  | 0.012710083 | 0.022832872 | 0.01015513 |
| si:dkey-153m14.1 | si:dkey-153m14.1 |  | 0.012512491 | 0.019774434 | 0.011502524 |
| zgc:158463 | zgc:158463 |  | 0.010274094 | 0.019097728 | 0.010035906 |
| hamp | hamp | HAMP | 0.00990099 | 0.178217822 | 1.613861386 |
| Metazoa_SRP_1 | – |  | 0.007559987 | 0.022899091 | 0.005697381 |
| si:dkey-77p23.8 | si:dkey-77p23.8 |  | 0.006954103 | 0.028743625 | 0.001390821 |
| si:dkey-77p23.6 | si:dkey-77p23.6 |  | 0.005714286 | 0.014285714 | 0.008571429 |
| si:dkeyp-3b12.12 | si:dkeyp-3b12.12 |  | 0 | 0.156862745 | 0 |
| RNaseP_nuc_3 | – | RPP40 | 0 | 0.044444444 | 0.02962963 |
| Metazoa_SRP_3 | – |  | 0 | 0.035714286 | 0 |
| AC024175.3 | – |  | 0 | 0 | 5.111111111 |
| Metazoa_SRP_38 | – |  | 0 | 0 | 0.018691589 |
