# Supplementary material for: Generation and characterization of keap1a- and keap1b-knockout zebrafish
Source: Redox Biol. 2020 Aug 11;36:101667. doi: 10.1016/j.redox.2020.101667 (PMC7452054; doi:10.1016/j.redox.2020.101667)
Supplement: Multimedia component 2 [file mmc2.docx]

Table S4. Biological processes up-regulated by sulforaphane treatment.

| Category | Term | Count | % | P Value | Genes | List Total | Pop Hits | Pop Total | Fold Enrichment | Bonferroni | Benjamini | FDR |
| --- | --- | --- | --- | --- | --- | --- | --- | --- | --- | --- | --- | --- |
| GOTERM_BP_DIRECT | GO:0055114~oxidation-reduction process | 38 | 19.79 | 9.66E-19 | ME1, C15ORF48, FTMT, HTATIP2, PGD, UGDH, PRDX1, GSR, P4HA2, PIR, TSTA3, GSTO1, HIGD1A, HPD, PTGR1, AIFM2, DHRS13, AIFM1, CYP2C8, DHRS11, QDPR, HGD, CBR3, CYB561A3, SOD3, DHDH, RDH12, DHRS2, RDH10, ADO, DHRS4, G6PD, TXN, TXNRD3, BCO1, ABCC4, AOC2, MGST1 | 178 | 592 | 16792 | 6.055420589 | 9.88E-16 | 9.88E-16 | 1.53E-15 |
| GOTERM_BP_DIRECT | GO:0006750~glutathione biosynthetic process | 6 | 3.125 | 4.89E-07 | HAGH, GSS, GCLC, GGT1, GCLM, MGST2 | 178 | 16 | 16792 | 35.37640449 | 5.00E-04 | 2.50E-04 | 7.75E-04 |
| GOTERM_BP_DIRECT | GO:0098869~cellular oxidant detoxification | 9 | 4.688 | 7.04E-07 | MGST3, GPX1, GSR, TXN, TXNRD3, GSTO1, MGST1, GSTP1, MGST2 | 178 | 70 | 16792 | 12.12905297 | 7.19E-04 | 2.40E-04 | 1.11E-03 |
| GOTERM_BP_DIRECT | GO:0006749~glutathione metabolic process | 8 | 4.167 | 1.93E-06 | GPX1, GSR, G6PD, ETHE1, GGT1, GSTO1, MGST1, GSTP1 | 178 | 56 | 16792 | 13.47672552 | 0.001967084 | 4.92E-04 | 0.003052423 |
| GOTERM_BP_DIRECT | GO:0010243~response to organonitrogen compound | 5 | 2.604 | 1.10E-05 | MGST3, CDKN1A, ABCC4, MGST1, MGST2 | 178 | 14 | 16792 | 33.6918138 | 0.011178007 | 0.002245665 | 0.017424739 |
| GOTERM_BP_DIRECT | GO:0006805~xenobiotic metabolic process | 8 | 4.167 | 1.81E-05 | MGST3, CYP2C8, GGT1, AOC2, MGST1, GSTP1, CMBL, MGST2 | 178 | 78 | 16792 | 9.67559781 | 0.018292639 | 0.003072274 | 0.028616605 |
| GOTERM_BP_DIRECT | GO:0000302~response to reactive oxygen species | 6 | 3.125 | 5.30E-05 | GPX1, GSR, TXN, PRDX1, GSTP1, SOD3 | 178 | 39 | 16792 | 14.51339672 | 0.052682902 | 0.007701816 | 0.083866382 |
| GOTERM_BP_DIRECT | GO:1901687~glutathione derivative biosynthetic process | 5 | 2.604 | 7.52E-05 | MGST3, GSTO1, MGST1, GSTP1, MGST2 | 178 | 22 | 16792 | 21.44024515 | 0.074022003 | 0.009567043 | 0.119150455 |
| GOTERM_BP_DIRECT | GO:0003333~amino acid transmembrane transport | 5 | 2.604 | 1.27E-04 | SLC38A5, SLC38A2, SLC7A9, SLC6A16, SLC6A19 | 178 | 25 | 16792 | 18.86741573 | 0.121687761 | 0.014313586 | 0.200947305 |
| GOTERM_BP_DIRECT | GO:0009408~response to heat | 6 | 3.125 | 1.46E-04 | HSP90AA1, GCLC, DNAJA1, ABCC2, SST, DNAJA4 | 178 | 48 | 16792 | 11.79213483 | 0.13851636 | 0.014799319 | 0.230873783 |
| GOTERM_BP_DIRECT | GO:0045454~cell redox homeostasis | 7 | 3.646 | 1.61E-04 | GPX1, GSR, GCLC, AIFM1, TXN, TXNRD3, PRDX1 | 178 | 77 | 16792 | 8.576098059 | 0.151541926 | 0.014828469 | 0.254435105 |
| GOTERM_BP_DIRECT | GO:0006559~L-phenylalanine catabolic process | 4 | 2.083 | 1.79E-04 | QDPR, HGD, HPD, FAH | 178 | 11 | 16792 | 34.30439224 | 0.16679399 | 0.015091166 | 0.282480714 |
| GOTERM_BP_DIRECT | GO:0006865~amino acid transport | 5 | 2.604 | 4.84E-04 | SLC38A5, SLC1A4, SLC38A2, SLC7A9, SLC6A19 | 178 | 35 | 16792 | 13.47672552 | 0.390310319 | 0.037346684 | 0.764136078 |
| GOTERM_BP_DIRECT | GO:0006986~response to unfolded protein | 5 | 2.604 | 9.77E-04 | HSPA1L, HSP90AA1, DNAJA1, HSPA4, SERPINH1 | 178 | 42 | 16792 | 11.2306046 | 0.631770906 | 0.068874039 | 1.536843078 |
| GOTERM_BP_DIRECT | GO:0006572~tyrosine catabolic process | 3 | 1.563 | 0.00108201 | HGD, HPD, FAH | 178 | 5 | 16792 | 56.60224719 | 0.669255453 | 0.071105953 | 1.700581954 |
| GOTERM_BP_DIRECT | GO:0006979~response to oxidative stress | 7 | 3.646 | 0.00109154 | GSS, GPX1, GCLC, PON2, ABCC2, PRNP, GCLM | 178 | 110 | 16792 | 6.003268641 | 0.672465474 | 0.067382484 | 1.715443035 |
| GOTERM_BP_DIRECT | GO:0043066~negative regulation of apoptotic process | 14 | 7.292 | 0.00109281 | HTATIP2, GCLC, MMP9, CIAPIN1, MIEN1, DHRS2, CDKN1A, SQSTM1, UCP2, DNAJA1, PRNP, SCX, HIGD1A, GSTP1 | 178 | 455 | 16792 | 2.902679343 | 0.672889737 | 0.063618988 | 1.717417919 |
| GOTERM_BP_DIRECT | GO:0042493~response to drug | 11 | 5.729 | 0.0014664 | XPO1, CDKN1A, HSP90AA1, SLC26A5, ABCC4, SPINK4, TIMP2, SST, PNP, GCLM, MGST1 | 178 | 304 | 16792 | 3.413512714 | 0.77681666 | 0.079943405 | 2.298182135 |
| GOTERM_BP_DIRECT | GO:0007568~aging | 8 | 4.167 | 0.00184834 | GSS, LONP1, GCLC, HAMP, UCP2, FOXG1, TIMP2, GCLM | 178 | 165 | 16792 | 4.573918965 | 0.849041915 | 0.094722108 | 2.88859439 |
| GOTERM_BP_DIRECT | GO:0009636~response to toxic substance | 6 | 3.125 | 0.00204311 | DHRS2, CDKN1A, AIFM1, SLC22A8, PON2, GSTP1 | 178 | 85 | 16792 | 6.659087905 | 0.876337232 | 0.099234077 | 3.188387062 |
| GOTERM_BP_DIRECT | GO:0009410~response to xenobiotic stimulus | 3 | 1.563 | 0.00378848 | GSS, GPX1, GCLC | 178 | 9 | 16792 | 31.44569288 | 0.97933217 | 0.168665226 | 5.836433674 |
| GOTERM_BP_DIRECT | GO:0042572~retinol metabolic process | 4 | 2.083 | 0.00379224 | RDH12, RDH10, DHRS4, BCO1 | 178 | 30 | 16792 | 12.57827715 | 0.979411856 | 0.161802741 | 5.842072632 |
| GOTERM_BP_DIRECT | GO:0034599~cellular response to oxidative stress | 5 | 2.604 | 0.0046358 | DHRS2, GPX1, LONP1, G6PD, NFE2L1 | 178 | 64 | 16792 | 7.37008427 | 0.991337937 | 0.186549067 | 7.097367 |
| GOTERM_BP_DIRECT | GO:0006098~pentose-phosphate shunt | 3 | 1.563 | 0.0057083 | G6PD, TALDO1, PGD | 178 | 11 | 16792 | 25.72829418 | 0.997121806 | 0.216335807 | 8.670712701 |
| GOTERM_BP_DIRECT | GO:0071222~cellular response to lipopolysaccharide | 6 | 3.125 | 0.00691073 | NR1D1, HAMP, SERPINE1, ACOD1, GSTP1, CD180 | 178 | 113 | 16792 | 5.009048424 | 0.999164333 | 0.246850275 | 10.40497614 |
| GOTERM_BP_DIRECT | GO:0006536~glutamate metabolic process | 3 | 1.563 | 0.00798418 | GCLC, GGT1, GCLM | 178 | 13 | 16792 | 21.77009507 | 0.9997233 | 0.270283452 | 11.92709346 |
| GOTERM_BP_DIRECT | GO:0051131~chaperone-mediated protein complex assembly | 3 | 1.563 | 0.00798418 | LONP1, HSP90AA1, HSPA4 | 178 | 13 | 16792 | 21.77009507 | 0.9997233 | 0.270283452 | 11.92709346 |
| GOTERM_BP_DIRECT | GO:0046685~response to arsenic-containing substance | 3 | 1.563 | 0.00798418 | CDKN1A, GCLC, ABCC2 | 178 | 13 | 16792 | 21.77009507 | 0.9997233 | 0.270283452 | 11.92709346 |
| GOTERM_BP_DIRECT | GO:0008152~metabolic process | 7 | 3.646 | 0.00883633 | UGT1A5, TKTL2, GSTO1, ACOD1, PRNP, UGT3A1, GSTP1 | 178 | 168 | 16792 | 3.93071161 | 0.999885033 | 0.285346753 | 13.11813066 |
| GOTERM_BP_DIRECT | GO:0009725~response to hormone | 4 | 2.083 | 0.00977904 | ME1, LONP1, GCLC, TIMP2 | 178 | 42 | 16792 | 8.984483681 | 0.999956527 | 0.301410397 | 14.41816448 |
| GOTERM_BP_DIRECT | GO:0042026~protein refolding | 3 | 1.563 | 0.01060051 | HSPA1L, HSP90AA1, DNAJA4 | 178 | 15 | 16792 | 18.86741573 | 0.999981385 | 0.313102898 | 15.53610643 |
| GOTERM_BP_DIRECT | GO:0008637~apoptotic mitochondrial changes | 3 | 1.563 | 0.01679456 | GCLC, AIFM2, GCLM | 178 | 19 | 16792 | 14.89532821 | 0.99999997 | 0.438415695 | 23.53559043 |
| GOTERM_BP_DIRECT | GO:0007601~visual perception | 7 | 3.646 | 0.01987573 | RDH12, RDH10, CRYGD, CABP4, NR2E3, AOC2, PDE6G | 178 | 201 | 16792 | 3.285370898 | 0.999999999 | 0.484107692 | 27.24504828 |
| GOTERM_BP_DIRECT | GO:0009051~pentose-phosphate shunt, oxidative branch | 2 | 1.042 | 0.02097098 | G6PD, PGD | 178 | 2 | 16792 | 94.33707865 | 1 | 0.491801542 | 28.52251865 |
| GOTERM_BP_DIRECT | GO:0097069~cellular response to thyroxine stimulus | 2 | 1.042 | 0.02097098 | GCLC, GCLM | 178 | 2 | 16792 | 94.33707865 | 1 | 0.491801542 | 28.52251865 |
| GOTERM_BP_DIRECT | GO:0019322~pentose biosynthetic process | 2 | 1.042 | 0.02097098 | G6PD, PGD | 178 | 2 | 16792 | 94.33707865 | 1 | 0.491801542 | 28.52251865 |
| GOTERM_BP_DIRECT | GO:0006090~pyruvate metabolic process | 3 | 1.563 | 0.02222479 | HAGH, SLC16A3, ME1 | 178 | 22 | 16792 | 12.86414709 | 1 | 0.501453784 | 29.95911596 |
| GOTERM_BP_DIRECT | GO:0045742~positive regulation of epidermal growth factor receptor signaling pathway | 3 | 1.563 | 0.02619289 | MMP9, PDE6G, HAP1 | 178 | 24 | 16792 | 11.79213483 | 1 | 0.549691466 | 34.3294448 |
| GOTERM_BP_DIRECT | GO:0006950~response to stress | 4 | 2.083 | 0.02652746 | HSP90AA1, SQSTM1, STIP1, GADD45A | 178 | 61 | 16792 | 6.186037944 | 1 | 0.543908244 | 34.68600069 |
| GOTERM_BP_DIRECT | GO:0034198~cellular response to amino acid starvation | 3 | 1.563 | 0.02827638 | CDKN1A, SLC38A2, UCP2 | 178 | 25 | 16792 | 11.32044944 | 1 | 0.55705194 | 36.52052377 |
| GOTERM_BP_DIRECT | GO:0046686~response to cadmium ion | 3 | 1.563 | 0.02827638 | GSS, GCLC, PRNP | 178 | 25 | 16792 | 11.32044944 | 1 | 0.55705194 | 36.52052377 |
| GOTERM_BP_DIRECT | GO:0032436~positive regulation of proteasomal ubiquitin-dependent protein catabolic process | 4 | 2.083 | 0.03002008 | HSPBP1, GCLC, KEAP1, TRIB2 | 178 | 64 | 16792 | 5.896067416 | 1 | 0.569110858 | 38.30142429 |
| GOTERM_BP_DIRECT | GO:0045604~regulation of epidermal cell differentiation | 2 | 1.042 | 0.03129189 | MAFF, KEAP1 | 178 | 3 | 16792 | 62.89138577 | 1 | 0.574732123 | 39.57073491 |
| GOTERM_BP_DIRECT | GO:0005975~carbohydrate metabolic process | 6 | 3.125 | 0.03722889 | B4GALT1, ME1, TALDO1, UGDH, FUCA2, DHDH | 178 | 174 | 16792 | 3.253002712 | 1 | 0.629984957 | 45.17900532 |
| GOTERM_BP_DIRECT | GO:0071260~cellular response to mechanical stimulus | 4 | 2.083 | 0.0390621 | SLC38A2, GCLC, SCX, GADD45A | 178 | 71 | 16792 | 5.314764994 | 1 | 0.638702074 | 46.80965692 |
| GOTERM_BP_DIRECT | GO:0010951~negative regulation of endopeptidase activity | 5 | 2.604 | 0.03920309 | SERPINB6, SERPINE1, SPINK4, TIMP2, SERPINH1 | 178 | 121 | 16792 | 3.898226391 | 1 | 0.630970717 | 46.93316377 |
| GOTERM_BP_DIRECT | GO:2000379~positive regulation of reactive oxygen species metabolic process | 3 | 1.563 | 0.03962525 | CDKN1A, ACOD1, GADD45A | 178 | 30 | 16792 | 9.433707865 | 1 | 0.626126685 | 47.30138692 |
| GOTERM_BP_DIRECT | GO:0098656~anion transmembrane transport | 3 | 1.563 | 0.03962525 | SLC22A8, ABCC4, ABCC2 | 178 | 30 | 16792 | 9.433707865 | 1 | 0.626126685 | 47.30138692 |
| GOTERM_BP_DIRECT | GO:0043065~positive regulation of apoptotic process | 8 | 4.167 | 0.04007515 | ABR, AIFM2, AIFM1, SQSTM1, SYCE3, DNAJA1, FAM162A, GADD45A | 178 | 300 | 16792 | 2.515655431 | 1 | 0.621710274 | 47.69115854 |

| GOTERM_BP_DIRECT | GO:0006534~cysteine metabolic process | 2 | 1.042 | 0.04150461 | GCLC, GCLM | 178 | 4 | 16792 | 47.16853933 | 1 | 0.626417493 | 48.91175102 |
| --- | --- | --- | --- | --- | --- | --- | --- | --- | --- | --- | --- | --- |
| GOTERM_BP_DIRECT | GO:0051409~response to nitrosative stress | 2 | 1.042 | 0.04150461 | GCLC, GCLM | 178 | 4 | 16792 | 47.16853933 | 1 | 0.626417493 | 48.91175102 |
| GOTERM_BP_DIRECT | GO:0051900~regulation of mitochondrial depolarization | 2 | 1.042 | 0.04150461 | GCLC, GCLM | 178 | 4 | 16792 | 47.16853933 | 1 | 0.626417493 | 48.91175102 |
| GOTERM_BP_DIRECT | GO:0044752~response to human chorionic gonadotropin | 2 | 1.042 | 0.04150461 | GCLC, GCLM | 178 | 4 | 16792 | 47.16853933 | 1 | 0.626417493 | 48.91175102 |
| GOTERM_BP_DIRECT | GO:0010044~response to aluminum ion | 2 | 1.042 | 0.04150461 | LONP1, QDPR | 178 | 4 | 16792 | 47.16853933 | 1 | 0.626417493 | 48.91175102 |
| GOTERM_BP_DIRECT | GO:0015811~L-cystine transport | 2 | 1.042 | 0.04150461 | SLC1A4, SLC7A9 | 178 | 4 | 16792 | 47.16853933 | 1 | 0.626417493 | 48.91175102 |
| GOTERM_BP_DIRECT | GO:0033158~regulation of protein import into nucleus, translocation | 2 | 1.042 | 0.04150461 | CDKN1A, TXN | 178 | 4 | 16792 | 47.16853933 | 1 | 0.626417493 | 48.91175102 |
| GOTERM_BP_DIRECT | GO:0043200~response to amino acid | 3 | 1.563 | 0.0420707 | GSS, SST, GSTP1 | 178 | 31 | 16792 | 9.129394708 | 1 | 0.623242313 | 49.38769878 |
| GOTERM_BP_DIRECT | GO:0007584~response to nutrient | 4 | 2.083 | 0.04331411 | GCLC, SST, SLC6A19, GCLM | 178 | 74 | 16792 | 5.099301549 | 1 | 0.626108554 | 50.41858829 |
| GOTERM_BP_DIRECT | GO:0007623~circadian rhythm | 4 | 2.083 | 0.04478088 | NR1D1, DBP, SERPINE1, PER1 | 178 | 75 | 16792 | 5.031310861 | 1 | 0.630728121 | 51.60935928 |
| GOTERM_BP_DIRECT | GO:0055085~transmembrane transport | 7 | 3.646 | 0.04498182 | SLC16A9, SLC22A14, SLC35B4, ABCC4, ABCC2, ABCC12, ABCA5 | 178 | 244 | 16792 | 2.706391601 | 1 | 0.624668988 | 51.770389 |
| GOTERM_BP_DIRECT | GO:0050728~negative regulation of inflammatory response | 4 | 2.083 | 0.05089155 | ABR, SAA1, ACP5, ACOD1 | 178 | 79 | 16792 | 4.776560944 | 1 | 0.663586638 | 56.28778555 |
| GOTERM_BP_DIRECT | GO:0045040~protein import into mitochondrial outer membrane | 2 | 1.042 | 0.05161027 | HSP90AA1, HSPA4 | 178 | 5 | 16792 | 37.73483146 | 1 | 0.661459133 | 56.80928876 |
| GOTERM_BP_DIRECT | GO:2001237~negative regulation of extrinsic apoptotic signaling pathway | 3 | 1.563 | 0.06065459 | GCLC, GCLM, GSTP1 | 178 | 38 | 16792 | 7.447664104 | 1 | 0.714608753 | 62.89289678 |
| GOTERM_BP_DIRECT | GO:0006520~cellular amino acid metabolic process | 3 | 1.563 | 0.06639509 | GSS, QDPR, GGT1 | 178 | 40 | 16792 | 7.075280899 | 1 | 0.740826417 | 66.32724806 |
| GOTERM_BP_DIRECT | GO:0051262~protein tetramerization | 3 | 1.563 | 0.06639509 | ME1, DHRS4, SLC26A5 | 178 | 40 | 16792 | 7.075280899 | 1 | 0.740826417 | 66.32724806 |
| GOTERM_BP_DIRECT | GO:0031397~negative regulation of protein ubiquitination | 3 | 1.563 | 0.06933071 | GCLC, ISG15, DNAJA1 | 178 | 41 | 16792 | 6.902713072 | 1 | 0.749803773 | 67.96618526 |
| GOTERM_BP_DIRECT | GO:0014823~response to activity | 3 | 1.563 | 0.07532664 | GCLC, MYH4, GCLM | 178 | 43 | 16792 | 6.58165665 | 1 | 0.772857721 | 71.08419268 |
| GOTERM_BP_DIRECT | GO:0032872~regulation of stress-activated MAPK cascade | 2 | 1.042 | 0.08129592 | PRDX1, GSTP1 | 178 | 8 | 16792 | 23.58426966 | 1 | 0.793111428 | 73.90410702 |
| GOTERM_BP_DIRECT | GO:0071493~cellular response to UV-B | 2 | 1.042 | 0.08129592 | CDKN1A, MFAP4 | 178 | 8 | 16792 | 23.58426966 | 1 | 0.793111428 | 73.90410702 |
| GOTERM_BP_DIRECT | GO:0019852~L-ascorbic acid metabolic process | 2 | 1.042 | 0.09098435 | GCLC, GSTO1 | 178 | 9 | 16792 | 20.96379526 | 1 | 0.824641312 | 77.9390593 |
| GOTERM_BP_DIRECT | GO:0034144~negative regulation of toll-like receptor 4 signaling pathway | 2 | 1.042 | 0.09098435 | NR1D1, ACOD1 | 178 | 9 | 16792 | 20.96379526 | 1 | 0.824641312 | 77.9390593 |
| GOTERM_BP_DIRECT | GO:0035914~skeletal muscle cell differentiation | 3 | 1.563 | 0.09422794 | MAFF, ANKRD33, SCX | 178 | 49 | 16792 | 5.775739509 | 1 | 0.830426979 | 79.15375166 |
| GOTERM_BP_DIRECT | GO:0032496~response to lipopolysaccharide | 5 | 2.604 | 0.09553742 | ABR, CXCL9, ACP5, MGST1, MGST2 | 178 | 164 | 16792 | 2.876130447 | 1 | 0.829559668 | 79.62614629 |
