# Supplementary material for: Generation and characterization of keap1a- and keap1b-knockout zebrafish
Source: Redox Biol. 2020 Aug 11;36:101667. doi: 10.1016/j.redox.2020.101667 (PMC7452054; doi:10.1016/j.redox.2020.101667)
Supplement: Multimedia component 3 [file mmc3.docx]

Table S5. Biological processes down-regulated by *keap1a* disruption.

| Category | Term | Count | % | P Value | Genes | List Total | Pop Hits | Pop Total | Fold Enrichment | Bonferroni | Benjamini | FDR |
| --- | --- | --- | --- | --- | --- | --- | --- | --- | --- | --- | --- | --- |
| GOTERM_BP_DIRECT | GO:0002934~desmosome organization | 5 | 1.437 | 6.87E-07 | DSG2, NECTIN1, DSP, PERP, GRHL1 | 327 | 5 | 16792 | 51.35168196 | 0.001461876 | 0.001461876 | 0.001189555 |
| GOTERM_BP_DIRECT | GO:0007623~circadian rhythm | 10 | 2.874 | 1.43E-05 | ATF5, NAMPT, NR1D1, KLF9, DBP, SERPINE1, PER1, NFIL3, NOCT, CLOCK | 327 | 75 | 16792 | 6.846890928 | 0.029993052 | 0.015110693 | 0.02475834 |
| GOTERM_BP_DIRECT | GO:0006366~transcription from RNA polymerase II promoter | 25 | 7.184 | 6.82E-05 | HLF, PTTG1, FOXO3, SOX9, NOCT, FOS, FUBP3, HSF2, TEF, POU3F2, NFIL3, MYC, EGR1, MYF6, SSBP4, BARHL1, KLF15, HMGA2, GRHL1, ATF5, ATF3, DBP, CSRNP1, SHOX, CLOCK | 327 | 513 | 16792 | 2.502518614 | 0.135232402 | 0.047277395 | 0.118073228 |
| GOTERM_BP_DIRECT | GO:0045944~positive regulation of transcription from RNA polymerase II promoter | 38 | 10.92 | 8.45E-05 | HLF, NAMPT, CREM, PPP3R1, FOXO3, SOX9, FOS, FUBP3, HSF2, SERPINE1, TEF, PER1, POU3F2, POU3F1, MYC, EGR1, MYF6, SSBP4, RXRB, BARHL1, NR4A1, SKI, KLF15, GRHL1, HMGA2, LPIN1, STAT3, ATF5, UHRF1, TET3, ATF3, ITGA6, RPS6KA1, DBP, SFRP2, CSRNP1, CLOCK, KDM6B | 327 | 981 | 16792 | 1.989157915 | 0.164637959 | 0.043976244 | 0.146166695 |
| GOTERM_BP_DIRECT | GO:0048147~negative regulation of fibroblast proliferation | 6 | 1.724 | 3.00E-04 | TRIM32, SKI, DACH1, PMAIP1, MYC, TP53INP1 | 327 | 31 | 16792 | 9.939035218 | 0.472340264 | 0.120024074 | 0.51848679 |
| GOTERM_BP_DIRECT | GO:0097186~amelogenesis | 4 | 1.149 | 5.58E-04 | MMP20, ITGA6, ITGB4, PERP | 327 | 9 | 16792 | 22.82296976 | 0.695666814 | 0.17985328 | 0.96265953 |
| GOTERM_BP_DIRECT | GO:0048511~rhythmic process | 7 | 2.011 | 6.03E-04 | CIPC, HLF, CDK5R1, NR1D2, CREM, TEF, SIK1 | 327 | 54 | 16792 | 6.656699513 | 0.723158235 | 0.167625434 | 1.038873605 |
| GOTERM_BP_DIRECT | GO:0010811~positive regulation of cell-substrate adhesion | 6 | 1.724 | 7.93E-04 | ITGA6, SPOCK2, NPNT, RAC1, PTN, NID1 | 327 | 38 | 16792 | 8.108160309 | 0.81558625 | 0.190486376 | 1.365247121 |
| GOTERM_BP_DIRECT | GO:0048333~mesodermal cell differentiation | 4 | 1.149 | 0.00106554 | ITGB4, HMGA2, ITGB1, KDM6B | 327 | 11 | 16792 | 18.67333889 | 0.896771494 | 0.222997918 | 1.829516436 |
| GOTERM_BP_DIRECT | GO:0048870~cell motility | 5 | 1.437 | 0.00109004 | CD34, RAC1, ITGB4, SKI, MMP14 | 327 | 24 | 16792 | 10.69826707 | 0.902025187 | 0.20729528 | 1.871203935 |
| GOTERM_BP_DIRECT | GO:0010629~negative regulation of gene expression | 10 | 2.874 | 0.00143022 | CDKN1A, LDLR, CD34, SFRP2, NDFIP1, PINK1, POU3F2, ACACB, NOCT, TP53INP1 | 327 | 137 | 16792 | 3.748297953 | 0.952572042 | 0.242051858 | 2.448384223 |
| GOTERM_BP_DIRECT | GO:0030198~extracellular matrix organization | 12 | 3.448 | 0.00159747 | COL4A4, MATN3, COL4A2, ITGA6, SPOCK2, ITGAV, NPNT, SERPINE1, ITGB4, NID1, SOX9, ITGB1 | 327 | 196 | 16792 | 3.143980528 | 0.966804362 | 0.247066273 | 2.730989511 |
| GOTERM_BP_DIRECT | GO:0035987~endodermal cell differentiation | 5 | 1.437 | 0.00171976 | COL4A2, ITGAV, MMP9, MMP14, HMGA2 | 327 | 27 | 16792 | 9.509570733 | 0.974427535 | 0.245740187 | 2.937123982 |
| GOTERM_BP_DIRECT | GO:0007602~phototransduction | 5 | 1.437 | 0.00171976 | UNC119, OPN1MW2, PDC, RCVRN, CDS1 | 327 | 27 | 16792 | 9.509570733 | 0.974427535 | 0.245740187 | 2.937123982 |
| GOTERM_BP_DIRECT | GO:0060048~cardiac muscle contraction | 6 | 1.724 | 0.00172887 | TNNT2, SRSF1, ATP1B1, SCN1B, TCAP, MYH6 | 327 | 45 | 16792 | 6.846890928 | 0.974919854 | 0.231460076 | 2.952465426 |
| GOTERM_BP_DIRECT | GO:0007160~cell-matrix adhesion | 8 | 2.299 | 0.00183701 | ITGA6, CD34, ITGAV, NPNT, RAC1, ITGB4, NID1, ITGB1 | 327 | 90 | 16792 | 4.564593952 | 0.980088145 | 0.229793023 | 3.134393119 |
| GOTERM_BP_DIRECT | GO:1902230~negative regulation of intrinsic apoptotic signaling pathway in response to DNA damage | 4 | 1.149 | 0.00225119 | SFRP2, TRIM32, TMEM161A, CXCL12 | 327 | 14 | 16792 | 14.67190913 | 0.991773912 | 0.259202372 | 3.82817527 |
| GOTERM_BP_DIRECT | GO:0035914~skeletal muscle cell differentiation | 6 | 1.724 | 0.00253347 | MYF6, EGR1, HLF, FOS, ATF3, NR4A1 | 327 | 49 | 16792 | 6.287961056 | 0.995497604 | 0.272275031 | 4.298334216 |
| GOTERM_BP_DIRECT | GO:0042493~response to drug | 15 | 4.31 | 0.00256861 | PTGS2, SLC6A11, GGH, ACACB, TIMP2, PNP, KCNJ11, STAT3, ALDH3A1, FOS, CDKN1A, CD86, SFRP2, PTN, MYC | 327 | 304 | 16792 | 2.533800097 | 0.9958231 | 0.262392666 | 4.356711101 |
| GOTERM_BP_DIRECT | GO:0014898~cardiac muscle hypertrophy in response to stress | 4 | 1.149 | 0.00277381 | TCAP, KLF15, MYH6, NPPA | 327 | 15 | 16792 | 13.69378186 | 0.997305178 | 0.267572327 | 4.69692249 |
| GOTERM_BP_DIRECT | GO:0045444~fat cell differentiation | 7 | 2.011 | 0.00292241 | ATF5, TRIM32, SOCS1, GDF10, NR4A1, CLIP3, HMGA2 | 327 | 73 | 16792 | 4.924133886 | 0.998038102 | 0.2677931 | 4.942585568 |
| GOTERM_BP_DIRECT | GO:0046034~ATP metabolic process | 5 | 1.437 | 0.0032656 | ATP1B1, SLC25A25, MYH4, MYH6, GUK1 | 327 | 32 | 16792 | 8.023700306 | 0.999057635 | 0.282345843 | 5.507675127 |
| GOTERM_BP_DIRECT | GO:0007420~brain development | 11 | 3.161 | 0.00408393 | CDK5R1, IRS2, CADM1, SPHK2, PFKFB3, SLC6A11, BAG3, FOXG1, PPP3CC, PHF8, DDIT4 | 327 | 190 | 16792 | 2.972992113 | 0.999836152 | 0.327133221 | 6.842343124 |
| GOTERM_BP_DIRECT | GO:0046718~viral entry into host cell | 7 | 2.011 | 0.0046095 | CD86, LDLR, XPR1, ITGAV, NECTIN1, SCARB2, ITGB1 | 327 | 80 | 16792 | 4.493272171 | 0.999946772 | 0.348101475 | 7.690148212 |
| GOTERM_BP_DIRECT | GO:0031668~cellular response to extracellular stimulus | 4 | 1.149 | 0.00476491 | FOS, CDKN1A, ITGA6, SFRP2 | 327 | 18 | 16792 | 11.41148488 | 0.999961832 | 0.345508099 | 7.939443961 |
| GOTERM_BP_DIRECT | GO:0032922~circadian regulation of gene expression | 6 | 1.724 | 0.00490571 | NAMPT, NR1D1, CREM, PER1, NOCT, CLOCK | 327 | 57 | 16792 | 5.405440206 | 0.999971763 | 0.342292305 | 8.164757876 |
| GOTERM_BP_DIRECT | GO:0007568~aging | 10 | 2.874 | 0.00499696 | FOS, CD86, HAMP, FOXG1, IGFBP1, FOXO3, TIMP2, LOXL2, STAT3, ALDH3A1 | 327 | 165 | 16792 | 3.112223149 | 0.999976773 | 0.336609959 | 8.310501901 |
| GOTERM_BP_DIRECT | GO:2001171~positive regulation of ATP biosynthetic process | 3 | 0.862 | 0.00535271 | PINK1, MYC, STAT3 | 327 | 6 | 16792 | 25.67584098 | 0.999989156 | 0.345185654 | 8.876633653 |
| GOTERM_BP_DIRECT | GO:0001894~tissue homeostasis | 4 | 1.149 | 0.00557785 | CD34, TRIM32, NANOS1, SOX9 | 327 | 19 | 16792 | 10.81088041 | 0.999993304 | 0.346558704 | 9.233203067 |
| GOTERM_BP_DIRECT | GO:0030049~muscle filament sliding | 5 | 1.437 | 0.00612042 | TNNT2, TCAP, MYH4, ACTN2, MYH6 | 327 | 38 | 16792 | 6.756800258 | 0.999997906 | 0.362955227 | 10.08712393 |
| GOTERM_BP_DIRECT | GO:0046426~negative regulation of JAK-STAT cascade | 5 | 1.437 | 0.00735195 | SOCS3, SOCS1, HGS, LEPROT, HMGA2 | 327 | 40 | 16792 | 6.418960245 | 0.999999851 | 0.407803405 | 11.99735867 |
| GOTERM_BP_DIRECT | GO:2001259~positive regulation of cation channel activity | 3 | 0.862 | 0.007398 | ACTN2, CTSS, KCNJ11 | 327 | 7 | 16792 | 22.0078637 | 0.999999865 | 0.399626828 | 12.06804431 |
| GOTERM_BP_DIRECT | GO:0030574~collagen catabolic process | 6 | 1.724 | 0.0080058 | COL4A4, COL4A2, MMP20, MMP9, CTSS, MMP14 | 327 | 64 | 16792 | 4.814220183 | 0.999999963 | 0.414348715 | 12.99594748 |
| GOTERM_BP_DIRECT | GO:0045892~negative regulation of transcription, DNA-templated | 19 | 5.46 | 0.0089354 | MYF6, CDK5R1, CCDC85B, TSG101, CREM, DACH1, HMGA2, SOX9, ATF5, CIPC, NR1D1, NR1D2, SFRP2, FOXG1, PER1, SMURF2, LOXL2, RBM15, CLOCK | 327 | 499 | 16792 | 1.955274463 | 0.999999995 | 0.439727231 | 14.39729455 |
| GOTERM_BP_DIRECT | GO:0034644~cellular response to UV | 5 | 1.437 | 0.01028105 | PTGS2, TMEM161A, PTN, MYC, TP53INP1 | 327 | 44 | 16792 | 5.835418404 | 1 | 0.476600944 | 16.38819909 |
| GOTERM_BP_DIRECT | GO:0030336~negative regulation of cell migration | 7 | 2.011 | 0.01048514 | SFRP2, SERPINE1, PTN, DACH1, CHRD, TP53INP1, THY1 | 327 | 95 | 16792 | 3.783808144 | 1 | 0.473479066 | 16.68631286 |
| GOTERM_BP_DIRECT | GO:0030643~cellular phosphate ion homeostasis | 3 | 0.862 | 0.0123609 | XPR1, GPCPD1, SLC34A2 | 327 | 9 | 16792 | 17.11722732 | 1 | 0.520931213 | 19.37975006 |
| GOTERM_BP_DIRECT | GO:0002053~positive regulation of mesenchymal cell proliferation | 4 | 1.149 | 0.01354386 | IRS2, SOX9, CHRD, MYC | 327 | 26 | 16792 | 7.900258763 | 1 | 0.543887296 | 21.03595787 |
| GOTERM_BP_DIRECT | GO:0042752~regulation of circadian rhythm | 5 | 1.437 | 0.01488401 | NR1D1, NR1D2, CREM, PER1, NOCT | 327 | 49 | 16792 | 5.239967547 | 1 | 0.568530717 | 22.87348974 |
| GOTERM_BP_DIRECT | GO:2001022~positive regulation of response to DNA damage stimulus | 3 | 0.862 | 0.01525458 | HMGA2, PRKCD, MYC | 327 | 10 | 16792 | 15.40550459 | 1 | 0.568097093 | 23.37443342 |
| GOTERM_BP_DIRECT | GO:2000009~negative regulation of protein localization to cell surface | 3 | 0.862 | 0.01525458 | GPM6B, ACTN2, LEPROT | 327 | 10 | 16792 | 15.40550459 | 1 | 0.568097093 | 23.37443342 |
| GOTERM_BP_DIRECT | GO:0022617~extracellular matrix disassembly | 6 | 1.724 | 0.01609148 | MMP20, MMP9, NID1, CTSS, MMP14, TIMP2 | 327 | 76 | 16792 | 4.054080155 | 1 | 0.578459938 | 24.49453097 |
| GOTERM_BP_DIRECT | GO:0030509~BMP signaling pathway | 6 | 1.724 | 0.01609148 | EGR1, BMP3, GDF10, SMURF2, SKI, MYH6 | 327 | 76 | 16792 | 4.054080155 | 1 | 0.578459938 | 24.49453097 |
| GOTERM_BP_DIRECT | GO:0050821~protein stabilization | 8 | 2.299 | 0.01695798 | ATP1B1, CDKN1A, A1CF, BAG3, STXBP1, CHP1, PINK1, PRKCD | 327 | 136 | 16792 | 3.020687174 | 1 | 0.588745577 | 25.63796742 |
| GOTERM_BP_DIRECT | GO:0008286~insulin receptor signaling pathway | 6 | 1.724 | 0.01782578 | PDK2, NAMPT, IRS2, BAIAP2, ATP6V0A1, IGFBP1 | 327 | 78 | 16792 | 3.950129381 | 1 | 0.598350787 | 26.76675189 |
| GOTERM_BP_DIRECT | GO:0045214~sarcomere organization | 4 | 1.149 | 0.01824081 | TCAP, ACTN2, MYH6, ITGB1 | 327 | 29 | 16792 | 7.082990615 | 1 | 0.598239458 | 27.30088076 |
| GOTERM_BP_DIRECT | GO:0046627~negative regulation of insulin receptor signaling pathway | 4 | 1.149 | 0.01824081 | SOCS3, SOCS1, PRKCD, PRKCB | 327 | 29 | 16792 | 7.082990615 | 1 | 0.598239458 | 27.30088076 |
| GOTERM_BP_DIRECT | GO:0001666~response to hypoxia | 9 | 2.586 | 0.01928272 | EGR1, ATP1B1, MMP14, LOXL2, CXCL12, NPPA, PRKCB, DDIT4, ALDH3A1 | 327 | 172 | 16792 | 2.687006614 | 1 | 0.610377199 | 28.62563231 |
| GOTERM_BP_DIRECT | GO:0031581~hemidesmosome assembly | 3 | 0.862 | 0.02180916 | ITGA6, ITGB4, PLEC | 327 | 12 | 16792 | 12.83792049 | 1 | 0.647858924 | 31.74409633 |
| GOTERM_BP_DIRECT | GO:0042981~regulation of apoptotic process | 10 | 2.874 | 0.02352891 | GLS2, EGR1, BMP3, GDF10, ACTN2, GAS1, PMAIP1, PERP, SOX9, TP53INP1 | 327 | 213 | 16792 | 2.410877087 | 1 | 0.667964241 | 33.79290182 |

| GOTERM_BP_DIRECT | GO:0030307~positive regulation of cell growth | 6 | 1.724 | 0.02375537 | ADNP2, RPS6KA1, SFRP2, TRIM32, IGFBP1, MMP14 | 327 | 84 | 16792 | 3.667977283 | 1 | 0.663637758 | 34.05833941 |
| --- | --- | --- | --- | --- | --- | --- | --- | --- | --- | --- | --- | --- |
| GOTERM_BP_DIRECT | GO:0045893~positive regulation of transcription, DNA-templated | 18 | 5.172 | 0.0238035 | ING5, EGR1, FOXO3, HMGA2, SOX9, STAT3, ATF5, FOS, FUBP3, CD86, NR1D1, NR1D2, USP21, POU3F1, MYC, PHF8, CLOCK, TP53INP1 | 327 | 515 | 16792 | 1.794816068 | 1 | 0.656667262 | 34.11462627 |
| GOTERM_BP_DIRECT | GO:0043401~steroid hormone mediated signaling pathway | 5 | 1.437 | 0.0246176 | NR1D1, NR1D2, RXRB, PAQR6, NR4A1 | 327 | 57 | 16792 | 4.504533505 | 1 | 0.661592416 | 35.05980801 |
| GOTERM_BP_DIRECT | GO:0010952~positive regulation of peptidase activity | 3 | 0.862 | 0.0254482 | PINK1, MMP14, CLPX | 327 | 13 | 16792 | 11.85038814 | 1 | 0.66650452 | 36.01096238 |
| GOTERM_BP_DIRECT | GO:0015758~glucose transport | 4 | 1.149 | 0.02573845 | PPBP, SLC2A2, SLC2A1, KLF15 | 327 | 33 | 16792 | 6.224446298 | 1 | 0.663458763 | 36.34023287 |
| GOTERM_BP_DIRECT | GO:0006357~regulation of transcription from RNA polymerase II promoter | 16 | 4.598 | 0.02588003 | MYF6, KLF9, BARHL1, ZNF76, FOXO3, LPIN1, STAT3, PRKCB, ATF5, FOS, ATF3, DBP, HSF2, FOXG1, TEF, CLOCK | 327 | 441 | 16792 | 1.863099572 | 1 | 0.658376045 | 36.5002705 |
| GOTERM_BP_DIRECT | GO:0008283~cell proliferation | 14 | 4.023 | 0.02687184 | CDK5R1, IRS2, SPHK2, SKI, DACH1, STAT3, TACC1, DDIT4, ZFP36L1, UHRF1, CD34, NAA60, RAC1, MYC | 327 | 366 | 16792 | 1.964271987 | 1 | 0.665366383 | 37.61077866 |
| GOTERM_BP_DIRECT | GO:0007165~signal transduction | 33 | 9.483 | 0.02906942 | CHKA, NAMPT, MPZL1, SPOCK2, CREM, PPP2R5D, HBS1L, FST, CXCL9, RCVRN, CABP5, SOX9, FAM13A, CXCL12, FGFBP1, IRS2, GUCA1C, NECTIN1, NDFIP1, NR4A1, CDS1, HMGA2, PRKCD, STAT3, PRKCB, GNAL, RPS6KA1, GRIA2, CD34, HGS, IGFBP1, CLOCK, SH3GL2 | 327 | 1161 | 16792 | 1.459608531 | 1 | 0.687647604 | 40.00651525 |
| GOTERM_BP_DIRECT | GO:0022011~myelination in peripheral nervous system | 3 | 0.862 | 0.02931437 | SKI, POU3F2, POU3F1 | 327 | 14 | 16792 | 11.00393185 | 1 | 0.684071002 | 40.26811057 |
| GOTERM_BP_DIRECT | GO:0043065~positive regulation of apoptotic process | 12 | 3.448 | 0.03295501 | ING5, ITGA6, PTGS2, SFRP2, RAC1, NR4A1, PTN, CLIP3, FOXO3, PMAIP1, HMGA2, ITGB1 | 327 | 300 | 16792 | 2.054067278 | 1 | 0.720453537 | 44.03172187 |
| GOTERM_BP_DIRECT | GO:0033627~cell adhesion mediated by integrin | 3 | 0.862 | 0.03339753 | ITGA6, ITGAV, ITGB1 | 327 | 15 | 16792 | 10.27033639 | 1 | 0.718979924 | 44.47364049 |
| GOTERM_BP_DIRECT | GO:0019886~antigen processing and presentation of exogenous peptide antigen via MHC class II | 6 | 1.724 | 0.03344948 | AP1M1, SEC31A, KLC1, SPTBN2, CTSS, SH3GL2 | 327 | 92 | 16792 | 3.349022736 | 1 | 0.713328383 | 44.5253025 |
| GOTERM_BP_DIRECT | GO:0034332~adherens junction organization | 4 | 1.149 | 0.034647 | RAB8B, CADM1, NECTIN1, DSP | 327 | 37 | 16792 | 5.551533185 | 1 | 0.720009047 | 45.70374711 |
| GOTERM_BP_DIRECT | GO:0007155~cell adhesion | 16 | 4.598 | 0.03517885 | PCDHA8, ATP1B1, SCN1B, NECTIN1, ITGB4, CD99, ACTN2, CXCL12, THY1, ITGA6, DSG2, CD34, ITGAV, RAC1, SSX2IP, LOXL2 | 327 | 459 | 16792 | 1.790036844 | 1 | 0.719545799 | 46.21951625 |
| GOTERM_BP_DIRECT | GO:0007411~axon guidance | 8 | 2.299 | 0.03577607 | CDK5R1, SCN1B, EFNA1, RAC1, SPTBN2, NECTIN1, SPTBN1, CXCL12 | 327 | 159 | 16792 | 2.583732426 | 1 | 0.719764252 | 46.79318535 |
| GOTERM_BP_DIRECT | GO:0030522~intracellular receptor signaling pathway | 4 | 1.149 | 0.03709214 | NR1D1, NR1D2, NR4A1, STAT3 | 327 | 38 | 16792 | 5.405440206 | 1 | 0.727066974 | 48.03705817 |
| GOTERM_BP_DIRECT | GO:0008217~regulation of blood pressure | 5 | 1.437 | 0.03745985 | PTGS2, CD34, MYH6, NPPA, GCH1 | 327 | 65 | 16792 | 3.950129381 | 1 | 0.724957811 | 48.3796695 |
| GOTERM_BP_DIRECT | GO:0030182~neuron differentiation | 6 | 1.724 | 0.03763439 | CDK5R1, ADNP2, POU3F2, RTN1, NRBP2, DDIT4 | 327 | 95 | 16792 | 3.243264124 | 1 | 0.721042841 | 48.54154902 |
| GOTERM_BP_DIRECT | GO:0035994~response to muscle stretch | 3 | 0.862 | 0.03768786 | FOS, TCAP, NPPA | 327 | 16 | 16792 | 9.628440367 | 1 | 0.71602703 | 48.59104238 |
| GOTERM_BP_DIRECT | GO:0071456~cellular response to hypoxia | 6 | 1.724 | 0.03909743 | ZFP36L1, PTGS2, PINK1, PTN, FOXO3, PMAIP1 | 327 | 96 | 16792 | 3.209480122 | 1 | 0.723932344 | 49.87978286 |
| GOTERM_BP_DIRECT | GO:0008284~positive regulation of cell proliferation | 16 | 4.598 | 0.03946364 | NAMPT, IRS2, SPHK2, SOX9, ITGB1, STAT3, ALDH3A1, CD86, ATF3, EPGN, SFRP2, ITGAV, PTN, POU3F2, FGFBP1, MYC | 327 | 466 | 16792 | 1.763147878 | 1 | 0.72196736 | 50.20957938 |
| GOTERM_BP_DIRECT | GO:0045668~negative regulation of osteoblast differentiation | 4 | 1.149 | 0.03962336 | GDF10, SKI, CHRD, NOCT | 327 | 39 | 16792 | 5.266839175 | 1 | 0.718156148 | 50.35278174 |
| GOTERM_BP_DIRECT | GO:0006006~glucose metabolic process | 5 | 1.437 | 0.04117004 | PDK2, IRS2, CREM, MYC, KCNJ11 | 327 | 67 | 16792 | 3.832215071 | 1 | 0.726868954 | 51.71954481 |
| GOTERM_BP_DIRECT | GO:0050796~regulation of insulin secretion | 5 | 1.437 | 0.04117004 | SLC2A2, SLC2A1, MARCKS, KCNJ11, CLOCK | 327 | 67 | 16792 | 3.832215071 | 1 | 0.726868954 | 51.71954481 |
| GOTERM_BP_DIRECT | GO:0031589~cell-substrate adhesion | 3 | 0.862 | 0.04217583 | ITGA6, ITGAV, ITGB1 | 327 | 17 | 16792 | 9.062061522 | 1 | 0.730503316 | 52.58923192 |
| GOTERM_BP_DIRECT | GO:0007601~visual perception | 9 | 2.586 | 0.04282215 | UNC119, DHRS3, GUCA1C, KERA, PITPNA, OPN1MW2, PDC, RCVRN, OAT | 327 | 201 | 16792 | 2.299329043 | 1 | 0.73098332 | 53.14027984 |
| GOTERM_BP_DIRECT | GO:0001935~endothelial cell proliferation | 3 | 0.862 | 0.04685219 | CD34, MMP14, LOXL2 | 327 | 18 | 16792 | 8.55861366 | 1 | 0.758180773 | 56.4424504 |
| GOTERM_BP_DIRECT | GO:0016337~single organismal cell-cell adhesion | 6 | 1.724 | 0.04692858 | ITGA6, CD34, NECTIN1, DSP, SOX9, THY1 | 327 | 101 | 16792 | 3.050594968 | 1 | 0.754008214 | 56.50286783 |
| GOTERM_BP_DIRECT | GO:0071260~cellular response to mechanical stimulus | 5 | 1.437 | 0.04919286 | PTGS2, BAG3, RAC1, SOX9, NPPA | 327 | 71 | 16792 | 3.616315631 | 1 | 0.765891399 | 58.25838785 |
| GOTERM_BP_DIRECT | GO:0014823~response to activity | 4 | 1.149 | 0.05059398 | SLC25A25, MYH4, PTN, KDM6B | 327 | 43 | 16792 | 4.776900647 | 1 | 0.771105042 | 59.31102163 |
| GOTERM_BP_DIRECT | GO:0043525~positive regulation of neuron apoptotic process | 4 | 1.149 | 0.05059398 | CDK5R1, RAPSN, FOXO3, PMAIP1 | 327 | 43 | 16792 | 4.776900647 | 1 | 0.771105042 | 59.31102163 |
| GOTERM_BP_DIRECT | GO:0001501~skeletal system development | 7 | 2.011 | 0.05103694 | BMP3, MATN3, MMP9, SHOX, GDF10, SOX9, CHRD | 327 | 137 | 16792 | 2.623808567 | 1 | 0.769653363 | 59.63856874 |
| GOTERM_BP_DIRECT | GO:1904659~glucose transmembrane transport | 3 | 0.862 | 0.051708 | PPBP, SLC2A2, SLC2A1 | 327 | 19 | 16792 | 8.108160309 | 1 | 0.769768761 | 60.13005377 |
| GOTERM_BP_DIRECT | GO:0001764~neuron migration | 6 | 1.724 | 0.05381717 | CDK5R1, GPM6A, MAPT, BARHL1, CXCL12, DDIT4 | 327 | 105 | 16792 | 2.934381826 | 1 | 0.779232886 | 61.63836378 |
| GOTERM_BP_DIRECT | GO:0030514~negative regulation of BMP signaling pathway | 4 | 1.149 | 0.0565743 | RBPMS2, SFRP2, SKI, CHRD | 327 | 45 | 16792 | 4.564593952 | 1 | 0.791997573 | 63.5290635 |
| GOTERM_BP_DIRECT | GO:0072531~pyrimidine-containing compound transmembrane transport | 2 | 0.575 | 0.05712196 | AQP9, SLC28A1 | 327 | 3 | 16792 | 34.23445464 | 1 | 0.791128962 | 63.89401195 |
| GOTERM_BP_DIRECT | GO:0015855~pyrimidine nucleobase transport | 2 | 0.575 | 0.05712196 | AQP9, SLC28A1 | 327 | 3 | 16792 | 34.23445464 | 1 | 0.791128962 | 63.89401195 |
| GOTERM_BP_DIRECT | GO:0060221~retinal rod cell differentiation | 2 | 0.575 | 0.05712196 | PTN, SOX9 | 327 | 3 | 16792 | 34.23445464 | 1 | 0.791128962 | 63.89401195 |
| GOTERM_BP_DIRECT | GO:0042512~negative regulation of tyrosine phosphorylation of Stat1 protein | 2 | 0.575 | 0.05712196 | SOCS3, SOCS1 | 327 | 3 | 16792 | 34.23445464 | 1 | 0.791128962 | 63.89401195 |
| GOTERM_BP_DIRECT | GO:1904823~purine nucleobase transmembrane transport | 2 | 0.575 | 0.05712196 | AQP9, SLC28A1 | 327 | 3 | 16792 | 34.23445464 | 1 | 0.791128962 | 63.89401195 |
| GOTERM_BP_DIRECT | GO:0051591~response to cAMP | 4 | 1.149 | 0.05968468 | FOS, CREM, PER1, ALDH3A1 | 327 | 46 | 16792 | 4.465363648 | 1 | 0.80175954 | 65.55650129 |
| GOTERM_BP_DIRECT | GO:0032956~regulation of actin cytoskeleton organization | 4 | 1.149 | 0.06287373 | CDK5R1, BAIAP2, GPM6B, PRKCD | 327 | 47 | 16792 | 4.370355911 | 1 | 0.814886189 | 67.52464069 |
| GOTERM_BP_DIRECT | GO:0010165~response to X-ray | 3 | 0.862 | 0.06726656 | CDKN1A, BRCC3, PMAIP1 | 327 | 22 | 16792 | 7.002502085 | 1 | 0.832544851 | 70.06273538 |
| GOTERM_BP_DIRECT | GO:0046329~negative regulation of JNK cascade | 3 | 0.862 | 0.06726656 | TAOK3, PER1, PINK1 | 327 | 22 | 16792 | 7.002502085 | 1 | 0.832544851 | 70.06273538 |
| GOTERM_BP_DIRECT | GO:0034113~heterotypic cell-cell adhesion | 3 | 0.862 | 0.06726656 | ITGAV, PERP, ITGB1 | 327 | 22 | 16792 | 7.002502085 | 1 | 0.832544851 | 70.06273538 |
| GOTERM_BP_DIRECT | GO:0035264~multicellular organism growth | 5 | 1.437 | 0.07014714 | ZFP36L1, ATF5, SLC25A25, SPTBN2, ADD1 | 327 | 80 | 16792 | 3.209480122 | 1 | 0.841847817 | 71.62431108 |
| GOTERM_BP_DIRECT | GO:0046835~carbohydrate phosphorylation | 3 | 0.862 | 0.07275594 | PFKFB4, PFKFB3, NAGK | 327 | 23 | 16792 | 6.698045473 | 1 | 0.849366444 | 72.97202732 |
| GOTERM_BP_DIRECT | GO:0070885~negative regulation of calcineurin-NFAT signaling cascade | 2 | 0.575 | 0.07543027 | CHP1, PRNP | 327 | 4 | 16792 | 25.67584098 | 1 | 0.856645035 | 74.29083897 |
| GOTERM_BP_DIRECT | GO:0015837~amine transport | 2 | 0.575 | 0.07543027 | RHCG, AQP9 | 327 | 4 | 16792 | 25.67584098 | 1 | 0.856645035 | 74.29083897 |
| GOTERM_BP_DIRECT | GO:0010499~proteasomal ubiquitin-independent protein catabolic process | 2 | 0.575 | 0.07543027 | ENC1, KEAP1 | 327 | 4 | 16792 | 25.67584098 | 1 | 0.856645035 | 74.29083897 |
| GOTERM_BP_DIRECT | GO:0046040~IMP metabolic process | 2 | 0.575 | 0.07543027 | NT5C2, HPRT1 | 327 | 4 | 16792 | 25.67584098 | 1 | 0.856645035 | 74.29083897 |
| GOTERM_BP_DIRECT | GO:0033631~cell-cell adhesion mediated by integrin | 2 | 0.575 | 0.07543027 | NPNT, ITGB1 | 327 | 4 | 16792 | 25.67584098 | 1 | 0.856645035 | 74.29083897 |
| GOTERM_BP_DIRECT | GO:0071322~cellular response to carbohydrate stimulus | 2 | 0.575 | 0.07543027 | MYC, PRKCB | 327 | 4 | 16792 | 25.67584098 | 1 | 0.856645035 | 74.29083897 |

| GOTERM_BP_DIRECT | GO:0006655~phosphatidylglycerol biosynthetic process | 2 | 0.575 | 0.07543027 | CDS2, CDS1 | 327 | 4 | 16792 | 25.67584098 | 1 | 0.856645035 | 74.29083897 |
| --- | --- | --- | --- | --- | --- | --- | --- | --- | --- | --- | --- | --- |
| GOTERM_BP_DIRECT | GO:0070837~dehydroascorbic acid transport | 2 | 0.575 | 0.07543027 | SLC2A2, SLC2A1 | 327 | 4 | 16792 | 25.67584098 | 1 | 0.856645035 | 74.29083897 |
| GOTERM_BP_DIRECT | GO:0070371~ERK1 and ERK2 cascade | 3 | 0.862 | 0.07838396 | ZFP36L1, ITGAV, SOX9 | 327 | 24 | 16792 | 6.418960245 | 1 | 0.864453953 | 75.67685007 |
| GOTERM_BP_DIRECT | GO:0015914~phospholipid transport | 3 | 0.862 | 0.08414319 | LDLR, PITPNA, TMEM30A | 327 | 25 | 16792 | 6.162201835 | 1 | 0.88086102 | 78.17933235 |
| GOTERM_BP_DIRECT | GO:2001235~positive regulation of apoptotic signaling pathway | 3 | 0.862 | 0.08414319 | ING5, PRKCD, TP53INP1 | 327 | 25 | 16792 | 6.162201835 | 1 | 0.88086102 | 78.17933235 |
| GOTERM_BP_DIRECT | GO:0034612~response to tumor necrosis factor | 3 | 0.862 | 0.08414319 | PTGS2, TRIM32, GCH1 | 327 | 25 | 16792 | 6.162201835 | 1 | 0.88086102 | 78.17933235 |
| GOTERM_BP_DIRECT | GO:0048013~ephrin receptor signaling pathway | 5 | 1.437 | 0.08627806 | CDK5R1, EFNA1, MMP9, RAC1, DNM1 | 327 | 86 | 16792 | 2.985562904 | 1 | 0.884607116 | 79.04372125 |
| GOTERM_BP_DIRECT | GO:0050900~leukocyte migration | 6 | 1.724 | 0.08925636 | ATP1B1, ITGA6, CD34, ITGAV, MMP9, ITGB1 | 327 | 122 | 16792 | 2.525492555 | 1 | 0.890593742 | 80.19583312 |
| GOTERM_BP_DIRECT | GO:0019432~triglyceride biosynthetic process | 3 | 0.862 | 0.09002647 | GPD1L, DGAT2, LPIN1 | 327 | 26 | 16792 | 5.925194072 | 1 | 0.890098698 | 80.48387667 |
| GOTERM_BP_DIRECT | GO:0019216~regulation of lipid metabolic process | 3 | 0.862 | 0.09002647 | IRS2, NR1D1, NR1D2 | 327 | 26 | 16792 | 5.925194072 | 1 | 0.890098698 | 80.48387667 |
| GOTERM_BP_DIRECT | GO:0008652~cellular amino acid biosynthetic process | 3 | 0.862 | 0.09002647 | GLS2, OAT, GPT2 | 327 | 26 | 16792 | 5.925194072 | 1 | 0.890098698 | 80.48387667 |
| GOTERM_BP_DIRECT | GO:0071557~histone H3-K27 demethylation | 2 | 0.575 | 0.09338415 | PHF8, KDM6B | 327 | 5 | 16792 | 20.54067278 | 1 | 0.896662731 | 81.69424259 |
| GOTERM_BP_DIRECT | GO:0042634~regulation of hair cycle | 2 | 0.575 | 0.09338415 | PER1, CLOCK | 327 | 5 | 16792 | 20.54067278 | 1 | 0.896662731 | 81.69424259 |
| GOTERM_BP_DIRECT | GO:0010807~regulation of synaptic vesicle priming | 2 | 0.575 | 0.09338415 | STXBP1, NAPB | 327 | 5 | 16792 | 20.54067278 | 1 | 0.896662731 | 81.69424259 |
| GOTERM_BP_DIRECT | GO:1901727~positive regulation of histone deacetylase activity | 2 | 0.575 | 0.09338415 | PINK1, C6ORF89 | 327 | 5 | 16792 | 20.54067278 | 1 | 0.896662731 | 81.69424259 |
| GOTERM_BP_DIRECT | GO:0071504~cellular response to heparin | 2 | 0.575 | 0.09338415 | EGR1, SOX9 | 327 | 5 | 16792 | 20.54067278 | 1 | 0.896662731 | 81.69424259 |
| GOTERM_BP_DIRECT | GO:0006003~fructose 2,6-bisphosphate metabolic process | 2 | 0.575 | 0.09338415 | PFKFB4, PFKFB3 | 327 | 5 | 16792 | 20.54067278 | 1 | 0.896662731 | 81.69424259 |
| GOTERM_BP_DIRECT | GO:0071447~cellular response to hydroperoxide | 2 | 0.575 | 0.09338415 | PRKCD, TP53INP1 | 327 | 5 | 16792 | 20.54067278 | 1 | 0.896662731 | 81.69424259 |
| GOTERM_BP_DIRECT | GO:1904385~cellular response to angiotensin | 2 | 0.575 | 0.09338415 | PRKCD, MYC | 327 | 5 | 16792 | 20.54067278 | 1 | 0.896662731 | 81.69424259 |
| GOTERM_BP_DIRECT | GO:0010917~negative regulation of mitochondrial membrane potential | 2 | 0.575 | 0.09338415 | HEBP2, PMAIP1 | 327 | 5 | 16792 | 20.54067278 | 1 | 0.896662731 | 81.69424259 |
| GOTERM_BP_DIRECT | GO:0010310~regulation of hydrogen peroxide metabolic process | 2 | 0.575 | 0.09338415 | RAC1, PINK1 | 327 | 5 | 16792 | 20.54067278 | 1 | 0.896662731 | 81.69424259 |
| GOTERM_BP_DIRECT | GO:0010976~positive regulation of neuron projection development | 5 | 1.437 | 0.09496157 | SCN1B, ENC1, PTN, GPRC5B, TMEM30A | 327 | 89 | 16792 | 2.884925953 | 1 | 0.898249867 | 82.23811222 |
| GOTERM_BP_DIRECT | GO:0055007~cardiac muscle cell differentiation | 3 | 0.862 | 0.09602684 | SIK1, ITGB1, KDM6B | 327 | 27 | 16792 | 5.70574244 | 1 | 0.898491331 | 82.59674729 |
| GOTERM_BP_DIRECT | GO:0072332~intrinsic apoptotic signaling pathway by p53 class mediator | 3 | 0.862 | 0.09602684 | PDK2, PMAIP1, PERP | 327 | 27 | 16792 | 5.70574244 | 1 | 0.898491331 | 82.59674729 |
| GOTERM_BP_DIRECT | GO:0060349~bone morphogenesis | 3 | 0.862 | 0.09602684 | DHRS3, SFRP2, SKI | 327 | 27 | 16792 | 5.70574244 | 1 | 0.898491331 | 82.59674729 |
