# Supplementary material for: Generation and characterization of keap1a- and keap1b-knockout zebrafish
Source: Redox Biol. 2020 Aug 11;36:101667. doi: 10.1016/j.redox.2020.101667 (PMC7452054; doi:10.1016/j.redox.2020.101667)
Supplement: Multimedia component 4 [file mmc4.docx]

Table S6. Biological processes down-regulated by *keap1b* disruption.

| Category | Term | Count | % | P Value | Genes | List Total | Pop Hits | Pop Total | Fold Enrichment | Bonferroni | Benjamini | FDR |
| --- | --- | --- | --- | --- | --- | --- | --- | --- | --- | --- | --- | --- |
| GOTERM_BP_DIRECT | GO:0045944~positive regulation of transcription from RNA polymerase II promoter | 85 | 10.38 | 1.33E-08 | HLF, NAMPT, ELF1, PPARG, ATP1B4, CTCF, ANKRD1, FOXO3, ACVR1B, FOS, FUBP3, SIN3A, RAD21, HSF2, SERPINE1, CREB3L2, POU4F2, CREB3L3, YAP1, RARB, MYC, SSBP4, SOX10, RXRB, BARHL1, STRN3, GRHL1, LPIN1, PROX1, UHRF1, HIF1A, NCOA2, NCOA3, NCK1, MAPK3, CLOCK, KDM6B, FGFR2, TFAP4, CREM, SOX2, PPP3R1, HCFC1, SOX9, TCF7L2, RGMA, TNFRSF1A, MEIS2, SQSTM1, TEF, PER1, POU3F3, POU3F2, PPP3CA, POU3F1, TCF3, PIK3R1, FOXD3, MAF, MYF6, MAFF, HCLS1, GRIN1, ASXL1, ESRRG, NR4A1, SKI, KLF15, STAT3, ATF5, TET3, ATF3, RPS6KA1, ITGA6, DBP, SFRP2, GSK3B, SP3, CSRNP1, PPRC1, MAFA, RBM14, TCF12, RERE, RNF111 | 765 | 981 | 16792 | 1.901914147 | 4.78E-05 | 4.78E-05 | 2.45E-05 |
| GOTERM_BP_DIRECT | GO:0007155~cell adhesion | 49 | 5.983 | 6.88E-08 | MTSS1, PCDHA8, ATP1B1, CD151, CXCL12, ARHGAP5, COL12A1, ZYX, LOXL2, TM9SF4, KIRREL2, MAGI1, PTPRF, INPPL1, FLOT2, NECTIN1, ACTN2, THY1, NCAM1, CD34, ANOS1, VCAN, LAMC1, CHL1, ACHE, SCN1B, ITGB4, PTK7, CTNND1, APLP1, RGMB, ITGAV, RAC1, COL6A2, COL6A1, SSX2IP, THBS1, APC, COL18A1, B4GALT1, CD99, PCDH17, MCAM, PRPH2, COL5A1, ITGA6, DSG2, ATP2A2, CDH17 | 765 | 459 | 16792 | 2.343281074 | 2.46E-04 | 1.23E-04 | 1.26E-04 |
| GOTERM_BP_DIRECT | GO:0042493~response to drug | 34 | 4.151 | 3.48E-06 | PTGS2, SRP68, PPARG, ABCA1, TIMP2, PNP, PTEN, KCNJ11, GCLM, ALDH3A1, FOS, SLC1A2, SLC1A3, PTN, THBS1, MYC, NPFF, COL18A1, SLC6A11, MAOB, GGH, DPYSL2, ACACB, CPT1A, STAT3, TJP1, CD86, CDKN1A, NPC1, SLC26A5, SFRP1, SFRP2, ABCC4, ABL1 | 765 | 304 | 16792 | 2.45497076 | 0.012385792 | 0.004145761 | 0.006393481 |
| GOTERM_BP_DIRECT | GO:0006897~endocytosis | 21 | 2.564 | 4.83E-06 | UNC119, SNX18, RAB7A, SNAP91, CALY, LDLR, INPPL1, SNX8, BCL2L1, DPYSL2, RAB1A, APLP1, AMPH, SYP, CDC42, NPC1, LRP12, DNM1, EPN1, EPN2, SH3GL1 | 765 | 139 | 16792 | 3.316236423 | 0.017134423 | 0.004311408 | 0.008865876 |
| GOTERM_BP_DIRECT | GO:0007623~circadian rhythm | 15 | 1.832 | 6.13E-06 | NAMPT, KLF9, PROX1, NOCT, CPT1A, ATF5, CRY2, NR1D1, DBP, GSK3B, SERPINE1, DYRK1A, PER1, NFIL3, CLOCK | 765 | 75 | 16792 | 4.390065359 | 0.02169844 | 0.004377852 | 0.011253378 |
| GOTERM_BP_DIRECT | GO:0002934~desmosome organization | 5 | 0.611 | 2.05E-05 | DSG2, NECTIN1, DSP, PERP, GRHL1 | 765 | 5 | 16792 | 21.9503268 | 0.070727393 | 0.012151096 | 0.037623651 |
| GOTERM_BP_DIRECT | GO:0030574~collagen catabolic process | 13 | 1.587 | 2.68E-05 | COL18A1, COL4A4, COL4A2, COL4A1, MMP9, CTSS, MMP14, COL5A1, MMP20, COL6A2,  COL12A1, COL6A1, COL11A1 | 765 | 64 | 16792 | 4.458660131 | 0.091410618 | 0.013601229 | 0.049165865 |
| GOTERM_BP_DIRECT | GO:0051017~actin filament bundle assembly | 9 | 1.099 | 4.24E-05 | CDC42, EZR, MYO1B, BAIAP2, FSCN1, DPYSL3, PLS3, ADD2, ADD1 | 765 | 30 | 16792 | 6.585098039 | 0.140901247 | 0.018804865 | 0.077880863 |
| GOTERM_BP_DIRECT | GO:0030198~extracellular matrix organization | 23 | 2.808 | 8.76E-05 | RECK, COL4A4, B4GALT1, COL18A1, COL4A2, COL4A1, SPOCK2, NPNT, ITGB4, NID1, SOX9, VIT, ITGB1, COL5A1, ITGA6, ITGAV, SERPINE1, COL6A2, COL6A1, VCAN, LAMC1, THBS1, COL11A1 | 765 | 196 | 16792 | 2.575803655 | 0.269217704 | 0.034248625 | 0.160770212 |
| GOTERM_BP_DIRECT | GO:0030336~negative regulation of cell migration | 15 | 1.832 | 9.63E-05 | RECK, DPYSL3, DACH1, ARID2, PTEN, KANK1, THY1, SFRP1, SFRP2, SERPINE1, PTN, CNN2, CHRD, DPEP1, TP53INP1 | 765 | 95 | 16792 | 3.465841073 | 0.291643991 | 0.033893157 | 0.176733062 |
| GOTERM_BP_DIRECT | GO:0048511~rhythmic process | 11 | 1.343 | 1.39E-04 | CIPC, HLF, CDK5R1, SIN3A, NR1D2, CREM, PPARG, PSPC1, TEF, PRKAA1, SIK1 | 765 | 54 | 16792 | 4.471362866 | 0.392152009 | 0.044248477 | 0.25506503 |
| GOTERM_BP_DIRECT | GO:0006006~glucose metabolic process | 12 | 1.465 | 2.01E-04 | PGM2, PDK2, IRS2, G6PD, INPPL1, CREM, PRKAA1, MYC, KCNJ11, FABP5, CPT1A, AKT2 | 765 | 67 | 16792 | 3.931401814 | 0.51309648 | 0.058211081 | 0.368525453 |
| GOTERM_BP_DIRECT | GO:0032922~circadian regulation of gene expression | 11 | 1.343 | 2.22E-04 | NAMPT, NCOA2, CRY2, NR1D1, CREM, CIART, PER1, PER3, NOCT, CLOCK, HNRNPU | 765 | 57 | 16792 | 4.236027978 | 0.548121156 | 0.059273818 | 0.406674013 |
| GOTERM_BP_DIRECT | GO:0035264~multicellular organism growth | 13 | 1.587 | 2.53E-04 | FGFR2, PDE4D, ZFP36L1, TAL2, ATF5, CDC42, SLC4A10, SLC1A2, STK40, SLC25A25, SPTBN2, RARB, ADD1 | 765 | 80 | 16792 | 3.566928105 | 0.594981269 | 0.062518928 | 0.462594392 |
| GOTERM_BP_DIRECT | GO:0042752~regulation of circadian rhythm | 10 | 1.221 | 3.18E-04 | CRY2, NR1D1, NR1D2, CREM, PPARG, PSPC1, PER1, PRKAA1, PROX1, NOCT | 765 | 49 | 16792 | 4.47965853 | 0.680028002 | 0.073154246 | 0.582878145 |
| GOTERM_BP_DIRECT | GO:0007411~axon guidance | 19 | 2.32 | 3.36E-04 | CDK5R1, SCN1B, EFNA1, NECTIN1, DPYSL5, DPYSL2, NRXN1, CXCL12, NCAM1, NRAS, EZR, SEMA3F, RAC1, MAPK3, SPTBN2, SPTBN1, ANOS1, POU4F2, CHL1 | 765 | 159 | 16792 | 2.622995026 | 0.700003459 | 0.072487512 | 0.615749736 |
| GOTERM_BP_DIRECT | GO:0045444~fat cell differentiation | 12 | 1.465 | 4.35E-04 | ATF5, GSK3B, TMEM120B, SOCS1, TRIM32, BSCL2, GDF10, NR4A1, CLIP3, SDF4, TCF7L2, AKT2 | 765 | 73 | 16792 | 3.608272898 | 0.789066512 | 0.087476926 | 0.795170687 |
| GOTERM_BP_DIRECT | GO:0048870~cell motility | 7 | 0.855 | 5.79E-04 | PLD1, CD34, FSCN1, RAC1, ITGB4, SKI, MMP14 | 765 | 24 | 16792 | 6.402178649 | 0.874024349 | 0.108716385 | 1.057153475 |
| GOTERM_BP_DIRECT | GO:0098609~cell-cell adhesion | 26 | 3.175 | 6.56E-04 | CNN3, SNX5, SNX1, ASAP1, HCFC1, RAB1A, PAK2, BAG3, CNN2, PLEC, COBLL1, MYO1B, BAIAP2, FSCN1, DOCK9, EIF4G2, TJP1, KRT18, EVPL, SERBP1, SPTBN2, SPTBN1, PERP, ADD1, SH3GL1, EPN2 | 765 | 271 | 16792 | 2.105935412 | 0.904403638 | 0.11623048 | 1.19712428 |
| GOTERM_BP_DIRECT | GO:0006366~transcription from RNA polymerase II promoter | 41 | 5.006 | 6.58E-04 | HLF, ELF1, TFAP4, SOX2, HCFC1, CTCF, FOXO3, PTTG1, SOX9, NOCT, POLR2A, FOS, FUBP3, RAD21, MEIS2, HSF2, CREB3L2, TEF, POU4F2, POU3F2, CREB3L3, NFIL3, MYC, MYF6, MAF, MAFF, SOX10, SSBP4, PHRF1, BARHL1, KLF15, GRHL1, ATF5, ATF3, HIF1A, DBP, CSRNP1, SHOX, MAFA, TCF12, CLOCK | 765 | 513 | 16792 | 1.754314617 | 0.905071534 | 0.111065374 | 1.200677928 |
| GOTERM_BP_DIRECT | GO:0000122~negative regulation of transcription from RNA polymerase II promoter | 53 | 6.471 | 6.80E-04 | EFNA1, FST, PPARG, CTCF, ANKRD1, FOXO3, CBX8, HOXC8, SIN3A, CRY2, PCBP3, HAMP, POU4F2, RARB, NR2F2, NFIL3, MYC, STRN3, RCOR1, ARID1A, PROX1, UHRF1, NCOA2, KDM2B, FGFR2, SOX2, HCFC1, CIC, TCF7L2, MSX2, TSC22D3, MEIS2, EZR, NR1D1, SQSTM1, PER1, PER3, BCOR, TCF3, FOXD3, MAF, INSM1, HCLS1, ASXL1, SKI, DACH1, STAT3, ATF3, SAP130, SP3, ATF7, SMURF2, RERE | 765 | 720 | 16792 | 1.615787945 | 0.912326244 | 0.109445592 | 1.240964654 |
| GOTERM_BP_DIRECT | GO:0045787~positive regulation of cell cycle | 8 | 0.977 | 7.20E-04 | FGFR2, TRIM32, TGM1, HCFC1, PROX1, TCF3, MYC, C6ORF89 | 765 | 34 | 16792 | 5.164782776 | 0.923982336 | 0.110527597 | 1.313214109 |
| GOTERM_BP_DIRECT | GO:0030035~microspike assembly | 4 | 0.488 | 8.76E-04 | MTSS1, FSCN1, ACTN2, ABL1 | 765 | 5 | 16792 | 17.56026144 | 0.956516263 | 0.127436946 | 1.595601448 |
| GOTERM_BP_DIRECT | GO:0008652~cellular amino acid biosynthetic process | 7 | 0.855 | 9.16E-04 | GLS2, CBSL, NAALAD2, GLUL, GOT1, OAT, CBS | 765 | 26 | 16792 | 5.909703369 | 0.9623458 | 0.127714019 | 1.668239999 |
| GOTERM_BP_DIRECT | GO:0035987~endodermal cell differentiation | 7 | 0.855 | 0.00113271 | COL4A2, ITGAV, MMP9, COL12A1, COL6A1, MMP14, COL11A1 | 765 | 27 | 16792 | 5.690825466 | 0.982686708 | 0.149772424 | 2.059402047 |

| GOTERM_BP_DIRECT | GO:0007165~signal transduction | 76 | 9.28 | 0.00127908 | GNA13, NAMPT, MPZL1, GABRB3, PPP2R5D, HBS1L, PPARG, FST, RCVRN, CXCL12, CANT1, ACVR1B, ARHGAP5, PAK2, ANK2, ZYX, RARB, NR2F2, ITPK1, IFNGR1, AKT2, CASKIN1, IRS2, NRXN2, NECTIN1, PRKAB1, PDE4D, NRXN1, PI4KB, CDS1, PRKCD, PRKCB, GNAL, NPC1, HIF1A, MAPK6, GNB1, CD34, GRN, LRP12, GNB5, CLOCK, CHL1, SH3GL2, SH3GL1, CHKA, C3, SPOCK2, CREM, CXCL9, PTK7, CNTFR, CABP5, SOX9, FAM13A, TNFRSF1A, RGMB, CAMK2B, PRKAA1, SH2B1, FGFBP1, PIK3R1, GUCA1C, DPYSL5, NDFIP1, NR4A1, DPYSL2, STAT3, SH3BP5, GRIA2, RPS6KA1, ULK2, HGS, IGFBP1, GRK3, CHRNE | 765 | 1161 | 16792 | 1.436886164 | 0.989752871 | 0.16153546 | 2.322567562 |
| --- | --- | --- | --- | --- | --- | --- | --- | --- | --- | --- | --- | --- |
| GOTERM_BP_DIRECT | GO:0050821~protein stabilization | 16 | 1.954 | 0.00133587 | ATP1B1, FLOT2, NLK, STXBP1, CHP1, HCFC1, PINK1, PRKCD, PTEN, LAMP2, CDKN1A, A1CF, ANK2, BAG3, PER3, PIK3R1 | 765 | 136 | 16792 | 2.582391388 | 0.991639677 | 0.162383358 | 2.424487024 |
| GOTERM_BP_DIRECT | GO:0045892~negative regulation of transcription, DNA-templated | 39 | 4.762 | 0.00138102 | CDK5R1, TFAP4, TSG101, CCDC85B, CREM, CIART, PPARG, CTCF, SOX9, TCF7L2, MSX2, CIPC, CRY2, SIN3A, NR1D1, NR1D2, BRD7, POU3F3, PER1, NR2F2, LOXL2, BCOR, ZNF423, MYF6, SOX10, STRN3, RCOR1, DACH1, PROX1, ATF5, SFRP1, SFRP2, SP3, FOXG1, PSPC1, SMURF2, RBM15, CLOCK, KAT6A | 765 | 499 | 16792 | 1.715556603 | 0.992888719 | 0.161923138 | 2.505452976 |
| GOTERM_BP_DIRECT | GO:0007568~aging | 18 | 2.198 | 0.00141087 | ND4, PDE4D, FOXO3, TIMP2, GCLM, PTEN, STAT3, ALDH3A1, GSS, SLC32A1, FOS, CD86, SIN3A, HAMP, FOXG1, CTSC, IGFBP1, LOXL2 | 765 | 165 | 16792 | 2.394581105 | 0.993610269 | 0.159907736 | 2.558949907 |
| GOTERM_BP_DIRECT | GO:0010629~negative regulation of gene expression | 16 | 1.954 | 0.00143869 | LDLR, INPPL1, FLOT2, NDFIP1, PINK1, ACACB, NOCT, CDC42, ACVR1B, CDKN1A, SFRP1, CD34, SFRP2, POU3F2, PC, TP53INP1 | 765 | 137 | 16792 | 2.563541816 | 0.994216667 | 0.157818029 | 2.608781217 |
| GOTERM_BP_DIRECT | GO:0030307~positive regulation of cell growth | 12 | 1.465 | 0.00144376 | CDC42, EIF4G2, ADNP2, KDM2B, ADAM10, SFRP1, RPS6KA1, SFRP2, TRIM32, IGFBP1, ACSL4, MMP14 | 765 | 84 | 16792 | 3.135760971 | 0.994320805 | 0.153635114 | 2.617859335 |
| GOTERM_BP_DIRECT | GO:0031581~hemidesmosome assembly | 5 | 0.611 | 0.00156982 | ITGA6, ITGB4, LAMC1, CD151, PLEC | 765 | 12 | 16792 | 9.145969499 | 0.99638547 | 0.161140711 | 2.843331064 |
| GOTERM_BP_DIRECT | GO:0006468~protein phosphorylation | 36 | 4.396 | 0.00182875 | PRPF4B, STK10, STK35, PTK7, PINK1, ACVR1B, MAP3K5, STK40, SNRK, PAK2, SQSTM1, CAMK2D, PRKAA1, CAMK2B, DYRK2, SIK1, PIK3R1, PDK2, CAMK1G, ADAM10, NLK, MEX3B, TAOK3, MYLK4, PRKAB1, PRKCD, DAPK3, SRPK1, PRKCB, MAPK6, RPS6KA1, GSK3B, DYRK1A, MAPK3, GRK3, NRBP2 | 765 | 456 | 16792 | 1.732920537 | 0.998571437 | 0.180054789 | 3.304911775 |
| GOTERM_BP_DIRECT | GO:0006730~one-carbon metabolic process | 7 | 0.855 | 0.00202299 | SHMT2, MAT2A, MAT1A, CA5B, AHCYL1, CA2, AHCYL2 | 765 | 30 | 16792 | 5.121742919 | 0.999288141 | 0.191977685 | 3.649818373 |
| GOTERM_BP_DIRECT | GO:0009611~response to wounding | 10 | 1.221 | 0.00208137 | ZFP36L1, SLC1A2, ACHE, SLC1A3, SULF2, SOX2, RAC1, ITGB4, MYC, FABP5 | 765 | 63 | 16792 | 3.484178857 | 0.999422605 | 0.191889669 | 3.753242819 |
| GOTERM_BP_DIRECT | GO:0016477~cell migration | 18 | 2.198 | 0.0022083 | CTHRC1, PTPRF, EFNA1, FSCN1, NANOS1, PTK7, CD151, PTEN, ITGB1, SCRIB, RAB1A, COL5A1, ITGAV, GSK3B, LAMC1, THBS1, ABL1, APC | 765 | 172 | 16792 | 2.297127223 | 0.999633763 | 0.19730795 | 3.977759328 |
| GOTERM_BP_DIRECT | GO:0071333~cellular response to glucose stimulus | 9 | 1.099 | 0.00225597 | SLC29A1, IRS2, TJP1, SIN3A, CYP7A1, PPP3CA, FOXO3, GCLM, KCNJ11 | 765 | 52 | 16792 | 3.799095023 | 0.99969133 | 0.19624911 | 4.061961791 |
| GOTERM_BP_DIRECT | GO:0010976~positive regulation of neuron projection development | 12 | 1.465 | 0.0023129 | RGMA, SCN1B, NCK1, ENC1, PTK7, PTN, DPYSL3, CAMK2B, ANKRD1, RAPGEF2, GPRC5B, TMEM30A | 765 | 89 | 16792 | 2.959594624 | 0.999748346 | 0.195947646 | 4.162417973 |
| GOTERM_BP_DIRECT | GO:0045893~positive regulation of transcription, DNA-templated | 39 | 4.762 | 0.0024068 | ING5, ELF1, TFAP4, SOX2, PPARG, CTCF, FOXO3, SOX9, FOS, RGMB, FUBP3, NR1D1, NR1D2, CREB3L2, BRD7, POU3F3, POU3F1, NR2F2, MYC, TCF3, ZNF423, ESRRG, ARID1A, PROX1, ATXN7L3, STAT3, ATF5, CD86, HIF1A, SFRP1, NCOA3, SP3, MAPK3, USP21, PHF8, CLOCK, KAT6A, RNF111, TP53INP1 | 765 | 515 | 16792 | 1.662257757 | 0.999820315 | 0.198392393 | 4.327889818 |
| GOTERM_BP_DIRECT | GO:0016032~viral process | 26 | 3.175 | 0.00258997 | TLN1, AP1M1, NUP98, NUP153, ZC3H7B, RCOR1, HLA-A, TSPAN7, SYNCRIP, LMBRD1, STAT3, SCRIB, SRPK1, RAB11FIP4, TNFRSF1A, MAP3K5, ACOT8, KRT18, PAK2, KLC1, MAPK3, ATF7, ZYX, TNPO1, RBM15, PIK3R1 | 765 | 299 | 16792 | 1.908724069 | 0.999906871 | 0.207084001 | 4.649910481 |
| GOTERM_BP_DIRECT | GO:0006810~transport | 29 | 3.541 | 0.00259091 | NUP98, CLCN3, AP1M1, AQP9, SLC15A1, SLC6A1, GABRB3, GABRB2, PITPNA, ATP1B4, SEC14L6, AQP6, SEC14L4, ENSA, AQP3, ATP2B2, ATP2B3, SLC25A1, SLC4A4, ABCB8, GOLIM4, CACNG2, CACNG1, ABCB6, RAB11FIP4, CDH17, SLC7A2, SLC25A10, CHRNE | 765 | 348 | 16792 | 1.8291939 | 0.999907185 | 0.202649515 | 4.651563277 |
| GOTERM_BP_DIRECT | GO:0090002~establishment of protein localization to plasma membrane | 8 | 0.977 | 0.00263316 | TNFRSF1A, PACSIN1, EZR, TSPAN33, PKP3, FLOT2, TSPAN14, ACTN2 | 765 | 42 | 16792 | 4.181014628 | 0.999920243 | 0.20122746 | 4.725699414 |
| GOTERM_BP_DIRECT | GO:0051932~synaptic transmission, GABAergic | 4 | 0.488 | 0.00286114 | CLSTN3, GABRB2, RAC1, DNM1 | 765 | 7 | 16792 | 12.54304388 | 0.999964809 | 0.212176907 | 5.124765276 |
| GOTERM_BP_DIRECT | GO:2001259~positive regulation of cation channel activity | 4 | 0.488 | 0.00286114 | ANK2, ACTN2, CTSS, KCNJ11 | 765 | 7 | 16792 | 12.54304388 | 0.999964809 | 0.212176907 | 5.124765276 |
| GOTERM_BP_DIRECT | GO:0043101~purine-containing compound salvage | 5 | 0.611 | 0.0029517 | GMPR2, ADK, GMPR, HPRT1, PNP | 765 | 14 | 16792 | 7.839402428 | 0.999974574 | 0.213724943 | 5.282828642 |
| GOTERM_BP_DIRECT | GO:1902230~negative regulation of intrinsic apoptotic signaling pathway in response to DNA damage | 5 | 0.611 | 0.0029517 | SFRP2, TRIM32, TMEM161A, BCL2L1, CXCL12 | 765 | 14 | 16792 | 7.839402428 | 0.999974574 | 0.213724943 | 5.282828642 |
| GOTERM_BP_DIRECT | GO:0032469~endoplasmic reticulum calcium ion homeostasis | 5 | 0.611 | 0.00388156 | ATP2A2, GRINA, KCTD17, CAMK2D, TMCO1 | 765 | 15 | 16792 | 7.316775599 | 0.999999098 | 0.266049948 | 6.89164702 |
| GOTERM_BP_DIRECT | GO:0014898~cardiac muscle hypertrophy in response to stress | 5 | 0.611 | 0.00388156 | TCAP, ATP2A2, PPP3CA, KLF15, NPPA | 765 | 15 | 16792 | 7.316775599 | 0.999999098 | 0.266049948 | 6.89164702 |
| GOTERM_BP_DIRECT | GO:0043401~steroid hormone mediated signaling pathway | 9 | 1.099 | 0.00405992 | NR1D1, RXRB, NR1D2, PPARG, ESRRG, PAQR6, NR4A1, RARB, NR2F2 | 765 | 57 | 16792 | 3.465841073 | 0.999999525 | 0.271321371 | 7.197267687 |
| GOTERM_BP_DIRECT | GO:0071456~cellular response to hypoxia | 12 | 1.465 | 0.00419601 | SLC29A1, ZFP36L1, HIF1A, SFRP1, PTGS2, GNB1, PINK1, PTN, PRKAA1, ANKRD1, FOXO3, PTEN | 765 | 96 | 16792 | 2.74379085 | 0.999999709 | 0.273991718 | 7.429818191 |
| GOTERM_BP_DIRECT | GO:0001504~neurotransmitter uptake | 4 | 0.488 | 0.00442365 | GLUL, SLC1A3, SV2B, SV2A | 765 | 8 | 16792 | 10.9751634 | 0.999999872 | 0.281486245 | 7.817593483 |
| GOTERM_BP_DIRECT | GO:0071260~cellular response to mechanical stimulus | 10 | 1.221 | 0.00474788 | TNFRSF1A, PTGS2, BAG3, MAPK3, RAC1, CNN2, ANKRD1, SOX9, GADD45A, NPPA | 765 | 71 | 16792 | 3.091595324 | 0.99999996 | 0.293629101 | 8.367232901 |
| GOTERM_BP_DIRECT | GO:0015914~phospholipid transport | 6 | 0.733 | 0.00478513 | NPC2, STARD7, LDLR, PITPNA, SCP2, TMEM30A | 765 | 25 | 16792 | 5.268078431 | 0.999999965 | 0.290604411 | 8.430188895 |
| GOTERM_BP_DIRECT | GO:0007050~cell cycle arrest | 15 | 1.832 | 0.00500262 | TSG101, SOX2, PRKAB1, GAS1, TCF7L2, MFN2, EIF4G2, CDKN1A, PRKAA1, THBS1, ABL1, MYC, GADD45A, TP53INP1, APC | 765 | 141 | 16792 | 2.335141149 | 0.999999984 | 0.296682669 | 8.796900979 |
| GOTERM_BP_DIRECT | GO:0001701~in utero embryonic development | 18 | 2.198 | 0.00521985 | GNA13, SRSF1, FGFR2, SOX10, MAFF, ADAM10, ND4, KEAP1, BCL2L1, MYH9, ITGB1, SLC34A2, ACVR1B, SIN3A, MYC, EPN1, FOXD3, ADD1 | 765 | 187 | 16792 | 2.112865681 | 0.999999993 | 0.302468381 | 9.161809335 |

| GOTERM_BP_DIRECT | GO:0046777~protein autophosphorylation | 17 | 2.076 | 0.00531763 | FGFR2, MEX3B, CAMK2G, STK10, NLK, TAOK3, DAPK3, THY1, ACVR1B, PAK2, ULK2, GSK3B, DYRK1A, CAMK2D, CAMK2B, SIK1, ABL1 | 765 | 172 | 16792 | 2.169509044 | 0.999999995 | 0.302357709 | 9.325602244 |
| --- | --- | --- | --- | --- | --- | --- | --- | --- | --- | --- | --- | --- |
| GOTERM_BP_DIRECT | GO:0043065~positive regulation of apoptotic process | 25 | 3.053 | 0.00542302 | ING5, PTGS2, TFAP4, GRIN1, NR4A1, ANKRD1, BCL2L1, ARHGEF9, FOXO3, DAPK3, ITGB1, SCRIB, MAP3K5, ITGA6, SFRP1, SFRP2, SQSTM1, RAC1, DNAJA1, PTN, CLIP3, RARB, ABL1, GADD45A, APC | 765 | 300 | 16792 | 1.8291939 | 0.999999996 | 0.302605429 | 9.501834374 |
| GOTERM_BP_DIRECT | GO:0030534~adult behavior | 6 | 0.733 | 0.00570639 | SLC1A2, NRXN2, SPTBN2, NRXN1, PCDH17, PTEN | 765 | 26 | 16792 | 5.06546003 | 0.999999999 | 0.310917332 | 9.974080563 |
| GOTERM_BP_DIRECT | GO:0016310~phosphorylation | 12 | 1.465 | 0.00572513 | CHKA, PAK2, PANK1, MAPK3, NT5C2, AK3, PPIP5K1, PINK1, ITPKC, ITPK1, PAPSS2, STAT3 | 765 | 100 | 16792 | 2.634039216 | 0.999999999 | 0.307154501 | 10.00521714 |
| GOTERM_BP_DIRECT | GO:0034332~adherens junction organization | 7 | 0.855 | 0.00604846 | CDC42, RAB8B, CADM1, CDH17, NECTIN1, CTNND1, DSP | 765 | 37 | 16792 | 4.152764529 | 1 | 0.31677623 | 10.54104378 |
| GOTERM_BP_DIRECT | GO:0007420~brain development | 18 | 2.198 | 0.00610901 | CDK5R1, IRS2, COL4A1, SPHK2, CADM1, PFKFB3, SLC6A11, CTNND1, DPYSL2, ABCB6, PROX1, DDIT4, SLC23A1, BAG3, FOXG1, PPP3CC, PHF8, HAP1 | 765 | 190 | 16792 | 2.079504644 | 1 | 0.314854411 | 10.64104202 |
| GOTERM_BP_DIRECT | GO:0016337~single organismal cell-cell adhesion | 12 | 1.465 | 0.0061686 | ITGA6, CD34, PKP3, NECTIN1, CYFIP2, GPA33, CTNND1, DSP, KIRREL2, SOX9, SCRIB, THY1 | 765 | 101 | 16792 | 2.607959619 | 1 | 0.312952793 | 10.73936286 |
| GOTERM_BP_DIRECT | GO:0007584~response to nutrient | 10 | 1.221 | 0.00624799 | SFRP2, PPARG, LIPG, ENSA, ABCA1, ACSL4, SLC6A19, PTEN, GCLM, ALDH3A1 | 765 | 74 | 16792 | 2.966260378 | 1 | 0.31192805 | 10.87019728 |
| GOTERM_BP_DIRECT | GO:0007399~nervous system development | 24 | 2.93 | 0.0062681 | GDA, PCDHA8, ACHE, CAMK2G, PPP2R5D, ENC1, DPYSL5, DPYSL4, CNTFR, GPM6B, ELAVL3, DPYSL2, MYT1, STAT3, APLP1, GSS, PCSK2, FOS, SLC4A10, TRIM3, MTR, DYRK1A, PTN, ZNF423 | 765 | 287 | 16792 | 1.835567398 | 1 | 0.30851936 | 10.90330261 |
| GOTERM_BP_DIRECT | GO:0071380~cellular response to prostaglandin E stimulus | 5 | 0.611 | 0.00629513 | SFRP1, GNB1, PPARG, ACACA, PRKAA1 | 765 | 17 | 16792 | 6.45597847 | 1 | 0.305484016 | 10.94779154 |
| GOTERM_BP_DIRECT | GO:0014070~response to organic cyclic compound | 8 | 0.977 | 0.00637324 | NAMPT, G6PD, SFRP1, ABCC4, ACACB, MMP14, CPT1A, POLR2A | 765 | 49 | 16792 | 3.583726824 | 1 | 0.30456606 | 11.07622144 |
| GOTERM_BP_DIRECT | GO:0097186~amelogenesis | 4 | 0.488 | 0.00641254 | MMP20, ITGA6, ITGB4, PERP | 765 | 9 | 16792 | 9.755700799 | 1 | 0.3021534 | 11.14077984 |
| GOTERM_BP_DIRECT | GO:1900034~regulation of cellular response to heat | 10 | 1.221 | 0.00682143 | HSPA1L, NUP153, NUP98, CAMK2G, GSK3B, BAG3, MAPK3, BAG2, CAMK2D, CAMK2B | 765 | 75 | 16792 | 2.92671024 | 1 | 0.314005248 | 11.80979664 |
| GOTERM_BP_DIRECT | GO:0007268~chemical synaptic transmission | 21 | 2.564 | 0.00685841 | UNC119, SCN1B, SNAP91, NRXN2, PTPRF, SLC6A1, GABRB2, SLC6A11, GLRA3, FLOT2, GRIN1, NRXN1, AMPH, SLC1A2, SLC1A3, SYN1, GRIA2, RAPSN, HAP1, NPFF, NOVA1 | 765 | 240 | 16792 | 1.920653595 | 1 | 0.311468488 | 11.87007135 |
| GOTERM_BP_DIRECT | GO:0018107~peptidyl-threonine phosphorylation | 7 | 0.855 | 0.00691221 | ACVR1B, CDK5R1, GSK3B, NLK, DYRK1A, CAMK2D, PRKCD | 765 | 38 | 16792 | 4.043481252 | 1 | 0.309623447 | 11.95769077 |
| GOTERM_BP_DIRECT | GO:0030522~intracellular receptor signaling pathway | 7 | 0.855 | 0.00691221 | NCOA2, NR1D1, NCOA3, NR1D2, NR4A1, NR2F2, STAT3 | 765 | 38 | 16792 | 4.043481252 | 1 | 0.309623447 | 11.95769077 |
| GOTERM_BP_DIRECT | GO:0010811~positive regulation of cell-substrate adhesion | 7 | 0.855 | 0.00691221 | ITGA6, SPOCK2, NPNT, RAC1, PTN, NID1, VIT | 765 | 38 | 16792 | 4.043481252 | 1 | 0.309623447 | 11.95769077 |
| GOTERM_BP_DIRECT | GO:0031668~cellular response to extracellular stimulus | 5 | 0.611 | 0.00780669 | FOS, SLC1A2, CDKN1A, ITGA6, SFRP2 | 765 | 18 | 16792 | 6.097312999 | 1 | 0.338004632 | 13.40235193 |
| GOTERM_BP_DIRECT | GO:0007223~Wnt signaling pathway, calcium modulating pathway | 7 | 0.855 | 0.00786184 | GNB1, NLK, PPP3R1, AGO4, PPP3CA, PDE6G, TCF7L2 | 765 | 39 | 16792 | 3.939802246 | 1 | 0.335952586 | 13.4906874 |
| GOTERM_BP_DIRECT | GO:0045668~negative regulation of osteoblast differentiation | 7 | 0.855 | 0.00786184 | TRPM4, SFRP1, GDF10, SKI, CHRD, NOCT, TOB1 | 765 | 39 | 16792 | 3.939802246 | 1 | 0.335952586 | 13.4906874 |
| GOTERM_BP_DIRECT | GO:0055088~lipid homeostasis | 7 | 0.855 | 0.00786184 | APOA4, IRS2, NR1D2, PPARG, ACACA, ACOX3, ABCA12 | 765 | 39 | 16792 | 3.939802246 | 1 | 0.335952586 | 13.4906874 |
| GOTERM_BP_DIRECT | GO:0000165~MAPK cascade | 22 | 2.686 | 0.00874251 | FGFR2, IRS2, EFNA1, NLK, CAMK2G, GRIN1, TAOK3, ACTN2, MARK3, NCAM1, ZFP36L1, NRAS, MAP3K5, MAPK6, MAPK3, SPTBN2, CAMK2D, POU4F2, SPTBN1, CAMK2B, RAPGEF2, MYC | 765 | 262 | 16792 | 1.843157212 | 1 | 0.361706606 | 14.88977278 |
| GOTERM_BP_DIRECT | GO:0008286~insulin receptor signaling pathway | 10 | 1.221 | 0.00878538 | PDK2, NAMPT, IRS2, BAIAP2, ATP6V0A1, IGFBP1, PIK3R3, PIK3R1, ATP6V0B, AKT2 | 765 | 78 | 16792 | 2.814144461 | 1 | 0.359056692 | 14.9573379 |
| GOTERM_BP_DIRECT | GO:0060674~placenta blood vessel development | 4 | 0.488 | 0.00885373 | SOCS3, ARID1A, NR2F2, RBM15 | 765 | 10 | 16792 | 8.780130719 | 1 | 0.357291482 | 15.06493951 |
| GOTERM_BP_DIRECT | GO:0046627~negative regulation of insulin receptor signaling pathway | 6 | 0.733 | 0.00920851 | SOCS3, SOCS1, LMBRD1, PRKCD, KANK1, PRKCB | 765 | 29 | 16792 | 4.541446924 | 1 | 0.364638177 | 15.62141009 |
| GOTERM_BP_DIRECT | GO:0001894~tissue homeostasis | 5 | 0.611 | 0.00953838 | CD34, TRIM32, NANOS1, ACACA, SOX9 | 765 | 19 | 16792 | 5.776401789 | 1 | 0.370945053 | 16.13572339 |
| GOTERM_BP_DIRECT | GO:0046718~viral entry into host cell | 10 | 1.221 | 0.0103147 | NCAM1, NPC1, NUP153, CD86, LDLR, XPR1, ITGAV, NECTIN1, SCARB2, ITGB1 | 765 | 80 | 16792 | 2.74379085 | 1 | 0.390291211 | 17.33443926 |
| GOTERM_BP_DIRECT | GO:0030182~neuron differentiation | 11 | 1.343 | 0.01109075 | ATP2B2, CDK5R1, HOXC8, ADNP2, POU4F2, POU3F2, RTN1, PROX1, DAPK3, NRBP2, DDIT4 | 765 | 95 | 16792 | 2.541616787 | 1 | 0.408565678 | 18.51651932 |
| GOTERM_BP_DIRECT | GO:0006357~regulation of transcription from RNA polymerase II promoter | 32 | 3.907 | 0.01128441 | ZNF76, FOXO3, TCF7L2, TAL2, FOS, RAD21, HSF2, CAMK2D, BRD7, TEF, POU4F2, NR2F2, MYF6, MAF, SOX10, MAFF, KLF9, BARHL1, ARID1A, TAF6L, LPIN1, STAT3, PRKCB, ATF5, ZNF865, ATF3, DBP, SP4, FOXG1, MAFA, TCF12, CLOCK | 765 | 441 | 16792 | 1.592767477 | 1 | 0.409914141 | 18.80900775 |
| GOTERM_BP_DIRECT | GO:0046545~development of primary female sexual characteristics | 3 | 0.366 | 0.01166693 | ACVR1B, DACH2, DACH1 | 765 | 4 | 16792 | 16.4627451 | 1 | 0.416365207 | 19.38380133 |
| GOTERM_BP_DIRECT | GO:0015837~amine transport | 3 | 0.366 | 0.01166693 | SLC32A1, RHCG, AQP9 | 765 | 4 | 16792 | 16.4627451 | 1 | 0.416365207 | 19.38380133 |
| GOTERM_BP_DIRECT | GO:0010748~negative regulation of plasma membrane long-chain fatty acid transport | 3 | 0.366 | 0.01166693 | IRS2, THBS1, AKT2 | 765 | 4 | 16792 | 16.4627451 | 1 | 0.416365207 | 19.38380133 |
| GOTERM_BP_DIRECT | GO:0070837~dehydroascorbic acid transport | 3 | 0.366 | 0.01166693 | SLC23A1, SLC2A2, SLC2A1 | 765 | 4 | 16792 | 16.4627451 | 1 | 0.416365207 | 19.38380133 |
| GOTERM_BP_DIRECT | GO:0048333~mesodermal cell differentiation | 4 | 0.488 | 0.01176684 | FGFR2, ITGB4, ITGB1, KDM6B | 765 | 11 | 16792 | 7.981937017 | 1 | 0.415058714 | 19.5333046 |
| GOTERM_BP_DIRECT | GO:0051764~actin crosslink formation | 4 | 0.488 | 0.01176684 | TNNT2, BAIAP2, DPYSL3, PLS3 | 765 | 11 | 16792 | 7.981937017 | 1 | 0.415058714 | 19.5333046 |
| GOTERM_BP_DIRECT | GO:0046676~negative regulation of insulin secretion | 6 | 0.733 | 0.01222872 | MIDN, SFRP1, VSNL1, PPP3CA, KCNJ11, NPFF | 765 | 31 | 16792 | 4.248450348 | 1 | 0.423312596 | 20.22101897 |
| GOTERM_BP_DIRECT | GO:0048147~negative regulation of fibroblast proliferation | 6 | 0.733 | 0.01222872 | SFRP1, TRIM32, SKI, DACH1, MYC, TP53INP1 | 765 | 31 | 16792 | 4.248450348 | 1 | 0.423312596 | 20.22101897 |
| GOTERM_BP_DIRECT | GO:0006629~lipid metabolic process | 15 | 1.832 | 0.01248114 | CYP3A4, CHKA, LDLR, PITPNA, PTPRN2, PPARG, GPCPD1, DAGLB, G6PD, LIPG, PLA2G6, ACSL4, NR2F2, FABP5, PC | 765 | 157 | 16792 | 2.097164981 | 1 | 0.425900133 | 20.59450803 |
| GOTERM_BP_DIRECT | GO:0050680~negative regulation of epithelial cell proliferation | 8 | 0.977 | 0.01310143 | FGFR2, MTSS1, SFRP1, SFRP2, SOX2, PTN, SOX9, PTEN | 765 | 56 | 16792 | 3.135760971 | 1 | 0.437637466 | 21.50530588 |
| GOTERM_BP_DIRECT | GO:0008284~positive regulation of cell proliferation | 33 | 4.029 | 0.01395864 | FGFR2, NAMPT, CNTFR, BCL2L1, CBX8, SOX9, CNOT6, ITGB1, PTEN, ALDH3A1, ITGAV, POU3F3, PTN, POU3F2, PRKAA1, YAP1, RARB, THBS1, MYC, FGFBP1, COL18A1, TRPM4, SHMT2, IRS2, ADAM10, SPHK2, HCLS1, PROX1, STAT3, CD86, ATF3, SFRP1, SFRP2 | 765 | 466 | 16792 | 1.554422284 | 1 | 0.454549823 | 22.74772897 |
| GOTERM_BP_DIRECT | GO:0030514~negative regulation of BMP signaling pathway | 7 | 0.855 | 0.01560873 | RBPMS2, SFRP1, SFRP2, SKI, ABL1, CHRD, TOB1 | 765 | 45 | 16792 | 3.41449528 | 1 | 0.488438878 | 25.08716563 |
| GOTERM_BP_DIRECT | GO:0051592~response to calcium ion | 8 | 0.977 | 0.01571798 | TNNT2, SEC31A, SLC6A1, ANXA11, AHCYL1, PPP3CA, THBS1, AQP3 | 765 | 58 | 16792 | 3.027631282 | 1 | 0.486792912 | 25.23967168 |
| GOTERM_BP_DIRECT | GO:0060999~positive regulation of dendritic spine development | 5 | 0.611 | 0.01616249 | EIF4G2, PALM, MAPK6, ARF1, RAC1 | 765 | 22 | 16792 | 4.988710636 | 1 | 0.492428533 | 25.85714724 |

| GOTERM_BP_DIRECT | GO:0043524~negative regulation of neuron apoptotic process | 13 | 1.587 | 0.01726473 | GABRB3, GABRB2, BARHL1, GRIN1, STXBP1, PINK1, CNTFR, BCL2L1, GCLM, KDM2B, CHL1, NRBP2, OXR1 | 765 | 132 | 16792 | 2.161774609 | 1 | 0.511510674 | 27.3675302 |
| --- | --- | --- | --- | --- | --- | --- | --- | --- | --- | --- | --- | --- |
| GOTERM_BP_DIRECT | GO:0048661~positive regulation of smooth muscle cell proliferation | 8 | 0.977 | 0.01868525 | FGFR2, RBPMS2, NAMPT, PTGS2, CAMK2D, ABCC4, THBS1, MYC | 765 | 60 | 16792 | 2.92671024 | 1 | 0.535654902 | 29.27118719 |
| GOTERM_BP_DIRECT | GO:0015812~gamma-aminobutyric acid transport | 3 | 0.366 | 0.01886111 | SLC32A1, SLC6A1, SLC6A11 | 765 | 5 | 16792 | 13.17019608 | 1 | 0.534998551 | 29.50353843 |
| GOTERM_BP_DIRECT | GO:0042634~regulation of hair cycle | 3 | 0.366 | 0.01886111 | FA2H, PER1, CLOCK | 765 | 5 | 16792 | 13.17019608 | 1 | 0.534998551 | 29.50353843 |
| GOTERM_BP_DIRECT | GO:0071447~cellular response to hydroperoxide | 3 | 0.366 | 0.01886111 | PRKCD, TP53INP1, OXR1 | 765 | 5 | 16792 | 13.17019608 | 1 | 0.534998551 | 29.50353843 |
| GOTERM_BP_DIRECT | GO:0070296~sarcoplasmic reticulum calcium ion transport | 3 | 0.366 | 0.01886111 | ANK2, ATP2A2, CACNG1 | 765 | 5 | 16792 | 13.17019608 | 1 | 0.534998551 | 29.50353843 |
| GOTERM_BP_DIRECT | GO:0015793~glycerol transport | 4 | 0.488 | 0.01905968 | AQP9, AQP11, AQP6, AQP3 | 765 | 13 | 16792 | 6.753946707 | 1 | 0.534785208 | 29.76504478 |
| GOTERM_BP_DIRECT | GO:0048546~digestive tract morphogenesis | 4 | 0.488 | 0.01905968 | SOX10, HIF1A, SFRP1, SFRP2 | 765 | 13 | 16792 | 6.753946707 | 1 | 0.534785208 | 29.76504478 |
| GOTERM_BP_DIRECT | GO:0006195~purine nucleotide catabolic process | 4 | 0.488 | 0.01905968 | GDA, NT5C1A, NT5C2, PNP | 765 | 13 | 16792 | 6.753946707 | 1 | 0.534785208 | 29.76504478 |
| GOTERM_BP_DIRECT | GO:0043647~inositol phosphate metabolic process | 7 | 0.855 | 0.01907064 | NUDT4, INPPL1, PPIP5K1, PLCD1, ITPKC, ITPK1, PTEN | 765 | 47 | 16792 | 3.269197608 | 1 | 0.531062602 | 29.77944859 |
| GOTERM_BP_DIRECT | GO:0032956~regulation of actin cytoskeleton organization | 7 | 0.855 | 0.01907064 | CDK5R1, EZR, BAIAP2, FSCN1, GPM6B, ABL1, PRKCD | 765 | 47 | 16792 | 3.269197608 | 1 | 0.531062602 | 29.77944859 |
| GOTERM_BP_DIRECT | GO:0086091~regulation of heart rate by cardiac conduction | 6 | 0.733 | 0.02014018 | TRPM4, SCN1B, ANK2, DSG2, CAMK2D, DSP | 765 | 35 | 16792 | 3.762913165 | 1 | 0.546832878 | 31.17198242 |
| GOTERM_BP_DIRECT | GO:0007416~synapse assembly | 8 | 0.977 | 0.02030711 | ACHE, NRXN2, GPM6A, CLSTN3, SPOCK2, SPTBN2, NRXN1, PTEN | 765 | 61 | 16792 | 2.878731383 | 1 | 0.545946435 | 31.38694603 |
| GOTERM_BP_DIRECT | GO:0030335~positive regulation of cell migration | 16 | 1.954 | 0.02103583 | INSM1, COL18A1, IRS2, ADAM10, MYO1C, MCAM, MMP14, DAPK3, ITGA6, ITGAV, SEMA3F, TRIM32, THBS1, PIK3R1, AKT2, APC | 765 | 184 | 16792 | 1.908724069 | 1 | 0.554907101 | 32.31797885 |
| GOTERM_BP_DIRECT | GO:0045471~response to ethanol | 11 | 1.343 | 0.02120314 | TJP1, G6PD, ND4, HAMP, GRIN1, MAOB, GGH, PTEN, MYC, SDF4, STAT3 | 765 | 105 | 16792 | 2.299558045 | 1 | 0.553979611 | 32.53004238 |
| GOTERM_BP_DIRECT | GO:0022617~extracellular matrix disassembly | 9 | 1.099 | 0.02189688 | MMP20, ADAM10, MMP9, NID1, CTSS, LAMC1, MMP14, CAPN2, TIMP2 | 765 | 76 | 16792 | 2.599380805 | 1 | 0.561946196 | 33.4026766 |
| GOTERM_BP_DIRECT | GO:0051453~regulation of intracellular pH | 6 | 0.733 | 0.02253924 | SLC4A10, SLC26A5, CHP1, CA2, TM9SF4, SLC4A4 | 765 | 36 | 16792 | 3.6583878 | 1 | 0.568782881 | 34.2011463 |
| GOTERM_BP_DIRECT | GO:0035914~skeletal muscle cell differentiation | 7 | 0.855 | 0.02302216 | MYF6, MAFF, FOS, HLF, ATF3, NR4A1, ANKRD1 | 765 | 49 | 16792 | 3.135760971 | 1 | 0.572844932 | 34.7954682 |
| GOTERM_BP_DIRECT | GO:0048172~regulation of short-term neuronal synaptic plasticity | 4 | 0.488 | 0.02345277 | SYP, SHISA7, PPFIA3, SYNGR1 | 765 | 14 | 16792 | 6.271521942 | 1 | 0.575971028 | 35.32111722 |
| GOTERM_BP_DIRECT | GO:2000147~positive regulation of cell motility | 4 | 0.488 | 0.02345277 | SLC26A5, SPOCK2, TRIM32, AKT2 | 765 | 14 | 16792 | 6.271521942 | 1 | 0.575971028 | 35.32111722 |
| GOTERM_BP_DIRECT | GO:0072659~protein localization to plasma membrane | 8 | 0.977 | 0.02383987 | ATP1B1, ANK2, RAC1, STXBP1, RAPGEF2, SCP2, AKT2, ABCA12 | 765 | 63 | 16792 | 2.787343085 | 1 | 0.578343367 | 35.79024272 |
| GOTERM_BP_DIRECT | GO:0034612~response to tumor necrosis factor | 5 | 0.611 | 0.02510981 | GSS, ADAM10, PTGS2, TRIM32, GCH1 | 765 | 25 | 16792 | 4.390065359 | 1 | 0.593895134 | 37.30677021 |
| GOTERM_BP_DIRECT | GO:0006656~phosphatidylcholine biosynthetic process | 5 | 0.611 | 0.02510981 | CHKA, ACHE, GPAT4, LPIN1, FABP5 | 765 | 25 | 16792 | 4.390065359 | 1 | 0.593895134 | 37.30677021 |
| GOTERM_BP_DIRECT | GO:2001235~positive regulation of apoptotic signaling pathway | 5 | 0.611 | 0.02510981 | ING5, CTSC, PTEN, PRKCD, TP53INP1 | 765 | 25 | 16792 | 4.390065359 | 1 | 0.593895134 | 37.30677021 |
| GOTERM_BP_DIRECT | GO:0001666~response to hypoxia | 15 | 1.832 | 0.0255226 | ATP1B1, ND4, MMP14, CAPN2, CXCL12, PRKCB, ALDH3A1, DDIT4, HIF1A, CAMK2D, PRKAA1, THBS1, TM9SF4, LOXL2, NPPA | 765 | 172 | 16792 | 1.914272686 | 1 | 0.596334848 | 37.79238231 |
| GOTERM_BP_DIRECT | GO:0006874~cellular calcium ion homeostasis | 10 | 1.221 | 0.02553164 | ATP1B1, ATP2B3, ANK2, ATP2A2, GRIN1, SV2A, CXCL12, ATP13A5, PRKCB, ATP13A4 | 765 | 93 | 16792 | 2.360250193 | 1 | 0.592895041 | 37.80297017 |
| GOTERM_BP_DIRECT | GO:0042632~cholesterol homeostasis | 8 | 0.977 | 0.02575554 | APOA4, NPC1, NPC2, LDLR, DGAT2, CYP7A1, LIPG, ABCA1 | 765 | 64 | 16792 | 2.74379085 | 1 | 0.592596429 | 38.0648341 |
| GOTERM_BP_DIRECT | GO:0006563~L-serine metabolic process | 3 | 0.366 | 0.02744563 | CBSL, SHMT2, CBS | 765 | 6 | 16792 | 10.9751634 | 1 | 0.61270994 | 40.00812605 |
| GOTERM_BP_DIRECT | GO:0031113~regulation of microtubule polymerization | 3 | 0.366 | 0.02744563 | MAPT, SLAIN2, ABL1 | 765 | 6 | 16792 | 10.9751634 | 1 | 0.61270994 | 40.00812605 |
| GOTERM_BP_DIRECT | GO:2001171~positive regulation of ATP biosynthetic process | 3 | 0.366 | 0.02744563 | PINK1, MYC, STAT3 | 765 | 6 | 16792 | 10.9751634 | 1 | 0.61270994 | 40.00812605 |
| GOTERM_BP_DIRECT | GO:2000323~negative regulation of glucocorticoid receptor signaling pathway | 3 | 0.366 | 0.02744563 | CRY2, PER1, CLOCK | 765 | 6 | 16792 | 10.9751634 | 1 | 0.61270994 | 40.00812605 |
| GOTERM_BP_DIRECT | GO:1901018~positive regulation of potassium ion transmembrane transporter activity | 3 | 0.366 | 0.02744563 | ATP1B1, ANK2, ACTN2 | 765 | 6 | 16792 | 10.9751634 | 1 | 0.61270994 | 40.00812605 |
| GOTERM_BP_DIRECT | GO:0006537~glutamate biosynthetic process | 3 | 0.366 | 0.02744563 | GLS2, SLC1A3, ALDH4A1 | 765 | 6 | 16792 | 10.9751634 | 1 | 0.61270994 | 40.00812605 |
| GOTERM_BP_DIRECT | GO:0007269~neurotransmitter secretion | 7 | 0.855 | 0.02748901 | SLC32A1, PPFIA3, SYN1, NRXN2, PTPRN2, STXBP1, NRXN1 | 765 | 51 | 16792 | 3.012789953 | 1 | 0.609816685 | 40.05724033 |
| GOTERM_BP_DIRECT | GO:0035019~somatic stem cell population maintenance | 8 | 0.977 | 0.02777371 | SFRP1, SOX2, SKI, YAP1, SOX9, STAT3, POLR2A, FOXD3 | 765 | 65 | 16792 | 2.701578683 | 1 | 0.610205866 | 40.37862169 |
| GOTERM_BP_DIRECT | GO:0034446~substrate adhesion-dependent cell spreading | 6 | 0.733 | 0.02787406 | EFNA1, ITGAV, RAC1, LAMC1, ABL1, RAB1A | 765 | 38 | 16792 | 3.465841073 | 1 | 0.608133303 | 40.49150456 |
| GOTERM_BP_DIRECT | GO:2001237~negative regulation of extrinsic apoptotic signaling pathway | 6 | 0.733 | 0.02787406 | ITGA6, ITGAV, YAP1, THBS1, GCLM, TCF7L2 | 765 | 38 | 16792 | 3.465841073 | 1 | 0.608133303 | 40.49150456 |
| GOTERM_BP_DIRECT | GO:0002063~chondrocyte development | 4 | 0.488 | 0.02834557 | MSX2, SULF2, SFRP2, COL11A1 | 765 | 15 | 16792 | 5.853420479 | 1 | 0.610997068 | 41.01922354 |
| GOTERM_BP_DIRECT | GO:0030502~negative regulation of bone mineralization | 4 | 0.488 | 0.02834557 | TRPM4, HIF1A, BCOR, SOX9 | 765 | 15 | 16792 | 5.853420479 | 1 | 0.610997068 | 41.01922354 |
| GOTERM_BP_DIRECT | GO:0002053~positive regulation of mesenchymal cell proliferation | 5 | 0.611 | 0.0286377 | FGFR2, IRS2, SOX9, CHRD, MYC | 765 | 26 | 16792 | 4.221216692 | 1 | 0.611463727 | 41.34396059 |
| GOTERM_BP_DIRECT | GO:0008283~cell proliferation | 26 | 3.175 | 0.02921572 | CDK5R1, ACHE, BCL2L1, TCF7L2, PTEN, SCRIB, ZFP36L1, TYR, RAC1, YAP1, MYC, IRS2, ARHGEF1, SPHK2, FSCN1, SKI, DACH1, STAT3, TACC1, DDIT4, UHRF1, GLUL, CD34, GNB1, NAA60, LIPG | 765 | 366 | 16792 | 1.559312833 | 1 | 0.615589484 | 41.9815138 |
| GOTERM_BP_DIRECT | GO:0034220~ion transmembrane transport | 17 | 2.076 | 0.03066421 | PLP2, ATP1B1, GABRB3, NCALD, GABRB2, GLRA3, ARHGEF9, AQP3, ATP13A5, ATP6V0B, ATP13A4, ATP2B2, TSC22D3, ATP2B3, GRIA2, ATP2A2, ATP6V0A1 | 765 | 210 | 16792 | 1.776931217 | 1 | 0.630359353 | 43.55054336 |
| GOTERM_BP_DIRECT | GO:0010628~positive regulation of gene expression | 20 | 2.442 | 0.03110725 | LDLR, PRKAB1, HCFC1, CTCF, STAT3, HNRNPU, CDC42, FUBP3, EZR, HIF1A, ANK2, NCOA3, CD34, POU3F3, HGS, CNN2, PRKAA1, POU3F1, NFIL3, MYC | 765 | 262 | 16792 | 1.675597465 | 1 | 0.632450051 | 44.02237885 |
| GOTERM_BP_DIRECT | GO:0060349~bone morphogenesis | 5 | 0.611 | 0.03244498 | FGFR2, DHRS3, SFRP2, SKI, ACTN3 | 765 | 27 | 16792 | 4.064875333 | 1 | 0.644948366 | 45.4245266 |
| GOTERM_BP_DIRECT | GO:0007602~phototransduction | 5 | 0.611 | 0.03244498 | OPN5, UNC119, PDC, RCVRN, CDS1 | 765 | 27 | 16792 | 4.064875333 | 1 | 0.644948366 | 45.4245266 |
| GOTERM_BP_DIRECT | GO:0055007~cardiac muscle cell differentiation | 5 | 0.611 | 0.03244498 | ARID1A, SIK1, PROX1, ITGB1, KDM6B | 765 | 27 | 16792 | 4.064875333 | 1 | 0.644948366 | 45.4245266 |
| GOTERM_BP_DIRECT | GO:0035994~response to muscle stretch | 4 | 0.488 | 0.03373503 | FOS, TCAP, ANKRD1, NPPA | 765 | 16 | 16792 | 5.487581699 | 1 | 0.656308348 | 46.74519992 |
| GOTERM_BP_DIRECT | GO:0009749~response to glucose | 8 | 0.977 | 0.03446374 | GLUL, IRS2, COL6A2, ENSA, MAFA, THBS1, PTEN, TCF7L2 | 765 | 68 | 16792 | 2.582391388 | 1 | 0.661110302 | 47.47780006 |
| GOTERM_BP_DIRECT | GO:0016050~vesicle organization | 5 | 0.611 | 0.03653399 | SNX18, SNX5, SNX8, SNX1, PINK1 | 765 | 28 | 16792 | 3.919701214 | 1 | 0.679698096 | 49.50743925 |
| GOTERM_BP_DIRECT | GO:0050690~regulation of defense response to virus by virus | 5 | 0.611 | 0.03653399 | AP1M1, PAK2, ARF1, RAC1, HLA-A | 765 | 28 | 16792 | 3.919701214 | 1 | 0.679698096 | 49.50743925 |
| GOTERM_BP_DIRECT | GO:0014047~glutamate secretion | 5 | 0.611 | 0.03653399 | GLS2, PPFIA3, SLC1A2, SLC1A3, STXBP1 | 765 | 28 | 16792 | 3.919701214 | 1 | 0.679698096 | 49.50743925 |

| GOTERM_BP_DIRECT | GO:0030916~otic vesicle formation | 3 | 0.366 | 0.03727942 | FGFR2, TCAP, SOX9 | 765 | 7 | 16792 | 9.407282913 | 1 | 0.684096541 | 50.21991047 |
| --- | --- | --- | --- | --- | --- | --- | --- | --- | --- | --- | --- | --- |
| GOTERM_BP_DIRECT | GO:0060074~synapse maturation | 3 | 0.366 | 0.03727942 | SEZ6L2, PALM, PTEN | 765 | 7 | 16792 | 9.407282913 | 1 | 0.684096541 | 50.21991047 |
| GOTERM_BP_DIRECT | GO:0050667~homocysteine metabolic process | 3 | 0.366 | 0.03727942 | CBSL, DPEP1, CBS | 765 | 7 | 16792 | 9.407282913 | 1 | 0.684096541 | 50.21991047 |
| GOTERM_BP_DIRECT | GO:0010882~regulation of cardiac muscle contraction by calcium ion signaling | 3 | 0.366 | 0.03727942 | ATP1B1, ANK2, ATP2A2 | 765 | 7 | 16792 | 9.407282913 | 1 | 0.684096541 | 50.21991047 |
| GOTERM_BP_DIRECT | GO:0014883~transition between fast and slow fiber | 3 | 0.366 | 0.03727942 | ATP2A2, ACTN3, PPP3CA | 765 | 7 | 16792 | 9.407282913 | 1 | 0.684096541 | 50.21991047 |
| GOTERM_BP_DIRECT | GO:0055013~cardiac muscle cell development | 3 | 0.366 | 0.03727942 | ACTN2, HNRNPU, SGCB | 765 | 7 | 16792 | 9.407282913 | 1 | 0.684096541 | 50.21991047 |
| GOTERM_BP_DIRECT | GO:0032808~lacrimal gland development | 3 | 0.366 | 0.03727942 | FGFR2, SOX10, SOX9 | 765 | 7 | 16792 | 9.407282913 | 1 | 0.684096541 | 50.21991047 |
| GOTERM_BP_DIRECT | GO:0008285~negative regulation of cell proliferation | 27 | 3.297 | 0.03957069 | INSM1, B4GALT1, COL18A1, ING5, TFAP4, PTGS2, TSG101, INPPL1, SKI, TIMP2, PTEN, ARID2, PROX1, STAT3, MSX2, ATF5, CDKN1A, SFRP1, SFRP2, BRD7, HGS, RARB, RAPGEF2, FGFBP1, TOB1, TP53INP1, APC | 765 | 396 | 16792 | 1.496613191 | 1 | 0.703082363 | 52.3508417 |
| GOTERM_BP_DIRECT | GO:1901216~positive regulation of neuron death | 4 | 0.488 | 0.039615 | MAP3K5, GSK3B, ABL1, DDIT4 | 765 | 17 | 16792 | 5.164782776 | 1 | 0.700475008 | 52.39119001 |
| GOTERM_BP_DIRECT | GO:1903779~regulation of cardiac conduction | 7 | 0.855 | 0.04104949 | ATP2B2, ATP1B1, ATP2B3, ATP2A2, AHCYL1, KCNJ11, NPPA | 765 | 56 | 16792 | 2.74379085 | 1 | 0.71055999 | 53.6800375 |
| GOTERM_BP_DIRECT | GO:0016311~dephosphorylation | 9 | 1.099 | 0.04172507 | DUSP4, PFKFB4, PFKFB3, NT5C1A, NT5C2, PPIP5K1, PPP3CA, ITPK1, LPIN1 | 765 | 86 | 16792 | 2.297127223 | 1 | 0.713586761 | 54.27554253 |
| GOTERM_BP_DIRECT | GO:0048013~ephrin receptor signaling pathway | 9 | 1.099 | 0.04172507 | CDC42, CDK5R1, ADAM10, EFNA1, MMP9, NCK1, GRIN1, RAC1, DNM1 | 765 | 86 | 16792 | 2.297127223 | 1 | 0.713586761 | 54.27554253 |
| GOTERM_BP_DIRECT | GO:0043066~negative regulation of apoptotic process | 30 | 3.663 | 0.04356642 | XIAP, MMP9, BCL2L1, SOX9, PTEN, MSX2, SIN3A, STK40, PAK2, SQSTM1, BAG3, DNAJA1, POU3F3, PRKAA1, RARB, AGO4, THBS1, MYC, DPEP1, PIK3R1, SOX10, SPHK2, SOCS3, STAT3, ATF5, CDKN1A, KRT18, SFRP1, RPS6KA1, GSK3B | 765 | 455 | 16792 | 1.447274294 | 1 | 0.726408708 | 55.86208679 |
| GOTERM_BP_DIRECT | GO:0031623~receptor internalization | 6 | 0.733 | 0.04448919 | ACHE, EZR, SNX1, GRK3, ITGB1, DNM1 | 765 | 43 | 16792 | 3.062836297 | 1 | 0.731130068 | 56.63743876 |
| GOTERM_BP_DIRECT | GO:0070588~calcium ion transmembrane transport | 11 | 1.343 | 0.04479594 | TRPM4, ATP2B2, ATP2B3, SLC25A25, ATP2A2, GPM6A, ITGAV, GRIN1, CACNG2, CACNG1, TMCO1 | 765 | 119 | 16792 | 2.029021805 | 1 | 0.730776357 | 56.89232823 |
| GOTERM_BP_DIRECT | GO:0048010~vascular endothelial growth factor receptor signaling pathway | 8 | 0.977 | 0.04493221 | CDC42, PAK2, ITGAV, BAIAP2, NCK1, RAC1, CYFIP2, PIK3R1 | 765 | 72 | 16792 | 2.4389252 | 1 | 0.729058069 | 57.00509488 |
| GOTERM_BP_DIRECT | GO:0046326~positive regulation of glucose import | 5 | 0.611 | 0.04556153 | SLC1A2, IRS2, ARPP19, PIK3R1, AKT2 | 765 | 30 | 16792 | 3.6583878 | 1 | 0.731295734 | 57.52229416 |
| GOTERM_BP_DIRECT | GO:0098656~anion transmembrane transport | 5 | 0.611 | 0.04556153 | SLC4A10, ABCC4, AQP6, ABCA1, SLC4A4 | 765 | 30 | 16792 | 3.6583878 | 1 | 0.731295734 | 57.52229416 |
| GOTERM_BP_DIRECT | GO:0090004~positive regulation of establishment of protein localization to plasma membrane | 5 | 0.611 | 0.04556153 | EZR, CLIP3, NRXN1, ITGB1, PIK3R1 | 765 | 30 | 16792 | 3.6583878 | 1 | 0.731295734 | 57.52229416 |
| GOTERM_BP_DIRECT | GO:0044344~cellular response to fibroblast growth factor stimulus | 5 | 0.611 | 0.04556153 | ZFP36L1, SFRP1, NR4A1, GCLM, MYC | 765 | 30 | 16792 | 3.6583878 | 1 | 0.731295734 | 57.52229416 |
| GOTERM_BP_DIRECT | GO:0048384~retinoic acid receptor signaling pathway | 4 | 0.488 | 0.04597667 | RXRB, CREM, ESRRG, RARB | 765 | 18 | 16792 | 4.877850399 | 1 | 0.731805174 | 57.86024574 |
| GOTERM_BP_DIRECT | GO:0048169~regulation of long-term neuronal synaptic plasticity | 4 | 0.488 | 0.04597667 | SYP, GRIN1, CAMK2B, SYNGR1 | 765 | 18 | 16792 | 4.877850399 | 1 | 0.731805174 | 57.86024574 |
| GOTERM_BP_DIRECT | GO:0001649~osteoblast differentiation | 10 | 1.221 | 0.04730055 | MSX2, BMP3, SFRP1, SOX2, GDF10, COL6A1, SYNCRIP, VCAN, CHRD, HNRNPU | 765 | 104 | 16792 | 2.110608346 | 1 | 0.739295236 | 58.92107543 |
| GOTERM_BP_DIRECT | GO:0060041~retina development in camera-type eye | 7 | 0.855 | 0.04747364 | ACHE, GNB1, NECTIN1, POU4F2, SKI, SOX9, PRPH2 | 765 | 58 | 16792 | 2.649177372 | 1 | 0.737899635 | 59.05789185 |
| GOTERM_BP_DIRECT | GO:0006661~phosphatidylinositol biosynthetic process | 7 | 0.855 | 0.04747364 | ARF1, INPPL1, PI4KB, CDS1, PIK3R3, PTEN, PIK3R1 | 765 | 58 | 16792 | 2.649177372 | 1 | 0.737899635 | 59.05789185 |
| GOTERM_BP_DIRECT | GO:0055114~oxidation-reduction process | 37 | 4.518 | 0.04793001 | CYP3A4, ME2, CYP2J2, GMPR2, PTGS2, IL4I1, KMO, BBOX1, ALDH3A1, CYP3A43, GPD1L, TYR, CYP7A1, ALDH4A1, HHIP, LOXL2, LBR, OXR1, MSMO1, CYP2C8, SCD, FA2H, MAOB, IDO2, GMPR, VAT1, DHRS3, KDM2B, G6PD, PHF2, KDM2A, CYP27A1, ABCC4, PHF8, KDM6B, PRODH, CBS | 765 | 592 | 16792 | 1.371895425 | 1 | 0.738651092 | 59.41655558 |
| GOTERM_BP_DIRECT | GO:0021707~cerebellar granule cell differentiation | 3 | 0.366 | 0.04823134 | ATP2B2, NRXN1, PROX1 | 765 | 8 | 16792 | 8.231372549 | 1 | 0.738237046 | 59.6517455 |
| GOTERM_BP_DIRECT | GO:0000059~protein import into nucleus, docking | 3 | 0.366 | 0.04823134 | NUP98, TNPO2, TNPO1 | 765 | 8 | 16792 | 8.231372549 | 1 | 0.738237046 | 59.6517455 |
| GOTERM_BP_DIRECT | GO:0021860~pyramidal neuron development | 3 | 0.366 | 0.04823134 | FGFR2, SLC4A10, OGDH | 765 | 8 | 16792 | 8.231372549 | 1 | 0.738237046 | 59.6517455 |
| GOTERM_BP_DIRECT | GO:0070544~histone H3-K36 demethylation | 3 | 0.366 | 0.04823134 | KDM2B, KDM2A, PHF8 | 765 | 8 | 16792 | 8.231372549 | 1 | 0.738237046 | 59.6517455 |
| GOTERM_BP_DIRECT | GO:0034644~cellular response to UV | 6 | 0.733 | 0.04839132 | PTGS2, TMEM161A, PTN, MYC, PIK3R1, TP53INP1 | 765 | 44 | 16792 | 2.993226381 | 1 | 0.736779164 | 59.77608494 |
| GOTERM_BP_DIRECT | GO:0001764~neuron migration | 10 | 1.221 | 0.04974718 | CDK5R1, GPM6A, MAPT, BARHL1, PEX5, RAPGEF2, NR2F2, CXCL12, CHL1, DDIT4 | 765 | 105 | 16792 | 2.090507314 | 1 | 0.744078207 | 60.81544188 |
| GOTERM_BP_DIRECT | GO:0007160~cell-matrix adhesion | 9 | 1.099 | 0.05220446 | ITGA6, CD34, ITGAV, NPNT, RAC1, ITGB4, NID1, ZYX, ITGB1 | 765 | 90 | 16792 | 2.19503268 | 1 | 0.758631945 | 62.63470366 |
| GOTERM_BP_DIRECT | GO:0010975~regulation of neuron projection development | 4 | 0.488 | 0.05280884 | SFRP1, SFRP2, DPYSL3, PTEN | 765 | 19 | 16792 | 4.621121431 | 1 | 0.760156131 | 63.06976545 |
| GOTERM_BP_DIRECT | GO:0034976~response to endoplasmic reticulum stress | 8 | 0.977 | 0.0539934 | MAP3K5, ATP2A2, PPP2CB, CREB3L2, PLA2G6, THBS1, ABL1, PIK3R1 | 765 | 75 | 16792 | 2.341368192 | 1 | 0.76543831 | 63.90860293 |
| GOTERM_BP_DIRECT | GO:0045732~positive regulation of protein catabolic process | 7 | 0.855 | 0.05448584 | RAB7A, GSK3B, TRIM32, NDFIP1, SNX1, SOX9, APC | 765 | 60 | 16792 | 2.56087146 | 1 | 0.766140052 | 64.25199131 |
| GOTERM_BP_DIRECT | GO:0051289~protein homotetramerization | 7 | 0.855 | 0.05448584 | SHMT2, MAT1A, SYT11, ACACA, ACTN2, ACACB, HPRT1 | 765 | 60 | 16792 | 2.56087146 | 1 | 0.766140052 | 64.25199131 |
| GOTERM_BP_DIRECT | GO:0032355~response to estradiol | 9 | 1.099 | 0.05506585 | SLC6A1, PTGS2, STRN3, PTN, NR2F2, PTEN, MYC, KCNJ11, STAT3 | 765 | 91 | 16792 | 2.170911441 | 1 | 0.767386965 | 64.65248245 |
| GOTERM_BP_DIRECT | GO:0030900~forebrain development | 6 | 0.733 | 0.05678292 | KDM2B, SOX2, ARID1A, POU3F1, NR2F2, CHRD | 765 | 46 | 16792 | 2.863086104 | 1 | 0.775629802 | 65.81338153 |
| GOTERM_BP_DIRECT | GO:0030509~BMP signaling pathway | 8 | 0.977 | 0.05724957 | RGMA, BMP3, RGMB, MAPK3, GDF10, SMURF2, SKI, TOB1 | 765 | 76 | 16792 | 2.310560716 | 1 | 0.776069637 | 66.12259591 |
| GOTERM_BP_DIRECT | GO:0019886~antigen processing and presentation of exogenous peptide antigen via MHC class II | 9 | 1.099 | 0.05802536 | RAB7A, AP1M1, SEC31A, ARF1, KLC1, SPTBN2, CTSS, SH3GL2, CTSF | 765 | 92 | 16792 | 2.147314578 | 1 | 0.778344577 | 66.63081042 |
| GOTERM_BP_DIRECT | GO:0032092~positive regulation of protein binding | 7 | 0.855 | 0.05821443 | SPPL3, CTHRC1, GSK3B, RAPGEF2, TCF7L2, ADD2, ADD1 | 765 | 61 | 16792 | 2.51888996 | 1 | 0.777119431 | 66.75356625 |
| GOTERM_BP_DIRECT | GO:0006833~water transport | 4 | 0.488 | 0.06009836 | AQP9, AQP11, AQP6, AQP3 | 765 | 20 | 16792 | 4.390065359 | 1 | 0.785718018 | 67.95367319 |
| GOTERM_BP_DIRECT | GO:0006853~carnitine shuttle | 3 | 0.366 | 0.06017957 | ACACA, ACACB, CPT1A | 765 | 9 | 16792 | 7.316775599 | 1 | 0.783890765 | 68.0044723 |
| GOTERM_BP_DIRECT | GO:0010469~regulation of receptor activity | 3 | 0.366 | 0.06017957 | SERPINE1, PDE4D, PRKCD | 765 | 9 | 16792 | 7.316775599 | 1 | 0.783890765 | 68.0044723 |
| GOTERM_BP_DIRECT | GO:0032000~positive regulation of fatty acid beta-oxidation | 3 | 0.366 | 0.06017957 | IRS2, CPT1A, AKT2 | 765 | 9 | 16792 | 7.316775599 | 1 | 0.783890765 | 68.0044723 |
| GOTERM_BP_DIRECT | GO:0030643~cellular phosphate ion homeostasis | 3 | 0.366 | 0.06017957 | XPR1, GPCPD1, SLC34A2 | 765 | 9 | 16792 | 7.316775599 | 1 | 0.783890765 | 68.0044723 |
| GOTERM_BP_DIRECT | GO:0021799~cerebral cortex radially oriented cell migration | 3 | 0.366 | 0.06017957 | RAC1, POU3F3, POU3F2 | 765 | 9 | 16792 | 7.316775599 | 1 | 0.783890765 | 68.0044723 |

| GOTERM_BP_DIRECT | GO:0042754~negative regulation of circadian rhythm | 3 | 0.366 | 0.06017957 | CIPC, SIN3A, CRY2 | 765 | 9 | 16792 | 7.316775599 | 1 | 0.783890765 | 68.0044723 |
| --- | --- | --- | --- | --- | --- | --- | --- | --- | --- | --- | --- | --- |
| GOTERM_BP_DIRECT | GO:0032836~glomerular basement membrane development | 3 | 0.366 | 0.06017957 | COL4A4, SULF2, NID1 | 765 | 9 | 16792 | 7.316775599 | 1 | 0.783890765 | 68.0044723 |
| GOTERM_BP_DIRECT | GO:0003094~glomerular filtration | 3 | 0.366 | 0.06017957 | SULF2, CD34, MCAM | 765 | 9 | 16792 | 7.316775599 | 1 | 0.783890765 | 68.0044723 |
| GOTERM_BP_DIRECT | GO:0032886~regulation of microtubule-based process | 3 | 0.366 | 0.06017957 | GSK3B, KLHL42, APC | 765 | 9 | 16792 | 7.316775599 | 1 | 0.783890765 | 68.0044723 |
| GOTERM_BP_DIRECT | GO:2000304~positive regulation of ceramide biosynthetic process | 3 | 0.366 | 0.06017957 | TNFRSF1A, PLA2G6, PRKCD | 765 | 9 | 16792 | 7.316775599 | 1 | 0.783890765 | 68.0044723 |
| GOTERM_BP_DIRECT | GO:0032869~cellular response to insulin stimulus | 8 | 0.977 | 0.06062474 | ZFP36L1, IRS2, GOT1, PPARG, LPIN1, MYC, PIK3R1, AKT2 | 765 | 77 | 16792 | 2.280553433 | 1 | 0.784132963 | 68.28159463 |
| GOTERM_BP_DIRECT | GO:0015758~glucose transport | 5 | 0.611 | 0.06121725 | PPBP, SLC2A2, SLC2A1, KLF15, FABP5 | 765 | 33 | 16792 | 3.325807091 | 1 | 0.785195119 | 68.64692066 |
| GOTERM_BP_DIRECT | GO:0045600~positive regulation of fat cell differentiation | 6 | 0.733 | 0.06127247 | ZFP36L1, TRPM4, SFRP1, SFRP2, PPARG, NOCT | 765 | 47 | 16792 | 2.802169378 | 1 | 0.783259739 | 68.68076395 |
| GOTERM_BP_DIRECT | GO:0006470~protein dephosphorylation | 11 | 1.343 | 0.06151558 | PPM1K, PPP2R5D, CAMK2G, PTPRN2, PPP2CB, PPP3R1, PPM1N, PPP3CC, PTPN13, PPP3CA, PTEN | 765 | 126 | 16792 | 1.916298371 | 1 | 0.782382207 | 68.82935256 |
| GOTERM_BP_DIRECT | GO:0006811~ion transport | 11 | 1.343 | 0.06419385 | SLC32A1, PLP2, SLC1A2, SLC1A3, SLC15A1, SLCO4A1, GABRB2, AQP11, SLC25A10, CHRNE, CLDN15 | 765 | 127 | 16792 | 1.901209408 | 1 | 0.794649622 | 70.42278346 |
| GOTERM_BP_DIRECT | GO:0038096~Fc-gamma receptor signaling pathway involved in phagocytosis | 11 | 1.343 | 0.06419385 | CDC42, MYO1C, BAIAP2, NCK1, MAPK3, RAC1, CYFIP2, PLA2G6, ABL1, PRKCD, PIK3R1 | 765 | 127 | 16792 | 1.901209408 | 1 | 0.794649622 | 70.42278346 |
| GOTERM_BP_DIRECT | GO:0001934~positive regulation of protein phosphorylation | 11 | 1.343 | 0.06419385 | C3, SQSTM1, MMP9, MAPK3, RAC1, PLA2G6, PINK1, CLIP3, ABL1, SOX9, AKT2 | 765 | 127 | 16792 | 1.901209408 | 1 | 0.794649622 | 70.42278346 |
| GOTERM_BP_DIRECT | GO:0001837~epithelial to mesenchymal transition | 5 | 0.611 | 0.06699096 | FGFR2, HIF1A, GSK3B, LOXL2, SOX9 | 765 | 34 | 16792 | 3.227989235 | 1 | 0.806698643 | 72.00452823 |
| GOTERM_BP_DIRECT | GO:0016573~histone acetylation | 5 | 0.611 | 0.06699096 | ING5, NCOA2, NCOA3, CLOCK, KAT6A | 765 | 34 | 16792 | 3.227989235 | 1 | 0.806698643 | 72.00452823 |
| GOTERM_BP_DIRECT | GO:0097192~extrinsic apoptotic signaling pathway in absence of ligand | 5 | 0.611 | 0.06699096 | ITGAV, GSK3B, BAG3, FOXO3, BCL2L1 | 765 | 34 | 16792 | 3.227989235 | 1 | 0.806698643 | 72.00452823 |
| GOTERM_BP_DIRECT | GO:0030879~mammary gland development | 4 | 0.488 | 0.06783033 | B4GALT1, IRS2, ARHGAP5, SOX9 | 765 | 21 | 16792 | 4.181014628 | 1 | 0.808694714 | 72.46335908 |
| GOTERM_BP_DIRECT | GO:0006699~bile acid biosynthetic process | 4 | 0.488 | 0.06783033 | ACOT8, CYP27A1, CYP7A1, SCP2 | 765 | 21 | 16792 | 4.181014628 | 1 | 0.808694714 | 72.46335908 |
| GOTERM_BP_DIRECT | GO:0034599~cellular response to oxidative stress | 7 | 0.855 | 0.07029393 | ADNP2, G6PD, ATP2A2, TMEM161A, PINK1, FOXO3, ABL1 | 765 | 64 | 16792 | 2.400816993 | 1 | 0.818223845 | 73.76934426 |
| GOTERM_BP_DIRECT | GO:0071560~cellular response to transforming growth factor beta stimulus | 6 | 0.733 | 0.07083622 | ZFP36L1, COL4A2, SFRP1, ANKRD1, ABL1, SOX9 | 765 | 49 | 16792 | 2.687795118 | 1 | 0.818675663 | 74.0488483 |
| GOTERM_BP_DIRECT | GO:0006139~nucleobase-containing compound metabolic process | 6 | 0.733 | 0.07083622 | SLC29A1, GDA, SLC23A1, DPYSL2, SLC28A1, PNP | 765 | 49 | 16792 | 2.687795118 | 1 | 0.818675663 | 74.0488483 |
| GOTERM_BP_DIRECT | GO:0071481~cellular response to X-ray | 3 | 0.366 | 0.07301103 | SFRP1, HAMP, SFRP2 | 765 | 10 | 16792 | 6.585098039 | 1 | 0.826323472 | 75.1417228 |
| GOTERM_BP_DIRECT | GO:0045616~regulation of keratinocyte differentiation | 3 | 0.366 | 0.07301103 | ZFP36L1, GRHL1, AQP3 | 765 | 10 | 16792 | 6.585098039 | 1 | 0.826323472 | 75.1417228 |
| GOTERM_BP_DIRECT | GO:0040011~locomotion | 3 | 0.366 | 0.07301103 | ATP2B2, SCN1B, SPNS1 | 765 | 10 | 16792 | 6.585098039 | 1 | 0.826323472 | 75.1417228 |
| GOTERM_BP_DIRECT | GO:0051533~positive regulation of NFAT protein import into nucleus | 3 | 0.366 | 0.07301103 | SPPL3, PPP3R1, PPP3CA | 765 | 10 | 16792 | 6.585098039 | 1 | 0.826323472 | 75.1417228 |
| GOTERM_BP_DIRECT | GO:0045475~locomotor rhythm | 3 | 0.366 | 0.07301103 | NCOA2, CIART, PTEN | 765 | 10 | 16792 | 6.585098039 | 1 | 0.826323472 | 75.1417228 |
| GOTERM_BP_DIRECT | GO:0050765~negative regulation of phagocytosis | 3 | 0.366 | 0.07301103 | SYT11, CNN2, PTEN | 765 | 10 | 16792 | 6.585098039 | 1 | 0.826323472 | 75.1417228 |
| GOTERM_BP_DIRECT | GO:0006163~purine nucleotide metabolic process | 3 | 0.366 | 0.07301103 | GMPR2, GMPR, GUK1 | 765 | 10 | 16792 | 6.585098039 | 1 | 0.826323472 | 75.1417228 |
| GOTERM_BP_DIRECT | GO:0016242~negative regulation of macroautophagy | 3 | 0.366 | 0.07301103 | NPC1, PINK1, NRBP2 | 765 | 10 | 16792 | 6.585098039 | 1 | 0.826323472 | 75.1417228 |
| GOTERM_BP_DIRECT | GO:0006865~amino acid transport | 5 | 0.611 | 0.07303622 | SLC38A5, SLC7A3, SLC7A2, SLC6A19, MYC | 765 | 35 | 16792 | 3.135760971 | 1 | 0.824473032 | 75.15412117 |
| GOTERM_BP_DIRECT | GO:0010719~negative regulation of epithelial to mesenchymal transition | 4 | 0.488 | 0.0759884 | SFRP1, SFRP2, EFNA1, ADIPOR1 | 765 | 22 | 16792 | 3.990968509 | 1 | 0.834964877 | 76.56749737 |
| GOTERM_BP_DIRECT | GO:0090103~cochlea morphogenesis | 4 | 0.488 | 0.0759884 | CTHRC1, RAC1, PTK7, SOX9 | 765 | 22 | 16792 | 3.990968509 | 1 | 0.834964877 | 76.56749737 |
| GOTERM_BP_DIRECT | GO:0010906~regulation of glucose metabolic process | 4 | 0.488 | 0.0759884 | PDK2, NCOA2, ADIPOR1, ACACB | 765 | 22 | 16792 | 3.990968509 | 1 | 0.834964877 | 76.56749737 |
| GOTERM_BP_DIRECT | GO:0045736~negative regulation of cyclin-dependent protein serine/threonine kinase activity | 4 | 0.488 | 0.0759884 | CDKN1A, TFAP4, NR2F2, APC | 765 | 22 | 16792 | 3.990968509 | 1 | 0.834964877 | 76.56749737 |
| GOTERM_BP_DIRECT | GO:0050790~regulation of catalytic activity | 7 | 0.855 | 0.07909017 | BRCC3, PPP2R5D, BAG3, BAG2, ARHGEF15, CBX8, PPP2R2D | 765 | 66 | 16792 | 2.328064963 | 1 | 0.845314495 | 77.97040214 |
| GOTERM_BP_DIRECT | GO:0030177~positive regulation of Wnt signaling pathway | 5 | 0.611 | 0.07934834 | FGFR2, SULF2, SFRP1, SKI, KANK1 | 765 | 36 | 16792 | 3.0486565 | 1 | 0.844472766 | 78.08351983 |
| GOTERM_BP_DIRECT | GO:0042059~negative regulation of epidermal growth factor receptor signaling pathway | 5 | 0.611 | 0.07934834 | CDC42, TSG101, HGS, SH3GL2, EPN1 | 765 | 36 | 16792 | 3.0486565 | 1 | 0.844472766 | 78.08351983 |
| GOTERM_BP_DIRECT | GO:0006469~negative regulation of protein kinase activity | 9 | 1.099 | 0.08152638 | SH3BP5, PAK2, SOCS3, NCK1, TAOK3, SOCS1, CHP1, GADD45A, THY1 | 765 | 99 | 16792 | 1.995484254 | 1 | 0.850772798 | 79.01619746 |
| GOTERM_BP_DIRECT | GO:0006914~autophagy | 11 | 1.343 | 0.08182456 | NPC1, TSG101, SQSTM1, ULK2, ITGB4, HGS, VPS37B, ABL1, HAP1, VTI1A, RAB1A | 765 | 133 | 16792 | 1.815440562 | 1 | 0.850085188 | 79.14092703 |
| GOTERM_BP_DIRECT | GO:0007605~sensory perception of sound | 11 | 1.343 | 0.08182456 | ATP2B2, TJP1, SLC1A3, SLC26A5, GABRB3, GABRB2, BARHL1, POU4F2, COL11A1, DNM1, FBXO11 | 765 | 133 | 16792 | 1.815440562 | 1 | 0.850085188 | 79.14092703 |
| GOTERM_BP_DIRECT | GO:0006461~protein complex assembly | 10 | 1.221 | 0.08219067 | TFAP4, MAGI1, TCAP, MAPK3, SLC2A1, MPP6, LAMC1, SOX9, ADD2, APC | 765 | 116 | 16792 | 1.892269551 | 1 | 0.849649283 | 79.29310555 |
| GOTERM_BP_DIRECT | GO:0060070~canonical Wnt signaling pathway | 8 | 0.977 | 0.08338964 | CDC42, SFRP1, GSK3B, PTK7, PTEN, MYC, TCF7L2, APC | 765 | 83 | 16792 | 2.115694149 | 1 | 0.852195145 | 79.78417329 |
| GOTERM_BP_DIRECT | GO:0050796~regulation of insulin secretion | 7 | 0.855 | 0.0837096 | SLC2A2, SLC2A1, MARCKS, ENSA, KCNJ11, CLOCK, CPT1A | 765 | 67 | 16792 | 2.293317725 | 1 | 0.851597014 | 79.91334637 |
| GOTERM_BP_DIRECT | GO:0051291~protein heterooligomerization | 7 | 0.855 | 0.0837096 | TNNT2, MAT2A, SQSTM1, TMEM120B, PRKAB1, PRKAA1, GCH1 | 765 | 67 | 16792 | 2.293317725 | 1 | 0.851597014 | 79.91334637 |
| GOTERM_BP_DIRECT | GO:0033137~negative regulation of peptidyl-serine phosphorylation | 4 | 0.488 | 0.08455507 | GPD1L, NCK1, PDE4D, DDIT4 | 765 | 23 | 16792 | 3.817448139 | 1 | 0.852847352 | 80.25092994 |
| GOTERM_BP_DIRECT | GO:0046835~carbohydrate phosphorylation | 4 | 0.488 | 0.08455507 | PFKFB4, PFKFB3, ADK, NAGK | 765 | 23 | 16792 | 3.817448139 | 1 | 0.852847352 | 80.25092994 |
| GOTERM_BP_DIRECT | GO:0001525~angiogenesis | 16 | 1.954 | 0.08494012 | FGFR2, COL18A1, COL4A2, PTGS2, EFNA1, NRXN1, MYH9, MCAM, MMP14, PTEN, PNPLA6, THY1, HIF1A, ITGAV, SERPINE1, PLCD1 | 765 | 223 | 16792 | 1.57491134 | 1 | 0.852482946 | 80.40289245 |
| GOTERM_BP_DIRECT | GO:0010468~regulation of gene expression | 9 | 1.099 | 0.0852837 | ZFP36L1, INSM1, ATP1B1, HIF1A, STK40, PPP2CB, SOX2, PROX1, MYC | 765 | 100 | 16792 | 1.975529412 | 1 | 0.851978801 | 80.53755194 |
| GOTERM_BP_DIRECT | GO:0045597~positive regulation of cell differentiation | 5 | 0.611 | 0.08592197 | INSM1, RPS6KA1, SOCS3, SOX2, POU4F2 | 765 | 37 | 16792 | 2.966260378 | 1 | 0.852495807 | 80.78539179 |
| GOTERM_BP_DIRECT | GO:0021549~cerebellum development | 5 | 0.611 | 0.08592197 | CDK5R1, ND4, PTN, SDF4, HAP1 | 765 | 37 | 16792 | 2.966260378 | 1 | 0.852495807 | 80.78539179 |
| GOTERM_BP_DIRECT | GO:0050732~negative regulation of peptidyl-tyrosine phosphorylation | 3 | 0.366 | 0.08662085 | SFRP1, SFRP2, PRKCD | 765 | 11 | 16792 | 5.986452763 | 1 | 0.853212875 | 81.05334481 |
| GOTERM_BP_DIRECT | GO:0006098~pentose-phosphate shunt | 3 | 0.366 | 0.08662085 | PGM2, G6PD, DERA | 765 | 11 | 16792 | 5.986452763 | 1 | 0.853212875 | 81.05334481 |
| GOTERM_BP_DIRECT | GO:0033120~positive regulation of RNA splicing | 3 | 0.366 | 0.08662085 | SRSF1, PIK3R1, POLR2A | 765 | 11 | 16792 | 5.986452763 | 1 | 0.853212875 | 81.05334481 |

| GOTERM_BP_DIRECT | GO:0033690~positive regulation of osteoblast proliferation | 3 | 0.366 | 0.08662085 | CTHRC1, ITGAV, ABL1 | 765 | 11 | 16792 | 5.986452763 | 1 | 0.853212875 | 81.05334481 |
| --- | --- | --- | --- | --- | --- | --- | --- | --- | --- | --- | --- | --- |
| GOTERM_BP_DIRECT | GO:0098911~regulation of ventricular cardiac muscle cell action potential | 3 | 0.366 | 0.08662085 | TRPM4, DSG2, DSP | 765 | 11 | 16792 | 5.986452763 | 1 | 0.853212875 | 81.05334481 |
| GOTERM_BP_DIRECT | GO:0090557~establishment of endothelial intestinal barrier | 3 | 0.366 | 0.08662085 | TJP1, RAPGEF2, RAB1A | 765 | 11 | 16792 | 5.986452763 | 1 | 0.853212875 | 81.05334481 |
| GOTERM_BP_DIRECT | GO:0000096~sulfur amino acid metabolic process | 3 | 0.366 | 0.08662085 | MAT1A, MTR, GCLM | 765 | 11 | 16792 | 5.986452763 | 1 | 0.853212875 | 81.05334481 |
| GOTERM_BP_DIRECT | GO:0021756~striatum development | 3 | 0.366 | 0.08662085 | RARB, OGDH, HPRT1 | 765 | 11 | 16792 | 5.986452763 | 1 | 0.853212875 | 81.05334481 |
| GOTERM_BP_DIRECT | GO:0045820~negative regulation of glycolytic process | 3 | 0.366 | 0.08662085 | ACTN3, STAT3, DDIT4 | 765 | 11 | 16792 | 5.986452763 | 1 | 0.853212875 | 81.05334481 |
| GOTERM_BP_DIRECT | GO:0008344~adult locomotory behavior | 6 | 0.733 | 0.08662293 | SEZ6L2, GRIN1, PUM1, CXCL12, DNM1, CHL1 | 765 | 52 | 16792 | 2.532730015 | 1 | 0.851553847 | 81.05413817 |
| GOTERM_BP_DIRECT | GO:0006633~fatty acid biosynthetic process | 6 | 0.733 | 0.08662293 | MSMO1, FA2H, ACACA, PRKAB1, PRKAA1, ACACB | 765 | 52 | 16792 | 2.532730015 | 1 | 0.851553847 | 81.05413817 |
| GOTERM_BP_DIRECT | GO:0001507~acetylcholine catabolic process in synaptic cleft | 2 | 0.244 | 0.08892824 | ACHE, COLQ | 765 | 2 | 16792 | 21.9503268 | 1 | 0.857622072 | 81.91313512 |
| GOTERM_BP_DIRECT | GO:0014733~regulation of skeletal muscle adaptation | 2 | 0.244 | 0.08892824 | CAMK2G, CAMK2B | 765 | 2 | 16792 | 21.9503268 | 1 | 0.857622072 | 81.91313512 |
| GOTERM_BP_DIRECT | GO:2000041~negative regulation of planar cell polarity pathway involved in axis elongation | 2 | 0.244 | 0.08892824 | SFRP1, SFRP2 | 765 | 2 | 16792 | 21.9503268 | 1 | 0.857622072 | 81.91313512 |
| GOTERM_BP_DIRECT | GO:0060849~regulation of transcription involved in lymphatic endothelial cell fate commitment | 2 | 0.244 | 0.08892824 | NR2F2, PROX1 | 765 | 2 | 16792 | 21.9503268 | 1 | 0.857622072 | 81.91313512 |
| GOTERM_BP_DIRECT | GO:0006560~proline metabolic process | 2 | 0.244 | 0.08892824 | ALDH4A1, PRODH | 765 | 2 | 16792 | 21.9503268 | 1 | 0.857622072 | 81.91313512 |
| GOTERM_BP_DIRECT | GO:0015951~purine ribonucleotide interconversion | 2 | 0.244 | 0.08892824 | GMPR2, GMPR | 765 | 2 | 16792 | 21.9503268 | 1 | 0.857622072 | 81.91313512 |
| GOTERM_BP_DIRECT | GO:0046386~deoxyribose phosphate catabolic process | 2 | 0.244 | 0.08892824 | PGM2, DERA | 765 | 2 | 16792 | 21.9503268 | 1 | 0.857622072 | 81.91313512 |
| GOTERM_BP_DIRECT | GO:1904956~regulation of midbrain dopaminergic neuron differentiation | 2 | 0.244 | 0.08892824 | SFRP1, SFRP2 | 765 | 2 | 16792 | 21.9503268 | 1 | 0.857622072 | 81.91313512 |
| GOTERM_BP_DIRECT | GO:0006535~cysteine biosynthetic process from serine | 2 | 0.244 | 0.08892824 | CBSL, CBS | 765 | 2 | 16792 | 21.9503268 | 1 | 0.857622072 | 81.91313512 |
| GOTERM_BP_DIRECT | GO:0061188~negative regulation of chromatin silencing at rDNA | 2 | 0.244 | 0.08892824 | PHF2, PHF8 | 765 | 2 | 16792 | 21.9503268 | 1 | 0.857622072 | 81.91313512 |
| GOTERM_BP_DIRECT | GO:2001295~malonyl-CoA biosynthetic process | 2 | 0.244 | 0.08892824 | ACACA, ACACB | 765 | 2 | 16792 | 21.9503268 | 1 | 0.857622072 | 81.91313512 |
| GOTERM_BP_DIRECT | GO:0042593~glucose homeostasis | 9 | 1.099 | 0.08914085 | PDK2, HIF1A, CRY2, PPARG, ADIPOR1, PRKAA1, GPRC5B, TCF7L2, STAT3 | 765 | 101 | 16792 | 1.955969715 | 1 | 0.856696935 | 81.99047168 |
| GOTERM_BP_DIRECT | GO:0001568~blood vessel development | 5 | 0.611 | 0.09275113 | SPHK2, ITGAV, RAPGEF2, TCF7L2, COL5A1 | 765 | 38 | 16792 | 2.888200894 | 1 | 0.8665095 | 83.25696378 |
| GOTERM_BP_DIRECT | GO:0006695~cholesterol biosynthetic process | 5 | 0.611 | 0.09275113 | APOA4, MSMO1, G6PD, PRKAA1, LBR | 765 | 38 | 16792 | 2.888200894 | 1 | 0.8665095 | 83.25696378 |
| GOTERM_BP_DIRECT | GO:0090314~positive regulation of protein targeting to membrane | 4 | 0.488 | 0.09351181 | CDK5R1, MYO1C, CHP1, AKT2 | 765 | 24 | 16792 | 3.6583878 | 1 | 0.867265589 | 83.51284469 |
| GOTERM_BP_DIRECT | GO:0048675~axon extension | 4 | 0.488 | 0.09351181 | ULK2, MAPT, POU4F2, ITGB1 | 765 | 24 | 16792 | 3.6583878 | 1 | 0.867265589 | 83.51284469 |
| GOTERM_BP_DIRECT | GO:0051149~positive regulation of muscle cell differentiation | 4 | 0.488 | 0.09351181 | MYF6, CDC42, ABL1, TCF3 | 765 | 24 | 16792 | 3.6583878 | 1 | 0.867265589 | 83.51284469 |
| GOTERM_BP_DIRECT | GO:0070371~ERK1 and ERK2 cascade | 4 | 0.488 | 0.09351181 | ZFP36L1, ITGAV, MAPK3, SOX9 | 765 | 24 | 16792 | 3.6583878 | 1 | 0.867265589 | 83.51284469 |
| GOTERM_BP_DIRECT | GO:0045648~positive regulation of erythrocyte differentiation | 4 | 0.488 | 0.09351181 | ACVR1B, HIF1A, ISG15, FOXO3 | 765 | 24 | 16792 | 3.6583878 | 1 | 0.867265589 | 83.51284469 |
| GOTERM_BP_DIRECT | GO:0051924~regulation of calcium ion transport | 4 | 0.488 | 0.09351181 | ANK2, CAMK2G, RCVRN, CAMK2B | 765 | 24 | 16792 | 3.6583878 | 1 | 0.867265589 | 83.51284469 |
| GOTERM_BP_DIRECT | GO:0019915~lipid storage | 4 | 0.488 | 0.09351181 | CRY2, DGAT2, FITM2, BSCL2 | 765 | 24 | 16792 | 3.6583878 | 1 | 0.867265589 | 83.51284469 |
| GOTERM_BP_DIRECT | GO:0090102~cochlea development | 4 | 0.488 | 0.09351181 | ATP2B2, SLC26A5, GABRB3, GABRB2 | 765 | 24 | 16792 | 3.6583878 | 1 | 0.867265589 | 83.51284469 |
| GOTERM_BP_DIRECT | GO:0046907~intracellular transport | 4 | 0.488 | 0.09351181 | AFTPH, NUDT4, RAB14, APPBP2 | 765 | 24 | 16792 | 3.6583878 | 1 | 0.867265589 | 83.51284469 |
| GOTERM_BP_DIRECT | GO:0006928~movement of cell or subcellular component | 8 | 0.977 | 0.09638202 | GNA13, PALM, MTSS1, TLN1, PTGS2, RAC1, ANOS1, STAT3 | 765 | 86 | 16792 | 2.041890865 | 1 | 0.874157421 | 84.44542928 |
| GOTERM_BP_DIRECT | GO:0090263~positive regulation of canonical Wnt signaling pathway | 10 | 1.221 | 0.09655102 | FGFR2, TRPM4, XIAP, SULF2, SFRP1, SFRP2, SMURF2, YAP1, GPRC5B, DAPK3 | 765 | 120 | 16792 | 1.8291939 | 1 | 0.873150045 | 84.49875677 |
| GOTERM_BP_DIRECT | GO:0030855~epithelial cell differentiation | 7 | 0.855 | 0.09844064 | FGFR2, HNRNPH3, COL4A1, RHCG, CNN3, PPARG, CPT1A | 765 | 70 | 16792 | 2.19503268 | 1 | 0.876981603 | 85.08335221 |
| GOTERM_BP_DIRECT | GO:0071300~cellular response to retinoic acid | 7 | 0.855 | 0.09844064 | ADNP2, PPARG, PTK7, YAP1, ABCA1, SOX9, MYC | 765 | 70 | 16792 | 2.19503268 | 1 | 0.876981603 | 85.08335221 |
| GOTERM_BP_DIRECT | GO:0030199~collagen fibril organization | 5 | 0.611 | 0.09982931 | SFRP2, COL12A1, LOXL2, COL11A1, COL5A1 | 765 | 39 | 16792 | 2.814144461 | 1 | 0.879323727 | 85.49961465 |
