# Supplementary material for: Generation and characterization of keap1a- and keap1b-knockout zebrafish
Source: Redox Biol. 2020 Aug 11;36:101667. doi: 10.1016/j.redox.2020.101667 (PMC7452054; doi:10.1016/j.redox.2020.101667)
Supplement: Multimedia component 5 [file mmc5.docx]

Table S2. Biological processes up-regulated by *keap1a* disruption.

| Category | Term | Count | % | P Value | Genes | List Total | Pop Hits | Pop Total | Fold Enrichment | Bonferroni | Benjamini | FDR |
| --- | --- | --- | --- | --- | --- | --- | --- | --- | --- | --- | --- | --- |
| GOTERM_BP_DIRECT | GO:0055114~oxidation-reduction process | 25 | 21.93 | 2.78E-13 | C15ORF48, FTMT, PTGR1, HTATIP2, CYP2C9, CYP2C18, AIFM1, CYP2C8, PGD, UGDH, RPE65, CBR3, PRDX1, CYB561A3, FTH1, DHDH, SOD3, DHRS2, GSR, CPOX, TXNRD3, GSTO1, TSTA3, CP, MGST1 | 108 | 592 | 16792 | 6.565940941 | 2.08E-10 | 2.08E-10 | 4.22E-10 |
| GOTERM_BP_DIRECT | GO:0006805~xenobiotic metabolic process | 7 | 6.14 | 1.03E-05 | CES1, CYP2C9, CYP2C18, CYP2C8, MGST1, GSTP1, CMBL | 108 | 78 | 16792 | 13.95346629 | 0.007668215 | 0.003841486 | 0.015656183 |
| GOTERM_BP_DIRECT | GO:0006749~glutathione metabolic process | 5 | 4.386 | 4.44E-04 | GSR, ETHE1, GSTO1, MGST1, GSTP1 | 108 | 56 | 16792 | 13.88227513 | 0.282438343 | 0.104731973 | 0.672810177 |
| GOTERM_BP_DIRECT | GO:0098869~cellular oxidant detoxification | 5 | 4.386 | 0.00103439 | GSR, TXNRD3, GSTO1, MGST1, GSTP1 | 108 | 70 | 16792 | 11.10582011 | 0.538893894 | 0.175956348 | 1.562257749 |
| GOTERM_BP_DIRECT | GO:0045454~cell redox homeostasis | 5 | 4.386 | 0.00147559 | GSR, GCLC, AIFM1, TXNRD3, PRDX1 | 108 | 77 | 16792 | 10.0962001 | 0.668641651 | 0.198211942 | 2.221636071 |
| GOTERM_BP_DIRECT | GO:0007601~visual perception | 7 | 6.14 | 0.00181183 | CRYGD, LAMB2, LRAT, RBP3, GRK7, RPE65, CABP4 | 108 | 201 | 16792 | 5.414777962 | 0.742433332 | 0.202345273 | 2.72138452 |
| GOTERM_BP_DIRECT | GO:0000302~response to reactive oxygen species | 4 | 3.509 | 0.00194574 | GSR, PRDX1, GSTP1, SOD3 | 108 | 39 | 16792 | 15.94681861 | 0.7670262 | 0.187889178 | 2.919746261 |
| GOTERM_BP_DIRECT | GO:0009636~response to toxic substance | 5 | 4.386 | 0.00212364 | DHRS2, CES1, AIFM1, SLC22A8, GSTP1 | 108 | 85 | 16792 | 9.145969499 | 0.79610927 | 0.180262723 | 3.182690388 |
| GOTERM_BP_DIRECT | GO:0019430~removal of superoxide radicals | 3 | 2.632 | 0.00254654 | APOA4, PRDX1, SOD3 | 108 | 12 | 16792 | 38.87037037 | 0.851509996 | 0.190966775 | 3.80507508 |
| GOTERM_BP_DIRECT | GO:0019373~epoxygenase P450 pathway | 3 | 2.632 | 0.00575806 | CYP2C9, CYP2C18, CYP2C8 | 108 | 18 | 16792 | 25.91358025 | 0.986693174 | 0.350756743 | 8.411001667 |
| GOTERM_BP_DIRECT | GO:0001523~retinoid metabolic process | 4 | 3.509 | 0.00692533 | APOA4, LRAT, RBP3, RPE65 | 108 | 61 | 16792 | 10.19550698 | 0.994473307 | 0.376596009 | 10.03340053 |
| GOTERM_BP_DIRECT | GO:1901687~glutathione derivative biosynthetic process | 3 | 2.632 | 0.00855068 | GSTO1, MGST1, GSTP1 | 108 | 22 | 16792 | 21.2020202 | 0.998376889 | 0.414497146 | 12.24781507 |
| GOTERM_BP_DIRECT | GO:0009058~biosynthetic process | 3 | 2.632 | 0.0155786 | ALAS1, KYAT3, PCYT1B | 108 | 30 | 16792 | 15.54814815 | 0.999992067 | 0.594820015 | 21.24950615 |
| GOTERM_BP_DIRECT | GO:0006270~DNA replication initiation | 3 | 2.632 | 0.01761742 | MCM2, MCM4, MCM5 | 108 | 32 | 16792 | 14.57638889 | 0.999998318 | 0.613128855 | 23.69477707 |
| GOTERM_BP_DIRECT | GO:0043124~negative regulation of I-kappaB kinase/NF-kappaB signaling | 3 | 2.632 | 0.0268088 | ZC3H12A, ABL1, GSTP1 | 108 | 40 | 16792 | 11.66111111 | 0.999999999 | 0.74208165 | 33.86345371 |
| GOTERM_BP_DIRECT | GO:0010828~positive regulation of glucose transport | 2 | 1.754 | 0.03146065 | GIP, C3 | 108 | 5 | 16792 | 62.19259259 | 1 | 0.775620959 | 38.51323385 |
| GOTERM_BP_DIRECT | GO:0006879~cellular iron ion homeostasis | 3 | 2.632 | 0.03198576 | FTMT, CP, FTH1 | 108 | 44 | 16792 | 10.6010101 | 1 | 0.760780392 | 39.0184736 |
| GOTERM_BP_DIRECT | GO:0006880~intracellular sequestering of iron ion | 2 | 1.754 | 0.0376341 | FTMT, FTH1 | 108 | 6 | 16792 | 51.82716049 | 1 | 0.796907561 | 44.21333372 |
| GOTERM_BP_DIRECT | GO:0010951~negative regulation of endopeptidase activity | 4 | 3.509 | 0.04219606 | SERPINA9, C3, SPINK4, SERPINH1 | 108 | 121 | 16792 | 5.139883685 | 1 | 0.816814898 | 48.10401036 |
| GOTERM_BP_DIRECT | GO:0043065~positive regulation of apoptotic process | 6 | 5.263 | 0.04294359 | AIFM1, TGM2, SYCE3, FAM162A, ABL1, DNM2 | 108 | 300 | 16792 | 3.10962963 | 1 | 0.806329295 | 48.71683391 |
| GOTERM_BP_DIRECT | GO:0006776~vitamin A metabolic process | 2 | 1.754 | 0.04376857 | LRAT, RPE65 | 108 | 7 | 16792 | 44.42328042 | 1 | 0.796917234 | 49.38530297 |
| GOTERM_BP_DIRECT | GO:1903351~cellular response to dopamine | 2 | 1.754 | 0.0498643 | ABL1, DNM2 | 108 | 8 | 16792 | 38.87037037 | 1 | 0.824324302 | 54.07804716 |
| GOTERM_BP_DIRECT | GO:0032872~regulation of stress-activated MAPK cascade | 2 | 1.754 | 0.0498643 | PRDX1, GSTP1 | 108 | 8 | 16792 | 38.87037037 | 1 | 0.824324302 | 54.07804716 |
| GOTERM_BP_DIRECT | GO:0032922~circadian regulation of gene expression | 3 | 2.632 | 0.05117625 | CRY2, PER2, CARTPT | 108 | 57 | 16792 | 8.183235867 | 1 | 0.81885167 | 55.03337453 |
| GOTERM_BP_DIRECT | GO:0060316~positive regulation of ryanodine-sensitive calcium-release channel activity | 2 | 1.754 | 0.05592153 | TRDN, GSTO1 | 108 | 9 | 16792 | 34.55144033 | 1 | 0.833625926 | 58.33594564 |
| GOTERM_BP_DIRECT | GO:0006782~protoporphyrinogen IX biosynthetic process | 2 | 1.754 | 0.05592153 | ALAS1, CPOX | 108 | 9 | 16792 | 34.55144033 | 1 | 0.833625926 | 58.33594564 |
| GOTERM_BP_DIRECT | GO:0019852~L-ascorbic acid metabolic process | 2 | 1.754 | 0.05592153 | GCLC, GSTO1 | 108 | 9 | 16792 | 34.55144033 | 1 | 0.833625926 | 58.33594564 |
| GOTERM_BP_DIRECT | GO:0032930~positive regulation of superoxide anion generation | 2 | 1.754 | 0.05592153 | CRP, GSTP1 | 108 | 9 | 16792 | 34.55144033 | 1 | 0.833625926 | 58.33594564 |
| GOTERM_BP_DIRECT | GO:0042754~negative regulation of circadian rhythm | 2 | 1.754 | 0.05592153 | CRY2, PER2 | 108 | 9 | 16792 | 34.55144033 | 1 | 0.833625926 | 58.33594564 |
| GOTERM_BP_DIRECT | GO:0097267~omega-hydroxylase P450 pathway | 2 | 1.754 | 0.05592153 | CYP2C9, CYP2C8 | 108 | 9 | 16792 | 34.55144033 | 1 | 0.833625926 | 58.33594564 |
| GOTERM_BP_DIRECT | GO:0006268~DNA unwinding involved in DNA replication | 2 | 1.754 | 0.0619405 | MCM2, MCM4 | 108 | 10 | 16792 | 31.0962963 | 1 | 0.852384079 | 62.19927025 |
| GOTERM_BP_DIRECT | GO:0010884~positive regulation of lipid storage | 2 | 1.754 | 0.0619405 | C3, ZC3H12A | 108 | 10 | 16792 | 31.0962963 | 1 | 0.852384079 | 62.19927025 |
| GOTERM_BP_DIRECT | GO:0034599~cellular response to oxidative stress | 3 | 2.632 | 0.06283328 | DHRS2, ZC3H12A, ABL1 | 108 | 64 | 16792 | 7.288194444 | 1 | 0.845405981 | 62.74294241 |
| GOTERM_BP_DIRECT | GO:0006098~pentose-phosphate shunt | 2 | 1.754 | 0.06792145 | TALDO1, PGD | 108 | 11 | 16792 | 28.26936027 | 1 | 0.857530636 | 65.70456524 |
| GOTERM_BP_DIRECT | GO:0050796~regulation of insulin secretion | 3 | 2.632 | 0.06807926 | GIP, PER2, CARTPT | 108 | 67 | 16792 | 6.96185738 | 1 | 0.847951822 | 65.79280364 |
| GOTERM_BP_DIRECT | GO:0042738~exogenous drug catabolic process | 2 | 1.754 | 0.07386462 | CYP2C9, CYP2C8 | 108 | 12 | 16792 | 25.91358025 | 1 | 0.861824437 | 68.88499216 |
| GOTERM_BP_DIRECT | GO:0070989~oxidative demethylation | 2 | 1.754 | 0.07386462 | CYP2C9, CYP2C8 | 108 | 12 | 16792 | 25.91358025 | 1 | 0.861824437 | 68.88499216 |
| GOTERM_BP_DIRECT | GO:0060315~negative regulation of ryanodine-sensitive calcium-release channel activity | 2 | 1.754 | 0.07386462 | TRDN, GSTO1 | 108 | 12 | 16792 | 25.91358025 | 1 | 0.861824437 | 68.88499216 |
| GOTERM_BP_DIRECT | GO:0061014~positive regulation of mRNA catabolic process | 2 | 1.754 | 0.07386462 | KHSRP, ZC3H12A | 108 | 12 | 16792 | 25.91358025 | 1 | 0.861824437 | 68.88499216 |
| GOTERM_BP_DIRECT | GO:0061158~3'-UTR-mediated mRNA destabilization | 2 | 1.754 | 0.07386462 | KHSRP, ZC3H12A | 108 | 12 | 16792 | 25.91358025 | 1 | 0.861824437 | 68.88499216 |
| GOTERM_BP_DIRECT | GO:0006826~iron ion transport | 2 | 1.754 | 0.07386462 | FTMT, FTH1 | 108 | 12 | 16792 | 25.91358025 | 1 | 0.861824437 | 68.88499216 |
| GOTERM_BP_DIRECT | GO:1902358~sulfate transmembrane transport | 2 | 1.754 | 0.07386462 | SLC26A3, SLC13A1 | 108 | 12 | 16792 | 25.91358025 | 1 | 0.861824437 | 68.88499216 |
| GOTERM_BP_DIRECT | GO:0006260~DNA replication | 4 | 3.509 | 0.0766004 | MCM2, MCM4, CHAF1B, MCM5 | 108 | 155 | 16792 | 4.012425329 | 1 | 0.862896226 | 70.25441568 |
| GOTERM_BP_DIRECT | GO:0030516~regulation of axon extension | 2 | 1.754 | 0.07977026 | ABL1, DNM2 | 108 | 13 | 16792 | 23.92022792 | 1 | 0.865459865 | 71.77064276 |
| GOTERM_BP_DIRECT | GO:0045019~negative regulation of nitric oxide biosynthetic process | 2 | 1.754 | 0.07977026 | KHSRP, ZC3H12A | 108 | 13 | 16792 | 23.92022792 | 1 | 0.865459865 | 71.77064276 |
| GOTERM_BP_DIRECT | GO:0046685~response to arsenic-containing substance | 2 | 1.754 | 0.07977026 | GCLC, CPOX | 108 | 13 | 16792 | 23.92022792 | 1 | 0.865459865 | 71.77064276 |
| GOTERM_BP_DIRECT | GO:0007623~circadian rhythm | 3 | 2.632 | 0.08273105 | CRY2, PER2, RPE65 | 108 | 75 | 16792 | 6.219259259 | 1 | 0.867150409 | 73.12136346 |
| GOTERM_BP_DIRECT | GO:0071732~cellular response to nitric oxide | 2 | 1.754 | 0.08563858 | AIFM1, DNM2 | 108 | 14 | 16792 | 22.21164021 | 1 | 0.868576728 | 74.388823 |
| GOTERM_BP_DIRECT | GO:0008152~metabolic process | 4 | 3.509 | 0.0922056 | CES1, UGT1A5, GSTO1, GSTP1 | 108 | 168 | 16792 | 3.701940035 | 1 | 0.880951202 | 77.04896535 |
| GOTERM_BP_DIRECT | GO:0005975~carbohydrate metabolic process | 4 | 3.509 | 0.09981993 | TALDO1, SI, UGDH, DHDH | 108 | 174 | 16792 | 3.574286931 | 1 | 0.894329463 | 79.80954363 |
