# Supplementary material for: Generation and characterization of keap1a- and keap1b-knockout zebrafish
Source: Redox Biol. 2020 Aug 11;36:101667. doi: 10.1016/j.redox.2020.101667 (PMC7452054; doi:10.1016/j.redox.2020.101667)
Supplement: Multimedia component 6 [file mmc6.docx]

Table S3. Biological processes up-regulated by *keap1b* disruption.

| Category | Term | Count | % | P Value | Genes | List Total | Pop Hits | Pop Total | Fold Enrichment | Bonferroni | Benjamini | FDR |
| --- | --- | --- | --- | --- | --- | --- | --- | --- | --- | --- | --- | --- |
| GOTERM_BP_DIRECT | GO:0055114~oxidation-reduction process | 18 | 21.4 | 1.67E-09 | C15ORF48, FTMT, PTGR1, CYP2C8, HGD, RPE65, CBR3, PRDX1, CYB561A3, DHDH, SOD3, FTH1, DHRS2, GPX5, CYBRD1, TSTA3, CP, HPD | 80 | 592 | 16792 | 6.38209459 | 8.53E-07 | 8.53E-07 | 2.42E-06 |
| GOTERM_BP_DIRECT | GO:0007601~visual perception | 7 | 8.33 | 3.67E-04 | CRYGB, CRYGC, CRYGD, LRAT, GRK7, RPE65, CABP4 | 80 | 201 | 16792 | 7.30995025 | 0.17080803 | 0.08940021 | 0.52894098 |
| GOTERM_BP_DIRECT | GO:0032720~negative regulation of tumor necrosis factor production | 4 | 4.76 | 7.51E-04 | TSPO, ACP5, ZC3H12A, GSTP1 | 80 | 38 | 16792 | 22.0947368 | 0.31825922 | 0.11988432 | 1.07889101 |
| GOTERM_BP_DIRECT | GO:0000302~response to reactive oxygen species | 4 | 4.76 | 8.11E-04 | GPX5, PRDX1, GSTP1, SOD3 | 80 | 39 | 16792 | 21.5282051 | 0.33876343 | 0.09824364 | 1.16438818 |
| GOTERM_BP_DIRECT | GO:0006879~cellular iron ion homeostasis | 4 | 4.76 | 0.001155 | FTMT, CYBRD1, CP, FTH1 | 80 | 44 | 16792 | 19.0818182 | 0.44539896 | 0.11121619 | 1.655317 |
| GOTERM_BP_DIRECT | GO:0045019~negative regulation of nitric oxide biosynthetic process | 3 | 3.57 | 0.001648 | TSPO, ACP5, ZC3H12A | 80 | 13 | 16792 | 48.4384615 | 0.56885991 | 0.13083332 | 2.35403023 |
| GOTERM_BP_DIRECT | GO:0006805~xenobiotic metabolic process | 4 | 4.76 | 0.005919 | CES1, CYP2C8, GSTP1, CMBL | 80 | 78 | 16792 | 10.7641026 | 0.9515604 | 0.35110936 | 8.21497648 |
| GOTERM_BP_DIRECT | GO:0009636~response to toxic substance | 4 | 4.76 | 0.007506 | DHRS2, CES1, PON2, GSTP1 | 80 | 85 | 16792 | 9.87764706 | 0.97855978 | 0.38140891 | 10.3089257 |
| GOTERM_BP_DIRECT | GO:0009058~biosynthetic process | 3 | 3.57 | 0.008729 | ALAS1, KYAT3, PCYT1B | 80 | 30 | 16792 | 20.99 | 0.98856841 | 0.39153715 | 11.8919004 |
| GOTERM_BP_DIRECT | GO:0043200~response to amino acid | 3 | 3.57 | 0.009303 | GIP, SST, GSTP1 | 80 | 31 | 16792 | 20.3129032 | 0.99149116 | 0.37914951 | 12.625469 |
| GOTERM_BP_DIRECT | GO:0010940~positive regulation of necrotic cell death | 2 | 2.38 | 0.018688 | TSPO, HEBP2 | 80 | 4 | 16792 | 104.95 | 0.99993367 | 0.58298394 | 23.8461158 |
| GOTERM_BP_DIRECT | GO:0006572~tyrosine catabolic process | 2 | 2.38 | 0.023306 | HGD, HPD | 80 | 5 | 16792 | 83.96 | 0.99999402 | 0.63293295 | 28.8605245 |
| GOTERM_BP_DIRECT | GO:0006880~intracellular sequestering of iron ion | 2 | 2.38 | 0.027902 | FTMT, FTH1 | 80 | 6 | 16792 | 69.9666667 | 0.99999946 | 0.67049847 | 33.5450259 |
| GOTERM_BP_DIRECT | GO:0006776~vitamin A metabolic process | 2 | 2.38 | 0.032477 | LRAT, RPE65 | 80 | 7 | 16792 | 59.9714286 | 0.99999995 | 0.69962534 | 37.9213077 |
| GOTERM_BP_DIRECT | GO:0034599~cellular response to oxidative stress | 3 | 3.57 | 0.036513 | DHRS2, GPX5, ZC3H12A | 80 | 64 | 16792 | 9.8390625 | 0.99999999 | 0.71767246 | 41.558213 |
| GOTERM_BP_DIRECT | GO:0032872~regulation of stress-activated MAPK cascade | 2 | 2.38 | 0.037031 | PRDX1, GSTP1 | 80 | 8 | 16792 | 52.475 | 1 | 0.69963613 | 42.0096326 |
| GOTERM_BP_DIRECT | GO:0042754~negative regulation of circadian rhythm | 2 | 2.38 | 0.041563 | CRY2, PER2 | 80 | 9 | 16792 | 46.6444444 | 1 | 0.72016336 | 45.8289325 |
| GOTERM_BP_DIRECT | GO:0032930~positive regulation of superoxide anion generation | 2 | 2.38 | 0.041563 | CRP, GSTP1 | 80 | 9 | 16792 | 46.6444444 | 1 | 0.72016336 | 45.8289325 |
| GOTERM_BP_DIRECT | GO:0032691~negative regulation of interleukin-1 beta production | 2 | 2.38 | 0.046075 | ACP5, GSTP1 | 80 | 10 | 16792 | 41.98 | 1 | 0.73723114 | 49.3968957 |
| GOTERM_BP_DIRECT | GO:0007623~circadian rhythm | 3 | 3.57 | 0.048634 | CRY2, PER2, RPE65 | 80 | 75 | 16792 | 8.396 | 1 | 0.73769522 | 51.3223548 |
| GOTERM_BP_DIRECT | GO:0005975~carbohydrate metabolic process | 4 | 4.76 | 0.048812 | TALDO1, LCTL, DHDH, MDH1 | 80 | 174 | 16792 | 4.82528736 | 1 | 0.72087755 | 51.4536382 |
| GOTERM_BP_DIRECT | GO:0006559~L-phenylalanine catabolic process | 2 | 2.38 | 0.050565 | HGD, HPD | 80 | 11 | 16792 | 38.1636364 | 1 | 0.7163879 | 52.7300482 |
| GOTERM_BP_DIRECT | GO:0006826~iron ion transport | 2 | 2.38 | 0.055035 | FTMT, FTH1 | 80 | 12 | 16792 | 34.9833333 | 1 | 0.73078884 | 55.8438306 |
| GOTERM_BP_DIRECT | GO:0043206~extracellular fibril organization | 2 | 2.38 | 0.055035 | MFAP4, MFAP5 | 80 | 12 | 16792 | 34.9833333 | 1 | 0.73078884 | 55.8438306 |
| GOTERM_BP_DIRECT | GO:0010447~response to acidic pH | 2 | 2.38 | 0.055035 | GIP, SST | 80 | 12 | 16792 | 34.9833333 | 1 | 0.73078884 | 55.8438306 |
| GOTERM_BP_DIRECT | GO:0019430~removal of superoxide radicals | 2 | 2.38 | 0.055035 | PRDX1, SOD3 | 80 | 12 | 16792 | 34.9833333 | 1 | 0.73078884 | 55.8438306 |
| GOTERM_BP_DIRECT | GO:0042493~response to drug | 5 | 5.95 | 0.055084 | TSPO, GIP, XPC, SPINK4, SST | 80 | 304 | 16792 | 3.45230263 | 1 | 0.71531054 | 55.87694 |
| GOTERM_BP_DIRECT | GO:0050830~defense response to Gram-positive bacterium | 3 | 3.57 | 0.060731 | PGLYRP2, CRP, ACP5 | 80 | 85 | 16792 | 7.40823529 | 1 | 0.73588611 | 59.535297 |
| GOTERM_BP_DIRECT | GO:0006783~heme biosynthetic process | 2 | 2.38 | 0.094338 | ALAS1, TSPO | 80 | 21 | 16792 | 19.9904762 | 1 | 0.86753311 | 76.090643 |
| GOTERM_BP_DIRECT | GO:0071222~cellular response to lipopolysaccharide | 3 | 3.57 | 0.099093 | TSPO, ZC3H12A, GSTP1 | 80 | 113 | 16792 | 5.57256637 | 1 | 0.87086862 | 77.8406517 |
