# Supplementary material for: Generation and characterization of keap1a- and keap1b-knockout zebrafish
Source: Redox Biol. 2020 Aug 11;36:101667. doi: 10.1016/j.redox.2020.101667 (PMC7452054; doi:10.1016/j.redox.2020.101667)
Supplement: Multimedia component 7 [file mmc7.docx]

Table S7. Biological processes down-regulated by sulforaphane treatment.

| Category | Term | Count | % | P Value | Genes | List Total | Pop Hits | Pop Total | Fold Enrichment | Bonferroni | Benjamini | FDR |
| --- | --- | --- | --- | --- | --- | --- | --- | --- | --- | --- | --- | --- |
| GOTERM_BP_DIRECT | GO:0042632~cholesterol homeostasis | 5 | 7.69 | 7.46E-05 | APOA4, APOB, NPC2, DGAT2, ANGPTL3 | 60 | 64 | 16792 | 21.8645833 | 0.03791735 | 0.03791735 | 0.10793677 |
| GOTERM_BP_DIRECT | GO:0006631~fatty acid metabolic process | 4 | 6.15 | 8.06E-04 | MSMO1, CREM, PER2, ANGPTL3 | 60 | 52 | 16792 | 21.5282051 | 0.34132045 | 0.18840925 | 1.15969559 |
| GOTERM_BP_DIRECT | GO:0008203~cholesterol metabolic process | 4 | 6.15 | 0.001756 | APOA4, APOB, NPC2, ANGPTL3 | 60 | 68 | 16792 | 16.4627451 | 0.59759467 | 0.26171976 | 2.51113878 |
| GOTERM_BP_DIRECT | GO:0019915~lipid storage | 3 | 4.62 | 0.003187 | DGAT2, ANGPTL3, CRY1 | 60 | 24 | 16792 | 34.9833333 | 0.80864633 | 0.3386076 | 4.5148623 |
| GOTERM_BP_DIRECT | GO:0033344~cholesterol efflux | 3 | 4.62 | 0.003457 | APOA4, APOB, NPC2 | 60 | 25 | 16792 | 33.584 | 0.83364342 | 0.30143311 | 4.88758203 |
| GOTERM_BP_DIRECT | GO:0006270~DNA replication initiation | 3 | 4.62 | 0.005626 | MCM3, MCM5, MCM6 | 60 | 32 | 16792 | 26.2375 | 0.94618814 | 0.38555974 | 7.83989636 |
| GOTERM_BP_DIRECT | GO:0006094~gluconeogenesis | 3 | 4.62 | 0.010444 | PER2, SLC25A1, CRY1 | 60 | 44 | 16792 | 19.0818182 | 0.99565379 | 0.54018058 | 14.0960834 |
| GOTERM_BP_DIRECT | GO:0042752~regulation of circadian rhythm | 3 | 4.62 | 0.012839 | CREM, PER2, CRY1 | 60 | 49 | 16792 | 17.1346939 | 0.99876098 | 0.5668534 | 17.055856 |
| GOTERM_BP_DIRECT | GO:0032922~circadian regulation of gene expression | 3 | 4.62 | 0.017115 | CREM, PER2, CRY1 | 60 | 57 | 16792 | 14.7298246 | 0.99986923 | 0.62974611 | 22.1064094 |
| GOTERM_BP_DIRECT | GO:0001523~retinoid metabolic process | 3 | 4.62 | 0.019449 | APOA4, DHRS3, APOB | 60 | 61 | 16792 | 13.7639344 | 0.99996185 | 0.63846533 | 24.7416242 |
| GOTERM_BP_DIRECT | GO:0010216~maintenance of DNA methylation | 2 | 3.08 | 0.0209 | UHRF1, HELLS | 60 | 6 | 16792 | 93.2888889 | 0.99998228 | 0.63014416 | 26.3373342 |
| GOTERM_BP_DIRECT | GO:0034383~low-density lipoprotein particle clearance | 2 | 3.08 | 0.027771 | APOB, DGAT2 | 60 | 8 | 16792 | 69.9666667 | 0.99999954 | 0.70351126 | 33.4748271 |
| GOTERM_BP_DIRECT | GO:0046903~secretion | 2 | 3.08 | 0.027771 | CA2, TPD52 | 60 | 8 | 16792 | 69.9666667 | 0.99999954 | 0.70351126 | 33.4748271 |
| GOTERM_BP_DIRECT | GO:2000001~regulation of DNA damage checkpoint | 2 | 3.08 | 0.027771 | WDR76, CRY1 | 60 | 8 | 16792 | 69.9666667 | 0.99999954 | 0.70351126 | 33.4748271 |
| GOTERM_BP_DIRECT | GO:0042158~lipoprotein biosynthetic process | 2 | 3.08 | 0.031189 | APOA4, APOB | 60 | 9 | 16792 | 62.1925926 | 0.99999993 | 0.71706644 | 36.7801704 |
| GOTERM_BP_DIRECT | GO:0042754~negative regulation of circadian rhythm | 2 | 3.08 | 0.031189 | PER2, CRY1 | 60 | 9 | 16792 | 62.1925926 | 0.99999993 | 0.71706644 | 36.7801704 |
| GOTERM_BP_DIRECT | GO:0010884~positive regulation of lipid storage | 2 | 3.08 | 0.034595 | APOB, ZC3H12A | 60 | 10 | 16792 | 55.9733333 | 0.99999999 | 0.72819294 | 39.9214685 |
| GOTERM_BP_DIRECT | GO:0006071~glycerol metabolic process | 2 | 3.08 | 0.037989 | DGAT2, ANGPTL3 | 60 | 11 | 16792 | 50.8848485 | 1 | 0.73748325 | 42.9068539 |
| GOTERM_BP_DIRECT | GO:0000082~G1/S transition of mitotic cell cycle | 3 | 4.62 | 0.049979 | MCM3, MCM5, MCM6 | 60 | 102 | 16792 | 8.23137255 | 1 | 0.80984 | 52.3834808 |
| GOTERM_BP_DIRECT | GO:0042953~lipoprotein transport | 2 | 3.08 | 0.051448 | APOB, PRKCB | 60 | 15 | 16792 | 37.3155556 | 1 | 0.79999479 | 53.4382359 |
| GOTERM_BP_DIRECT | GO:0030301~cholesterol transport | 2 | 3.08 | 0.054784 | APOB, NPC2 | 60 | 16 | 16792 | 34.9833333 | 1 | 0.80237457 | 55.7526277 |
| GOTERM_BP_DIRECT | GO:0055114~oxidation-reduction process | 6 | 9.23 | 0.056476 | DHRS3, CYP2J2, MSMO1, CYP27A1, IDO2, CYP2A7 | 60 | 592 | 16792 | 2.83648649 | 1 | 0.79503287 | 56.8854747 |
| GOTERM_BP_DIRECT | GO:0071356~cellular response to tumor necrosis factor | 3 | 4.62 | 0.057149 | APOB, SFRP1, ZC3H12A | 60 | 110 | 16792 | 7.63272727 | 1 | 0.78219351 | 57.3285724 |
| GOTERM_BP_DIRECT | GO:0019373~epoxygenase P450 pathway | 2 | 3.08 | 0.061421 | CYP2J2, CYP2A7 | 60 | 18 | 16792 | 31.0962963 | 1 | 0.7906125 | 60.042378 |
| GOTERM_BP_DIRECT | GO:0045766~positive regulation of angiogenesis | 3 | 4.62 | 0.061803 | ZC3H12A, ANGPTL3, PRKCB | 60 | 115 | 16792 | 7.30086957 | 1 | 0.77733586 | 60.2772939 |
| GOTERM_BP_DIRECT | GO:0048844~artery morphogenesis | 2 | 3.08 | 0.07129 | APOB, ANGPTL3 | 60 | 21 | 16792 | 26.6539683 | 1 | 0.81093979 | 65.7107537 |
| GOTERM_BP_DIRECT | GO:0007399~nervous system development | 4 | 6.15 | 0.079929 | GDA, APOB, PCDHA8, ZC3H12A | 60 | 287 | 16792 | 3.90058072 | 1 | 0.83436682 | 70.0481066 |
| GOTERM_BP_DIRECT | GO:0070328~triglyceride homeostasis | 2 | 3.08 | 0.087514 | APOA4, ANGPTL3 | 60 | 26 | 16792 | 21.5282051 | 1 | 0.85006947 | 73.4297697 |
| GOTERM_BP_DIRECT | GO:0046627~negative regulation of insulin receptor signaling pathway | 2 | 3.08 | 0.097114 | SOCS3, PRKCB | 60 | 29 | 16792 | 19.3011494 | 1 | 0.86936033 | 77.2006816 |
| GOTERM_BP_DIRECT | GO:0042149~cellular response to glucose starvation | 2 | 3.08 | 0.097114 | SLC2A1, ZC3H12A | 60 | 29 | 16792 | 19.3011494 | 1 | 0.86936033 | 77.2006816 |
