# Supplementary material for: Generation and characterization of keap1a- and keap1b-knockout zebrafish
Source: Redox Biol. 2020 Aug 11;36:101667. doi: 10.1016/j.redox.2020.101667 (PMC7452054; doi:10.1016/j.redox.2020.101667)
Supplement: Multimedia component 8 [file mmc8.docx]

Table S8. Biological processes up-regulated by *keap1a* disruption, but not by *keap1b* disruption.

| Category | Term | Count | % | P Value | Genes | List Total | Pop Hits | Pop Total | Fold Enrichment | Bonferroni | Benjamini | FDR |
| --- | --- | --- | --- | --- | --- | --- | --- | --- | --- | --- | --- | --- |
| GOTERM_BP_DIRECT | GO:0055114~oxidation-reduction process | 11 | 20.8 | 7.91E-06 | GSR, HTATIP2, CYP2C9, CYP2C18, AIFM1, CPOX, PGD, UGDH, TXNRD3, GSTO1, MGST1 | 51 | 592 | 16792 | 6.11791203 | 0.00371642 | 0.00371642 | 0.0112875 |
| GOTERM_BP_DIRECT | GO:0098869~cellular oxidant detoxification | 4 | 7.55 | 0.001182 | GSR, TXNRD3, GSTO1, MGST1 | 51 | 70 | 16792 | 18.8145658 | 0.4270297 | 0.24305198 | 1.67425537 |
| GOTERM_BP_DIRECT | GO:0045454~cell redox homeostasis | 4 | 7.55 | 0.001556 | GSR, GCLC, AIFM1, TXNRD3 | 51 | 77 | 16792 | 17.1041508 | 0.51980564 | 0.21692081 | 2.19941366 |
| GOTERM_BP_DIRECT | GO:0006270~DNA replication initiation | 3 | 5.66 | 0.004071 | MCM2, MCM4, MCM5 | 51 | 32 | 16792 | 30.8676471 | 0.85358036 | 0.38141436 | 5.65838372 |
| GOTERM_BP_DIRECT | GO:0006260~DNA replication | 4 | 7.55 | 0.011011 | MCM2, MCM4, CHAF1B, MCM5 | 51 | 155 | 16792 | 8.4969007 | 0.99456656 | 0.64761696 | 14.6243888 |
| GOTERM_BP_DIRECT | GO:0043065~positive regulation of apoptotic process | 5 | 9.43 | 0.01207 | AIFM1, TGM2, FAM162A, ABL1, DNM2 | 51 | 300 | 16792 | 5.4875817 | 0.9967191 | 0.61452271 | 15.9201616 |
| GOTERM_BP_DIRECT | GO:0006749~glutathione metabolic process | 3 | 5.66 | 0.012078 | GSR, GSTO1, MGST1 | 51 | 56 | 16792 | 17.6386555 | 0.9967316 | 0.55852618 | 15.9298934 |
| GOTERM_BP_DIRECT | GO:0006805~xenobiotic metabolic process | 3 | 5.66 | 0.022597 | CYP2C9, CYP2C18, MGST1 | 51 | 78 | 16792 | 12.6636501 | 0.99997888 | 0.73962536 | 27.845947 |
| GOTERM_BP_DIRECT | GO:1903351~cellular response to dopamine | 2 | 3.77 | 0.023579 | ABL1, DNM2 | 51 | 8 | 16792 | 82.3137255 | 0.99998685 | 0.71313563 | 28.8746389 |
| GOTERM_BP_DIRECT | GO:0019852~L-ascorbic acid metabolic process | 2 | 3.77 | 0.026488 | GCLC, GSTO1 | 51 | 9 | 16792 | 73.167756 | 0.99999677 | 0.71758927 | 31.8410991 |
| GOTERM_BP_DIRECT | GO:0006268~DNA unwinding involved in DNA replication | 2 | 3.77 | 0.029388 | MCM2, MCM4 | 51 | 10 | 16792 | 65.8509804 | 0.99999921 | 0.72118381 | 34.6840018 |
| GOTERM_BP_DIRECT | GO:1902358~sulfate transmembrane transport | 2 | 3.77 | 0.035163 | SLC26A3, SLC13A1 | 51 | 12 | 16792 | 54.875817 | 0.99999995 | 0.75463457 | 40.0194787 |
| GOTERM_BP_DIRECT | GO:0000082~G1/S transition of mitotic cell cycle | 3 | 5.66 | 0.037055 | MCM2, MCM4, MCM5 | 51 | 102 | 16792 | 9.6839677 | 0.99999998 | 0.74539032 | 41.6768036 |
| GOTERM_BP_DIRECT | GO:0030516~regulation of axon extension | 2 | 3.77 | 0.038038 | ABL1, DNM2 | 51 | 13 | 16792 | 50.6546003 | 0.99999999 | 0.72874283 | 42.5217002 |
| GOTERM_BP_DIRECT | GO:0046685~response to arsenic-containing substance | 2 | 3.77 | 0.038038 | GCLC, CPOX | 51 | 13 | 16792 | 50.6546003 | 0.99999999 | 0.72874283 | 42.5217002 |
| GOTERM_BP_DIRECT | GO:0071732~cellular response to nitric oxide | 2 | 3.77 | 0.040905 | AIFM1, DNM2 | 51 | 14 | 16792 | 47.0364146 | 1 | 0.73056222 | 44.9196761 |
| GOTERM_BP_DIRECT | GO:0019373~epoxygenase P450 pathway | 2 | 3.77 | 0.052288 | CYP2C9, CYP2C18 | 51 | 18 | 16792 | 36.583878 | 1 | 0.79421338 | 53.5532076 |
| GOTERM_BP_DIRECT | GO:0006508~proteolysis | 5 | 9.43 | 0.061118 | C3, RBP3, PRSS48, TMPRSS13, PROC | 51 | 500 | 16792 | 3.29254902 | 1 | 0.82575283 | 59.3647769 |
| GOTERM_BP_DIRECT | GO:1901687~glutathione derivative biosynthetic process | 2 | 3.77 | 0.063538 | GSTO1, MGST1 | 51 | 22 | 16792 | 29.9322638 | 1 | 0.82052922 | 60.8350763 |
| GOTERM_BP_DIRECT | GO:0071353~cellular response to interleukin-4 | 2 | 3.77 | 0.069114 | KEAP1, MCM2 | 51 | 24 | 16792 | 27.4379085 | 1 | 0.8305811 | 64.0365432 |
| GOTERM_BP_DIRECT | GO:0033344~cholesterol efflux | 2 | 3.77 | 0.07189 | APOA4, NPC2 | 51 | 25 | 16792 | 26.3403922 | 1 | 0.82742785 | 65.5378917 |
| GOTERM_BP_DIRECT | GO:0050766~positive regulation of phagocytosis | 2 | 3.77 | 0.082912 | C3, DNM2 | 51 | 29 | 16792 | 22.7072346 | 1 | 0.85647299 | 70.942889 |
| GOTERM_BP_DIRECT | GO:0051402~neuron apoptotic process | 2 | 3.77 | 0.093806 | AIFM1, FAM162A | 51 | 33 | 16792 | 19.9548425 | 1 | 0.87862029 | 75.5011716 |
