# Supplementary material for: Generation and characterization of keap1a- and keap1b-knockout zebrafish
Source: Redox Biol. 2020 Aug 11;36:101667. doi: 10.1016/j.redox.2020.101667 (PMC7452054; doi:10.1016/j.redox.2020.101667)
Supplement: Multimedia component 9 [file mmc9.docx]

Table S9. Biological processes down-regulated by *keap1a* disruption, but not by *keap1b* disruption.

| Category | Term | Count | % | P Value | Genes | List Total | Pop Hits | Pop Total | Fold Enrichment | Bonferroni | Benjamini | FDR |
| --- | --- | --- | --- | --- | --- | --- | --- | --- | --- | --- | --- | --- |
| GOTERM_BP_DIRECT | GO:0008016~regulation of heart contraction | 2 | 10 | 0.03273 | CYP2J2, MYH6 | 19 | 31 | 16792 | 57.0186757 | 0.98917357 | 0.98917357 | 32.3380719 |
| GOTERM_BP_DIRECT | GO:0046034~ATP metabolic process | 2 | 10 | 0.033769 | MYH4, MYH6 | 19 | 32 | 16792 | 55.2368421 | 0.99064549 | 0.90328129 | 33.1861679 |
| GOTERM_BP_DIRECT | GO:0030049~muscle filament sliding | 2 | 10 | 0.039979 | MYH4, MYH6 | 19 | 38 | 16792 | 46.5152355 | 0.99610815 | 0.84270365 | 38.0570926 |
| GOTERM_BP_DIRECT | GO:0046426~negative regulation of JAK-STAT cascade | 2 | 10 | 0.042041 | SOCS3, LEPROT | 19 | 40 | 16792 | 44.1894737 | 0.99709484 | 0.76783719 | 39.6008672 |
| GOTERM_BP_DIRECT | GO:0030509~BMP signaling pathway | 2 | 10 | 0.078446 | EGR1, MYH6 | 19 | 76 | 16792 | 23.2576177 | 0.99998504 | 0.89161544 | 61.6724897 |
