# Supplementary material for: Generation and characterization of keap1a- and keap1b-knockout zebrafish
Source: Redox Biol. 2020 Aug 11;36:101667. doi: 10.1016/j.redox.2020.101667 (PMC7452054; doi:10.1016/j.redox.2020.101667)
Supplement: Multimedia component 10 [file mmc10.docx]

**Table S10**. Relative expression of *prdx1.*

| Sulforaphane μM | 0 | 10 | 20 | 30 | 40 |
| --- | --- | --- | --- | --- | --- |
| WT | 1.00 ± 4.58E^-5^ | 5.52 ± 0.811 | 7.80 ± 1.16 | 11.5 ± 1.48 | 17.8 ± 3.28 |
| *keap1a-/-* | 3.90 ± 0.193 | 18.1 ± 0.795 | 25.2 ± 1.30 | 33.4 ± 1.46 | 44.8 ± 1.78 |
| *keap1b-/-* | 2.81 ± 9.85E^-2^ | 7.22 ± 0.402 | 8.76 ± 0.344 | 11.0 ± 0.725 | 12.7 ± 0.451 |
