# Supplementary material for: Generation and characterization of keap1a- and keap1b-knockout zebrafish
Source: Redox Biol. 2020 Aug 11;36:101667. doi: 10.1016/j.redox.2020.101667 (PMC7452054; doi:10.1016/j.redox.2020.101667)
Supplement: Multimedia component 11 [file mmc11.docx]

**Table S11**. Relative expression of *gstp1.*

| Sulforaphane μM | 0 | 10 | 20 | 30 | 40 |
| --- | --- | --- | --- | --- | --- |
| WT | 1.00 ± 6.53E^-4^ | 1.64 ± 0.130 | 2.13 ± 0.249 | 2.79 ± 0.378 | 4.43 ± 0.834 |
| *keap1a-/-* | 2.03 ± 0.193 | 5.29 ± 0.795 | 7.70 ± 1.30 | 10.0 ± 1.46 | 12.8 ± 1.78 |
| *keap1b-/-* | 1.60 ± 9.85E^-2^ | 2.33 ± 0.402 | 2.43 ± 0.344 | 2.50 ± 0.725 | 3.66 ± 0.451 |
